# ST6GAL1‐Mediated Sialylation Stabilizes PD‐L1 and Drives Immunosuppressive Tumor Microenvironment in Colorectal Cancer

**DOI:** 10.1002/advs.202406225

**Published:** 2025-08-22

**Authors:** Ximo Xu, Jingyi Liu, Wei Qin, Chengsheng Ding, Zhenghao Cai, Duohuo Shu, Hao Zhong, Yanyan Hu, Mengqin Yu, Leqi Zhou, Jianwen Li, Minhua Zheng, Bin Li, Sen Zhang, Xiao Yang, Bo Feng

**Affiliations:** ^1^ Department of General Surgery, Ruijin Hospital Shanghai JiaoTong University School of Medicine No.197, Ruijin Er Road Shanghai 200025 China; ^2^ Department of General Surgery, Shanghai General Hospital Shanghai Jiao Tong University School of Medicine No.80/90, Wujin Road Shanghai 200080 China; ^3^ Department of General Surgery Shanghai Changhai Hospital No.168, Changhai Road, Yangpu District Shanghai China; ^4^ Shanghai Institute of Immunology, Department of Immunology and Microbiology, Shanghai JiaoTong University School of Medicine Shanghai JiaoTong University No. 280, Chongqing Nan Road Shanghai 200031 China

**Keywords:** colorectal cancer, glycogenes, immunotherapy, sialylation, ST6GAL1

## Abstract

Abnormal glycogene expression is a recognized cancer hallmark, but its impact on the colorectal cancer (CRC) tumor microenvironment (TME) remains unclear. Utilizing bioinformatics analysis on TCGA and GEO datasets, a seven‐glycogene signature is identified for precise glycogene‐based classification in CRC. ST6GAL1, a key focus, emerges as a significant predictor of poor prognosis, with its upregulation linked to unfavorable outcomes in CRC. Functional experiments demonstrate that loss of ST6GAL1 inhibits CRC proliferation, migration, invasion, and metastasis. ST6GAL1‐mediated sialylation of PD‐L1 is critical for maintaining its stability in colorectal cancer cells, and ST6GAL1 knockdown leads to reduced protein stability and increased ubiquitination. ST6GAL1 knockdown in MC38 tumor‐bearing mice enhances the antitumor effect of anti‐PD‐L1 therapy, resulting in smaller tumor sizes and reduced tumor volume compared to control groups. Single‐cell analysis reveals ST6GAL1's influence on immune cell composition in the TME, particularly affecting CD8^+^ T cells. Taken together, ST6GAL1 is confirmed to act as an important regulating factor in CRC development through immune response and TME composition and has the potential to serve as a novel biomarker in CRC treatment.

## Graphical abstract

ST6GAL1 plays a critical role in tumor progression and immune regulation in colorectal cancer (CRC). Its depletion significantly suppresses the malignant phenotype of CRC cells and enhances the efficacy of anti‐PD‐L1 therapy. High ST6GAL1 expression promotes an immune‐activated tumor microenvironment (TME), characterized by increased infiltration of CD8^+^ T cells and M1 macrophages, thereby improving sensitivity to immunotherapy.

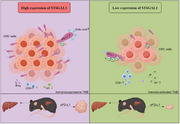

## 1 Introduction

Colorectal cancer (CRC) is the third most common malignancies worldwide, with ≈1 million new cases and 400 000 deaths annually.^[^ ^1^ ^]^ Although great advances have been made in surgical skills and adjuvant therapy techniques in recent decades, the clinical outcomes of CRC, especially unresectable metastatic CRC (mCRC), still remain grim, which urgently requires a novel treatment strategy. Recently, immune checkpoint blockage (ICB) with monoantibody targeting programmed cell death‐1 (PD1) or programmed cell death ligand‐1 (PD‐L1) has been proved to be superior to typical chemotherapy in improving progression‐free survival (PFS) time of highly microsatellite instable or deficient mismatch repair (MSI‐H/dMMR) CRC patients, which is confirmed in a series of clinical trials, providing novel options for treatment of mCRC.^[^ ^2^ ^]^

While the remarkable efficacy of immunotherapy in CRC is encouraging, its clinical applicability remains limited. Currently, only ≈15% of CRC patients exhibit tumors with MSI‐H/dMMR status, significantly restricting the broader use of ICB therapies in CRC.^[^ ^3^, ^4^ ^]^ The tumor microenvironment (TME), comprising cancer cells, stromal cells, and, critically, immune cells, plays a central role in supporting malignant traits, including sustained proliferation, resistance to cell death, angiogenesis, invasion, metastasis, and immune evasion.^[^ ^5^ ^]^ Emerging evidence suggests that the effectiveness of immunotherapy is closely linked to the immunological state of the TME. The majority of CRC cases are microsatellite stable (MSS) and proficient in mismatch repair (pMMR), typically associated with low tumor mutation burden and an immunologically “cold” or “desert” TME characterized by scarce tumor‐infiltrating lymphocytes, thereby limiting response to immunotherapy.^[^ ^6^ ^]^ Overcoming the primary resistance of MSS/pMMR CRC to immunotherapy remains a major clinical challenge, which fundamentally relies on a deeper understanding of the TME and its role in tumor progression and immune suppression.

Glycosylation is an enzymatic process of glycoconjugate formation in which carbohydrate chains named glycans are added to target proteins and lipids. As the most prominent posttranslational modifications, glycosylation is regulating protein biosynthesis, folding, and stability by altering the structure of proteins and their interactions with other molecules.^[^ ^7^ ^]^ The spatial structure of abnormal tumor glycosylation changes the way immune system perceives tumors, and can also induce immunosuppressive signals through glycosylation products binding receptors. For example, the binding of Tn glycan to macrophage galactose‐specific lectin on MUC‐1, indicating dendritic cell‐driven TH2‐mediated responses, may contribute to the occurrence of immune evasion in tumors.^[^ ^8^ ^]^ Accumulating evidence indicates that cell membrane immune checkpoint proteins, such as programmed death‐ligand 1 (PD‐L1), are highly glycosylated with N‐linked glycan moieties in human cancers to maintain its protein stability and interaction with PD‐1, which in turn promotes evasion from T‐cell mediated adaptive immunity.^[^ ^9^ ^]^ Notably, studies have suggested targeting PD‐L1 glycosylation to enhance its detection and therapeutic efficacy as a rational option in ICB treatment.^[^ ^7^, ^9^, ^10^ ^]^ However, the biological functions, molecular mechanisms, and clinical significance of PD‐L1 glycosylation in CRC have not been fully elucidated.

In this study, we interpreted multi‐omic datasets, including transcriptomic, single‐cell sequence, and spatial transcriptomic data to gain comprehensive insights into glycosylation of CRC. As shown, unsupervised clustering of glycogenes could distinguish CRC patients into subgroups with distinct prognosis, different TME components, and discrepant response to ICB therapy, suggesting a crucial role of glycosylation in regulating carcinogenesis and progression of CRC. Subsequent analysis of single‐cell dataset demonstrated a detailed landscape of cell‐cell communication in TME of different subgroups, unveiling potential chemokines and intrinsic pathways by which glycogenes reshape TME. More importantly, we identified through in vivo and vitro experiments that knockdown of ST6GAL1 resulted in a dampened proliferative and metastatic ability of CRC cells, probably due to the decreased α2,6‐sialylation of PD‐L1 mediated by ST6GAL1, which downregulates the stability of PD‐L1 and leads to further ubiquitination and degradation. Moreover, ST6GAL1 knockdown help improve tumor response together with anti‐PD‐L1 therapy in vivo, implicating that ST6GAL1 has the potential to be a vital target in improving drug resistance in immunotherapy. In addition, single‐cell analysis reveals ST6GAL1‐high CRC cells are associated with immunosuppressive TME. Contrasting, ST6GAL1‐low cells show heightened CD8^+^ T cell association, indicating a potential for enhanced immunotherapy response and suggesting glycometabolic distinctions between subgroups.

## 2 Results

### 2.1 Genomic Landscape of Distinct Glycosylation Patterns in CRC

**Figure 1:**
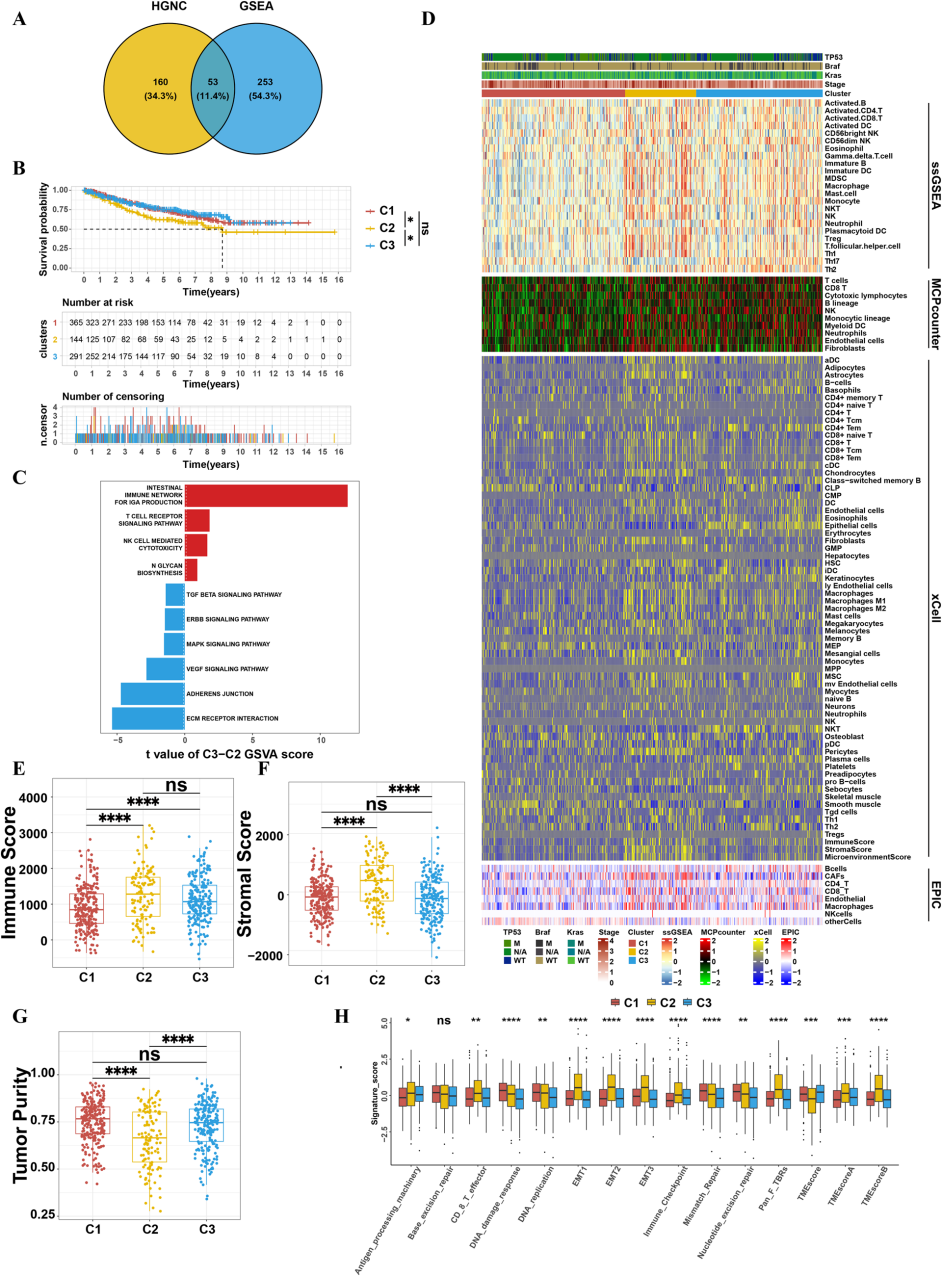
TME characteristics and relevant biological pathway in distinct glycogene clusters. A) Venn diagram of the intersection of 53 glycogenes between HGNC and GSEA gene sets. HGNC, HUGO Gene Nomenclature Committee; GSEA, Gene Set Enrichment Analysis. B) Kaplan–Meier curves of overall survival (OS) for different glycogene clusters in the meta‐GEO cohort (GSE39582, GSE38832, and GSE87211). C) In the GSE39582 cohort, differential KEGG pathways between cluster C3 and cluster C2 analyzed through GSVA scoring. D) Analysis of tumor‐infiltrating immune cells in three clusters within the GSE39582 cohort using ssGSEA, EPIC, MCPcounter, and XCell algorithms. E–G) ESTIMATE algorithm assessments of E) immune scores, F) stromal scores, and G) tumor purity for three glyco‐gene clusters in the GSE39582 cohort. H) Proportions of different labels (immune‐related, mismatch‐related, and stromal‐related) and TMEscore in the GSE39582 cohort. Statistical differences between the three glyco‐gene clusters were analyzed using the Kruskal‐Wallis test. *p < 0.05; **p < 0.01; ***p < 0.001; ****p < 0.0001; ns, not significant.

A flowchart was developed to systematically illustrate our study design (Figure S1, Supporting Information). To identify distinct glycosylation patterns and their downstream mechanisms in CRC, we acquired 53 (11.4%) overlapping glycogenes from GSEA and HGNC (**Figure** **1**A). Based on the expression profile of these 53 glycogenes in GSE39582, GSE38832, and GSE87211 datasets, we classified 800 CRC patients into 3 clusters termed as C1–3 by unsupervised hierarchical clustering (Figure S2A, Supporting Information). Survival analysis revealed worse overall survival (OS) among patients in C2 compared with the other clusters (C1 vs C2, P = 0.02; C2 vs C3, P = 0.02, Figure 1B). To further explore the characteristics of clinical features and biological behaviors between different glycosylation modification patterns, CRC patients in the GSE39582 cohort were selected for subsequent analysis because of intact clinical information. After unsupervised hierarchical clustering, CRC patients in the GSE39582 cohort were also clustered into 3 clusters (Figure S2B, Supporting Information) with 236 patients in C1, 115 patients in C2, and 205 patients in C3, respectively. Kaplan–Meier curves showed that patients in C2 group had worse OS, which was consistent with the above findings (C1 vs C2, P = 0.015; C2 vs C3, P = 0.015, Figure S2C, Supporting Information). In addition, we also found there were significant differences in biological behaviors between these three glycosylation modification patterns. The results of GSVA indicated that the intestinal immune network for IgA production, T cell receptor signaling, and natural killer cell mediated cytotoxicity pathways were enriched in Cluster C3, while in Cluster C1, pathways related to apoptosis and T‐cell receptor signaling are notably enriched. On the contrary, oncogenic and metabolic reprogramming processes (ECM receptor interaction, VEGF signaling, cell adhesion, MAPK signaling, and TGF‐β signaling pathways) were enriched in Cluster C2 (Figure 1D; Figure S2D,E, Supporting Information), which might account for the unfavorable clinical outcomes of C2 group patients.

To further investigate the potential mechanisms governing different biological behaviors of CRC patients from three clusters, we utilized several deconvolutional algorithms to simulate the cell‐type‐specific gene expression profiles to predict the abundance of each cell types of TME. We found that MDSCs and Tregs were predominantly enriched in Cluster C2 as depicted by the ssGSEA analysis. Similarly, macrophages and CAFs also exhibited higher enrichment in Cluster C2, primarily identified through ssGSEA and corroborated by additional methods such as MCPcounter, xCell, and EPIC. This multi‐method analysis further supports the hypothesis that Cluster C2 is associated with an immunosuppressive TME (Figure 1D). Moreover, the results of ESTIMATE algorithm showed that patients in C2 had higher stromal scores, immune scores and lower tumor purity (Figure 1E–G). Finally, we evaluated their TME scores, a novel concept from previous studies representing the characteristics of the tumor immune microenvironment as TMEscore A – TMEscore B.^[^ ^11^ ^]^ We found that TMEscore in cluster C2 is significantly lower than C1 and C2. Besides, scoring of several known signatures revealed that stromal activities in C2 was significantly enhanced, including angiogenesis, epithelial mesenchymal transition (EMT), and transforming growth factor β (TGFβ) pathway (pan_F_TBRs), further validating our above results. Notably, we notified that expression of immune checkpoint genes was enhanced in C2 (Figure 1H), indicating a global immunosuppressive TME of these patients. Similarly, CRC patients from TCGA CRC cohort could also be divided into 3 Clusters (Figure S2F, Supporting Information), among which C2 was enriched with immunosuppressive cells and stromal pathways (Figure S2G–I, Supporting Information). Furthermore, we also observed that the C2 group in the TCGA cohort has a higher stromal score (Figure S2J,K, Supporting Information) and is characterized by lower levels of CD8^+^ T cells and NK cell infiltration (Figure S2L,M, Supporting Information). Pathway analysis also shows that the TMEscore of the C2 cluster in the TCGA cohort is lower (Figure S2N, Supporting Information), lending further support to our speculations. Taken together, these results indicated that there were prominent distinctions between CRC patients with the different glycosylation pattern.

### 2.2 Glycogene Signature for Heterogeneous CRC Subtypes

**Figure 2:**
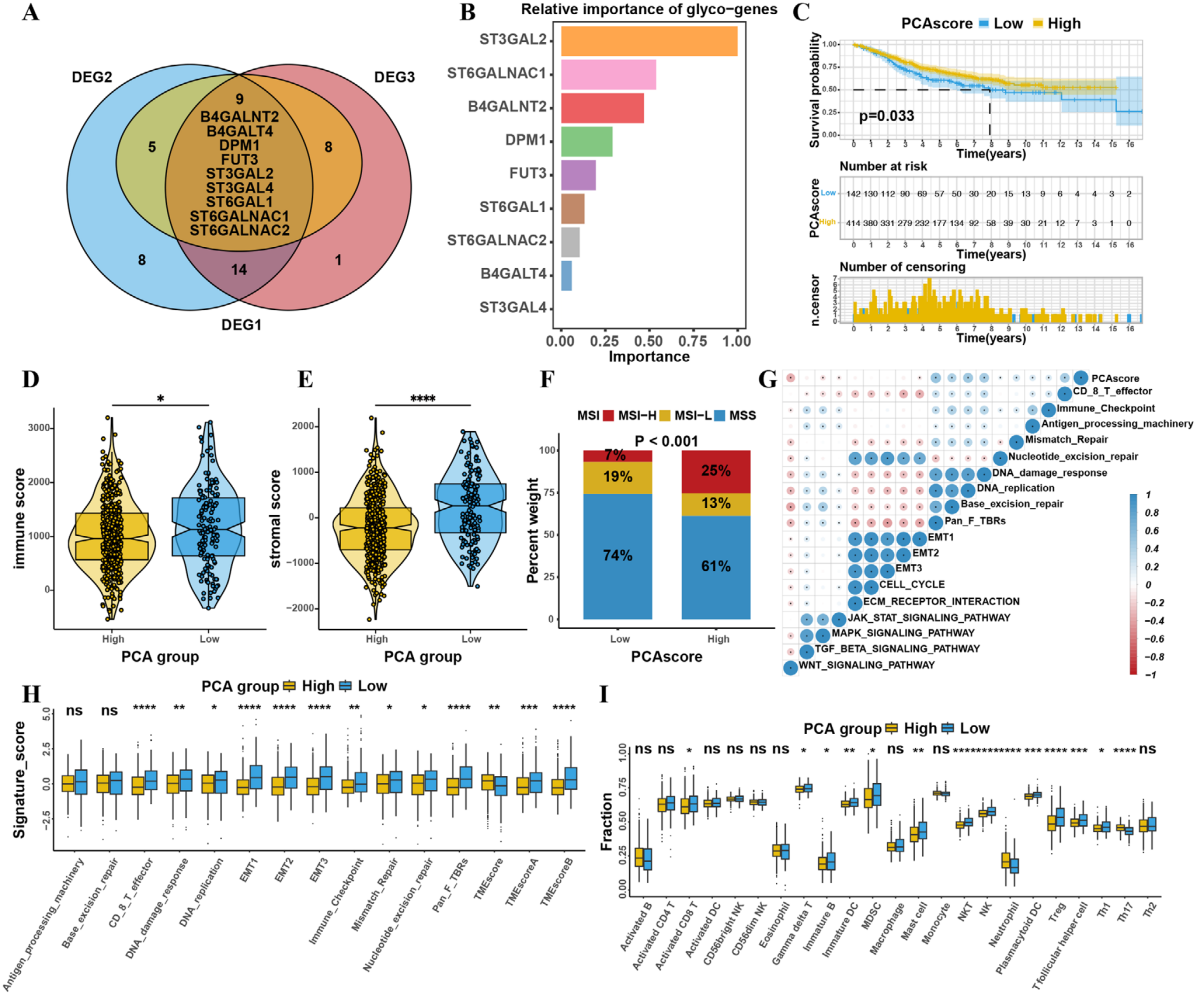
Construction and functional annotation of glycogene signature. A) Venn diagrams of differentially expressed genes (DEGs) among clusters. DEG1: C1 versus C2; DEG2: C1 versus C3; DEG3: C2 versus C3. B) Random Forest algorithm identification of genes relevant to prognosis within the three sets of DEGs (GSE39582 cohort), with rectangle length representing the importance of prognosis. C) Kaplan–Meier curves for patients with high and low PCA scores in the GSE39582 cohort, analyzed using the Log‐rank test. D,E) ESTIMATE algorithm assessments of D) immune scores, and E) stromal scores for patients with high and low PCA scores in the GSE39582 cohort. F) Proportions of MSI status in high and low PCA score groups. G) Spearman analysis of the correlation between PCA scores and biological gene labels (immune‐related, mismatch‐related, and stromal‐related) in the GSE39582 cohort. Blue indicates a negative correlation; orange indicates a positive correlation. H) Differences in immune‐related labels, mismatch‐related labels, and stromal‐related labels, as well as TMEscore, between high and low PCA score groups in the GSE39582 cohort. I) Proportions of tumor‐infiltrating immune cells in high and low PCA score groups. Statistical differences in PCA high and low groups were analyzed using the Kruskal‐Wallis test. *p < 0.05; **p < 0.01; ***p < 0.001; ****p < 0.0001; ns, not significant.

To comprehensively investigate the heterogeneity of CRC samples with distinct glycosylation modification patterns, we next screened key regulators among 53 glycogenes between three clusters. As shown, nine overlapping differential expressed genes, including B4GALNT2, B4GALT4, DPM1, FUT3, ST3GAL2, ST3GAL4, ST6GAL1, ST6GALNAC1, and ST6GALNAC2 (**Figure** **2**A) were then transferred to random forest (RF) method to identify genes mostly relevant to CRC patient prognosis in GEO and TCGA cohorts. Seven (B4GALNT2, DPM1, FUT3, ST3GAL2, ST6GAL1, ST6GALNAC1, and ST6GALNAC2) of them were intersected between the GSE39582 and TCGA datasets (Figure 2B; Figure S3B, Supporting Information) and were applied to develop principal component analysis (PCA) scoring system. As expected, the median value of PCA scores could divide CRC patients from GEO dataset into high and low group with distinct clinical outcome (Figure 2C), which confirmed the robustness of glycogene signature. To our surprise, subsequent TME cell type analyses showed that PCA‐low group was remarkably enriched with immune cell infiltration including natural killer cell, macrophage, MDSC, dendritic cell even activated CD8^+^T cells (Figure 2I), thus possessing a higher immune score (Figure 2D). However, patients within this group did not show a matching survival advantage (log‐rank test, p = 0.033; Figure 2C). We presumed that PCA‐low CRC cases may belong to immune excluded phenotype in which abundant immune cells were retained in stroma instead of penetrating into parenchyma tissue. Besides, higher proportion of Tregs (Figure 2I) and hyperactivated EMT pathways (Figure 2G,H) were also observed in PCA‐low CRC patients, which formed an immunosuppressive TME and might result in poorer survival. Meanwhile, we employed PCAscoring system to TCGA CRC cohort. Consistent with above results, PCA‐low group was also associated with infiltration of immuno‐suppressive cells and activation of stromal pathways (Figure S3E,G–I, Supporting Information). In addition, lower PCA score was significantly related to microsatellite instability low (MSI‐L) or microsatellite stable (MSS) status in TCGA cohort (Figure 2F), raising the question that whether the unfavorable prognosis of PCA‐low patients was partly due to the negligible response of MSI‐L or MSS CRC to anti‐PD1 immunotherapy.

### 2.3 Glycogene Signature Predicted Response to Immunotherapy

**Figure 3:**
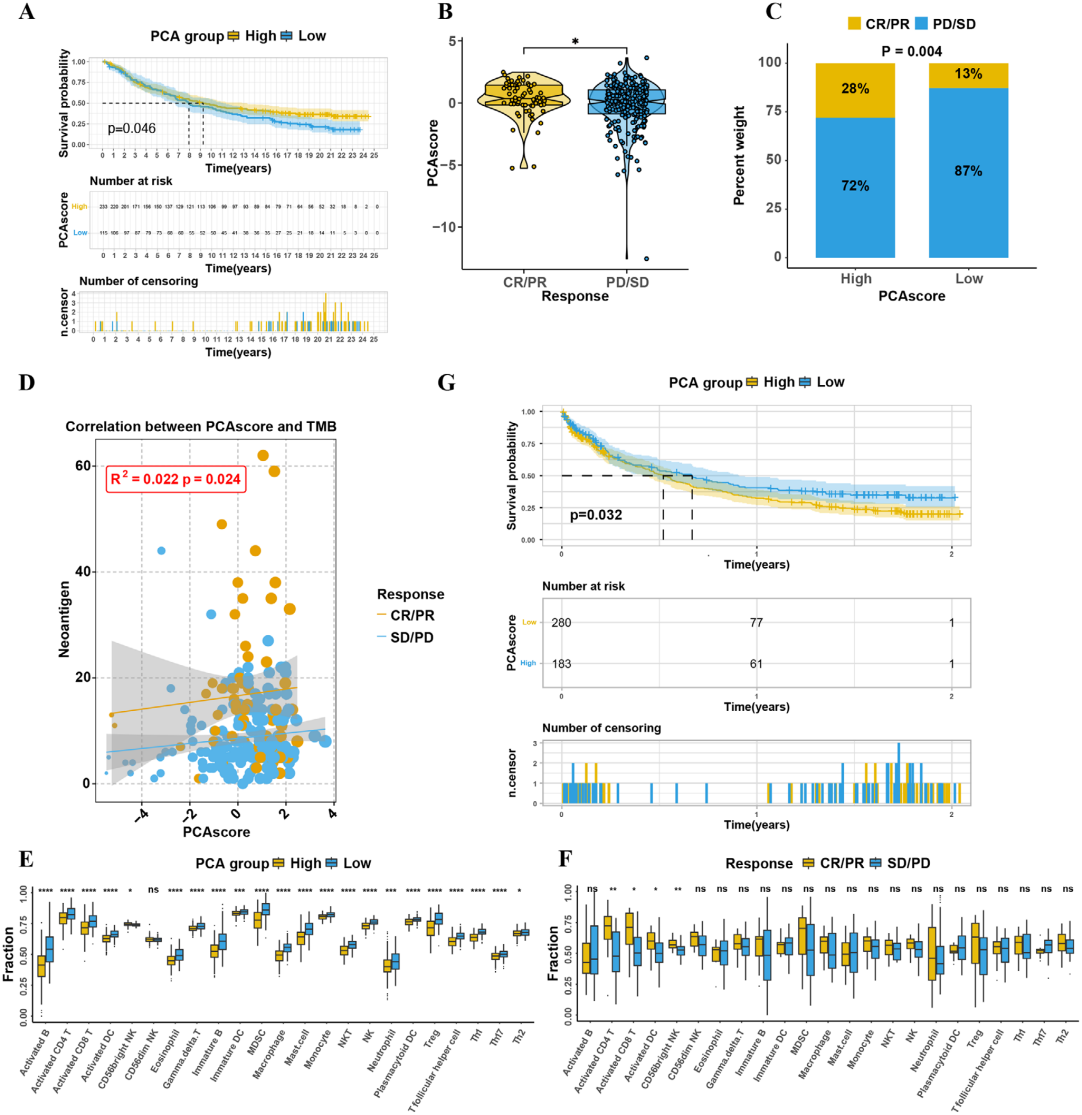
The Role of Glycogene Signature in Predicting the Efficacy of Immunotherapy. A) Kaplan–Meier curves for patients with high and low PCA scores in the IMvigor210 immunotherapy cohort, analyzed using the Log‐rank test. B,C) Distribution of PCA scores between responders and non‐responders to immunotherapy in the IMvigor210 cohort. SD, stable disease; PD, progressive disease; CR, complete remission; PR, partial remission. D) Correlation between PCA scores and tumor mutational burden in the CR/PR and SD/PD groups. E) Proportions of tumor‐infiltrating immune cells in high and low PCA score groups (IMvigor210). F) Proportions of tumor‐infiltrating immune cells in CR/PR and SD/PD groups (IMvigor210). G) Kaplan–Meier curves for patients with high and low PCA scores in a combined immunotherapy cohort, analyzed using the Log‐rank test. Statistical differences in PCA high and low groups were analyzed using the Kruskal‐Wallis test. *p < 0.05; **p < 0.01; ***p < 0.001; ****p < 0.0001; ns, not significant.

ICB with monoclonal antibodies targeting T‐cell inhibitory molecules such as PD‐1, PD‐L1 and CTLA‐4 has recently emerged as a novel treatment strategy with unprecedented survival benefits for cancer patients, especially for MSI‐H CRC patients. To answer the above question, we next explored the prognostic and predictive value of glycogene‐originated PCA score in several ICB cohorts. As expected, patients with urothelial cancer from IMvigor210 cohort could be dichotomized into three clusters with varied expression of glycogenes and two subgroups with distinct prognosis. Similarly, patients in high PCA score group showed remarkable survival benefit (log‐rank test, p = 0.046; **Figure** **3**A) as a result of more active response to anti‐PD‐L1 therapy (Figure 3B‐C), which showed a high uniformity with a classical marker, the tumor mutation burden (TMB, Figure 3D). Notably, PCA‐low group showed dramatic enrichment of immune cells especially immunosuppressive cells such as Tregs, type17 T helper cells, and MDSCs (Figure 3E,F), which may impair response to anti‐PD‐L1 and led to poor prognosis. Moreover, we combined available anti‐PD‐L1 treatment cohort (IMvigor210, GSE78220, GSE126044, and GSE176307) to generate an ICB meta cohort consisting of over 460 patients. Still, the glycogene PCA scoring system made precise prediction that PCA‐high group had significantly prolonged survival time (Figure 3G). In summary, our work apparently demonstrated that glycosylation pattern was significantly correlated with tumor immune phenotypes and response to immunotherapy in pancancer anti‐PD‐L1 datasets, highlighting the clinical importance of glycogene signature in planning treatment strategy for CRC patients.

### 2.4 ST6GAL1 Upregulation Correlates with Poor Prognosis in CRC Patients

**Figure 4:**
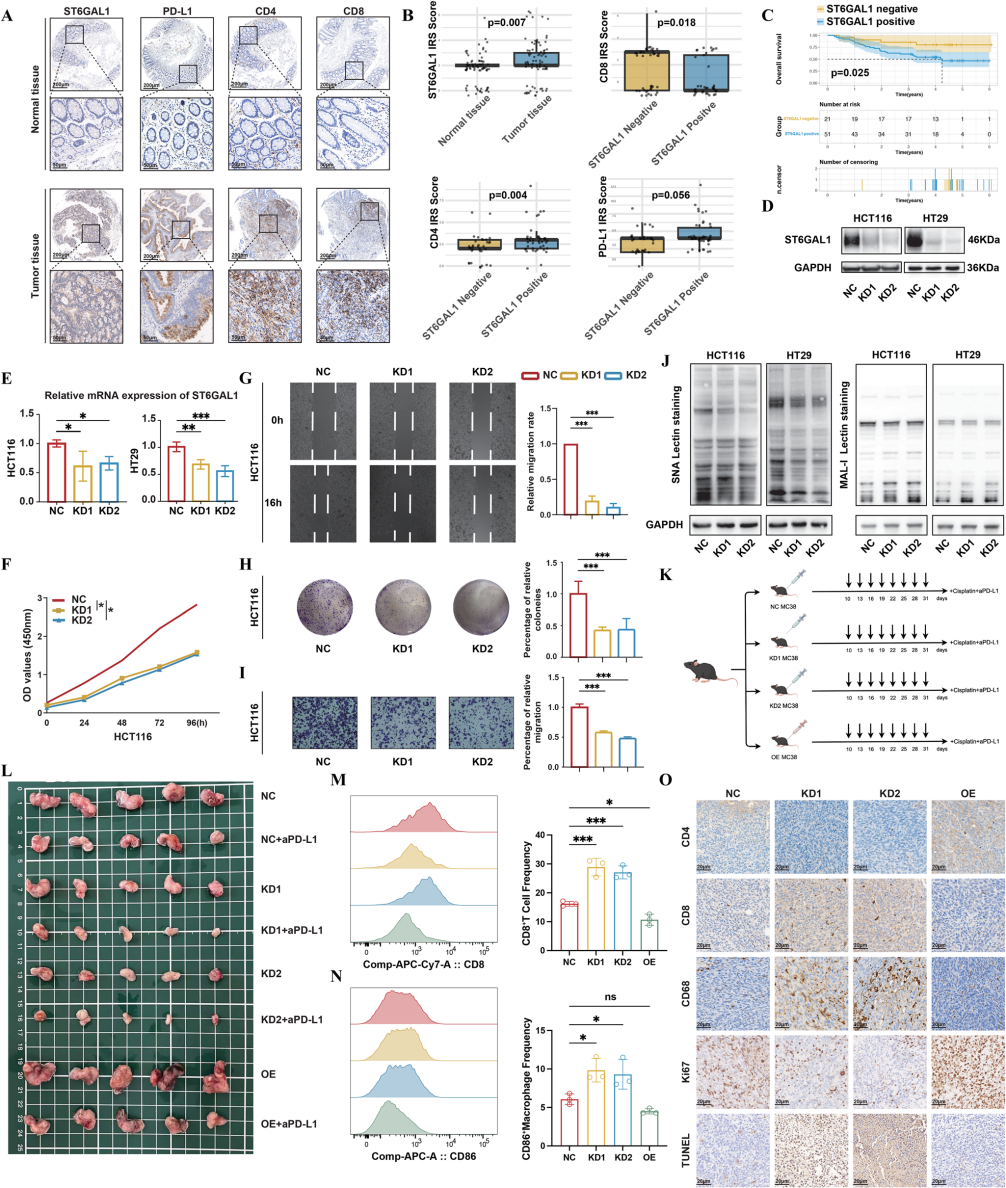
ST6GAL1 mediates the malignant phenotype of CRC. A,B) Immunohistochemical analysis of ST6GAL1, PD‐L1, CD4, and CD8 in representative CRC tissues and normal tissues. Comparison of IRS scores of CD4, CD8, and PD‐L1 in ST6GAL1 positive and negative groups of CRC tumor tissues. n = 76. Scale bar: 200 and 50 µm. C) Kaplan–Meier analysis of overall survival in our immunohistochemical cohort based on ST6GAL1 protein expression level detected in the tumor tissues. D,E) The transfection efficiency of lentivirus was verified by Western blotting and RT‐qPCR (n = 3). F–I) The proliferative ability of the transfected CRC cell line was assessed using the CCK8 assay and colony formation assay (n = 3), while the migration ability was evaluated through the healing and transwell assay (n = 3). J) Lectin blot analysis of glycoproteins in negative control cells and ST6GAL1‐KD cells using SNA (specific for α2,6‐linked sialic acid) and MAL‐I (specific for α2,3‐linked sialic acid), n = 3. K) Schematic representation of the xenograft mouse model and dosage regimen. L–O) Stable MC38 cells transfected with the indicated lentiviruses were injected into C57BL/6 mice. Treated groups were administrated with anti‐PD‐L1 mAb plus Cisplatin. L) Representative images of tumors, M,N) Representative flow cytometry histogram plots (left) and proportion (right) of tumor‐infiltrating CD8^+^ T cells and M1 macrophages in tumor tissues (n = 5). O) Representative IHC images of subcutaneous tumor tissue. Scale bar: 20 µm. Data were presented as mean ±SD. P values of Kaplan–Meier survival curves were calculated by log‐rank test. Statistical analysis in other panels was performed with one‐way ANOVA followed by Tukey's test, ns no statistical significance; *p < 0.05, **p < 0.01, ***p < 0.001.

Among the above gene signatures, ST6GAL1 catalyzes the α2,6‐sialylation process and plays crucial roles in tumorigenesis and tumor progression.^[^ ^12^, ^13^ ^]^ To further confirm the validity of the model and explore the molecular mechanism of glycosylation, ST6GAL1 was selected for further research. ST6GAL1 has been proven to be up‐regulated in a variety of cancers and plays a key role in tumor invasion and metastasis.^[^ ^14^, ^15^, ^16^ ^]^ ST6GAL1 expression was evaluated by IHC in tumor and adjacent normal tissues from 72 CRC patients at our center. The results showed that ST6GAL1 protein levels were significantly higher in tumor tissues compared to normal tissues (p = 0.007; **Figure** **4**A,B). Based on staining intensity and coverage, this cohort was categorized into ST6GAL1‐negative (n = 21) and ST6GAL1‐positive (n = 51) groups according to the German score. Kaplan–Meier survival analysis revealed that the ST6GAL1‐positive cohort had significantly poorer prognosis (p = 0.025; Figure 4C). To further investigate differences in the TME between the two groups, immunoreactivity score (IRS) for CD4, CD8, PD‐L1, CD66b, and CD68 were calculated and compared. Notably, the ST6GAL1‐negative group exhibited significantly higher expression of CD8 (p = 0.018) and CD4 (p = 0.004) (Figure 4A,B), whereas no significant differences were observed in the expression of CD66b (p = 0.093) and CD68 (p = 0.107) between the two groups (Figure S4A,B, Supporting Information). Although the difference in PD‐L1 expression between the ST6GAL1‐positive and ‐negative groups did not reach statistical significance (p = 0.056), a positive trend was observed, suggesting a potential correlation between ST6GAL1 and PD‐L1 (Figure 4A,B). To further investigate the role of ST6GAL1 in CRC progression, we established shRNA‐mediated knockdown in HT‐29 and HCT116 cell lines. The efficiency of transfection was validated by both RT‐qPCR and western blotting. Western blot results confirmed that ST6GAL1 protein expression was significantly reduced in cells transfected with ST6GAL1‐knockdown lentivirus compared to the negative control (NC) group (Figure 4D), consistent with the decreased ST6GAL1 mRNA levels observed in the knockdown groups by RT‐qPCR (Figure 4E). Functional assays including CCK8 (Figure 4F; Figure S4F, Supporting Information), wound healing (Figure 4G; Figure S4E, Supporting Information), colony formation (Figure 4H; Figure S4C, Supporting Information), and transwell migration assays (Figure 4I; Figure S4D, Supporting Information) demonstrated that ST6GAL1 knockdown significantly inhibited the proliferation and migration abilities of CRC cells. Furthermore, lectin blot analysis revealed a significant reduction in SNA binding signals following ST6GAL1 knockdown, while MAL‐I binding remained unchanged (Figure 4J), suggesting that ST6GAL1 specifically regulates tumor progression predominantly via α2,6‐sialylation without affecting α2,3‐sialylation.

To further study the role of ST6GAL1 in vivo, we established a subcutaneous tumor implantation model in C57BL/6 mice using MC38 cells transfected with NC, ST6GAL1‐KD, or ST6GAL1‐OE lentivirus. In vivo experiments further validated our previous findings. Macroscopic images of subcutaneous tumors showed that tumor size and volume were significantly reduced in the ST6GAL1‐KD groups compared to the NC group. Conversely, tumors in the ST6GAL1‐OE group were markedly larger (Figure 4L). Consistent with these observations, tumor growth curves demonstrated that ST6GAL1 knockdown suppressed, whereas overexpression promoted tumor growth in vivo (Figure S5A,C, Supporting Information). Survival analyses further indicated that mice bearing ST6GAL1‐KD tumors exhibited prolonged survival, while those in the ST6GAL1‐OE group showed impaired survival compared to controls (Figure S5G, Supporting Information). Additionally, liver metastasis was significantly inhibited in the ST6GAL1‐KD group (Figure S5H, Supporting Information).

We further examined the immune cell profile of subcutaneous tumors. Flow cytometry analysis revealed significantly increased infiltration and functional activity (IFN𝛾^+^) of CD8^+^ T cells in the ST6GAL1‐KD groups, whereas the ST6GAL1‐OE group exhibited reduced CD8^+^ T cell infiltration (Figure 4M; Figure S5F, Supporting Information). Similarly, M1 macrophage infiltration was upregulated upon ST6GAL1 knockdown, while an opposite trend was observed in the ST6GAL1‐OE group (Figure 4N). No significant differences in CD4^+^ T cell infiltration were observed among the groups (Figure S5E, Supporting Information). IHC analysis of subcutaneous tumor sections also confirmed that ST6GAL1 knockdown promotes the infiltration of CD8^+^ T cells, in line with flow cytometry findings. Moreover, IHC results demonstrated that ST6GAL1 knockdown suppressed the expression of the proliferation marker Ki‐67 and enhanced apoptosis, as detected by the TUNEL assay (Figure 4O).

### 2.5 ST6GAL1 Induced Sialylation of PD‐L1 to Maintain its Stability in CRC Cells

**Figure 5:**
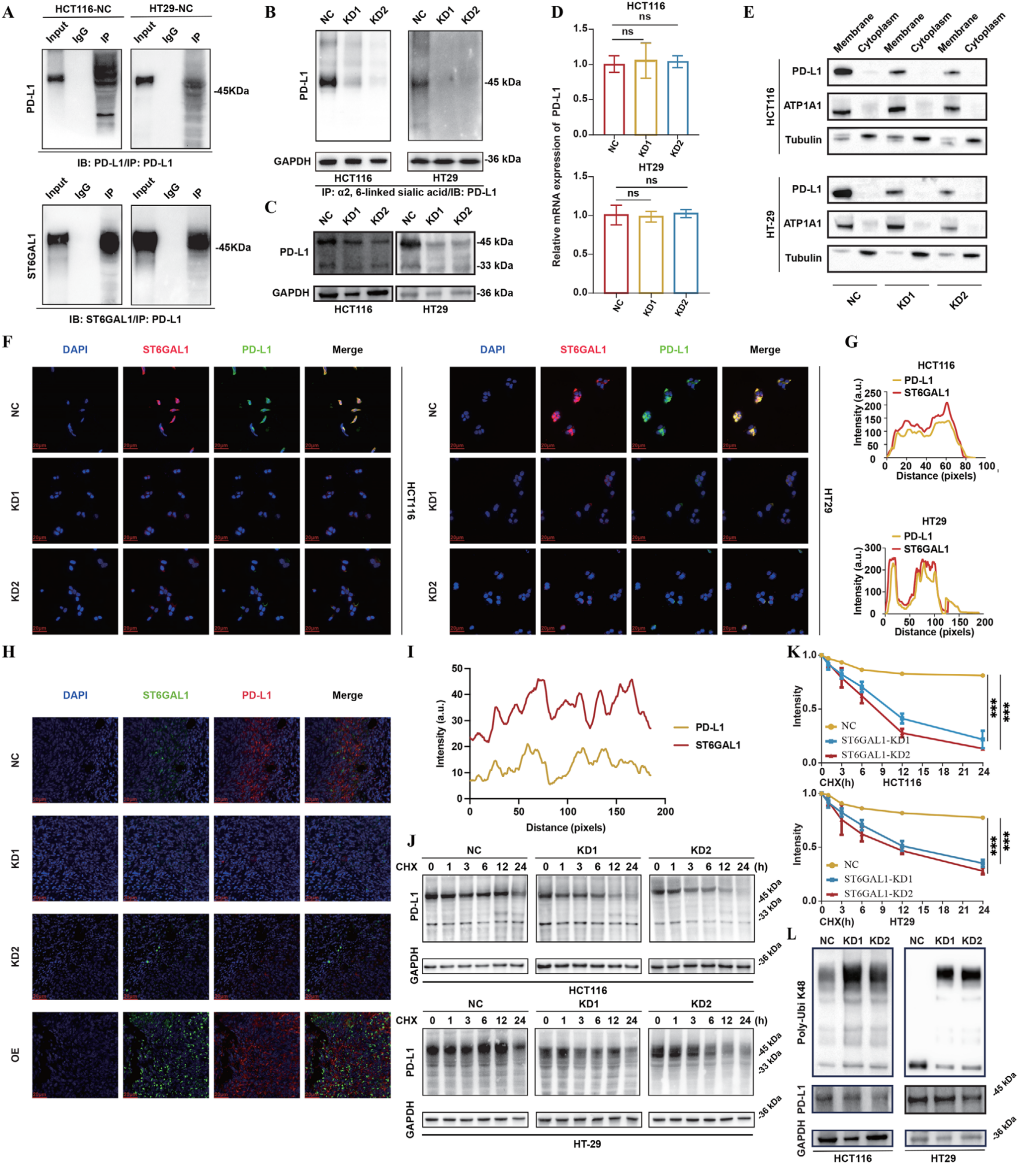
ST6GAL1 was involved in sialylation and maintains the stability of PD‐L1 in CRC. A) The endogenous complex of PD‐L1 and ST6GAL1 was detected by immunoprecipitation using anti‐PD‐L1 antibody and analyzed by immunoblot by anti‐PD‐L1 or anti‐ST6GAL1 antibody in HCT116 WT and HT‐29 WT cells (n = 3). B) Detection of sialylation pattern of PD‐L1 in stable cell model by SNA‐Lectin agarose immunoprecipitation (n = 3). C,D) Detection of PD‐L1 expression in ST6GAL1‐NC and ‐KD CRC cell lines by Western blotting and qRT‐PCR (n = 3). E) Subcellular fractionation of PD‐L1 after ST6GAL1 knockdown. Cell lysates were fractionated into cytoplasmic and membrane components via ultracentrifugation, and PD‐L1 expression levels were analyzed in each fraction (n = 3). Tubulin was used as a cytoplasmic marker, and Na^+^/K^+^‐ATPase was used as a membrane marker. F) Representative immunofluorescence images showing the expression and colocalization of ST6GAL1 (red) and PD‐L1 (green) in ST6GAL1‐NC and ‐KD HCT116 and HT29 cells. n = 3 independent samples. Scale bar: 20 µm. G) Line scan intensity profiles of ST6GAL1 and PD‐L1 in immunofluorescence‐stained ST6GAL1‐NC and ‐KD HCT116 and HT29 cells. Overlapping peaks indicate spatial colocalization between the two markers. H) Representative immunofluorescence images (left) showing the expression and colocalization of ST6GAL1 (green) and PD‐L1 (red) in subcutaneous tumor sections. n = 3 independent samples. Scale bar: 20 µm. Line scan intensity profiles (right) of ST6GAL1 and PD‐L1. Overlapping peaks indicate spatial colocalization between the two markers. J,K) CRC transfected cell lines were treated with 100 µg/mL cycloheximide (CHX) at 0, 1, 3, 6, 12, and 24 h to detect the changes of PD‐L1 stability (n = 3). The intensity of PD‐L1 protein was quantified using ImageJ software. L) Ubiquitination of PD‐L1 proteins in ST6GAL1‐NC and ‐KD cells. PD‐L1 proteins were immunoprecipitated and subsequently immunoblotted with a ubiquitin antibody (n = 3). Data are presented as the mean ± SD; Statistical analysis was performed with one‐way ANOVA followed by Tukey's test; ns no statistical significance; *p < 0.05, **p < 0.01, ***p < 0.001.

PD‐L1 undergoes extensive glycosylation, which plays a critical role in immune evasion and tumor progression.^[^ ^9^, ^17^ ^]^ Previous studies have demonstrated that N‐glycosylation is the major post‐translational modification (PTM) of PD‐L1, and sialylation is responsible for the terminal modification after N‐glycosylation. Given the known role of ST6GAL1 in catalyzing α2,6‐sialylation of N‐glycans^[^ ^12^, ^18^ ^]^ and its involvement in immune escape,^[^ ^19^ ^]^ combined with our observation of a positive correlation trend between ST6GAL1 and PD‐L1 expression, we hypothesized that ST6GAL1 may participate in the PTM regulation of PD‐L1. To test this hypothesis, we first performed immunoprecipitation assays. Endogenous PD‐L1 was immunoprecipitated from cell lysates using a specific antibody, and ST6GAL1 was detected in the precipitated complex, indicating a potential interaction between PD‐L1 and ST6GAL1 within the cellular context (**Figure** **5**A). Subsequent western blot analysis showed that PD‐L1 protein levels were markedly reduced in ST6GAL1 knockdown cell lines (Figure 5B), whereas no significant change was detected at the mRNA level, suggesting that ST6GAL1knockdown does not regulate PD‐L1 transcriptionally (Figure 5D). To further investigate whether ST6GAL1 regulates PD‐L1 via sialylation, SNA lectin pulldown following PD‐L1 immunoprecipitation was conducted. The results revealed a marked decrease in PD‐L1‐associated α2,6‐sialylation upon ST6GAL1 knockdown (Figure 5B), suggesting that ST6GAL1 regulates PD‐L1 expression through terminal α2,6‐sialylation in CRC cell lines. To further study the subcellular distribution of PD‐L1 and its alterations following ST6GAL1 knockdown, we performed subcellular fractionation. Western blot analysis of membrane and cytoplasmic fractions revealed a marked reduction in membrane‐localized PD‐L1 in the ST6GAL1‐KD group compared to the control (Figure 5E). To better visualize the interaction between PD‐L1 and ST6GAL1 in cells, we performed immunofluorescence staining on coverslips of HCT116 and HT‐29 cell lines. Notably, ST6GAL1 knockdown led to a reduction in PD‐L1 fluorescence intensity, suggesting that ST6GAL1 may regulate PD‐L1 expression. Line scan analysis of immunofluorescence images revealed a strong colocalization of PD‐L1 and ST6GAL1 in the control groups, as indicated by overlapping fluorescence intensity peaks along the scanned distance (Figure 5F,G). Similar results were observed in the immunofluorescence staining outcomes of the subcutaneous tumor tissues from mice, where ST6GAL1 and PD‐L1 exhibited strong colocalization. Moreover, PD‐L1 expression decreased following ST6GAL1 knockdown and increased upon ST6GAL1 overexpression, indicating that ST6GAL1 positively regulated PD‐L1 levels (Figure 5H,I). We next examined whether the downregulation of PD‐L1 expression after ST6GAL1 knockdown was attributable to changes in its protein stability. As anticipated, treatment with the protein synthesis inhibitor cycloheximide (CHX) revealed a faster degradation rate of PD‐L1 in ST6GAL1‐KD cell lines compared to the NC group, indicating that ST6GAL1 knockdown significantly reduces PD‐L1 protein stability (Figure 5J,K). One step further, we want to explore the involvement of proteasomal degradation pathway, so we treated cells with the proteasome inhibitor MG132. The ST6GAL1‐KD group exhibited markedly increased PD‐L1 ubiquitination compared to the NC group (Figure 5L), suggesting that the enhanced degradation of PD‐L1 after ST6GAL1 knockdown is primarily mediated by the proteasome pathway. In summary, these results suggest that ST6GAL1‐mediated sialylation plays a crucial role in maintaining PD‐L1 protein stability.

Given the correlation between ST6GAL1 expression and PD‐L1 protein stability, we hypothesized that silencing ST6GAL1 could have synergistic effects with anti‐PD‐L1 therapy. To confirm this hypothesis, we established a subcutaneous tumor implantation model using C57BL/6 mice with ST6GAL1‐NC, ST6GAL1‐KD, and ST6GAL1‐OE MC38 cell lines, followed by treatment with either PBS or a combination of anti‐PD‐L1 and cisplatin (Figure 4K). Representative images of subcutaneous tumors at the experimental endpoint demonstrated that the ST6GAL1‐KD groups receiving combination therapy developed significantly smaller tumors compared with the control group (Figure 4L). Similarly, tumor growth curves revealed that ST6GAL1 knockdown significantly suppressed tumor progression under anti‐PD‐L1 treatment, whereas ST6GAL1 overexpression slightly dampened this synergistic effect, although the differences were not statistically significant (Figure S5B,D, Supporting Information). These in vivo results indicate that ST6GAL1 knockdown enhances the antitumor efficacy of anti‐PD‐L1 therapy, highlighting a potential synergistic effect. Combined with our mechanistic findings that ST6GAL1 stabilizes PD‐L1 via α2,6‐sialylation, these results further underscore the functional importance of ST6GAL1 in modulating immune checkpoint dynamics in CRC.

### 2.6 Single‐Cell Analysis of Glycogene Signature in TME

**Figure 6:**
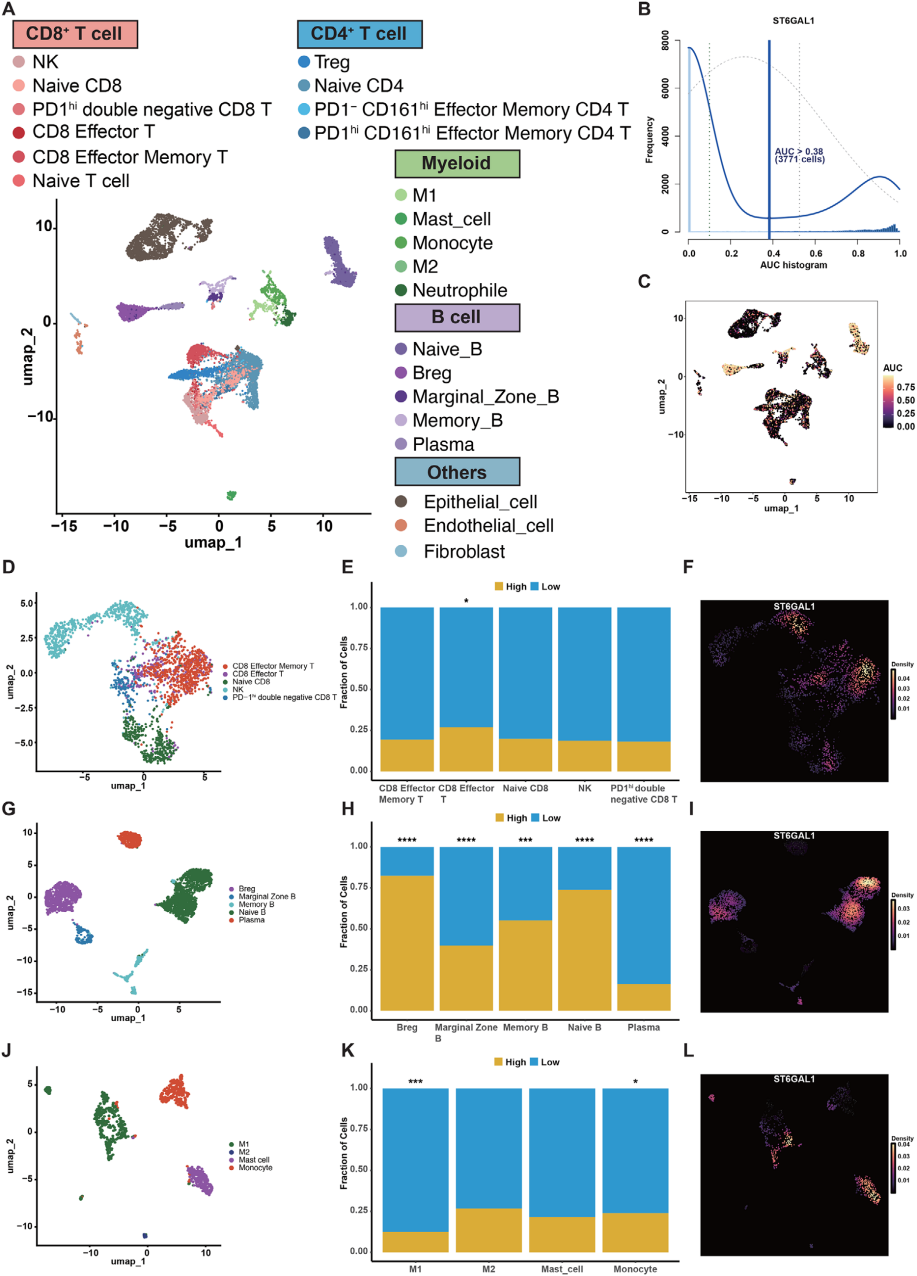
Regulation of TME Immune Cell Infiltration by ST6GAL1 in Single‐Cell Sequencing. A) UMAP plot showing single cells colored by cell type annotations. Each point represents a cell; colors indicate cell annotations. B) Cells are divided into high and low ST6GAL1 expression groups using a threshold of 0.38 based on AUCell scores. C) UMAP plot displaying AUCell scores for individual cells. D) UMAP plot of CD8^+^ T cells following subpopulation annotation. E) Proportions of different CD8^+^ T cell subsets in high and low ST6GAL1 expression groups. F) UMAP plot showing ST6GAL1 expression in CD8^+^ T cells. G) UMAP plot of B cells following subpopulation annotation. H) Proportions of different B cell subsets in high and low ST6GAL1 expression groups. I) UMAP plot showing ST6GAL1 expression in B cells. J) UMAP plot of monocytes/macrophages following subpopulation annotation. K) Proportions of different monocyte/macrophage subsets in high and low ST6GAL1 expression groups. L) UMAP plot showing ST6GAL1 expression in monocytes/macrophages. Data are presented as the mean ± SD; Statistical analysis was performed with Chi‐square test; ns no statistical significance; *p < 0.05, **p < 0.01, ***p < 0.001.

Bulk‐sample expression profiling has a limitation in potentially attributing the dysregulation of cell‐type‐specific genes to cancer, which could arise from overall variations in cellular composition. To mitigate this and substantiate our findings, we conducted single‐cell analysis for a granular view of glycogene‐mediated TME alterations. Using four CRC sample pairs from our cohort, we curated a dataset of 12213 cells through rigorous normalization and data cleansing. Post normalization using principal component analysis, we applied graph‐based clustering to segregate the cells into 18 clusters. These clusters were assigned to 23 cell types through typical marker genes or differentially expressed genes (**Figure** **6**A).

Utilizing the AUCell algorithm, each cell was scored based on the expression level of ST6GAL1. Cells were then divided into high and low expression groups using a cutoff value of 0.38 (Figure 6B,C). Subgroup analysis of CD8^+^ T cells, CD4^+^ T cells, myeloid, and B cells was subsequently conducted. We observed a significant increase in the infiltration of CD8^+^ effector T cells in the ST6GAL1 low expression group (Figure 6D–F), while the infiltration of CD4^+^ T cell subtypes remained unaffected (Figure S6A–C, Supporting Information). In B cells, we also noted a significant infiltration of plasma cells, which were potentially related to tertiary lymphoid structures (TLS), in the low expression group and a notable decrease in the infiltration of immunosuppressive Breg cells (Figure 6G–I). Consistent with the previous findings, the proportion of M1 macrophages in the myeloid population was significantly increased (Figure 6J–L). These results suggest that ST6GAL1 plays a pivotal role in the CRC TME, where low expression of ST6GAL1 may be associated with CD8^+^ effector T cell infiltration, tertiary lymphoid structure, and M1 macrophage polarization, thus fostering an “immune‐activated” TME that potentially improves prognosis and may enhance the sensitivity to PD‐L1 immunotherapy. Further, we continued investigating the developmental differentiation of the significantly altered cell subpopulations, performing pseudo‐temporal analysis for each subgroup. In line with our results, we observed differentiation from naive T cells to CD8^+^ effector T cells in the CD8^+^ T cell subgroup, from naive B cells toward Breg and plasma cells in B cell subgroups, and from monocytes toward M1 macrophages in the myeloid group (Figure S6D–I, Supporting Information). Finally, we extracted epithelial cells and distinguished aneuploid cells (malignant cells) from diploid cells (normal cells) using the CopyKat algorithm. Consistent with our bulk RNA‐seq results, ST6GAL1 was found to be highly expressed in malignant cells (Figure S6J,K, Supporting Information).

Subsequently, we sought to explore the mechanisms mediated by ST6GAL1 in altering the TME. We performed GSVA analysis on cells with high and low expression of ST6GAL1 (Figure S7A, Supporting Information). The results revealed significant upregulation of mismatch repair and VEGF pathways in the ST6GAL1 high expression group, which may explain why CRC patients with high ST6GAL1 expression have poorer prognosis. Conversely, in the ST6GAL1 low expression group, we observed activation of numerous metabolic pathways including oxidative phosphorylation, as well as activation of apoptosis and the MAPK pathway, potentially explaining the better prognosis and immune activation observed in these patients. Additionally, we noted significant enrichment of the ubiquitin‐mediated proteolysis pathway in the ST6GAL1 low expression group, consistent with our previous in vitro experiments. The interaction between tumor cells and immune components is crucial in reshaping the TME. To further identify potential mediators in this dynamic process, we employed the NicheNet package to quantify the interactions between epithelial cells and immune cells. As shown in the figures, compared to cells with high ST6GAL1 expression, a greater number of differentially expressed ligand‐receptor pairs were found in low expression cells to mediate intercellular communication (Figure S7B,C, Supporting Information). Notably, in the high‐expression group, cancer cells enhanced interactions with M1 macrophages, CD8^+^ effector T cells, and Bregs through HEBP1−EGFR, YARS−EGFR, and ICAM1−EGFR pathways, respectively. Here, we discovered that high expression of ST6GAL1 in CRC cells might drive the activation of EGFR‐related pathways, offering new insights for selecting CRC patients suitable for cetuximab therapy (anti‐EGFR). In contrast, cancer cells in the low expression group showed stronger associations with M1 and CD8^+^ effector T cells through interactions mediated by CXCL8−CXCR2, TNFSF10−TNFRSF10B, and IFNG−IFNGR2, potentially leading to higher immunotherapy sensitivity. Furthermore, we utilized the scMetabolism algorithm to decode the metabolic dysregulation between high and low expression groups (Figure S7D,E, Supporting Information). As anticipated, we observed significant differences in the glycolysis pathways between the high and low expression groups, confirming our results that ST6GAL1 serves as an effective marker to stratify CRC patients into distinct metabolic subgroups, particularly regarding glycolysis (Figure S7F, Supporting Information). Further studies are required to explore other aberrantly enriched metabolites, including fatty acids, amino acids, and purines.

**Figure 7:**
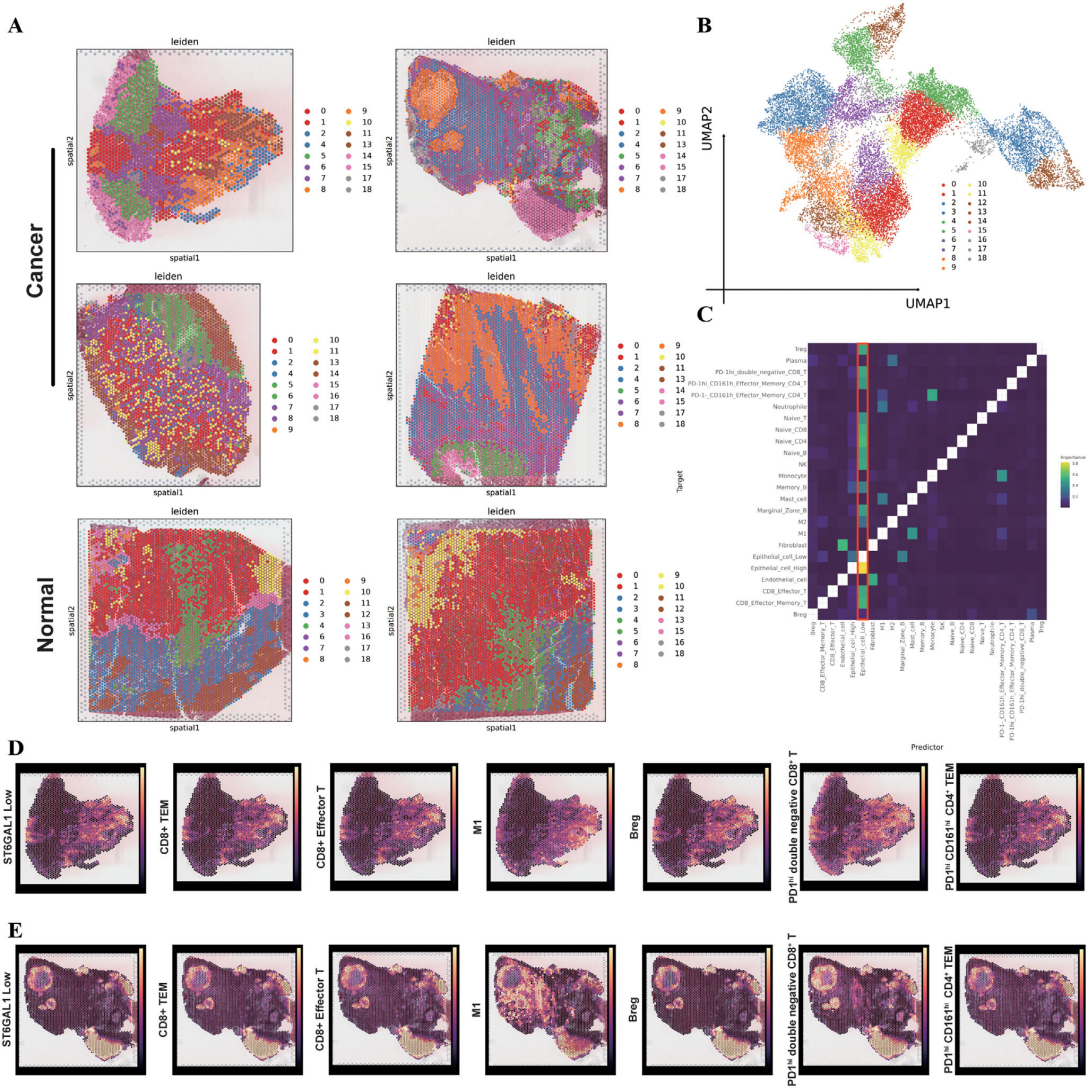
Spatial niche reprogramming around ST6GAL1‐high and ‐low cancer cells. A) Spatial transcriptomics spots from GSE225857, including four cancer samples and two normal samples, were categorized into 19 regional subgroups. B) The regional subgroups based on UMAP downscaling and niches. C) Niche colocalization patterns at adjacent regions, with color intensity increasing from black to yellow to indicate the relative importance of each pattern. D,E) Representative density plots showing spatial distribution of main cell types colocalized with ST6GAL1‐low cancer cells. D) tumor sample 1. E) tumor sample 2.

Single cell sequencing provides a high‐resolution view of TME. However, the change of relative spatial distribution patterns of different cell types, which cannot be revealed by single cell profiling due to tissue lysis, is of particular importance for TME reprogramming. To verify our findings from single cell sequencing, we collected six CRC and normal samples underwent spatial transcriptomic sequencing with 10×Visium platform. After quality control and normalization, all spots from CRC and normal samples were clustered as 19 niches (**Figure** **7**A,B). Diverse niche proximity was shown across cancer and normal tissues (Figure 7A), indicating a very high degree of TME heterogeneity in CRC. To quantify and demonstrate the spatial distribution relationship of different niches, we first evaluated cell type abundance in each spot using our inhouse CRC single cell data as a reference (Figure S8A, Supporting Information) and then analyzed the colocalization patterns of different cell populations in the view of intra‐ (in one spot, Figure S8B, Supporting Information) adjacent (adjacent six surrounding spots, Figure 7C) and para‐spot (beyond six surrounding spots, Figure S8C, Supporting Information), respectively. As shown in cancer tissue, both ST6GAL1‐high and ‐low epithelial cells were mainly located in niche 0–7 and niche 13–14 (Figure 7A,B,D,E; Figure S8D,E, Supporting Information), forming the cancer nest. However, ST6GAL1‐low cancer cells showed more significant attraction to CD8+ effector T cells, memory T cells, and M1 macrophages (Figure 7C–E), compared to ST6GAL1‐high cells (Figure 7C; Figure S8B,C, Supporting Information). Moreover, we notified that two kinds of PD‐i1^+^ T cells were found within the adjacent spots near ST6GAL1‐low cancer cells (Figure 7C–E). This suggested immunotherapy targeting PD1 to breakdown the cancer cell‐T cell interaction might rebuild the recognition ability of T cells to tumor cells, which enhanced immune response as above mentioned (Figure 3). Interestingly, similar spatial distribution patterns were observed in normal tissue (Figure S8F,G, Supporting Information), indicating ST6GAL1 as an initiating factor of CRC by regulating tissue microenvironment reprogramming, which needed further investigation.

## 3 Discussion

Glycosylation has long been proven to be involved in tumor immune editing and immunoregulation. Abnormal tumor glycosylation antigen masking not only changes the way immune system perceives tumors but also induces immunosuppressive signals through sugar‐binding receptors in lung, pancreatic cancer, and CRC.^[^ ^20^, ^21^, ^22^ ^]^ However, the role of glycosylation in CRC remains relatively unclear. Therefore, investigating the role of glycosylation in human cancer will help us deepen our understanding of the regulation mechanisms of TME and provide more effective immunotherapy strategies.

In this study, we identified three distinct glycosylation‐related molecular subtypes of CRC, each characterized by unique biological behaviors and TME immune infiltration profiles. Among the glycosylation‐related genes, ST6GAL1 emerged as a key sialyltransferase responsible for catalyzing the transfer of α2,6‐linked sialic acid from CMP‐sialic acid to substrates containing terminal galactose residues. Previous studies have shown that ST6GAL1 is upregulated in CRC and various other malignancies, and that its elevated expression is associated with tumor progression, metastasis, and poor prognosis.^[^ ^12^, ^13^, ^14^, ^15^, ^16^ ^]^ Consistent with these findings, our data demonstrated that knockdown of ST6GAL1 suppressed CRC cell proliferation, migration, and tumorigenicity both in vitro and in vivo. Moreover, ST6GAL1 was found to be upregulated in colorectal tumor samples from our clinical cohort, and its high expression was significantly correlated with poor prognosis in CRC patients, further supporting its oncogenic role. Importantly, our glycosylation‐based classification model demonstrated predictive value for immune response. One possible explanation of above results is that ST6GAL1 may induce an immunosuppressive phenotype, where abundant immune cells are retained in the stroma instead of penetrating into the tumor nests.^[^ ^23^, ^24^ ^]^ Using CRC tissue microarrays, we confirmed that high ST6GAL1 expression is associated with reduced CD8⁺ T cell infiltration and increased PD‐L1 expression. These findings were further validated in murine tumor models, where high ST6GAL1 expression correlated with decreased infiltration of both CD8⁺ T cells and M1‐like macrophages. Given that T cell infiltration is a key determinant of favorable prognosis and immunotherapy efficacy, these results suggest that aberrant ST6GAL1 expression and its mediated sialylation may drive immune evasion and resistance to immunotherapy by shaping a suppressive TME.^[^ ^19^, ^25^ ^]^

Previous studies have demonstrated that PD‐L1 is highly glycosylated during tumor development and progression. Liu et al. reported that N‐glycosylation of PD‐L1 in B‐cell lymphoma suppressed the cytotoxic function of CD8⁺ T cells against tumor cells.^[^ ^26^ ^]^ Similarly, Lee et al. found that deglycosylation of PD‐L1 in breast cancer samples significantly enhanced the binding affinity and signaling strength of anti–PD‐L1 antibodies, thereby facilitating the reactivation of T cell immunity by blocking PD‐1/PD‐L1 interactions.^[^ ^17^ ^]^ These findings indicate that the glycosylation status of PD‐L1 is closely associated with its immunomodulatory function. Thus, targeting PD‐L1 glycosylation on tumor cells may offer significant therapeutic potential in cancer immunotherapy. In our study, we observed a higher IRS of PD‐L1 in ST6GAL1‐positive tumor samples, supporting the notion that ST6GAL1 and PD‐L1 are co‐localized and may interact functionally. Li et al. further confirmed that PD‐L1 is a highly N‐glycosylated protein with four N‐glycosylation sites, and that glycosylation protects PD‐L1 from ubiquitination and proteasome‐dependent degradation.^[^ ^10^ ^]^ Our results also suggest that ST6GAL1 stabilizes PD‐L1 protein via α2,6‐sialylation, thereby preventing its proteasomal degradation and promoting an immunosuppressive tumor microenvironment. Notably, the combination of ST6GAL1 knockdown and anti–PD‐L1 therapy elicited the most pronounced anti‐tumor response, indicating a potential synergistic effect. These findings suggest that ST6GAL1 may serve as a promising therapeutic target to enhance the efficacy of ICB in colorectal cancer.

Our single‐cell analysis has elucidated the role of ST6GAL1 in the formation of the CRC TME, highlighting interactions of high ST6GAL1‐expressing tumor cells with B cells, tumor‐associated macrophages, and potential EGFR‐related pathways. Cells with low ST6GAL1 expression demonstrated enhanced associations with CD8^+^ effector T cells and M1‐type macrophages, and distinct glycolytic metabolic features, indicating different therapeutic implications. EGFR is widely expressed on the surface of mammalian epithelial cells and fibroblasts, involved in multiple pathways including the PI3K)/Akt/mTOR and RAS/RAF/MAPK pathways.^[^ ^27^ ^]^ The activation or dysregulation of these pathways, or imbalance in sensitive feedback loops, leads to the transcription of genes that promote cell survival, anti‐apoptosis, proliferation, angiogenesis, and metastatic potential. Cetuximab, an EGFR monoclonal antibody, binds to its ligand to block the activation of the RAS/RAF/MAPK pathway, inhibiting tumor growth.^[^ ^28^, ^29^ ^]^ The NCCN, CSCO, and EMSO guidelines recommend cetuximab as a first‐line treatment for RAS wild‐type metastatic CRC. Our findings indicate that cancer cells in the high ST6GAL1 expression group interact with M1 macrophages, Breg, and CD8^+^ T cells via HEBP1−EGFR, YARS−EGFR, and ICAM1−EGFR, suggesting that CRC patients with high ST6GAL1 expression may benefit from anti‐EGFR therapy. Our scRNA‐seq as well as flow cytometry results also show reduced expression of the T cell effector molecules IFN‐γ in the high ST6GAL1‐expressing CD8^+^ effector T cells, indicative of potential T cell exhaustion. Targeting this T cell subtype through immunotherapy could enhance treatment efficacy, aligning with our findings in the aforementioned immunotherapy cohort. These insights advance our understanding of ST6GAL1's role in modifying the TME and underscore its potential as a therapeutic target and prognostic marker.

In summary, our study highlights the upregulation of ST6GAL1 in colorectal cancer (CRC) and its strong association with tumor progression, suggesting its potential as a therapeutic target. We further demonstrate that ST6GAL1 stabilizes PD‐L1 through α2,6‐sialylation, which may contribute to immune evasion and therapeutic resistance. Therefore, ST6GAL1 may not only serve as a prognostic biomarker for CRC but also influence patient responsiveness to immunotherapy. These findings offer new insights into the mechanisms underlying immune escape in CRC and provide a rationale for the development of ST6GAL1‐targeted therapeutic strategies.

This study has several limitations. First, although ST6GAL1 may regulate the protein stability of PD‐L1 through α‐2,6 sialylation, the specific binding sites between ST6GAL1 and PD‐L1 remain undefined and require further structural or mutagenesis‐based investigation. Second, although we analyzed multiple pan‐cancer immunotherapy cohorts, the lack of an independent, colorectal cancer–specific immunotherapy dataset limits the direct clinical relevance of our findings to this tumor type. Future studies with CRC‐specific cohorts are warranted to validate our observations and strengthen translational applicability.

## 4 Conclusion

In conclusion, the present findings confirmed an extensive regulatory mechanism of glycogenes in the TME and are related to immunotherapeutic response. ST6GAL1 promoted the malignant phenotype of CRC cells, and its upregulated expression was associated with a poor prognosis for patients. Furthermore, the found that ST6GAL1 is involved in immune cell enrichment and sialylation modification of PD‐L1 while maintaining its stability may contribute to the development of glycosylation specific antibodies and guide more effective immunotherapy strategies.

## 5 Experimental Section

### Colorectal Cancer Dataset Source and Preprocessing

In this study, 213 and 306 glycogenes were downloaded from HGNC (https://www.genenames.org/) and GSEA (http://www.gsea‐msigdb.org/gsea/index.jsp), respectively. Then, 53 glycogenes were obtained at the intersection of the two genesets. The gene expression data and clinical characteristics of CRC samples were collected from the NCBI GEO (https://www.ncbi.nlm.nih.gov/geo/) database and the public data set of TCGA (https://cancergenome.nih.gov/). A total of 1315 patients were included in the analysis, including patients from the GSE39582, GSE38832, GSE87211 cohort and the TCGA‐COAD/READ dataset. “ComBat” algorithm of R package sva was used to correct the batch effect among the different datasets. The expression of 53 glycogenes at the intersection was combined with the corresponding clinical data for further analysis.

### Unsupervised Clustering for 53 Glycogenes

Based on the expression of 53 glycogenes, unsupervised clustering analysis was used to identify different glycogene clusters, and CRC samples were classified into different clusters for further analysis. Consensus clustering algorithm controls the number of clusters and their stability.^[^ ^23^ ^]^ To ensure the stability of the classification, the above steps were carried out using the “ConsenceClusterPlus” package and repeated 1000 times.^[^ ^30^ ^]^

### Function Annotation and Gene Set Variation Analysis (GSVA)

GSVA is commonly used in the estimation of samples of expression datasets to explore variations in pathway and biological process activity. The “GSVA” R package was used for GSVA enrichment analysis to evaluate the heterology of the different clusters. Gene sets “c2.cp.kegg.v7.5.1.symbols” downloaded from MSigDB, as implemented in the GSVA package (version 1.42.0).

### Analysis of TME Immune Cell Infiltration

Several algorithms such as single sample gene set enrichment analysis (ssGSEA), EPIC, xCELL, and MCPcounter was used to quantify the relative abundance of immune cell infiltration in tumor microenvironment. ESTIMATE algorithm was performed to estimate the immune and stromal cells in CRC.

### Identification of Differentially Expressed Genes (DEGs) between the Three Clusters

To determine DEGs between distinct clusters, the empirical Bayesian algorithm was applied with the “limma” R package^[^ ^31^ ^]^ and an adjusted P value < 0.05 was employed. The Random Survival Forest algorithm was further used to rank the importance of DEGs. The model was trained with 1000 trees. For further analysis, genes with importance = 0 either in GSE39582 cohort or in TCGA cohort were removed.

### Construction of the PCA Score

A scoring algorithm for glycogenes was constructed. The main components of the genes were extracted using PCA. Principal components 1 and 2 were selected as signature scores. Thereafter, to determine the PCAscore for each CRC patient using an approach similar to gene expression grading index:^[^ ^32^ ^]^ *PCAscore* = ∑(*PC*1*_i_* + *PC*2*_i_*). R package “survminer” was used to estimate the best cutoff value for distinguishing high and low risk groups associated with prognosis.

### Collection of Immune‐Checkpoint Blockade Genomic and Clinical Information

Gene expression profiles of publicly available ICI treatments containing detailed clinicopathological information were systematically searched. The study eventually included five immunotherapy cohorts, including GSE78220, GSE126044, GSE176307, IMvigor210, and Kim. The gene expression profiles of biopsy samples before treatment were sorted out and transformed into TPM format for further analysis.

### Single‐Cell RNA Sequencing (scRNA‐Seq) and Data Processing

Four tumor tissue samples were collected from patients who underwent colorectal cancer radical surgery with informed written consent at the Department of General Surgery, Ruijin Hospital, Shanghai JiaoTong University School of Medicine. After removing fat tissue and visible blood vessels, tissue samples were processed into single‐cell suspensions by mechanical and enzymatic dissociation. By following the manufacturer's protocol of 10X Chromium 3′ v3 kit (10X Genomics, Pleasanton, CA), single‐cell suspensions were processed. Library was prepared and sequencing was performed on the NovaSeq 6000 platform (Illumina, Inc., San Diego, CA). In this study, raw sequencing reads were transformed into fastq file by the bcl2fastq2 Conversion Software (v2.20, Illumina) and conducted quality control by FastQC software (Version 0.11.9). Based on the human reference, GRCh38 (GENCODE v32/Ensembl 98), standard pipelines of cell ranger were used to do sequence processing. The cell X gene matrix were then loaded into Seurat (v4.2) for QC, filtering, normalization, Uniform Manifold Approximation and Projection (UMAP) visualization, and clustering. To correct for the batch effect between different samples, and the Harmony method in the harmony package was applied to integrate the complete data set.^[^ ^33^ ^]^ The marker genes of each cluster were identified using FindAllMarkers function in Seurat. The multiple‐test corrected P < 0.05 was used as cut‐off for significance. The activity of glycol‐related metabolism was evaluated with scMetabolism and AUCell package.^[^ ^34^, ^35^ ^]^ Communication intensity between cells within TME were measured by NicheNetr package.^[^ ^36^ ^]^ Top ligand and receptor were shown between glycol‐high and ‐low group.

### Spatial Transcriptomic Data Collection and Analysis

Spatial transcriptome data was available in the Gene Expression Omnibus database under accession number GSE225857. Four human colorectal cancer and two normal tissue samples (10X visium platform) were included in this dataset. Data preparation was conducted using Scanpy workflow.^[^ ^37^ ^]^ Briefly, expression and spatial data from each slide were read in with sc_read_visium function. The only quality control procedure was to screen out mitochondrial genes. After anndata object was constructed, all spatial slides were merged and integrated with Harmony.^[^ ^38^ ^]^ Spots from all six slides were clustered with Leiden algorithm from Scanpy. Cell2location was used for deconvolution and annotation of spatial data.^[^ ^39^ ^]^ The inhouse CRC single cell transcriptomic data was used as reference. Epoch of model training was set as 2000. For colocalization analysis, MISTY was used to identify the relative spatial distribution of spots.^[^ ^40^ ^]^ Six neighboring spots were defined as adjacent distance. 200 µm was set as effective range of cell–cell interaction.

### Patient Sample Collection and Immunohistochemistry (IHC)

The tissue microarray of CRC (n = 72) was collected from Ruijin Hospital, Shanghai Jiao Tong University School of Medicine, and the written informed consent of all patients was obtained at the time of registration (2023ZD0501600). The specific operation of IHC was described earlier.^[^ ^41^ ^]^ The following antibodies were used: anti‐ST6GAL1 (ab 203 304, rabbit; 1:100, Abcam, UK), anti‐CD66b (ab197678, rabbit; 1:200, Abcam, UK), anti‐PD‐L1 (ab 205 921, rabbit; 2 µg ml^−1^, Abcam, UK), Goat Anti‐Rabbit IgG H&L (HRP) (ab97080, goat anti‐rabbit; 1:200, Abcam, UK). The German score was used to evaluate the ST6GAL1 staining intensity and area. The staining intensity score staining area score was the final score, and a score greater than 3 was judged to be positive. The score was 1–4. 1: no staining or sporadic cells; 2: moderate number of cells; 3: large number of cells; 4: super large number of cells.^[^ ^42^ ^]^

### Cell Lines and Cell Culture

Human HEK293T, HT‐29, HCT116, and mouse colon carcinoma MC38 cells were purchased from the China Center for Type Culture Collection (CCTCC; Shanghai, China). HEK293T, HCT116, and MC38 cells were cultured in DMEM (Gibco, Thermo Scientific, Waltham, USA) supplemented with 10% fetal bovine serum (FBS, Gibco), 100 U/ml penicillin, and 100 µg ml^−1^ streptomycin at 37 °C in a humidified atmosphere of 5% CO2. HT‐29 cells were maintained in RPMI 1640 (Gibco, Thermo Scientific, Waltham, USA) medium supplemented with 10% FBS at 37 °C under 5% CO2. The cell lines were genotyped by short tandem repeat profiling and were routinely tested for mycoplasma contamination.

### Establishment of ST6GAL1 Knockdown Cell Lines

According to the manufacturer's instructions, ST6GAL1 was knocked down with short hairpin RNAs (shRNA) (Genomeditech, Shanghai, CHN). In the presence of polybrene (8 µg ml^−1^), tumor cells were transfected with viral particles for 24 h, and then cells were selected with puromycin (2 µg ml^−1^, MCE, #HY‐B1743A, USA) for 1 week. Based on the knockdown efficiency of ST6GAL1 mRNA expression level, two shST6GAL1 clones were selected for this study. The shRNA sequence of lentivirus that inhibits the expression of human or mouse PD‐L1 was shown in the Table S1 (Supporting Information).

### Quantitative Real‐Time Reverse Transcription PCR (qRT‐PCR)

Cells were washed twice with PBS and subjected to total RNA extraction using the RNA‐Quick Purification Kit (Esunbio, Shanghai, CHN). According to the manufacturer's instructions, cDNA was synthesized from 1 µg purified total RNA with the HiScript Q RT Supermix for qPCR (Vazyme, Nanjing, China). qPCR was performed using a real‐time PCR machine with the following primers: 5′‐TTTGCCTTTGCAGATGAGTT‐3′ (ST6GAL1 forward), 5′‐GAAGAAAGACCAGGACGCAG‐3′ (ST6GAL1 reverse), 5′‐GCAGGGCATTCCAGAAAGAT‐3′ (PD‐L1 forward), 5′‐ TCTTGGAATTGGTGGTGGTG‐3′ (PD‐L1 reverse), 5′‐GAAGAAAGACCAGGACGCAG‐3′ (GAPDH forward), and 5′‐AGTTAAAAGCAGCCCTGGTG‐3′ (GAPDH reverse). All the data analyses were performed using the comparative Ct method. Results were first normalized to internal control GAPDH mRNA.

### CCK8 Assay

Cell proliferation assay was conducted by using cell counting kit‐8 (CCK‐8). In brief, 3000 cells per well were plated into the 96‐well plate, and a concentration of 10% CCK8 was added to each well for 2 h. The spectrometric absorbance was measured by microplate reader at 450 nm. The proliferation detection time points were 24, 48, 72, and 96 h.

### Wound Healing Assay

For wound healing assay, CRC cells were seeded into 6‐well plates until the cells reached >95% confluence, and then wounded with 10‐microliter gun tips. The plates were washed with PBS to remove detached cell debris and added with fresh medium. The healing of the wound zone was observed and recorded at 0 and 16 h.

### Colony Formation Assay

CRC cells were inoculated into 6‐well plates at 1000 per well. The foci were formed 10–14 days later. The colonies were fixed by 4% paraformaldehyde for 20 min, and stained with 0.2% crystal violet. Photographs were taken and counted.

### Transwell Assay

The transwell assays were performed using transwell plates (Corning, New York, USA) with 24‐well insert, 8 µm pore size. A total of 2×10^5^ CRC cells were suspended in 500 µL serum‐free medium and seeded into the upper chamber, and a complete medium with 10% FBS was added into the lower chamber. After incubation for 24 h, cells in the upper chamber and those could not pass through the culture membrane were gently removed. Then the transwell plate were fixed with 4% paraformaldehyde at room temperature for 30 min, and then stained with 0.1% crystal violet at 37 °C for 1 h. Three fields of view per chamber were randomly selected, and the mean cell number was calculated.

### Subcellular Fractionation

HCT116 and HT‐29 cells (2×10^6^) were fractionated into cytoplasmic extract and membrane extract using the Subcellular Protein Fractionation Kit (Thermo Fisher Scientific, Waltham, MA, USA) according to the manufacturer's instructions. The cytoplasmic and membrane protein extracts were then subjected to western blotting analysis with specific antibodies against proteins from cellular compartments such as tubulin and ATP1A1.

### Western Blotting

Cells were washed with PBS and then lysed with RIPA Lysis Buffer containing protease and phosphatase inhibitor cocktail (Roche, Indianapolis, IN, USA). Insoluble materials were removed by centrifugation at 14 000 g for 10 min at 4 °C. Equal amounts of protein were separated using 4–12% SDS‐PAGE, transferred to polyvinylidene difluoride (PVDF) membranes, blocked with 5% non‐fat milk at room temperature for 1 h, and incubated with primary antibodies overnight in 4 °C. The membranes were then incubated with secondary antibodies at 37 °C for 1 h. Immunoreactive bands were visualized using an ECL kit (Amersham Biosciences, Piscataway, NJ, USA). All experiments were repeated three times independently. For detailed methods and materials, please refer to the Supporting Information. The antibodies and reagents used in this study were described in detail in Table S2 (Supporting Information).

### Lectin Blotting

The CRC cells were lysed and incubated overnight at 4 °C with rotation using SNA agarose beads (Vector, Burlingame, CA, USA), which specifically recognize α2,6 sialylation. After washing three times with PBS, the lectin precipitates were subjected to western blotting analysis.

### Co‐immunoprecipitation (CO‐IP)

The immunoprecipitation was performed by the Pierce Classic Magnetic IP/Co‐IP Kit (Thermo Fisher Scientific, Waltham, MA, USA) according to the manufacturer's instructions. CRC cells were lysed with lysis buffer. After centrifuging at 13 000 g for 10 min, the supernatant was added with Pierce Protein A/G magnetic beads and incubated with the appropriate antibody overnight at 4 °C. The immunoprecipitants were subjected to western blotting assay according to the protocol above.

### Immunofluorescence Staining (IF)

The cells were rinsed with PBS and fixed in 4% neutral formaldehyde at room temperature for 20 min. After three times of PBS washes, the primary antibodies (ab225793, Abcam, UK; ab279292, Abcam, UK) were added to the cells and incubated overnight at 4 °C. After three additional washes with PBS, the cells were incubated at room temperature with either anti‐rabbit Alexa Fluor 488‐conjugated antibody (4412S, CST, US) or anti‐mouse Alexa Fluor 647‐conjugated antibody (4410S, CST, US). The cells were then washed and stained with DAPI (100 ng mL^−1^). Finally, anti‐fluorescence quencher was used to seal the slide, and cells were analyzed by confocal microscopy (Nikon, Japan). All steps were performed in the dark to preserve fluorescence signals.

### Determination of PD‐L1 Stability

HT‐29 and HCT116 cells were treated with 100 µg mL^−1^ cycloheximide (CHX) (Sigma, St. Louis, MO, USA). The cells were collected at 0, 1, 3, 6, 12, and 24 h. The cell lysates were subjected to western blotting with anti‐PD‐L1 and anti‐GAPDH antibodies.

### Animal Experiments and Tumor Infiltration Lymphocyte Analysis

All animal experiments were conducted in compliance with the guidelines approved by the Institutional Animal Care and Use Committee of Ruijin Hospital, Shanghai Jiao Tong University School of Medicine (AUP‐20241023‐01). To establish a subcutaneous tumor model, stably transfected MC38 cell lines (1×10^6^ cells/mouse) were subcutaneously injected into the hind legs of 6‐week‐old female C57BL/6 mice (5 mice/cell line). After tumor formation, the tumor volume of the mice was measured once a day using the formula: tumor volume (mm3) = π/6 × length × width^2. In the In vivo treatment subcutaneous tumor model, when the volume of the subcutaneous tumor reached 100 mm^3^, the mice were randomly distributed into eight groups: NC group, NC+aPD‐L1 group, KD1 group, KD1+aPD‐L1 group, KD2 group, KD2+aPD‐L1 group, OE group, and OE+aPD‐L1 group. The treated groups were treated with anti‐mouse PD‐L1 plus Cisplatin, while control groups were treated with equal volumes of PBS solution. The anti‐mouse PD‐L1 antibody was administered via intraperitoneal injection (1 mg in 1 mL per mouse) every two days for up to three weeks, and Cisplatin was administrated via intraperitoneal injection (3 mg kg^−1^ per mouse) every week for up to three weeks. The weight and tumor volume of mice were regularly observed every three days. After 21 days, the tumors were isolated removed and weighed for further analysis.

### Statistical Analysis

Molecular experimental and clinical data were analyzed using GraphPad Prism software software version 8.0.2 (GraphPad Software, San Diego, CA, USA). Data are presented as mean ± standard deviation (SD), and each molecular biology experiment was performed at least three times. Student's t test, Chi‐square test or Fisher exact test were used to compare the significant differences between the two groups. Single factor analysis of variance (ANOVA) was used to determine the significant differences among groups. The survival curve was calculated by Kaplan–Meier method, and the difference was evaluated by logarithmic rank test (log‐rank). For immunofluorescence detection, we performed at least three independent experimental repeats and analyzed the fluorescence intensity and colocalization of stained proteins using IMAGE J (version 2.1.0) software. Spearman correlation analysis was used to determine the correlation between IHC scores and protein expression. All the statistical tests were bilateral. The P values in the figures were depicted with the following symbols: *p < 0.05, **p < 0.01, ***p < 0.001.

### Ethic statement

The human (2023ZD0501600) and animal experimental protocol (AUP‐20241023‐01) was approved by the Ethics Boards of Ruijin Hospital, Shanghai Jiao Tong University School of Medicine).

## Conflict of Interest

The authors declare no conflict of interest.

## Author Contributions

X.X., J.L., and W.Q. contributed equally to this work. The conceptualization of the study was carried out by B.F., X.Y., S.Z., and B.L. Methodology was developed by X.Y., S.Z., and L.Z., while software was implemented by X.X., C.D., W.Q., and H.Z. Validation was performed by W.Q., D.S., X.X., and Y.H. Formal analysis was conducted by X.Y., X.X., W.Q., D.S., J.Y.L., and Z.C. The investigation was undertaken by J.L., W.Q., D.S., H.Z., and M.Y. Resources were provided by B.F. Data curation was managed by X.X., D.S., X.Y., Z.C., J.L., and J.Y.L. The original draft of the manuscript was written by X.X. and J.Y.L., while B.F., X.Y., B.L., S.Z., X.X., and J.Y.L. contributed to review and editing. Visualization was handled by X.X., JY.L., X.Y., and W.Q. Supervision was provided by B.F., M.Z., X.Y., B.L., and S.Z. Project administration was managed by B.F. All authors read and approved the final manuscript.

## Supporting information



Supporting Information

Supporting Information

Supporting Information

## Data Availability

The data that support the findings of this study are available on request from the corresponding author. The data are not publicly available due to privacy or ethical restrictions.
