# Supplementary material for: ST6GAL1‐Mediated Sialylation Stabilizes PD‐L1 and Drives Immunosuppressive Tumor Microenvironment in Colorectal Cancer
Source: Adv Sci (Weinh). 2025 Aug 22;12(42):e06225. doi: 10.1002/advs.202406225 (PMC12622430; doi:10.1002/advs.202406225)
Supplement: Supplementary file 1 — Supporting Information [file ADVS-12-e06225-s003.pdf]

Supplementary information

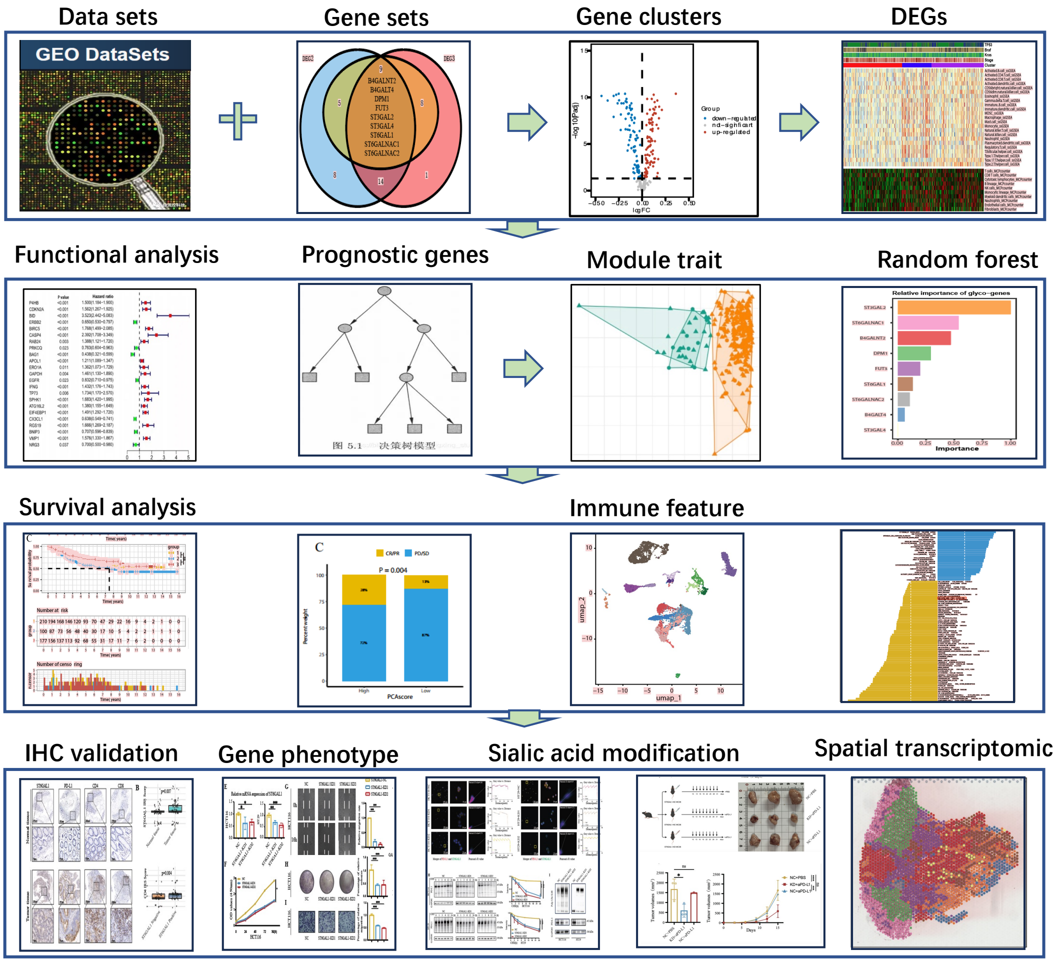

Figure S1. The flow chart of this study.

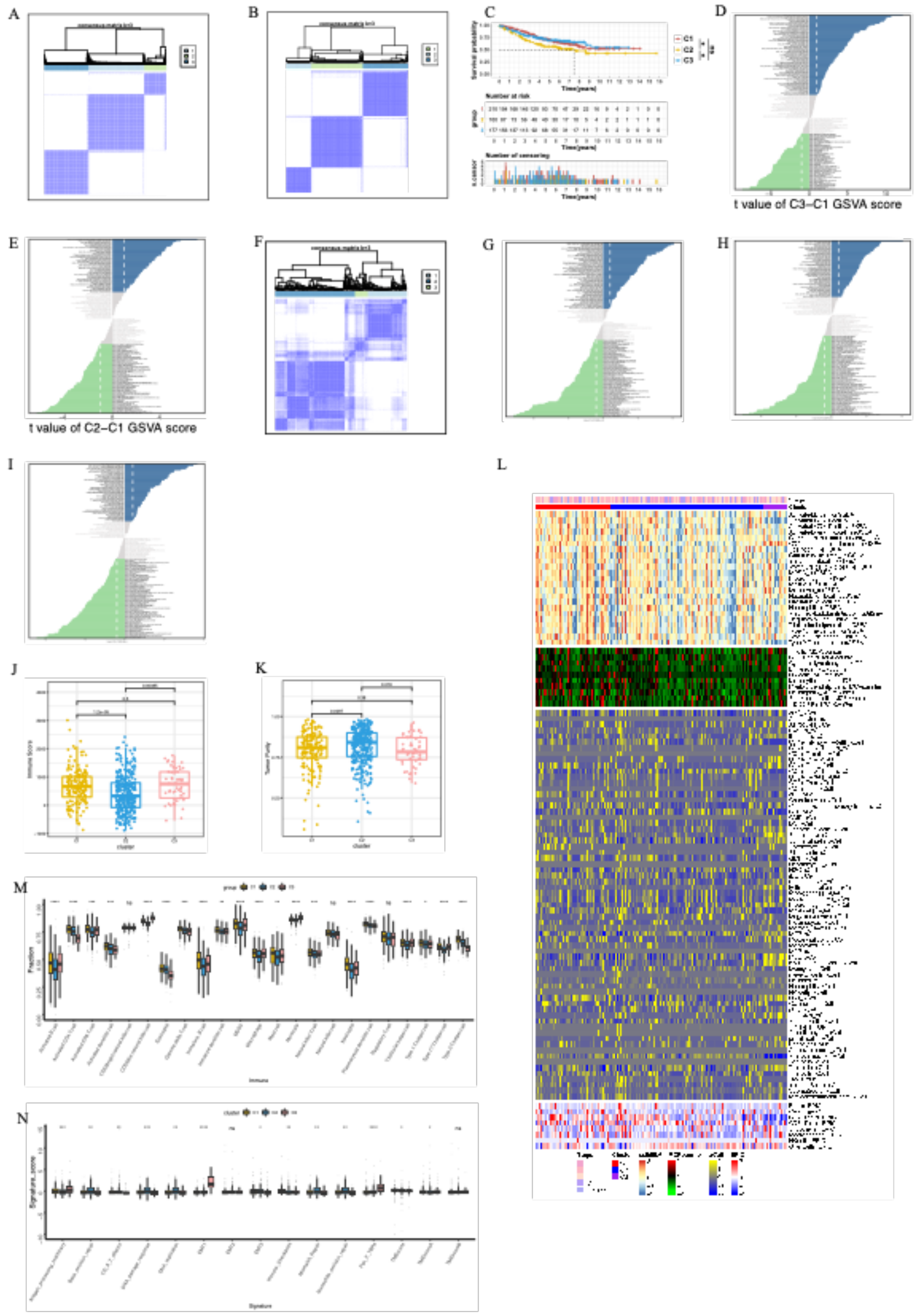

Figure S2. The landscape of immuno-cell infiltration in the TME of CRC.

A. In cohorts of GSE39582, GSE38832, and GSE87211, a color heat map of consistency matrix with k = 3 obtained by consistency clustering. The color gradient indicates the consistency values between 0 and 1, with white representing 0 and dark blue representing 1. **B.** The consistency clustering was applied to obtain a colored heat map of the consistency matrix corresponding to k=3(In the cohort of GSE39582). **C.** Kaplan-Meier curves of overall survival (OS) for different glyco-gene clusters in the GSE39582 cohort. **D-E.** The difference of KEGG pathway between **(D)** cluster C3 and cluster C1 **(E)** cluster C2 and cluster C1 was compared by GSVA score in the cohort of GSE39582. **F.** In the cohort of TCGA, a color heat map of consistency matrix with k = 3 obtained by consistency clustering. **G.** The difference of

KEGG pathway between cluster C3 and cluster C2 was compared by GSVA score in TCGA cohort. **H.** The difference of KEGG pathway between cluster C2 and cluster C1 was compared by GSVA score in TCGA cohort. **I.** The difference of KEGG pathway between cluster C3 and cluster C1 was compared by GSVA score in TCGA cohort. **J-K.** The immune score **(J)** and stromal score **(K)** and of the three glycogene clusters were compared in TCGA cohort. **L.** Heatmap shows the GSVA score of representative tumor-infiltrating immune cells curated from intersection in distinct glycogene clusters (TCGA cohort). **M.** The fraction of tumor-infiltrating immune cells in three glycogene clusters. The statistical differences of three glycogene clusters were compared by Kruskal-Wallis test. **N.** Enrichment level of the three glycogene clusters in the classical signaling pathway (TCGA cohort). *p < 0.05; **p < 0.01; ***p < 0.001; ****p < 0.0001; ns, no significant.

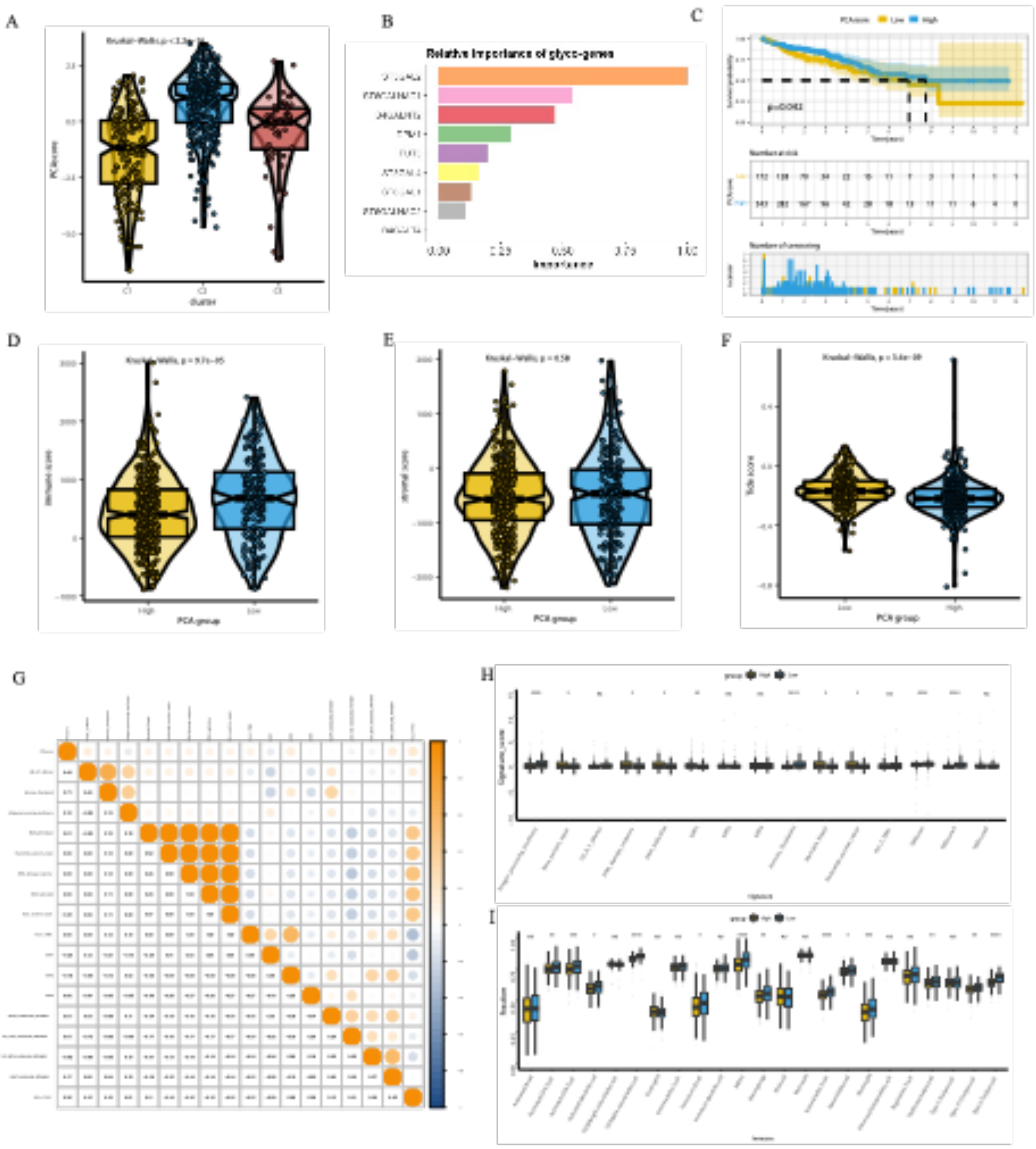

Figure S3. Construction and functional annotation of glycogene signature in TCGA cohort. A. Differences in PCAscore among three glycogene clusters (TCGA cohort, p=2.2e-16, Kruskal-Wallis test). **B.** Random Forest method was used to show the importance of common DEGs among clusters in prognosis (TCGA cohort). **C.** Kaplan-Meier curves for patients with high and low PCA scores in the TCGA cohort. Log-rank test, p=0.042. **D-F.** Differences in immune score **(D)** stromal score **(E)** and tide score **(F)** between high and low PCA score subgroups in TCGA cohort. **G.** Correlations between PCAscore and the known gene signatures using Spearman analysis. **H.** Enrichment level of high and low PCAscore subgroups in the classical signaling pathway. **I.** The fraction of tumor-infiltrating immune cells in high and low PCAscore subgroups.

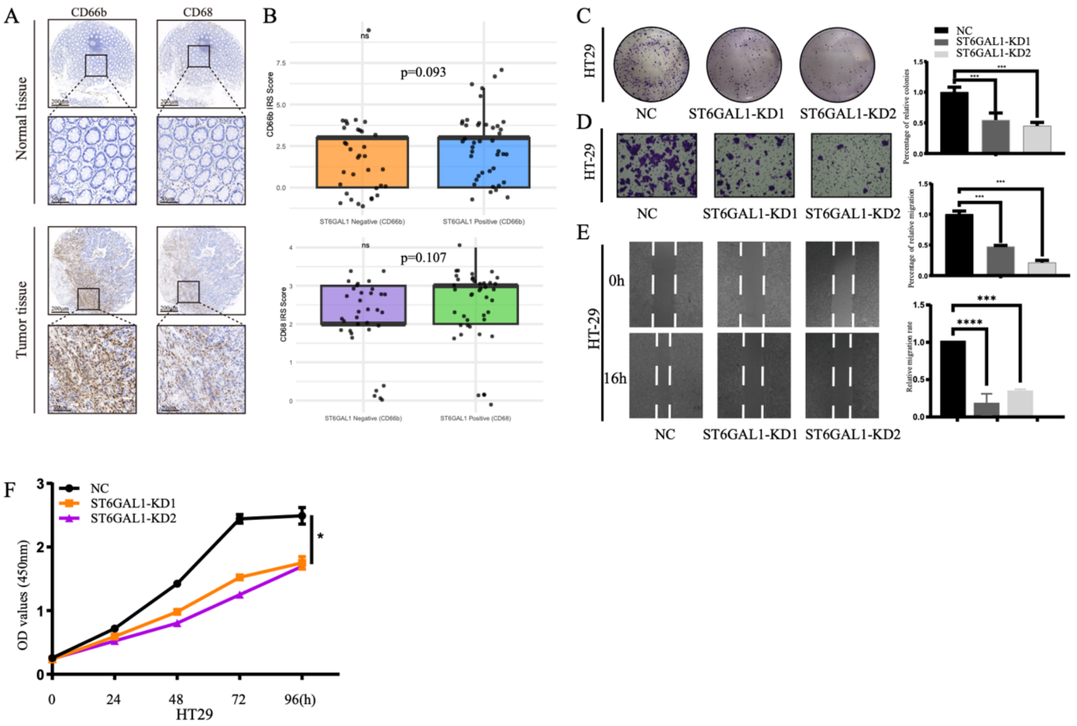

Figure S4. ST6GAL1 mediates the malignant phenotype in HT29 cell line. A-B. Immunohistochemical analysis of CD66b and CD68 in representative CRC tissues and normal tissues. Comparison of IRS scores of CD66b and CD68 in ST6GAL1 positive and negative groups of CRC tumor tissues. n=76. Scale bar: 200 μm and 50 μm. **C-F.** The proliferative ability of the transfected CRC cell line was assessed using the CCK8 assay and colony formation assay (n=3), while the migration ability was evaluated through the healing and transwell assay (n=3). Data were presented as mean ±SD. Statistical analysis was performed with one-way ANOVA followed by Tukey's test, *p <0.05, ***p <0.001, ****p <0.0001.

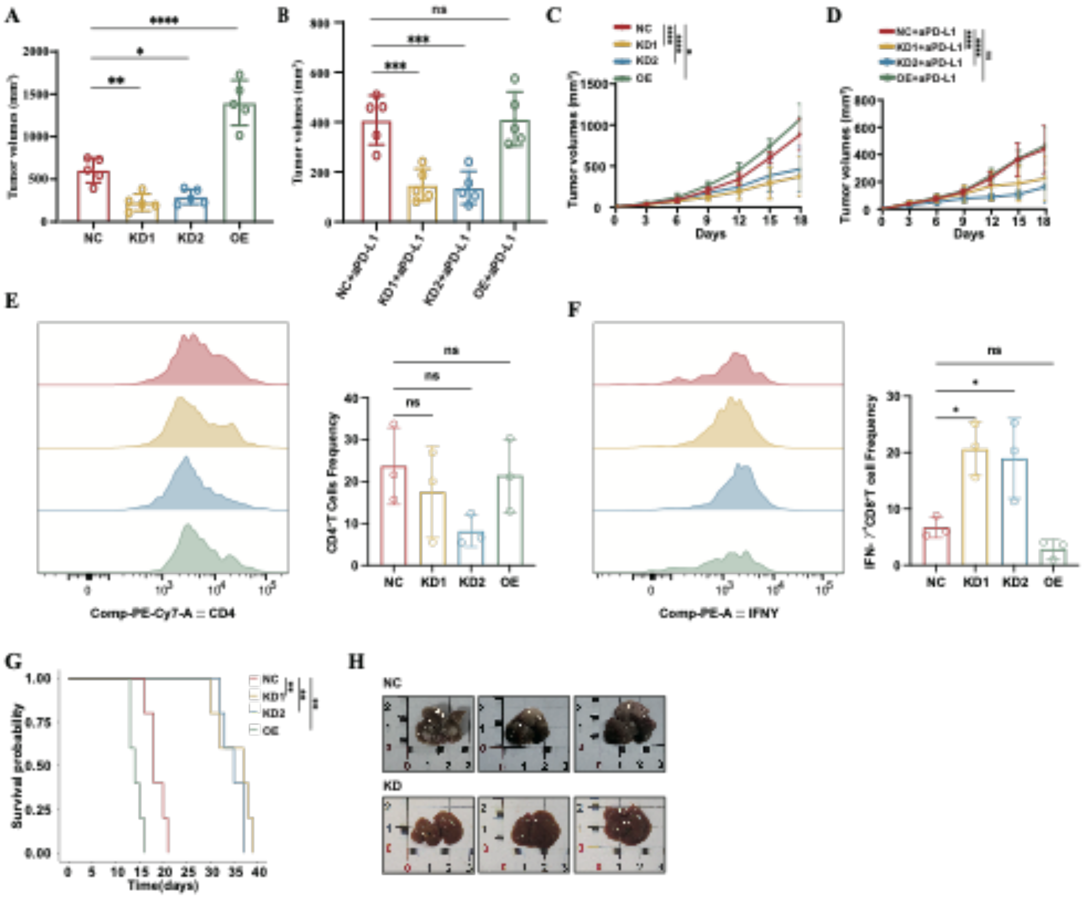

Figure S5. ST6GAL1 promotes CRC progression in vivo.

A-B. statistical analysis of tumor volumes (n=5). **C-D.** growth curves of subcutaneous tumor (n=5). **E-F.** Representative flow cytometry histogram plots (left) and proportion (right) of tumor-infiltrating $CD4^{+}$ T cells and IFN-γ^{+} $CD8^{+}$ T cells in tumor tissues (n=5). **G.** survival differences (n=5). **H.** The representative images shown macroscopic observations of metastases in liver (n=3). Data were presented as mean ±SD. P values of Kaplan–Meier survival curves were calculated by log-rank test. Statistical analysis in other panels was performed with one-way ANOVA followed by Tukey's test, ns no statistical significance; *p <0.05, ** p <0.01, ***p <0.001, ****p <0.0001.

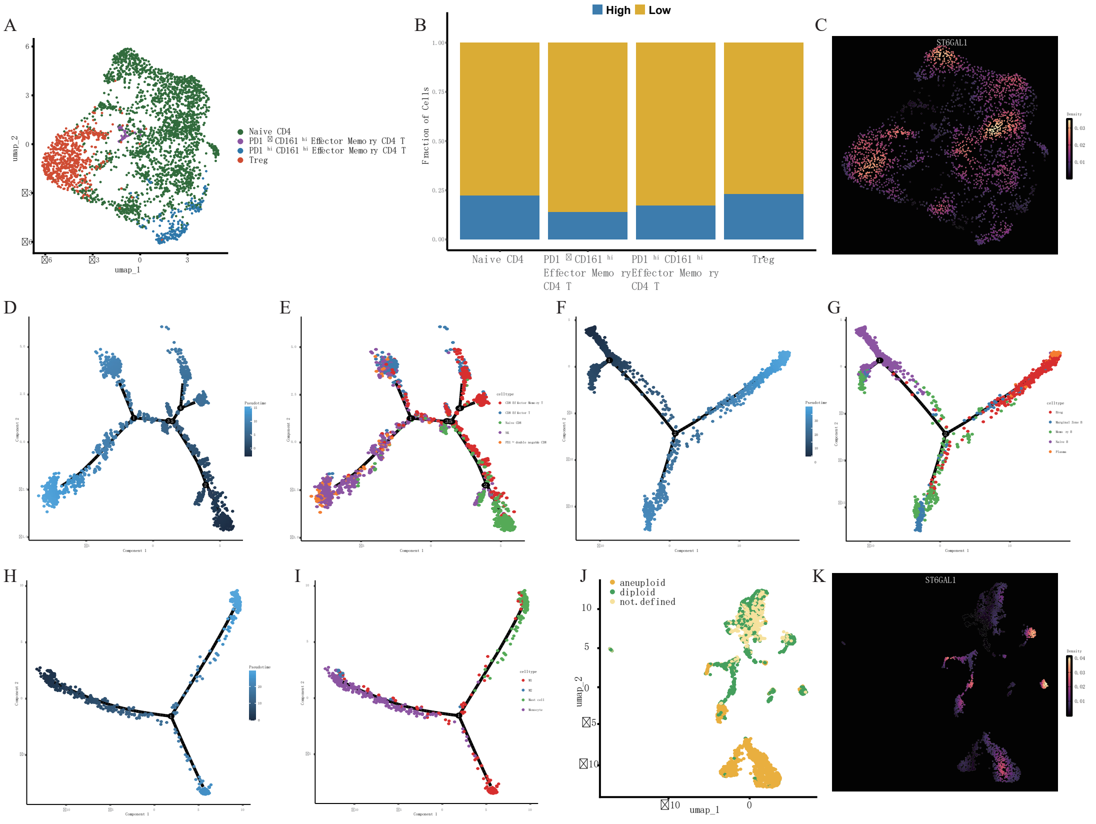

Figure S6. Regulation of TME Immune Cell Infiltration by ST6GAL1

A. UMAP plot of CD4+ T cells following subpopulation annotation. **B.** Proportions of different CD4+ T cell subsets in high and low ST6GAL1 expression groups. **C.** UMAP plot showing ST6GAL1 expression in CD4+ T cells. **D-E.** The cell trajectory analysis of CD8+ T cells. **F-G.** The cell trajectory analysis of B cells. **H-I.** The cell trajectory analysis of monocytes and macrophages. **J.** The aneuploid (tumor) cells and diploid (normal) cells were mapped in the UMAP graph using copykat algorithm. **K.** UMAP plot showing ST6GAL1 expression in Epithelial cells.

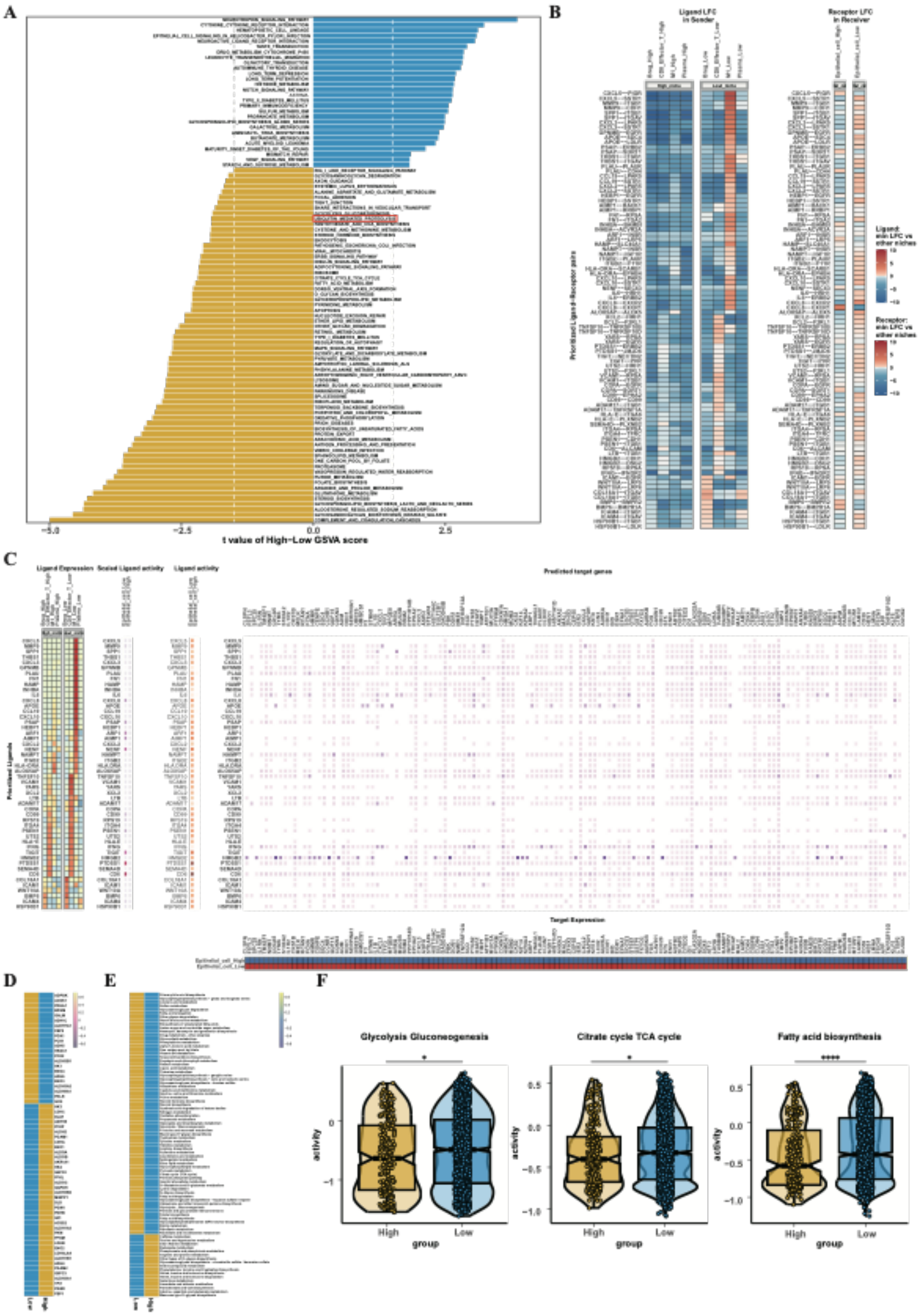

Figure S7. ST6GAL1 Mediates Cellular Crosstalk and Metabolic Dysregulation in TME. A. GSVA scoring to compare differences in KEGG pathways in epithelial cells between high and low ST6GAL1 expression groups. **B.** Top 90 ligand-receptor pairs involving epithelial cells, Breg cells, CD8+ effector T cells, M1 macrophages, and plasma cells. **C.** Ligands and potential receptors involving epithelial cells and Breg cells, CD8+ effector T cells, M1 macrophages, and plasma cells; on the left, interactions between epithelial and immune cells are shown, sorted by the top 50 ligand activities; the right side

heatmap displays the regulatory potential of downstream target genes. **D-E.** Differences in Metabolism between high and low ST6GAL1 expression groups, including differences in glycolysis-related genes **(D)** and metabolic pathways **(E)**. **F.** Differences in glycolysis, the citric acid cycle (TCA cycle), and fatty acid biosynthesis pathways between high and low ST6GAL1 expression groups.

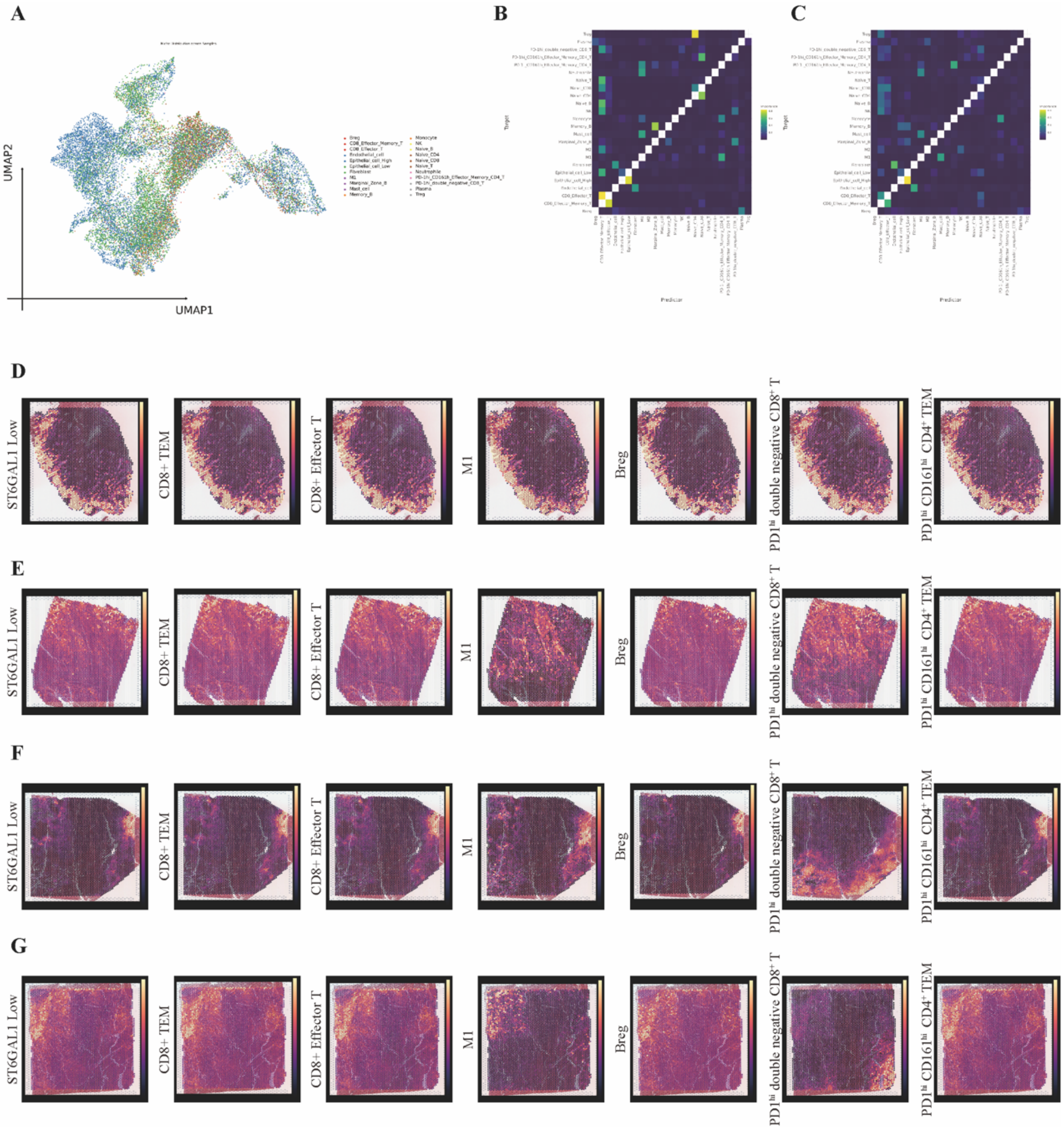

Figure S8. Spatial transcriptome described the spatial distribution of ST6GAL1 A. The regional subgroups with cell annotation based on UMAP downscaling and niches. **B.-C.** Niche colocalization patterns at intra **(B)** and para-regions **(C)**, with color intensity increasing from black to yellow to indicate the relative importance of each pattern. **D-G.** Tumor sample 3 **(D)**, sample 4 **(E)** and normal sample 1 **(F)** and sample 2 **(G)** showing density plots of different cell infiltrastions within the TME. Yellow color intensity indicates higher levels of cell infiltration.
