# Supplementary figures and images for: ST6GAL1‐Mediated Sialylation Stabilizes PD‐L1 and Drives Immunosuppressive Tumor Microenvironment in Colorectal Cancer (part 2 of 2)
Source: Adv Sci (Weinh). 2025 Aug 22;12(42):e06225. doi: 10.1002/advs.202406225 (PMC12622430; doi:10.1002/advs.202406225)

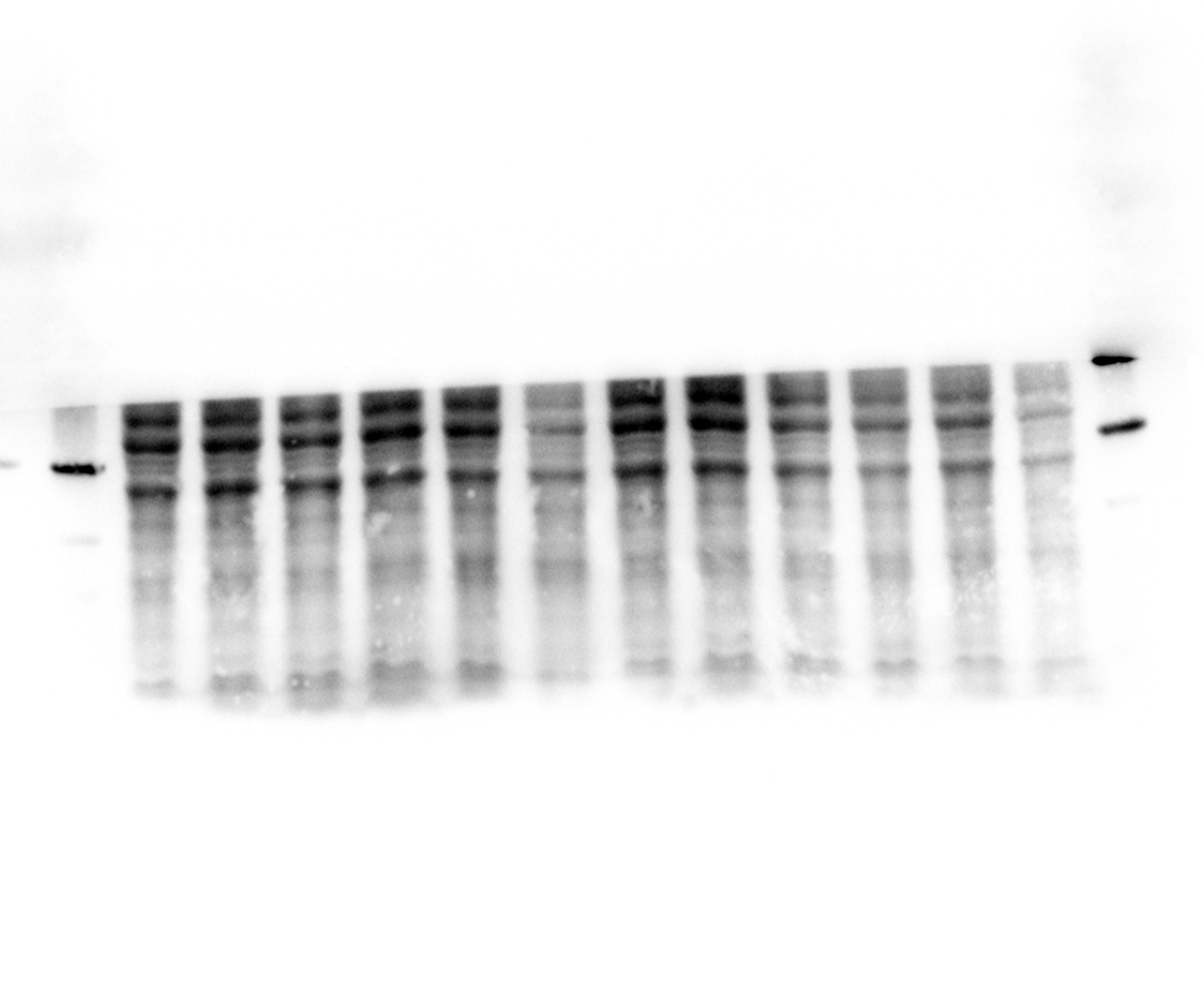

Supplement: Supplementary file 2 — Supporting Information [file ADVS-12-e06225-s001.zip › CHX/3-AB-PD-L1-116SH2 TQ-4S-11.Tif]

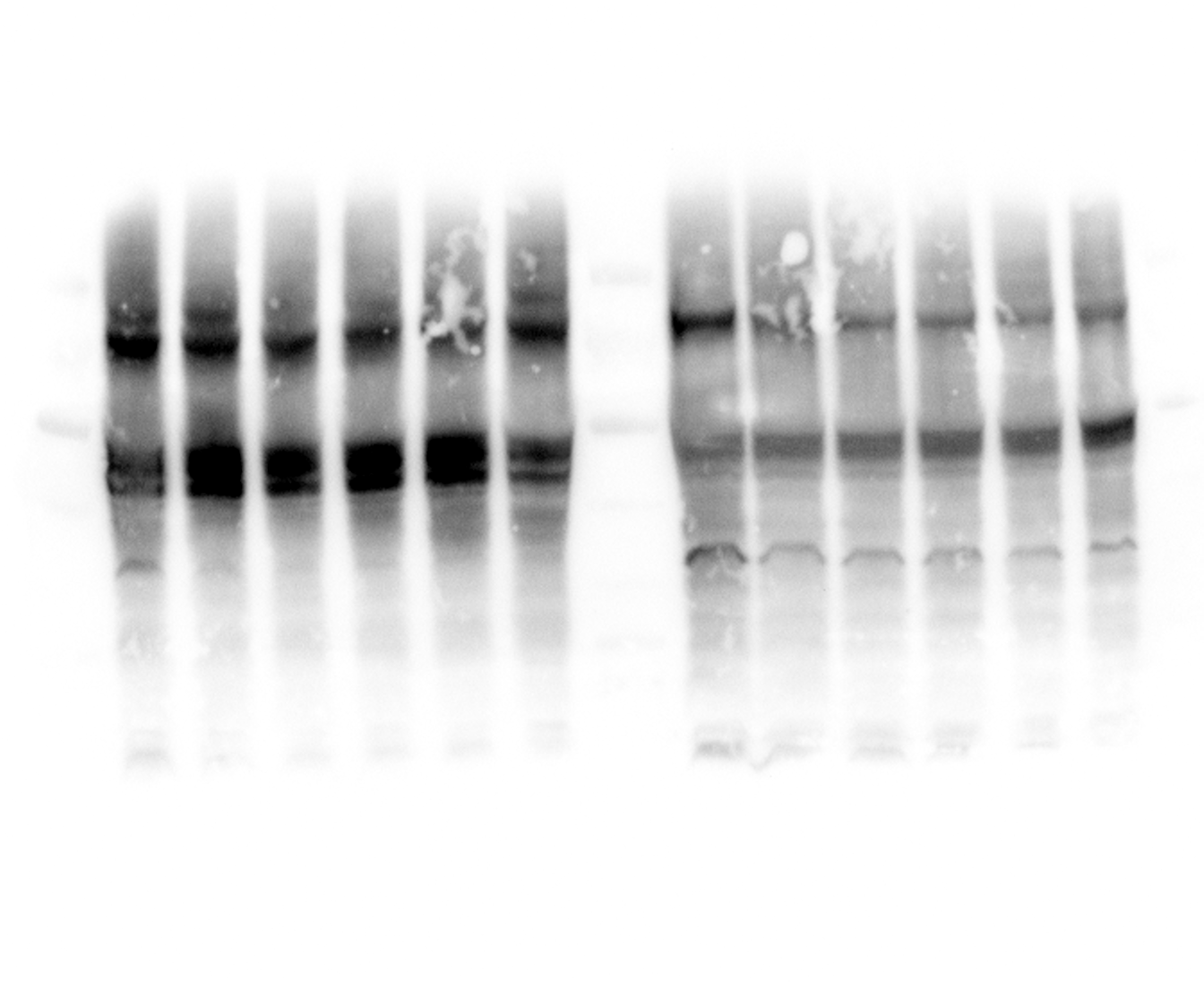

Supplement: Supplementary file 2 — Supporting Information [file ADVS-12-e06225-s001.zip › CHX/3-CHX-116WT------.Tif]

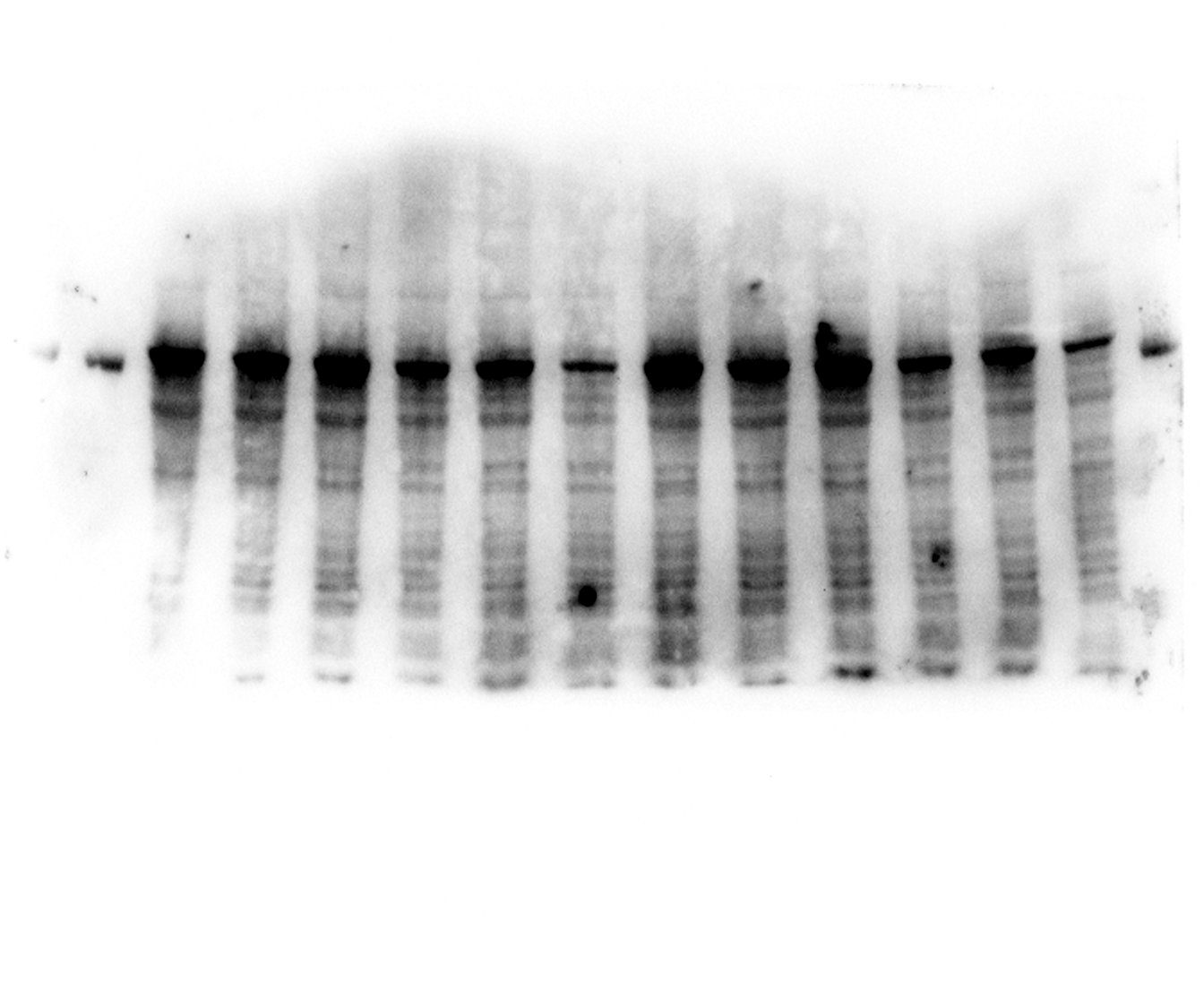

Supplement: Supplementary file 2 — Supporting Information [file ADVS-12-e06225-s001.zip › CHX/3-CHX-H1 H1 -2-5S-6.Tif]

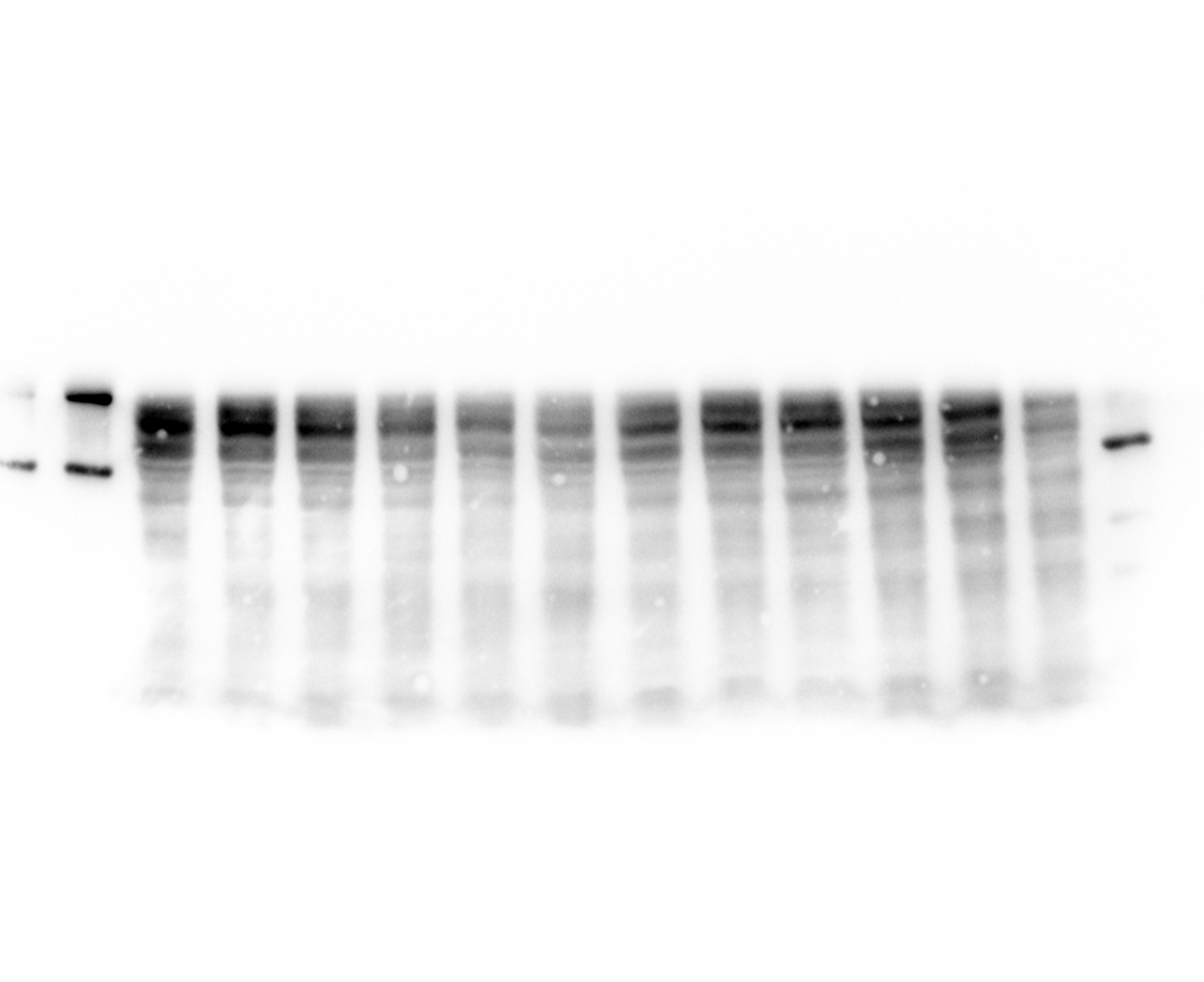

Supplement: Supplementary file 2 — Supporting Information [file ADVS-12-e06225-s001.zip › CHX/3-CHX-PD-L1-116SH2 .......-3S-2.Tif]

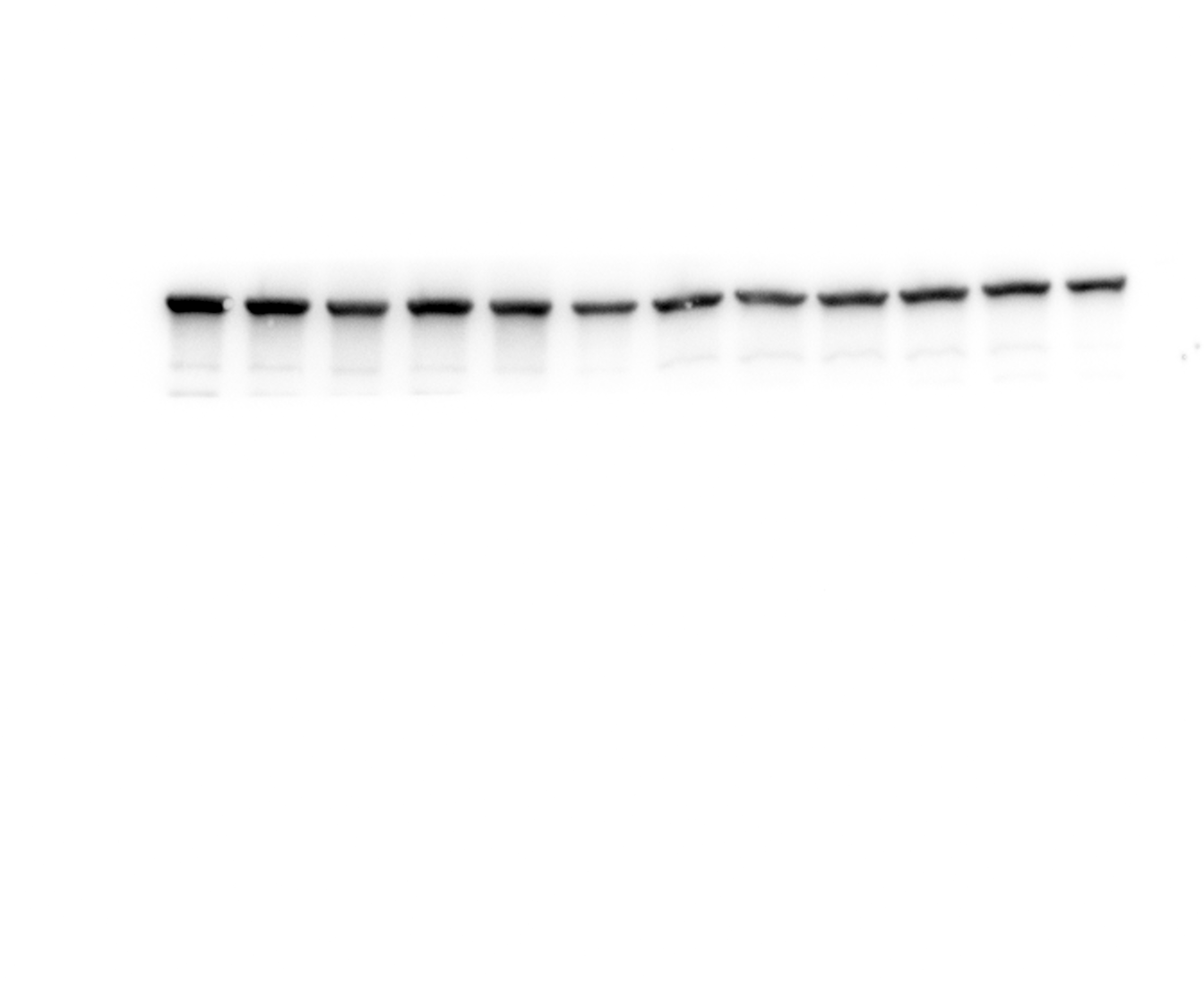

Supplement: Supplementary file 2 — Supporting Information [file ADVS-12-e06225-s001.zip › CHX/3-GAP-CHX-CSTPDL1-116SH2-HWT-1S.Tif]

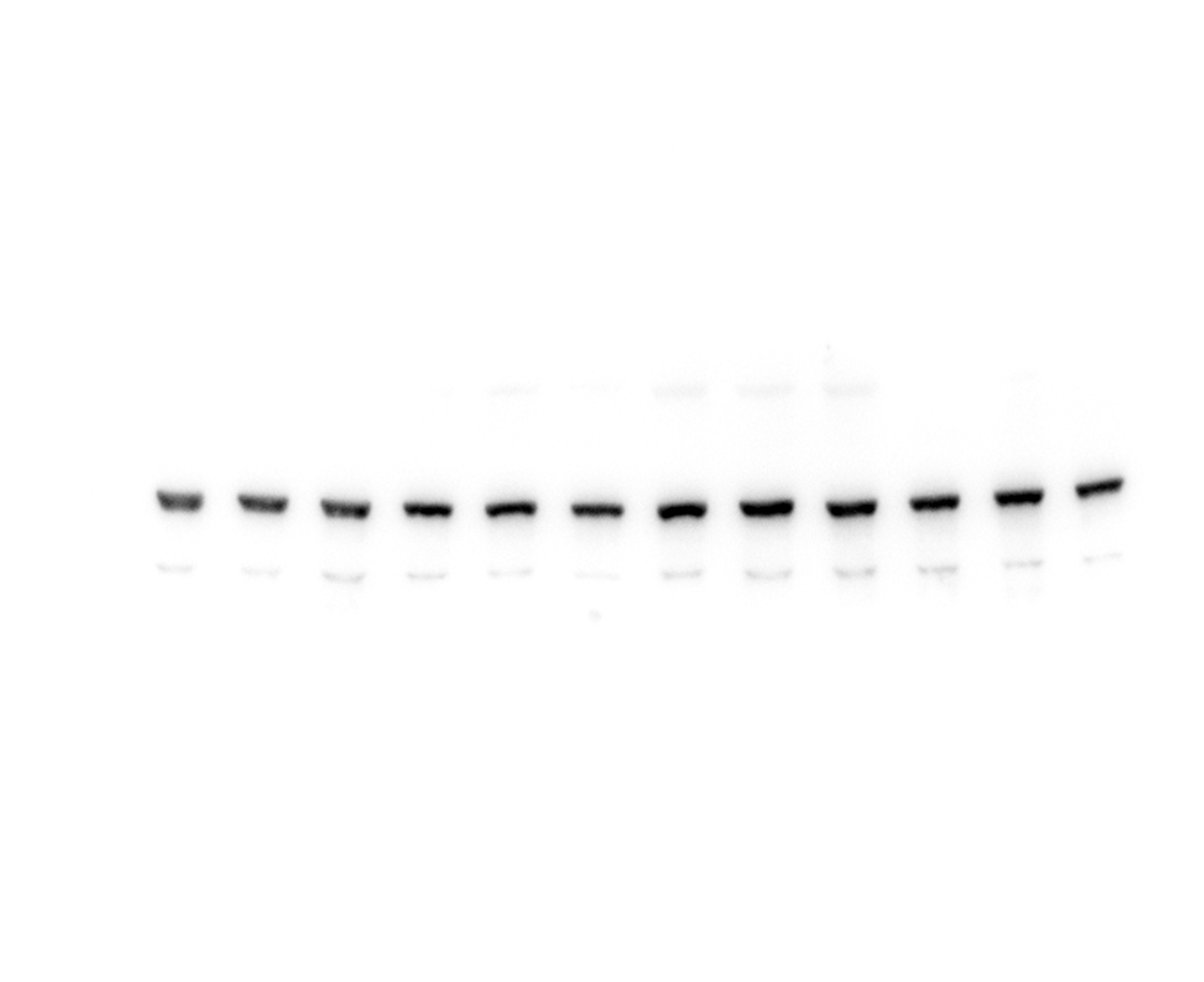

Supplement: Supplementary file 2 — Supporting Information [file ADVS-12-e06225-s001.zip › CHX/3-GAP-CHX-H1 H1 -0.8S.Tif]

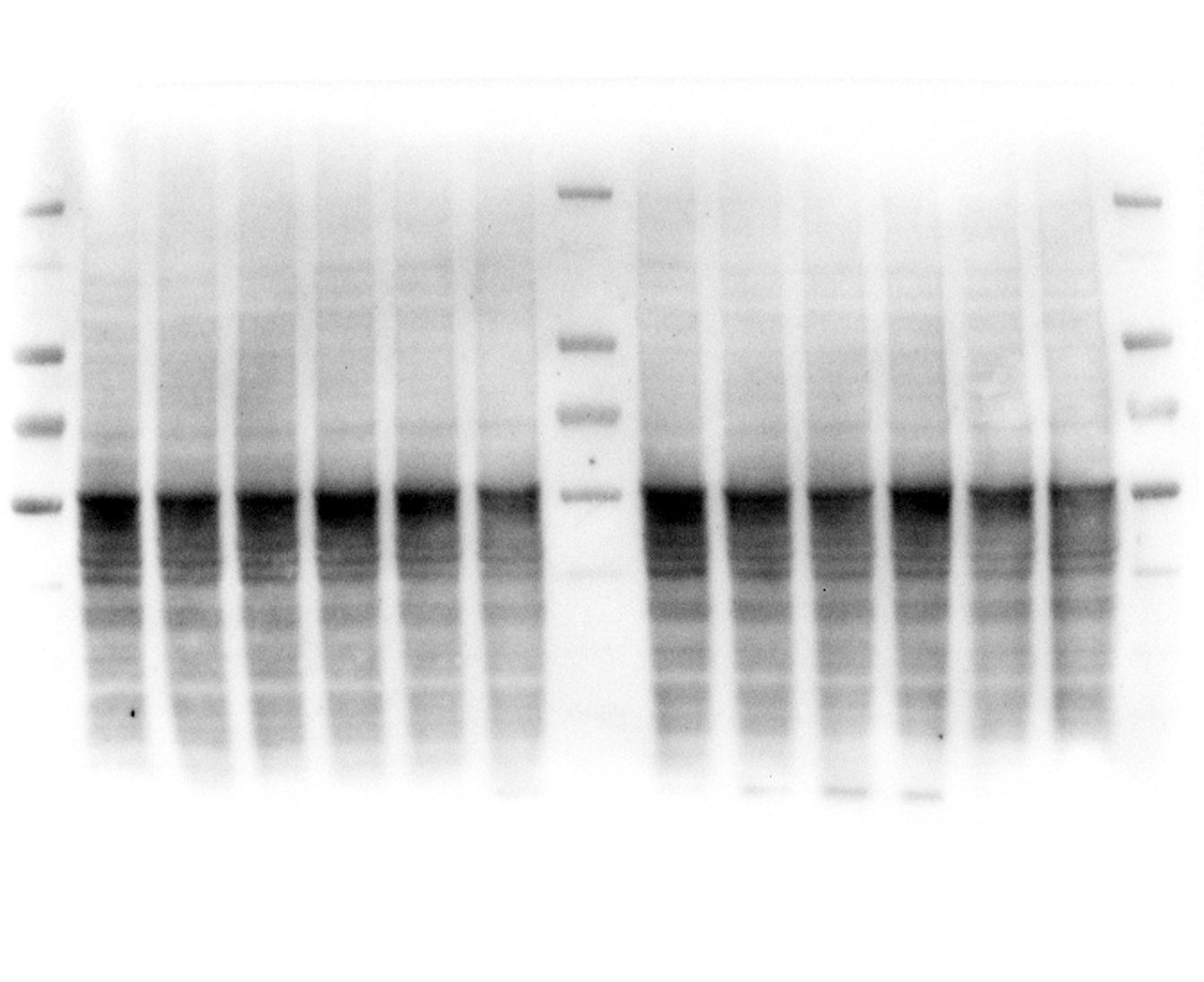

Supplement: Supplementary file 2 — Supporting Information [file ADVS-12-e06225-s001.zip › CHX/3CHX-H- TQ .Tif]

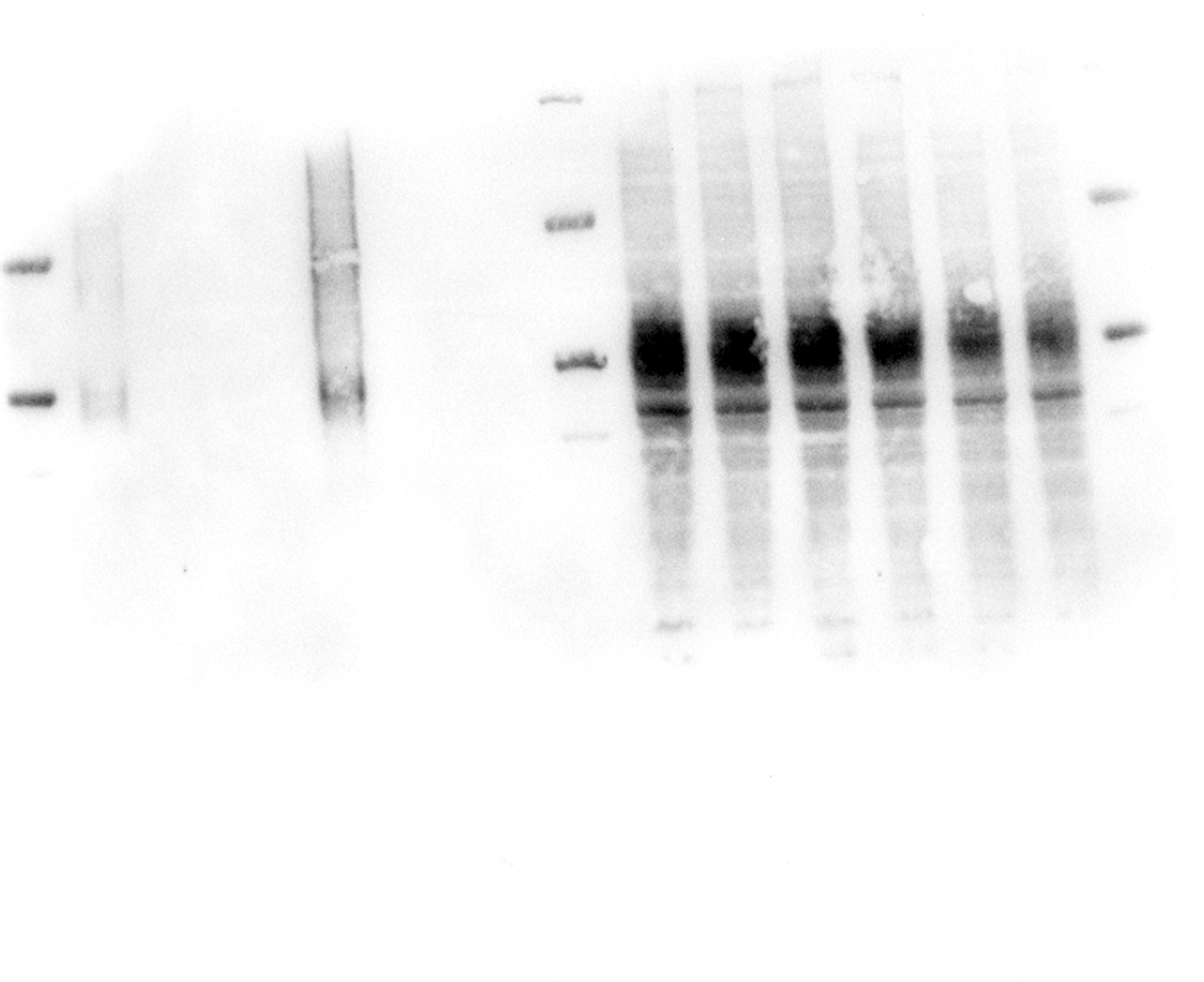

Supplement: Supplementary file 2 — Supporting Information [file ADVS-12-e06225-s001.zip › CHX/3CHX-H2.Tif]

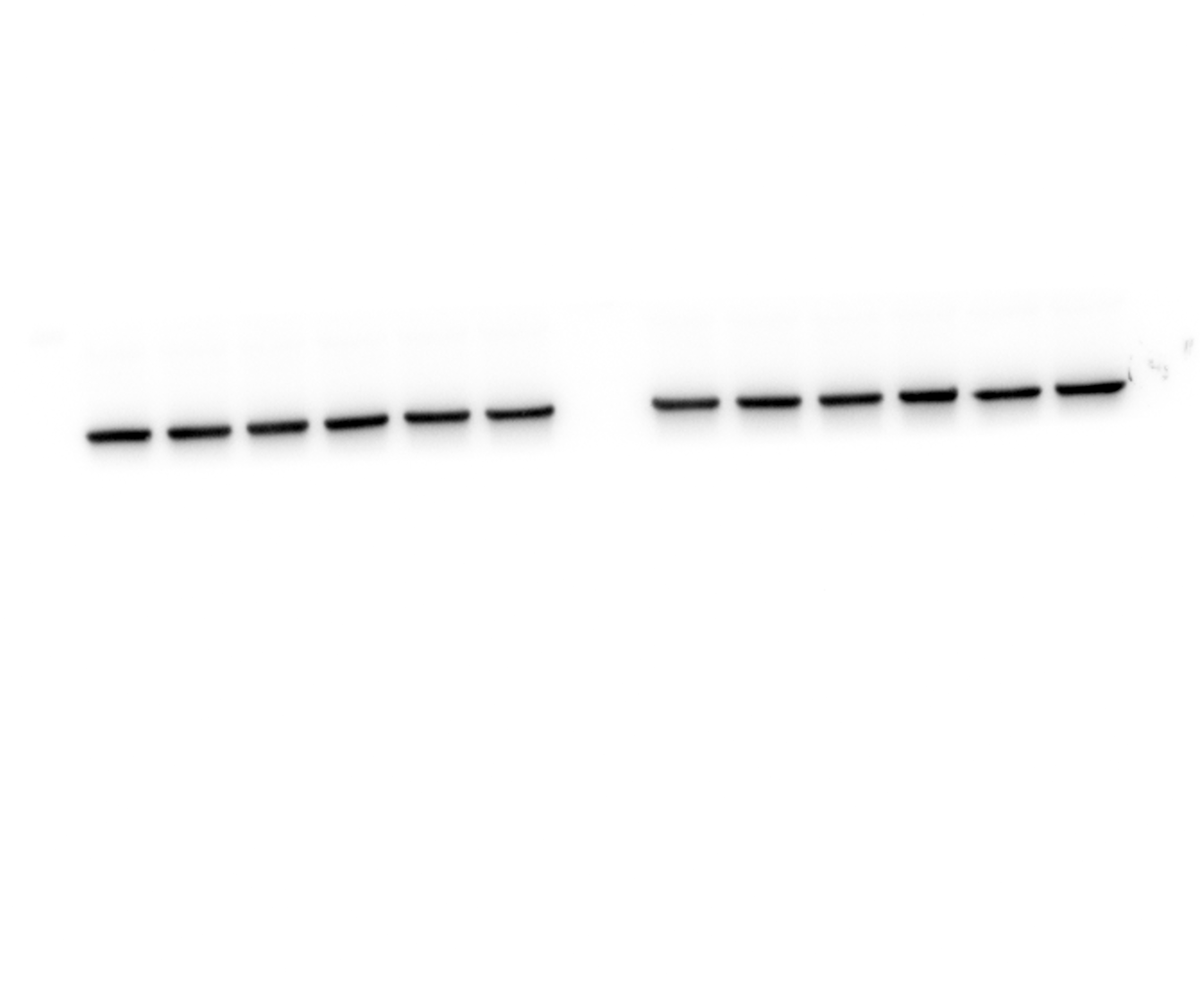

Supplement: Supplementary file 2 — Supporting Information [file ADVS-12-e06225-s001.zip › CHX/3GAP-H-TQ .Tif]

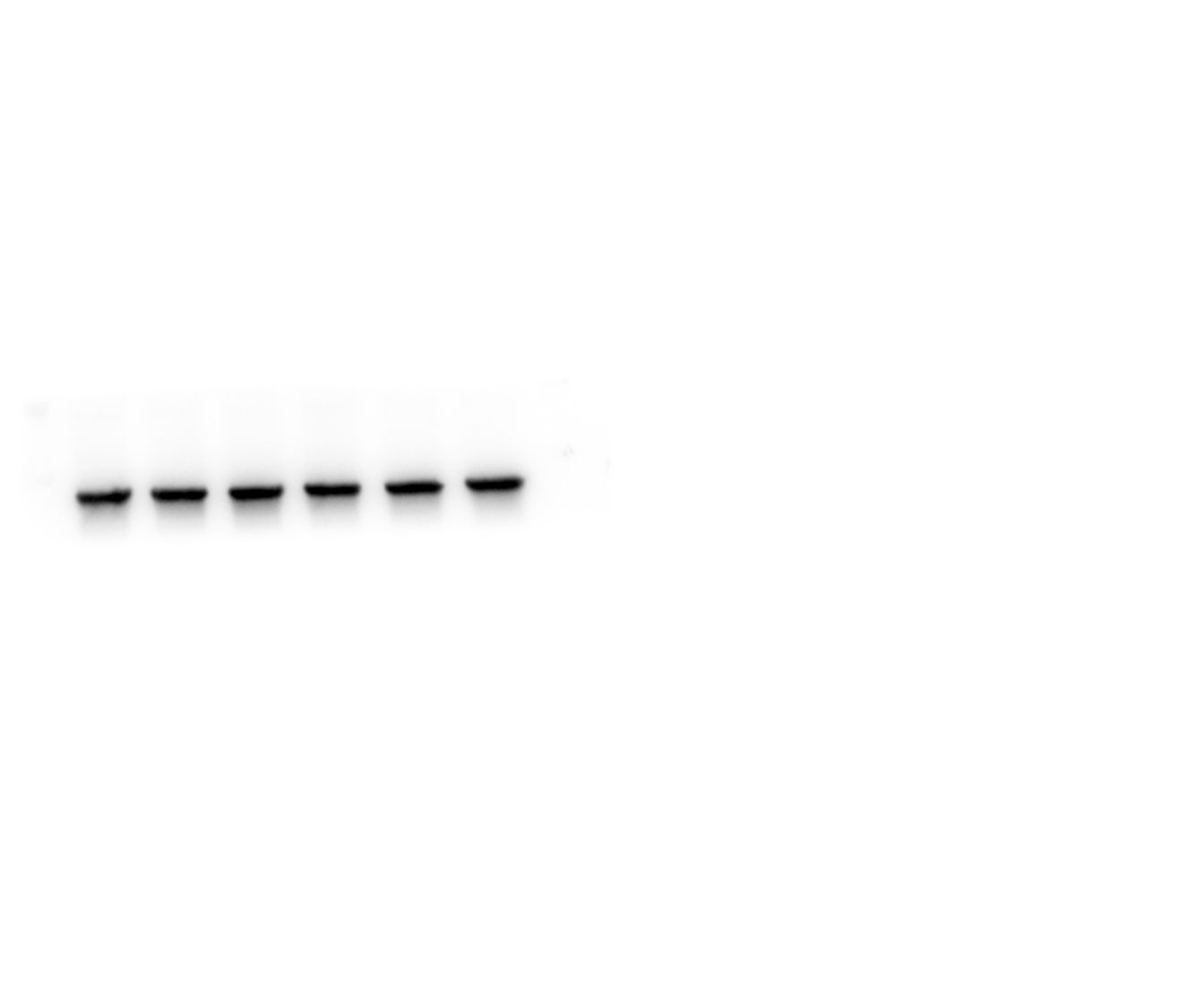

Supplement: Supplementary file 2 — Supporting Information [file ADVS-12-e06225-s001.zip › CHX/3GAP-H2.Tif]

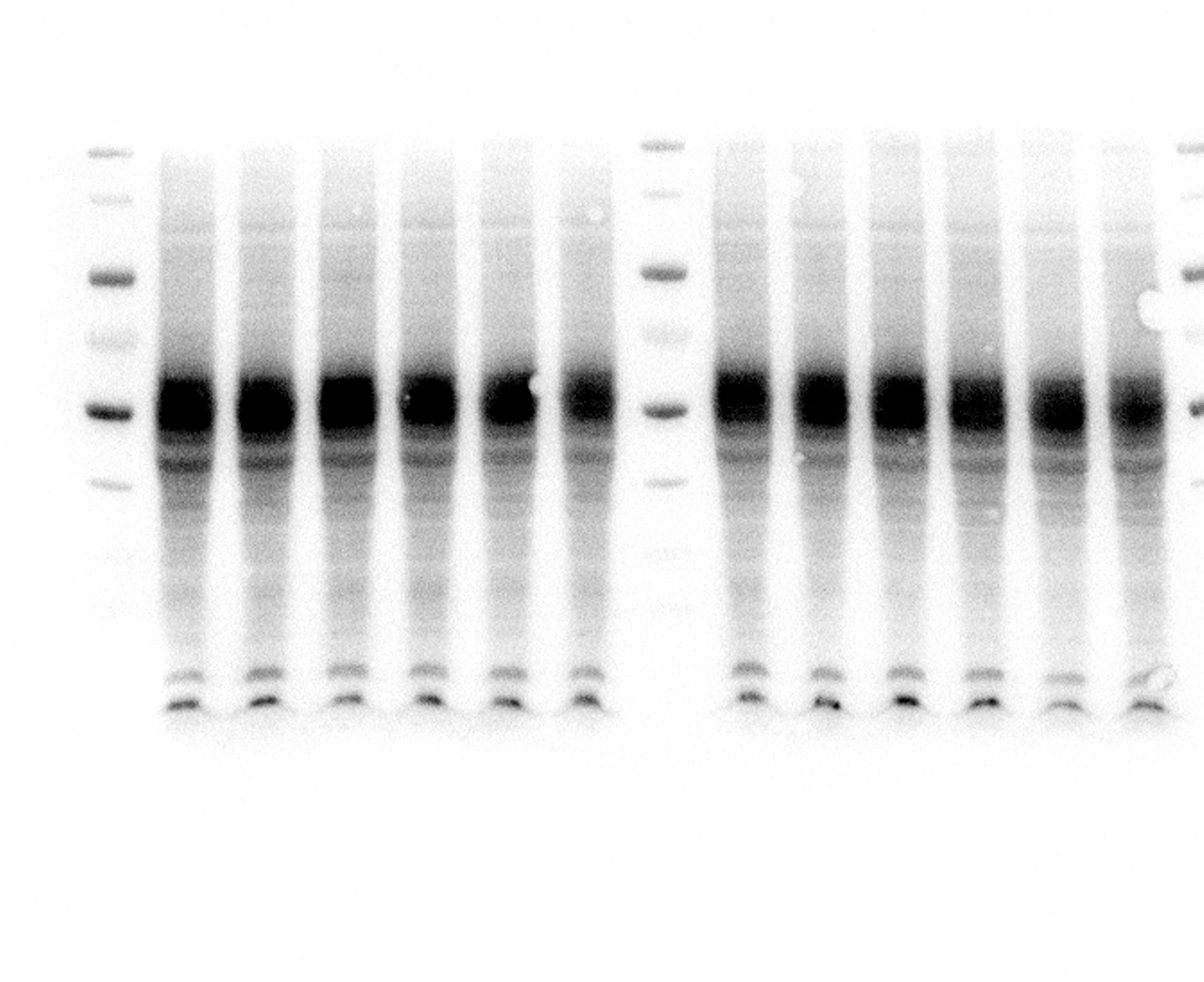

Supplement: Supplementary file 2 — Supporting Information [file ADVS-12-e06225-s001.zip › CHX/4-CHX-H-TQ .Tif]

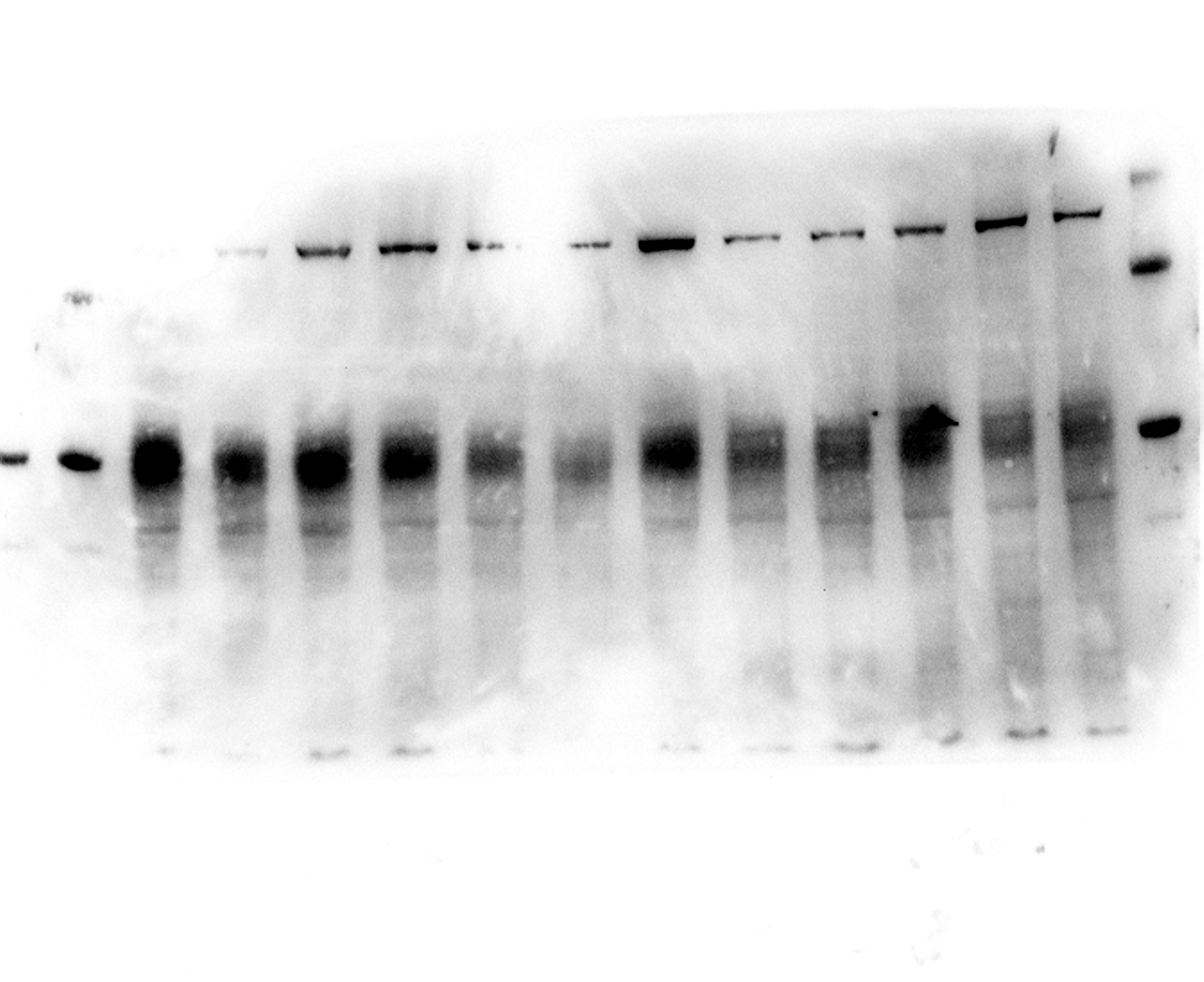

Supplement: Supplementary file 2 — Supporting Information [file ADVS-12-e06225-s001.zip › CHX/4-CHX-H1 & H2-5.Tif]

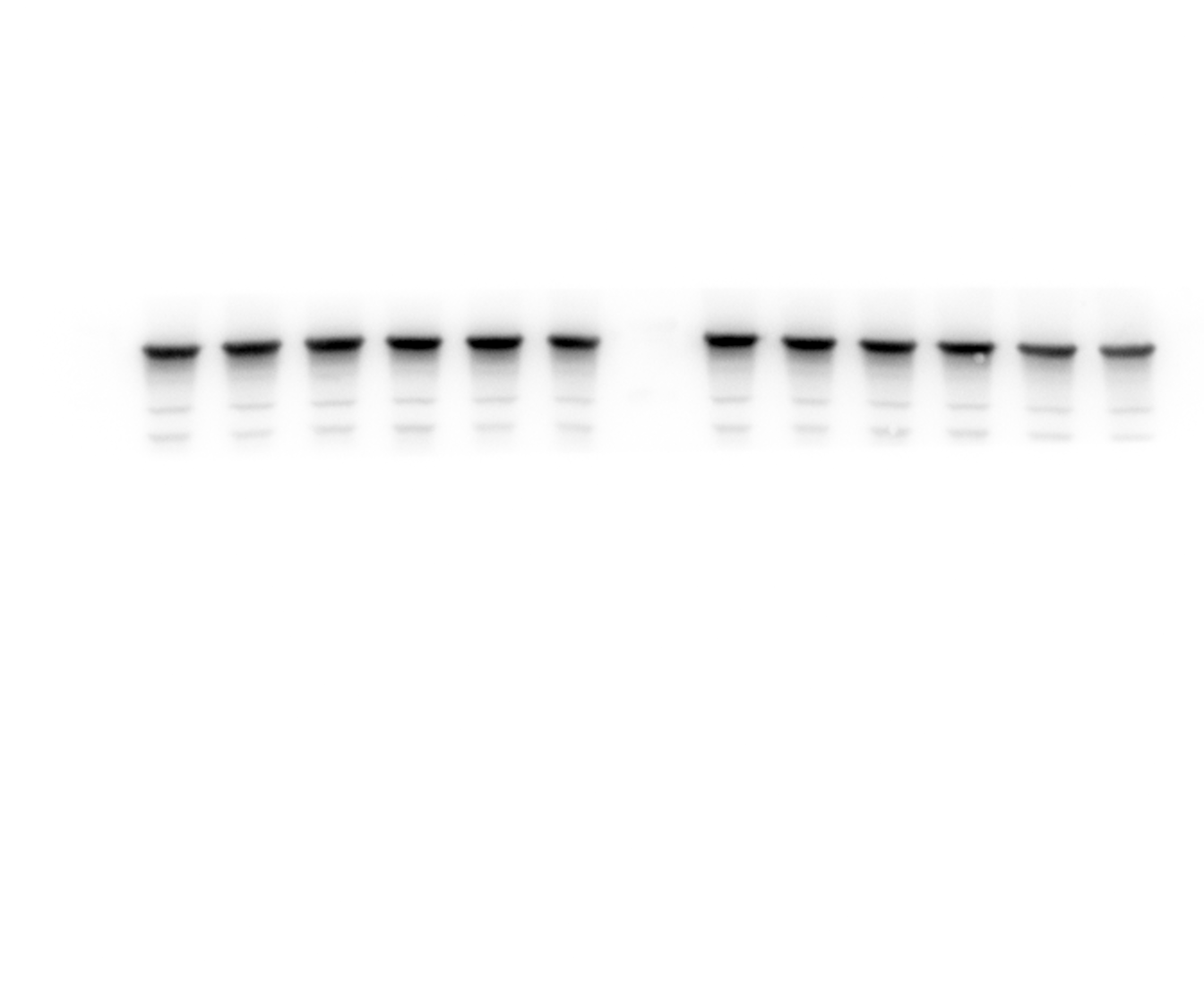

Supplement: Supplementary file 2 — Supporting Information [file ADVS-12-e06225-s001.zip › CHX/4-GAP-H M TQ .Tif]

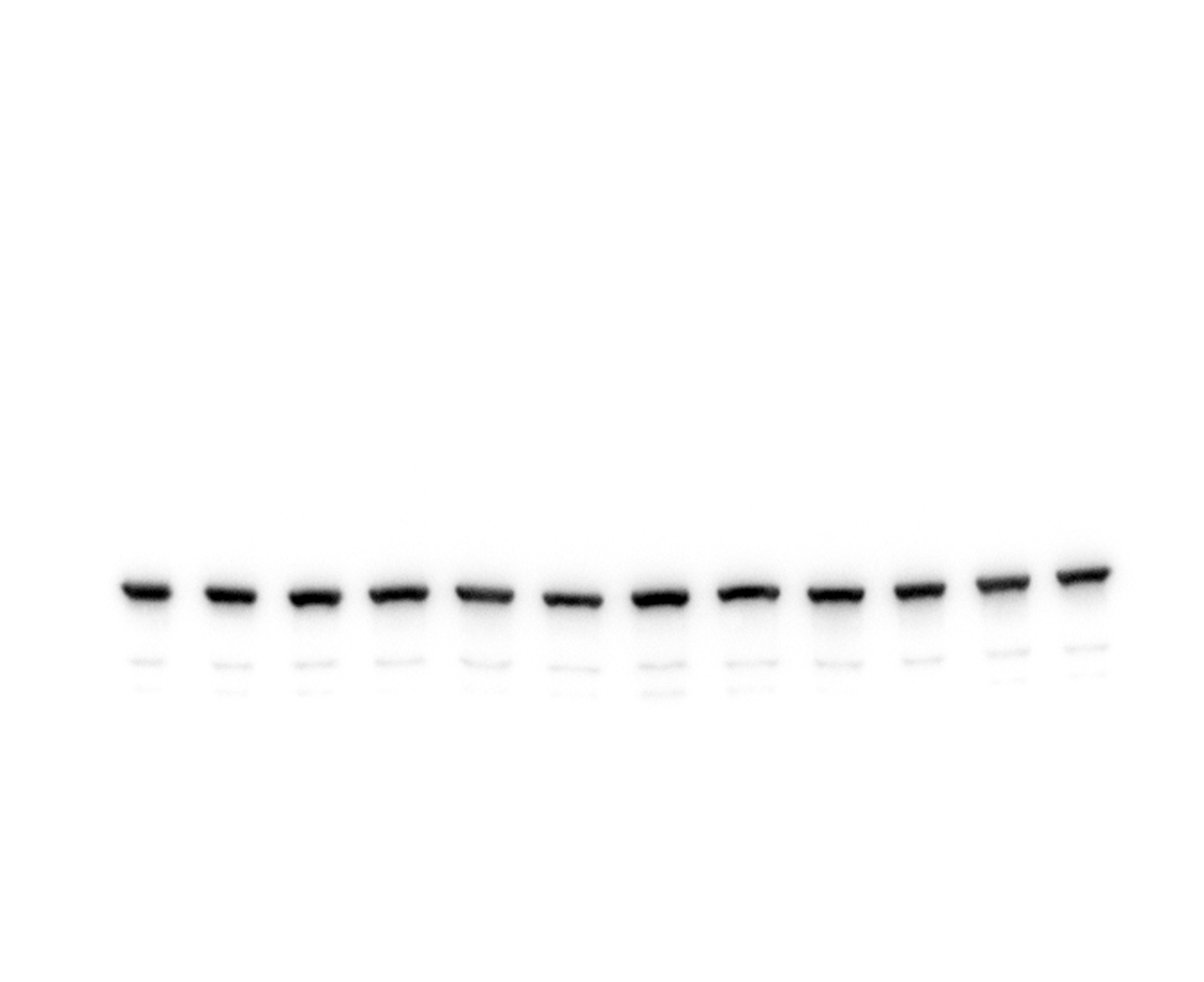

Supplement: Supplementary file 2 — Supporting Information [file ADVS-12-e06225-s001.zip › CHX/4-GAP-H1 & H2-400MS.Tif]

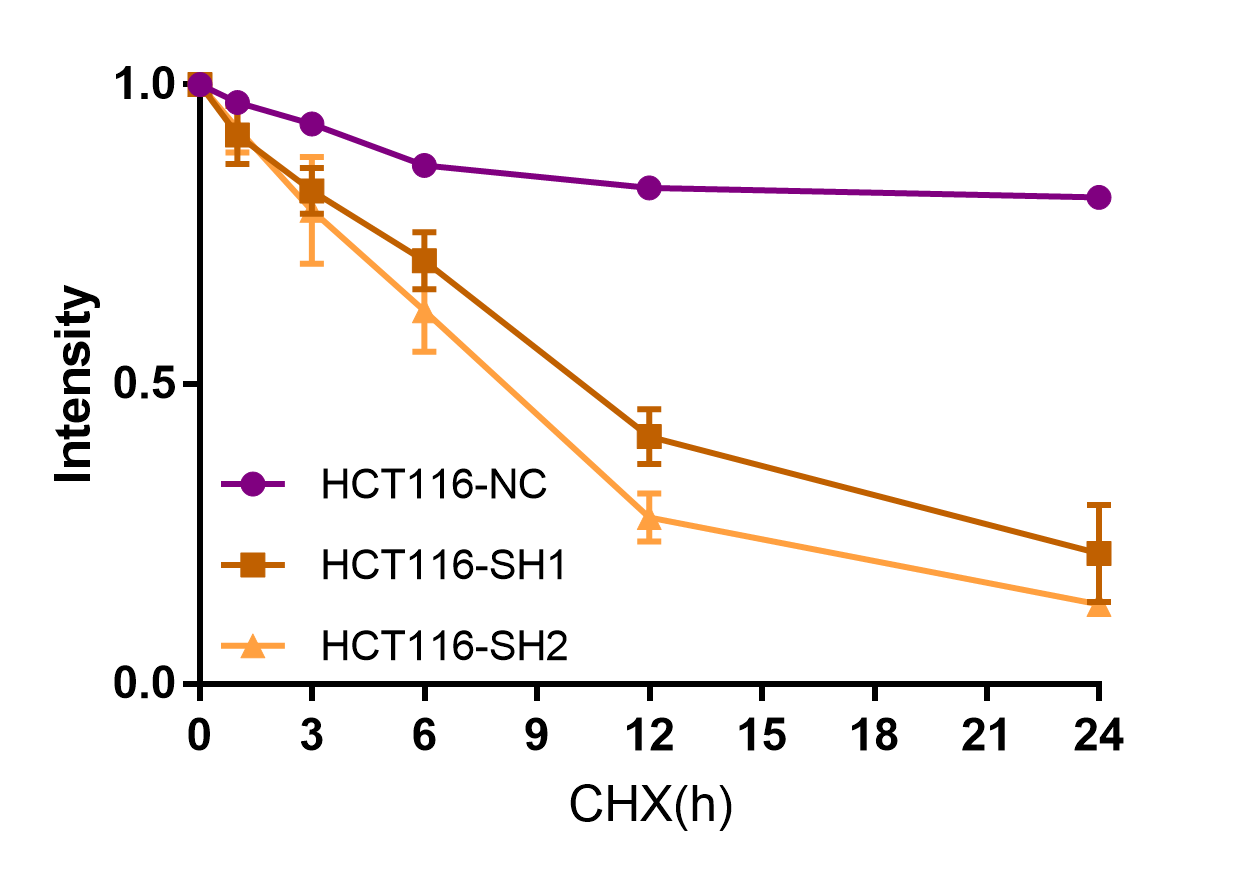

Supplement: Supplementary file 2 — Supporting Information [file ADVS-12-e06225-s001.zip › CHX/chx-116.tif]

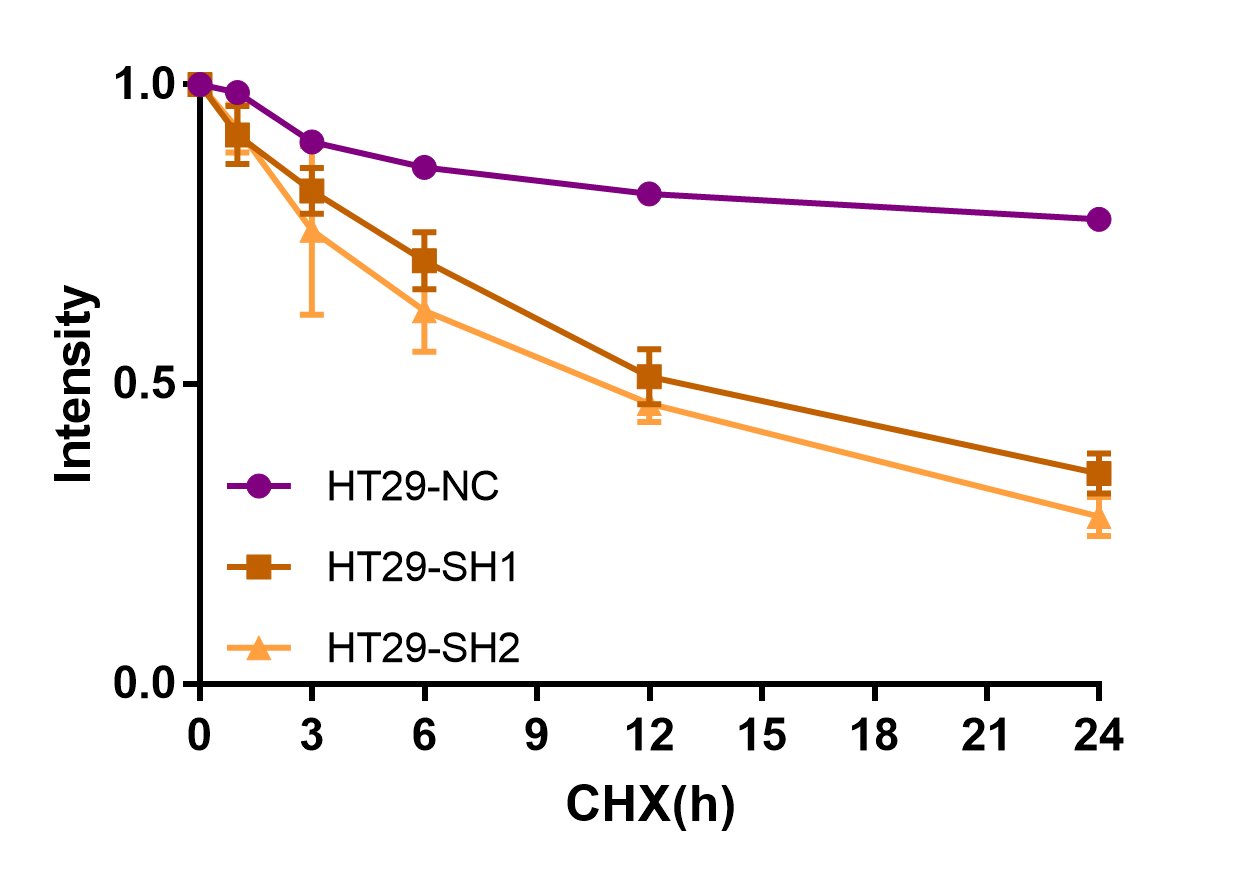

Supplement: Supplementary file 2 — Supporting Information [file ADVS-12-e06225-s001.zip › CHX/chx-29.tif]

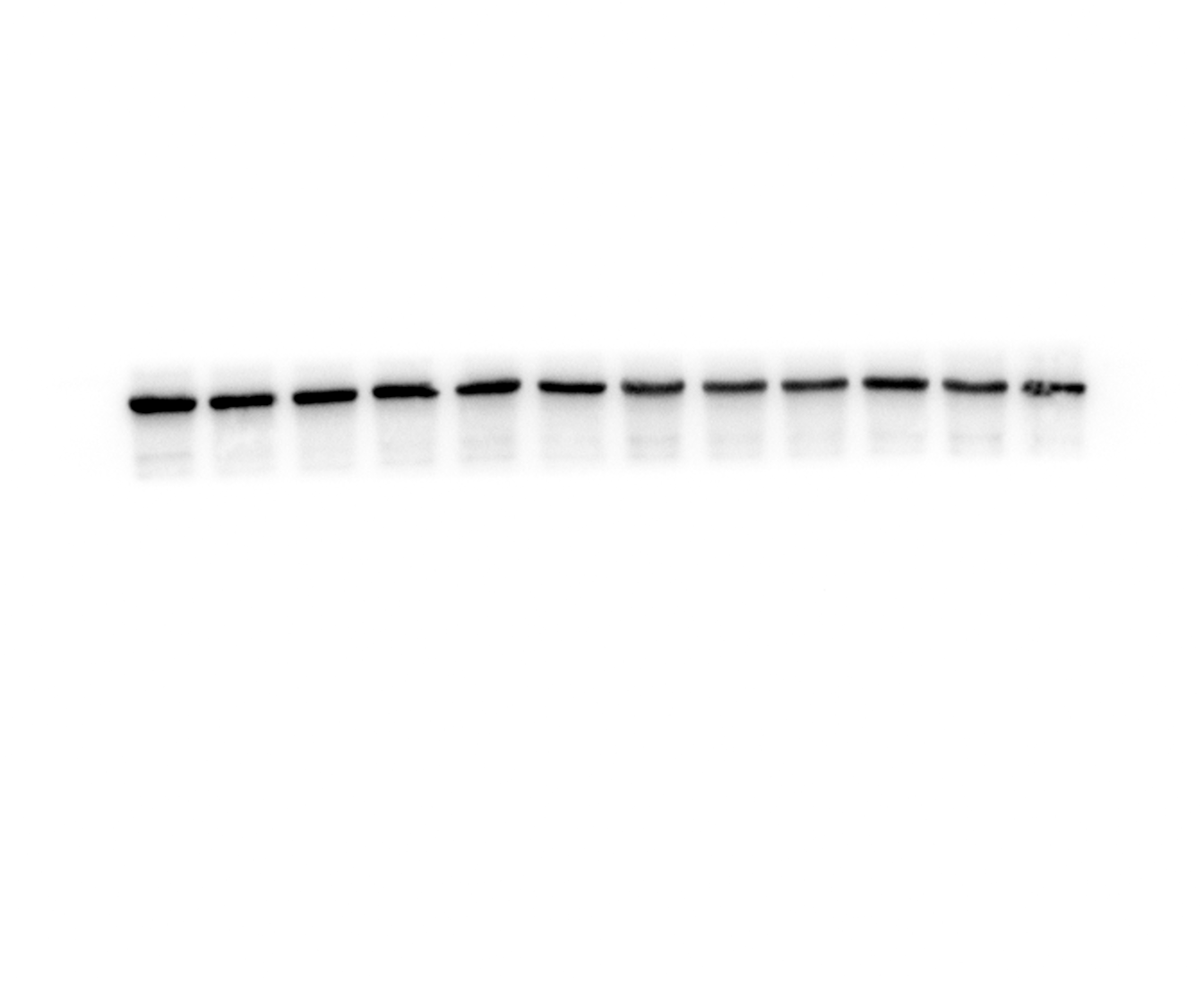

Supplement: Supplementary file 2 — Supporting Information [file ADVS-12-e06225-s001.zip › CHX/GAP-H 0 1 3 6 12 24 M H1 0 1 3 6 12 24-1S-2.Tif]
