# Supplementary figures and images for: ST6GAL1‐Mediated Sialylation Stabilizes PD‐L1 and Drives Immunosuppressive Tumor Microenvironment in Colorectal Cancer (part 1 of 2)
Source: Adv Sci (Weinh). 2025 Aug 22;12(42):e06225. doi: 10.1002/advs.202406225 (PMC12622430; doi:10.1002/advs.202406225)

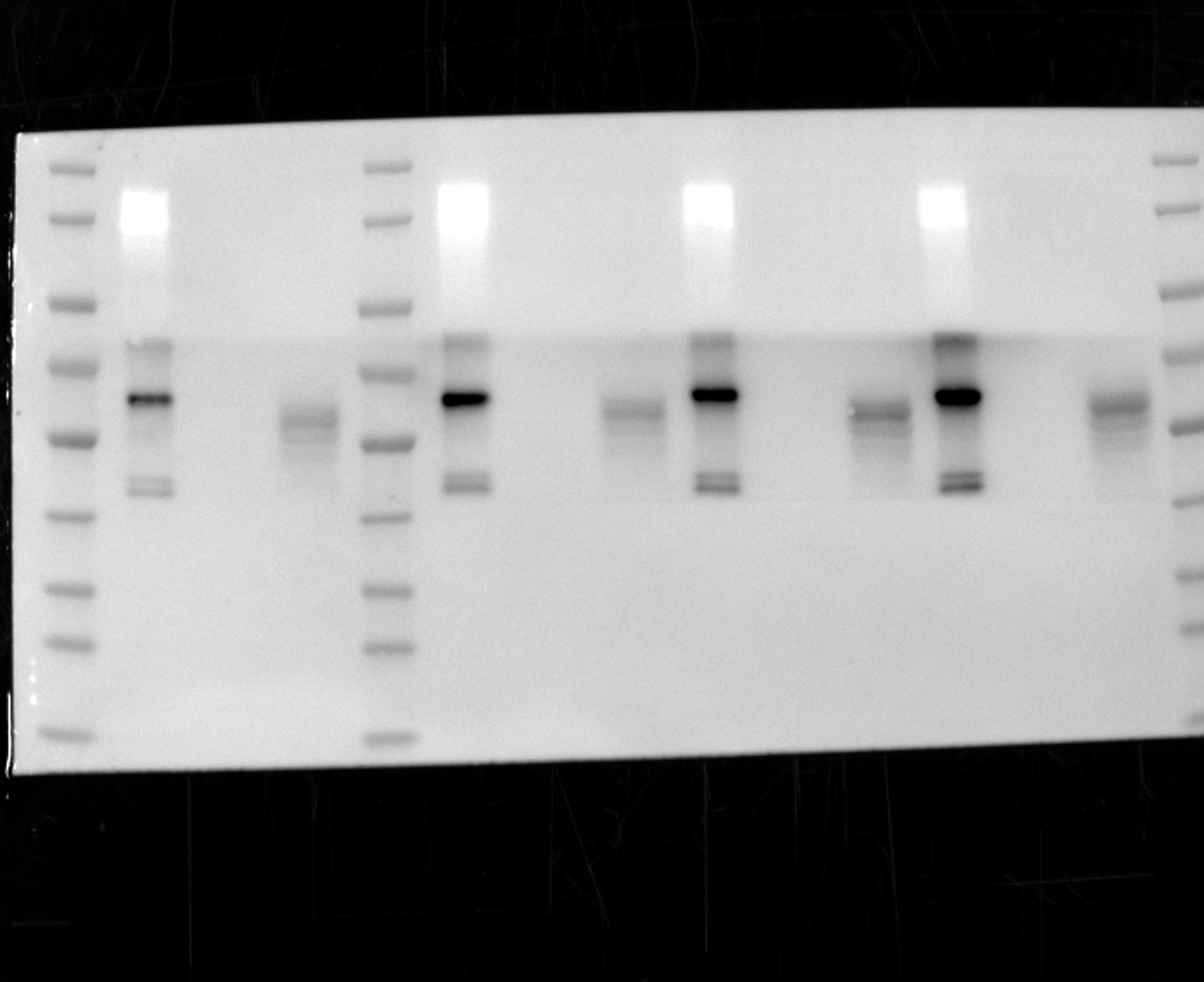

Supplement: Supplementary file 2 — Supporting Information [file ADVS-12-e06225-s001.zip › CO-IP/CD75-CD75液-HIP HIgG HIN-1.Tif]

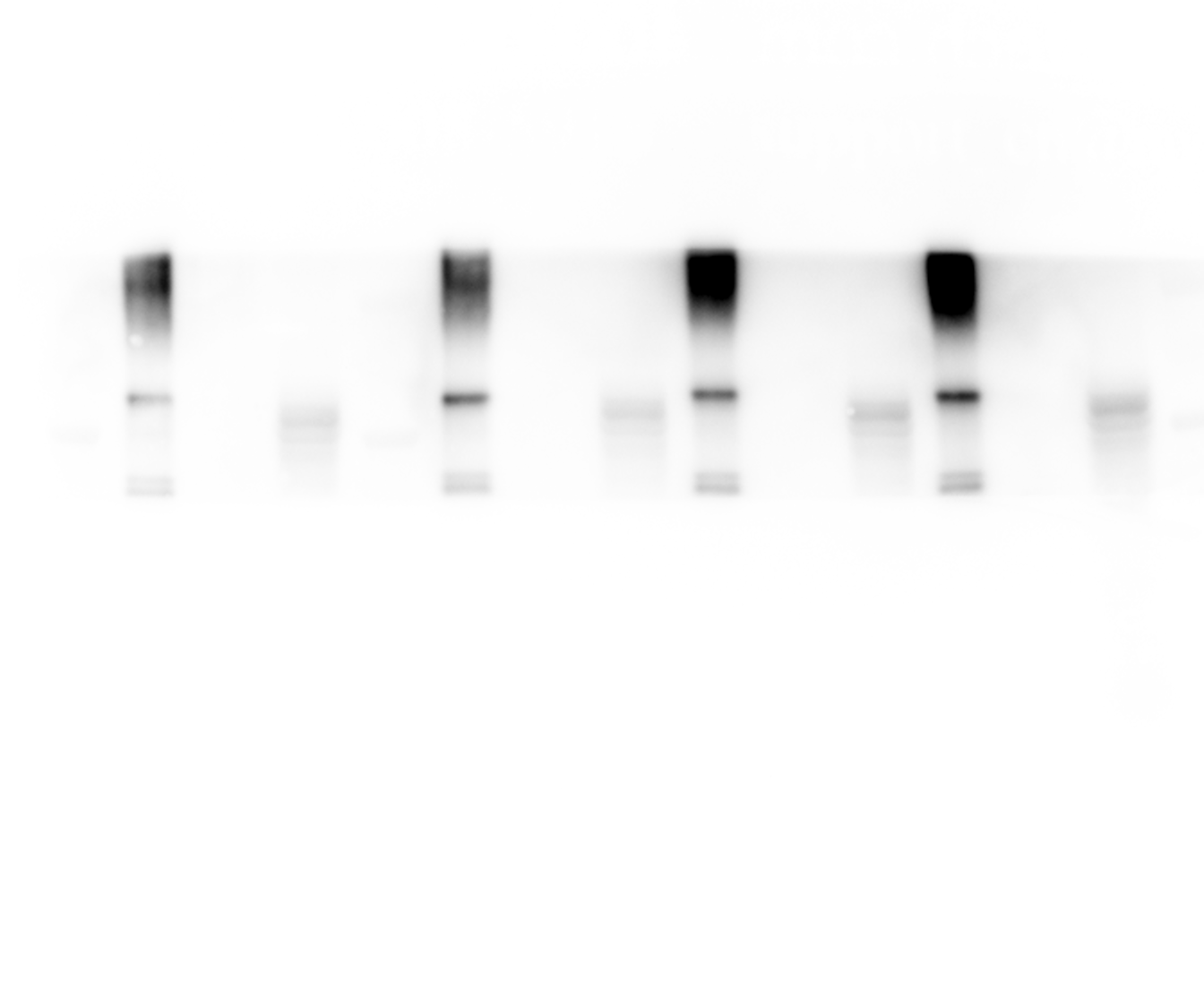

Supplement: Supplementary file 2 — Supporting Information [file ADVS-12-e06225-s001.zip › CO-IP/CD75-CD75液-HIP HIgG HIN-10.Tif]

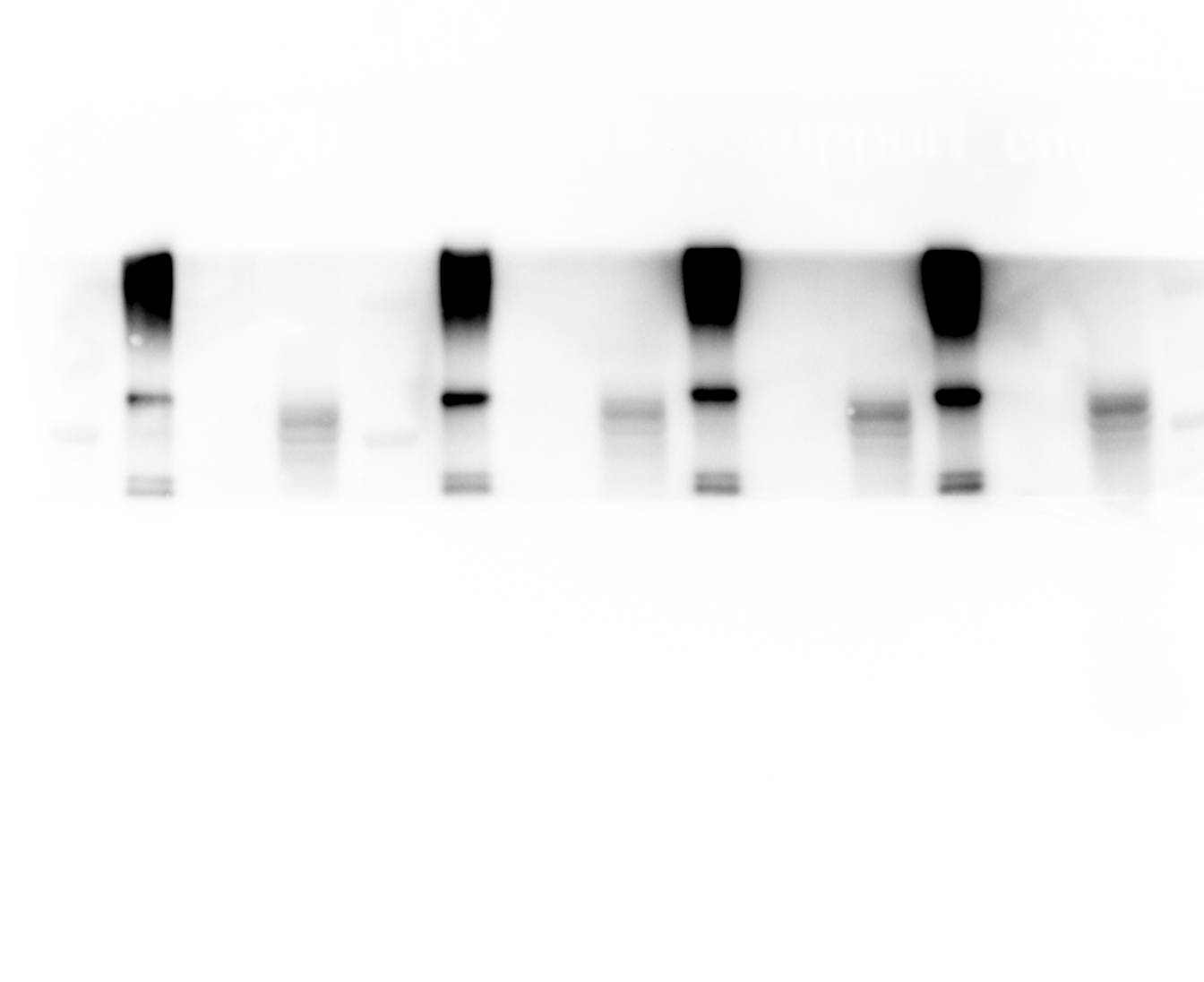

Supplement: Supplementary file 2 — Supporting Information [file ADVS-12-e06225-s001.zip › CO-IP/CD75-CD75液-HIP HIgG HIN-11.Tif]

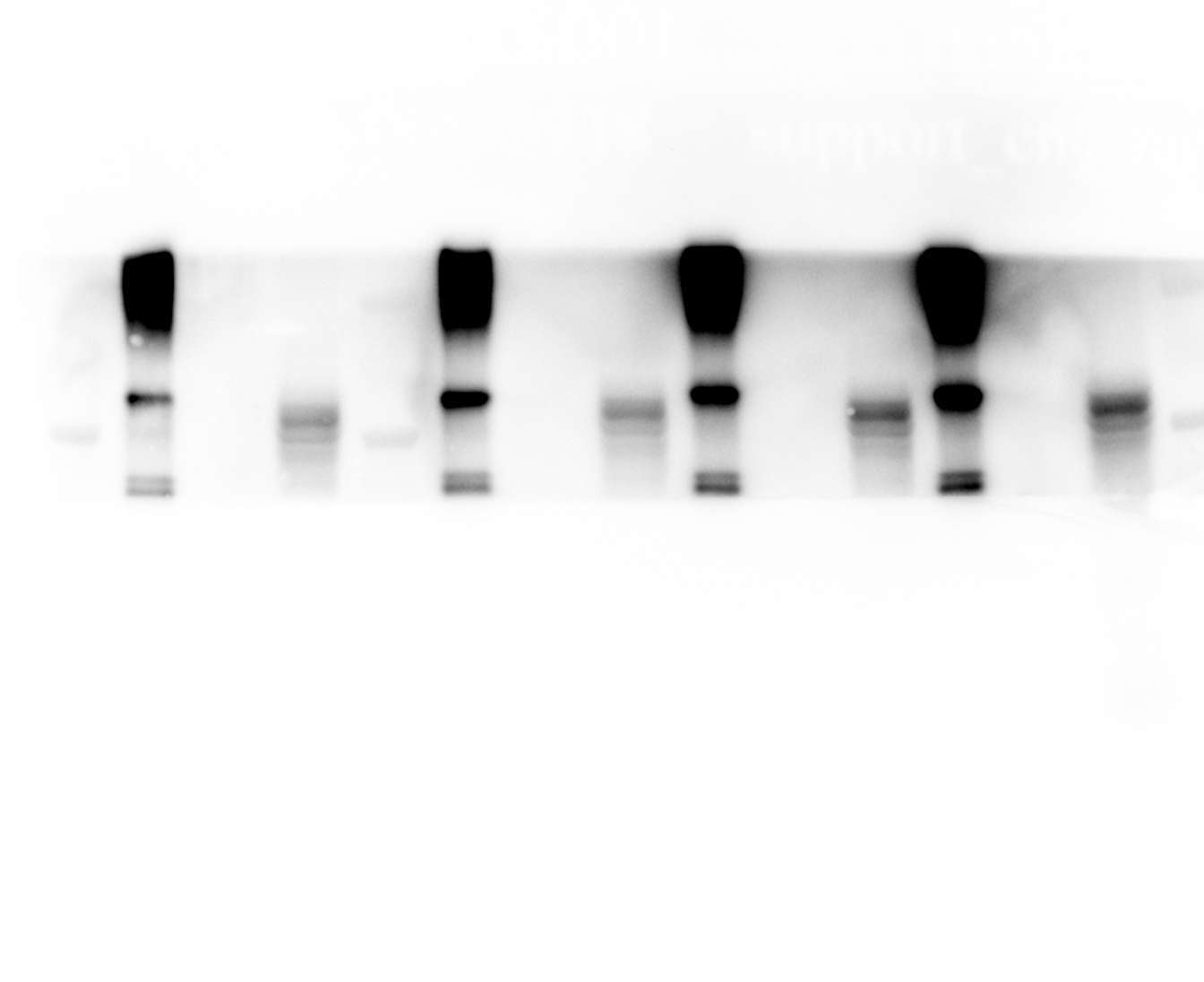

Supplement: Supplementary file 2 — Supporting Information [file ADVS-12-e06225-s001.zip › CO-IP/CD75-CD75液-HIP HIgG HIN-12.Tif]

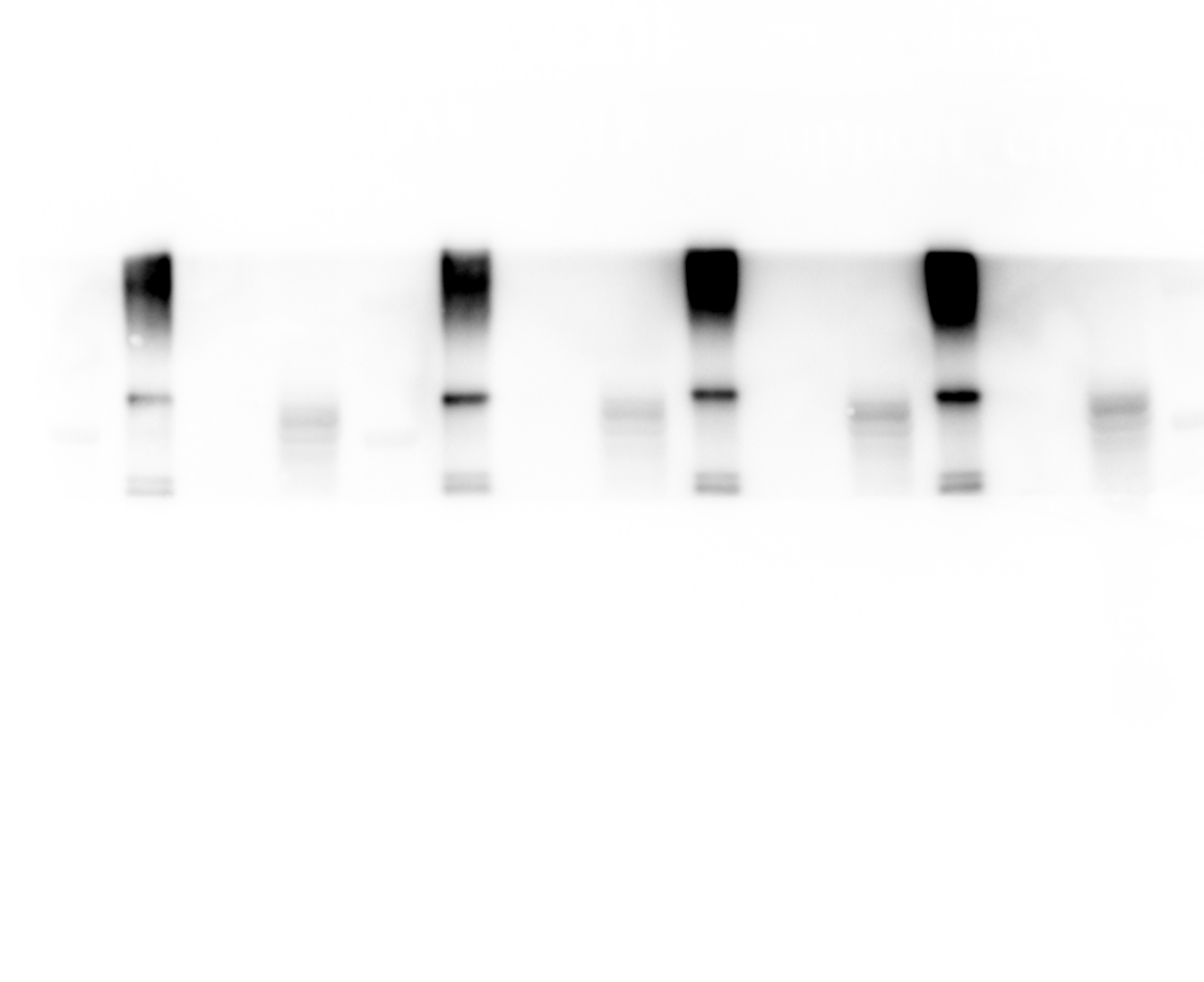

Supplement: Supplementary file 2 — Supporting Information [file ADVS-12-e06225-s001.zip › CO-IP/CD75-CD75液-HIP HIgG HIN-13.Tif]

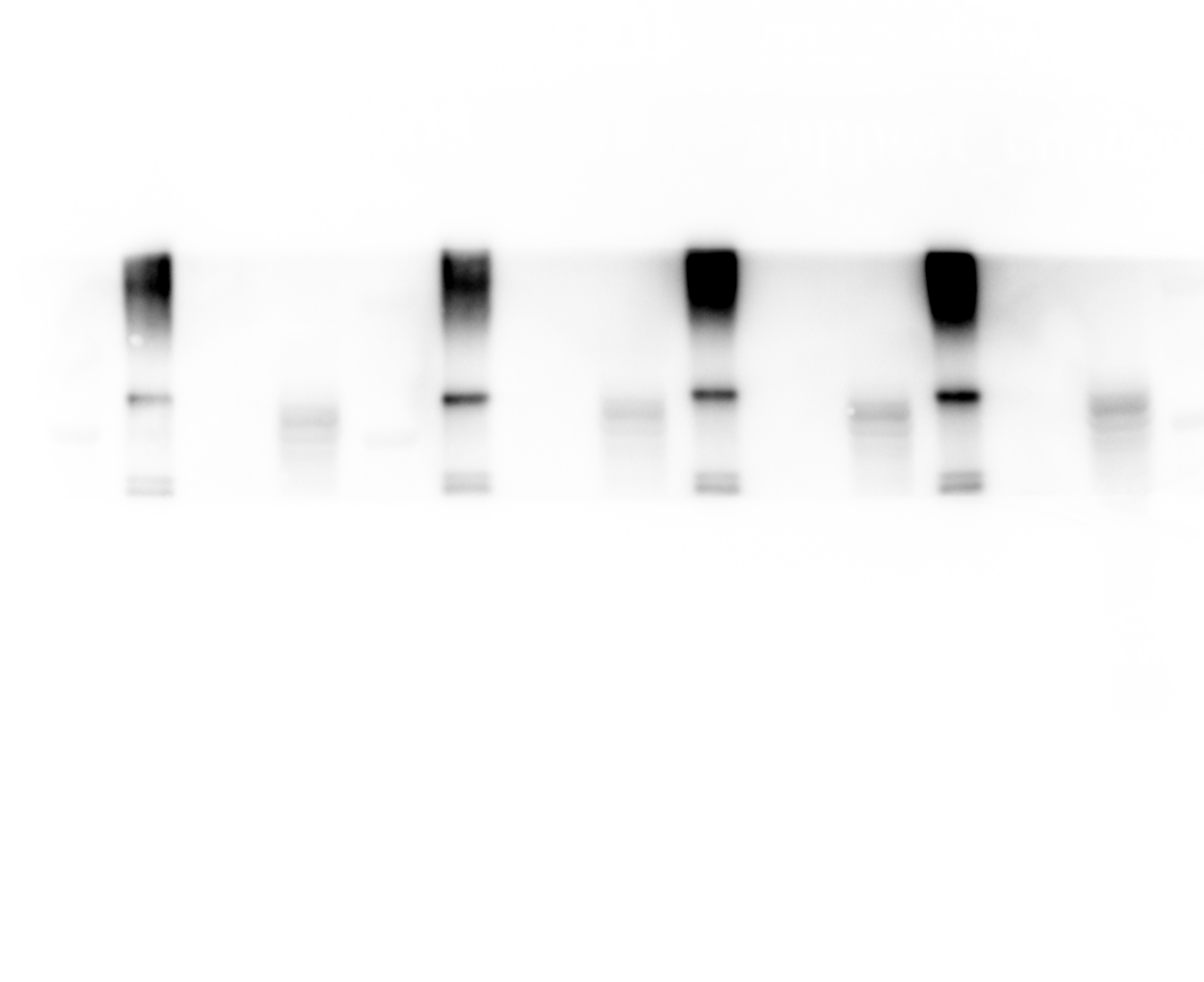

Supplement: Supplementary file 2 — Supporting Information [file ADVS-12-e06225-s001.zip › CO-IP/CD75-CD75液-HIP HIgG HIN-14.Tif]

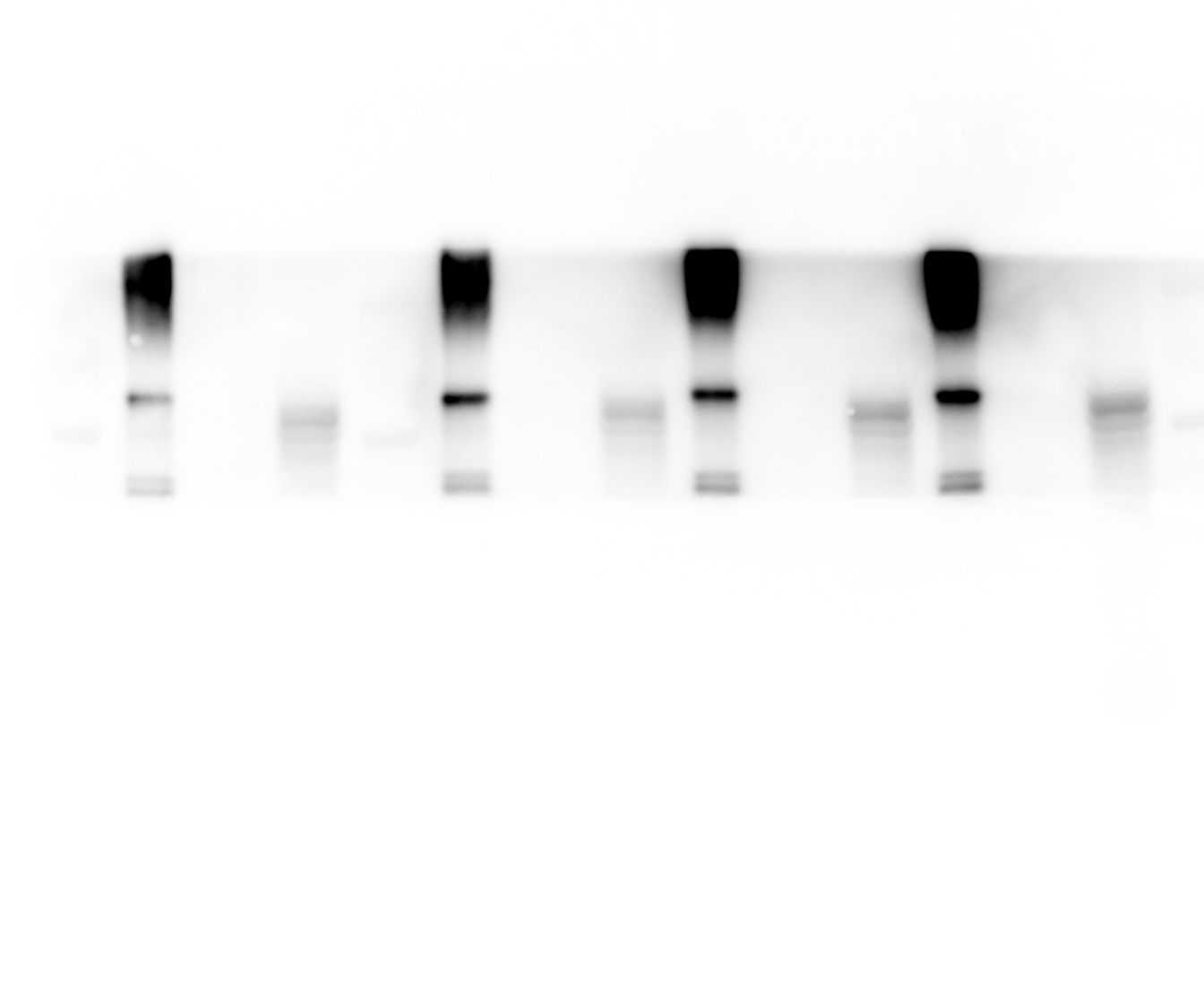

Supplement: Supplementary file 2 — Supporting Information [file ADVS-12-e06225-s001.zip › CO-IP/CD75-CD75液-HIP HIgG HIN-15.Tif]

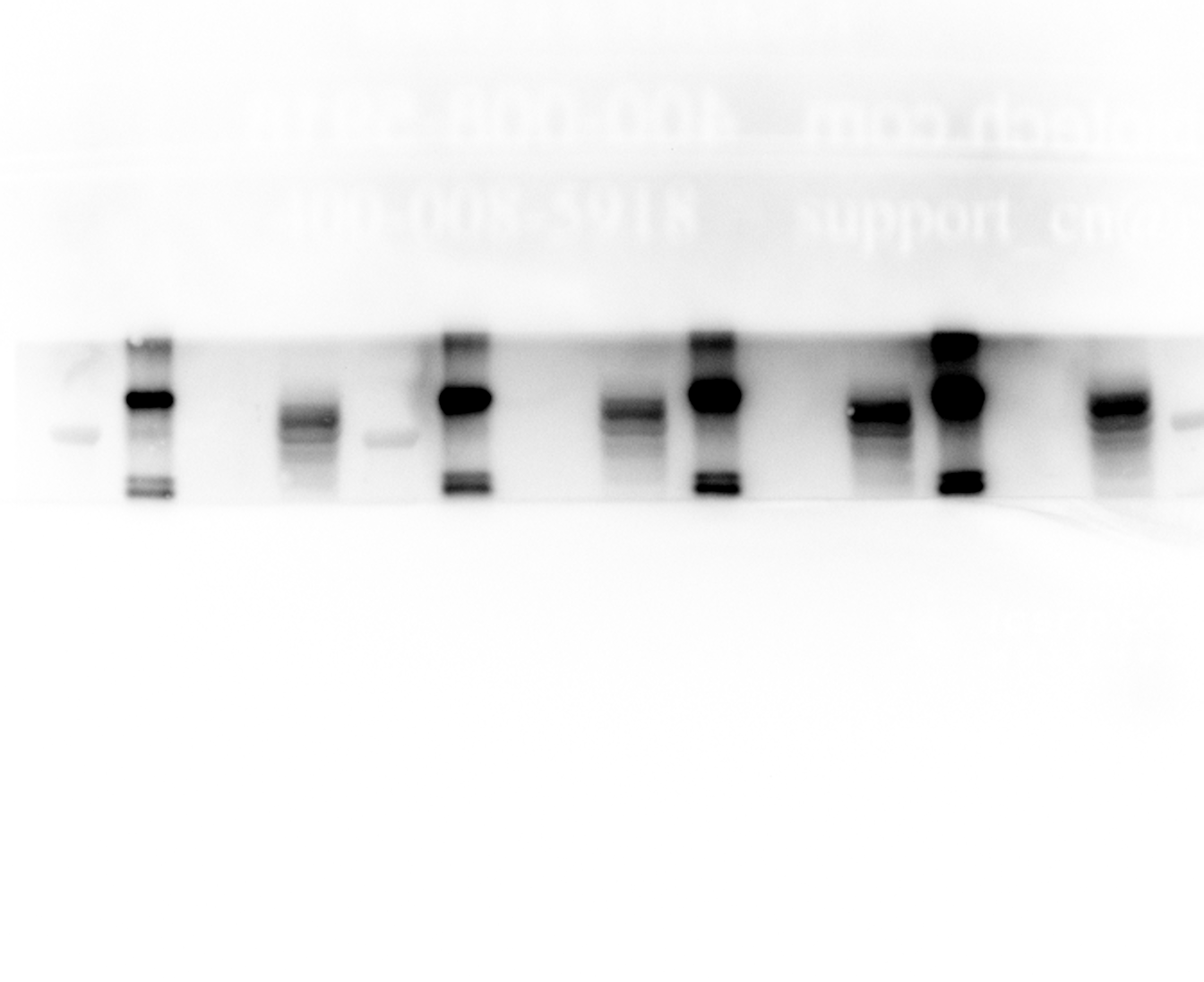

Supplement: Supplementary file 2 — Supporting Information [file ADVS-12-e06225-s001.zip › CO-IP/CD75-CD75液-HIP HIgG HIN-2.Tif]

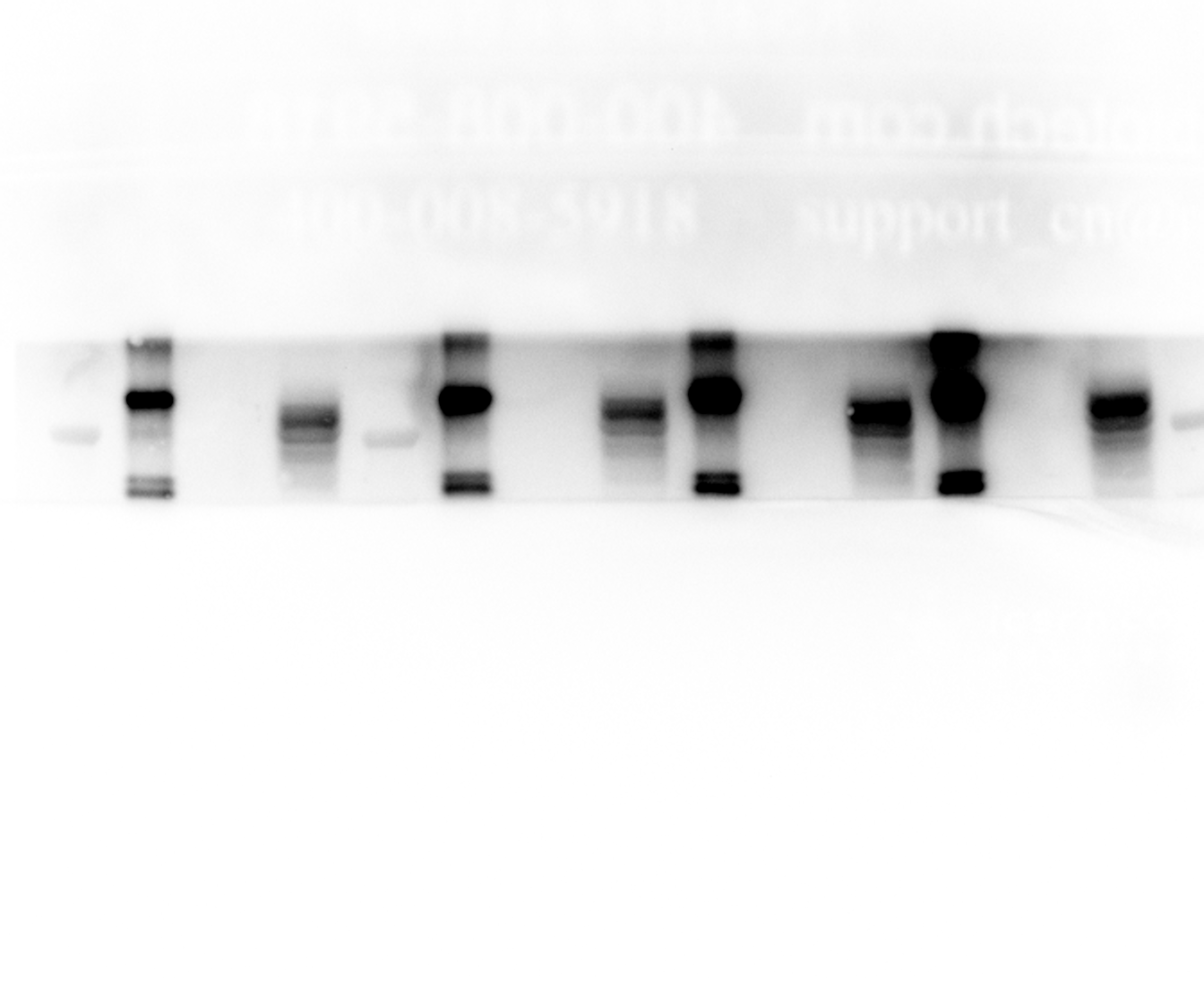

Supplement: Supplementary file 2 — Supporting Information [file ADVS-12-e06225-s001.zip › CO-IP/CD75-CD75液-HIP HIgG HIN-3.Tif]

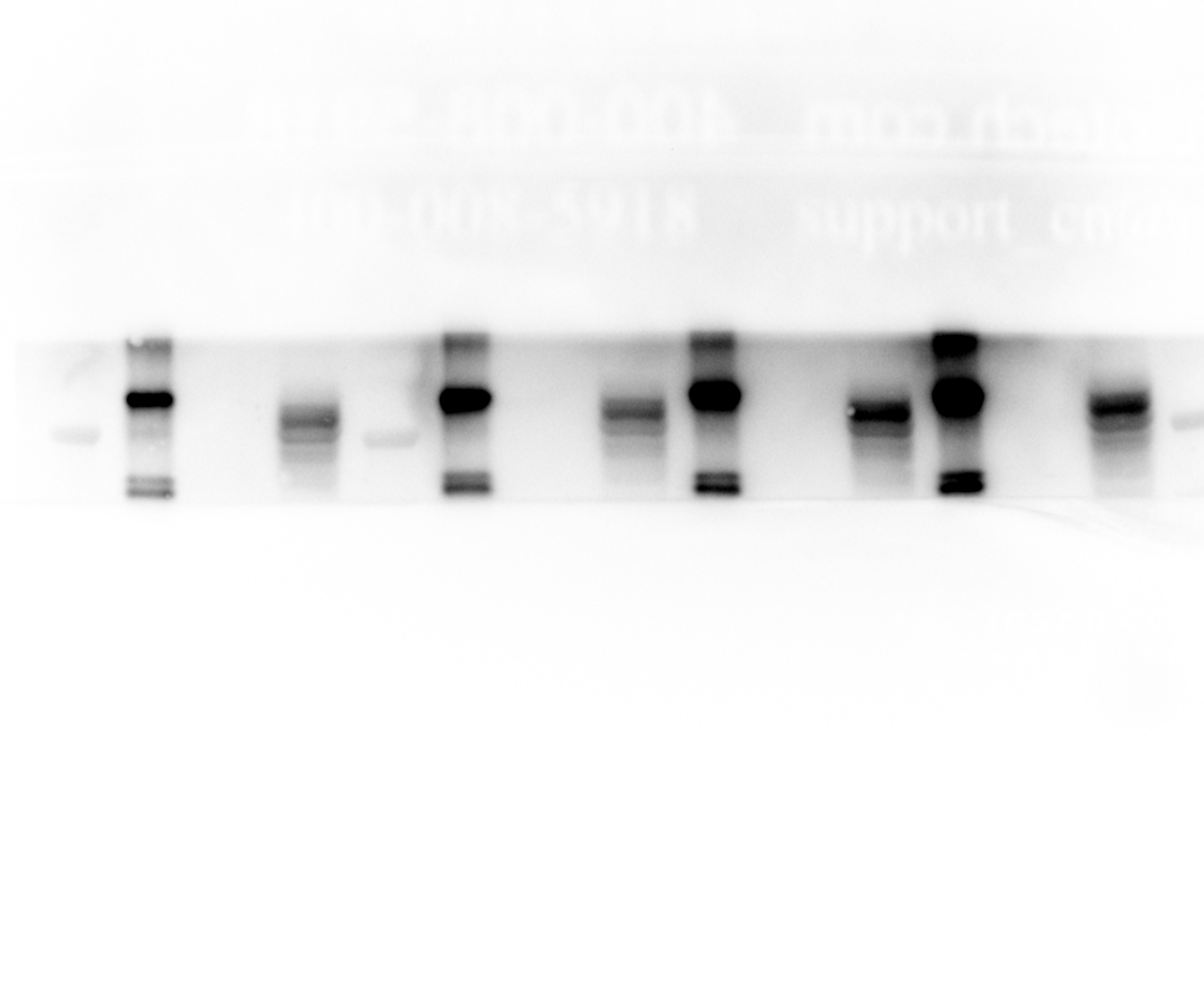

Supplement: Supplementary file 2 — Supporting Information [file ADVS-12-e06225-s001.zip › CO-IP/CD75-CD75液-HIP HIgG HIN-4.Tif]

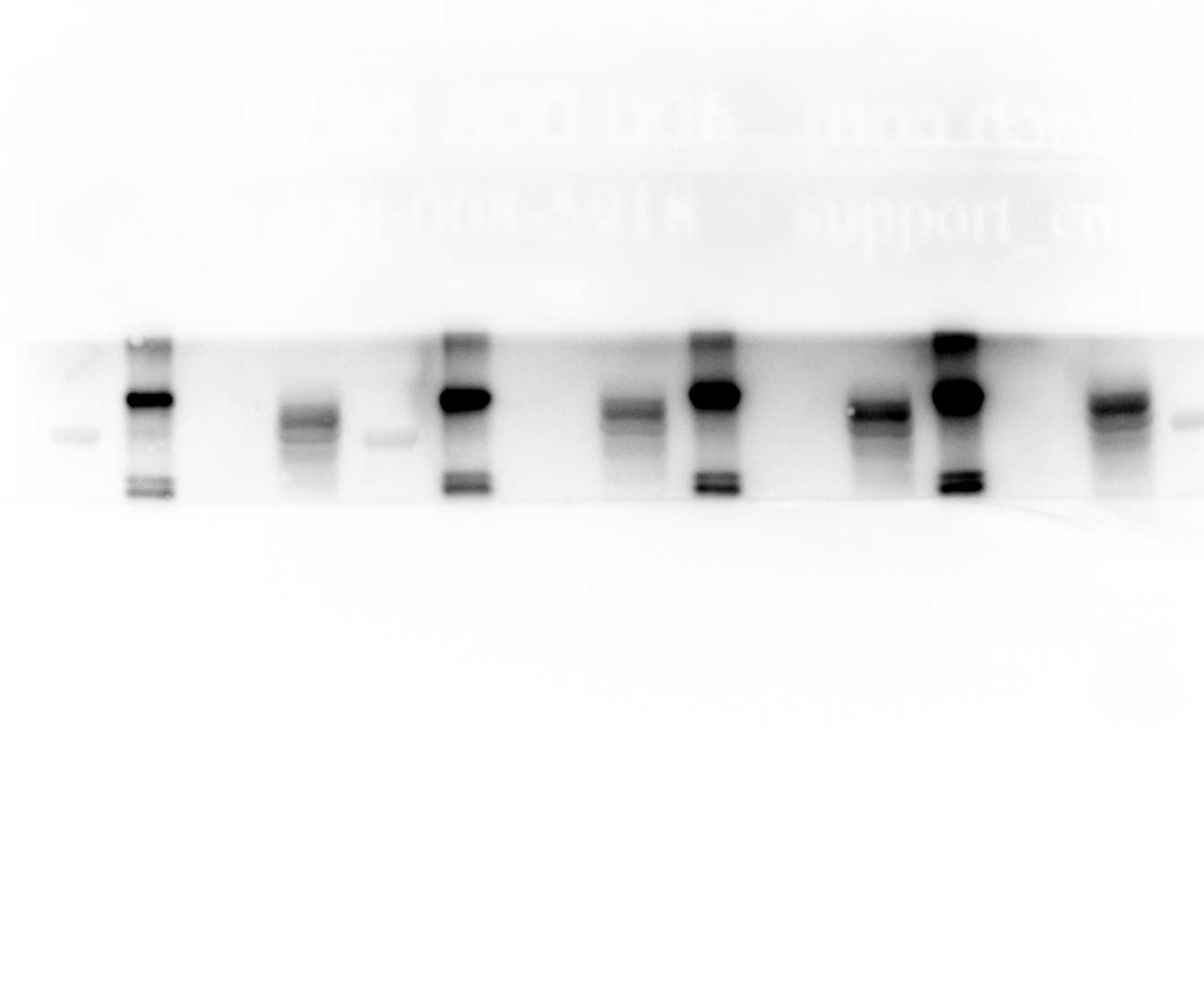

Supplement: Supplementary file 2 — Supporting Information [file ADVS-12-e06225-s001.zip › CO-IP/CD75-CD75液-HIP HIgG HIN-5.Tif]

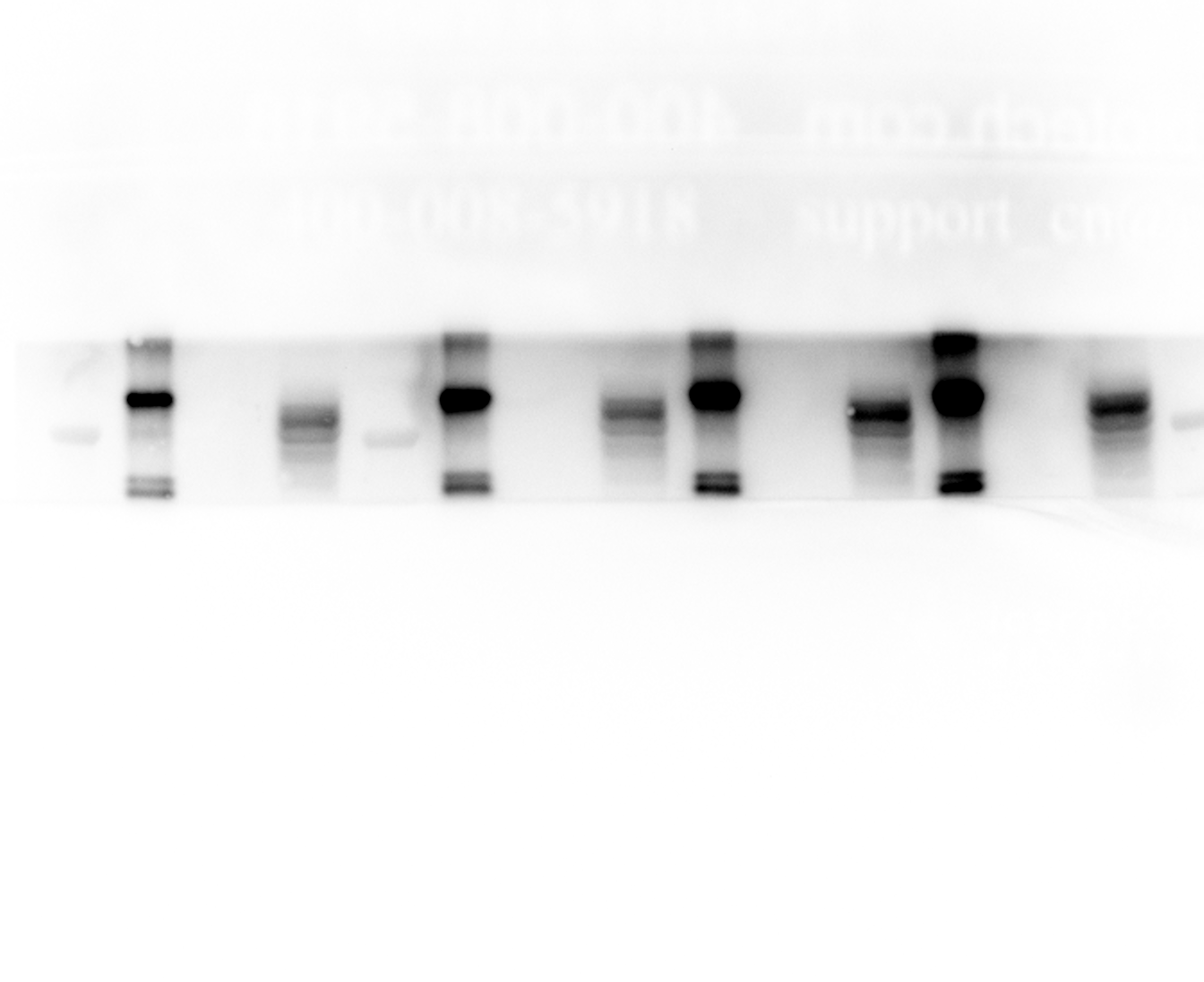

Supplement: Supplementary file 2 — Supporting Information [file ADVS-12-e06225-s001.zip › CO-IP/CD75-CD75液-HIP HIgG HIN-6.Tif]

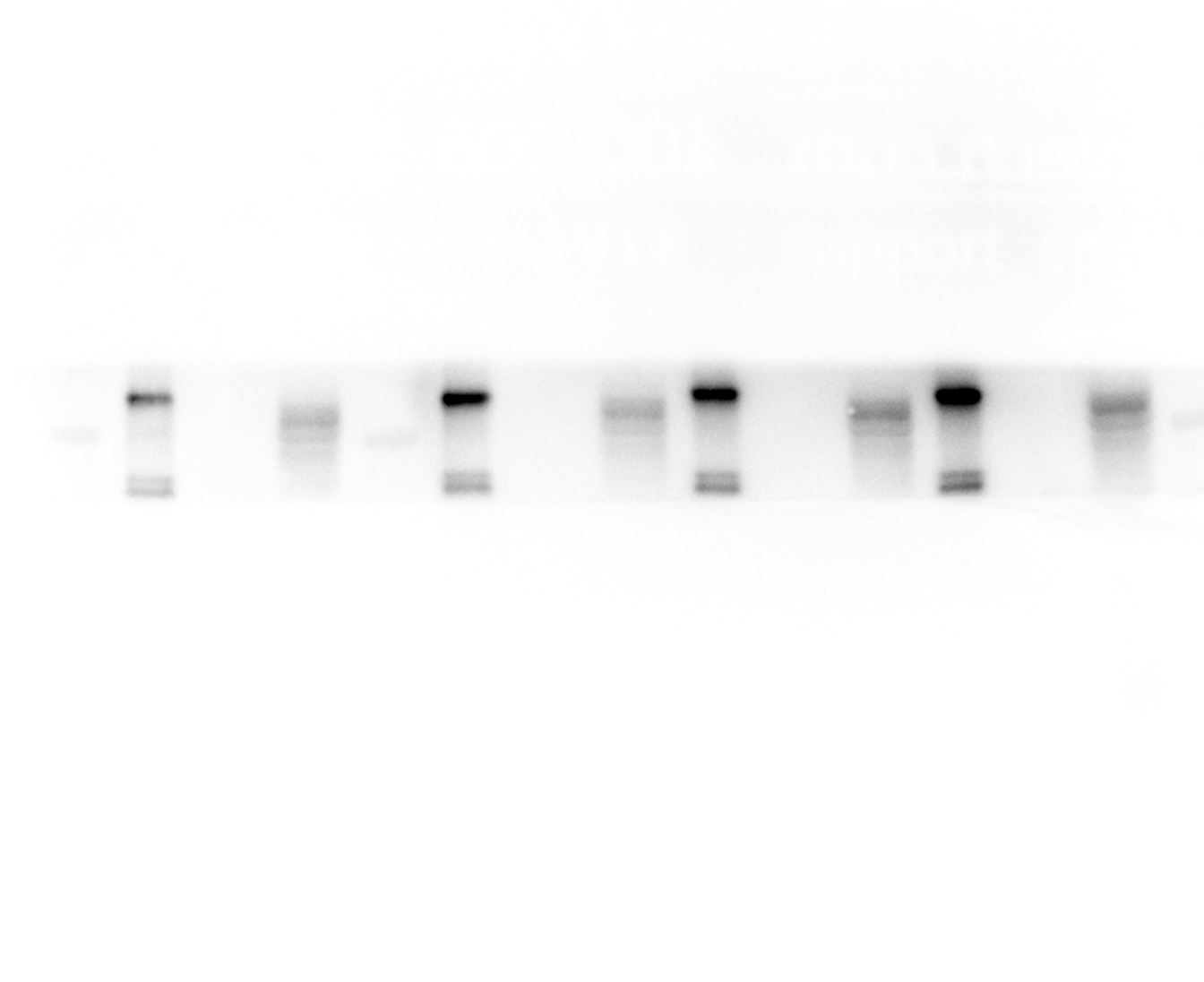

Supplement: Supplementary file 2 — Supporting Information [file ADVS-12-e06225-s001.zip › CO-IP/CD75-CD75液-HIP HIgG HIN-7.Tif]

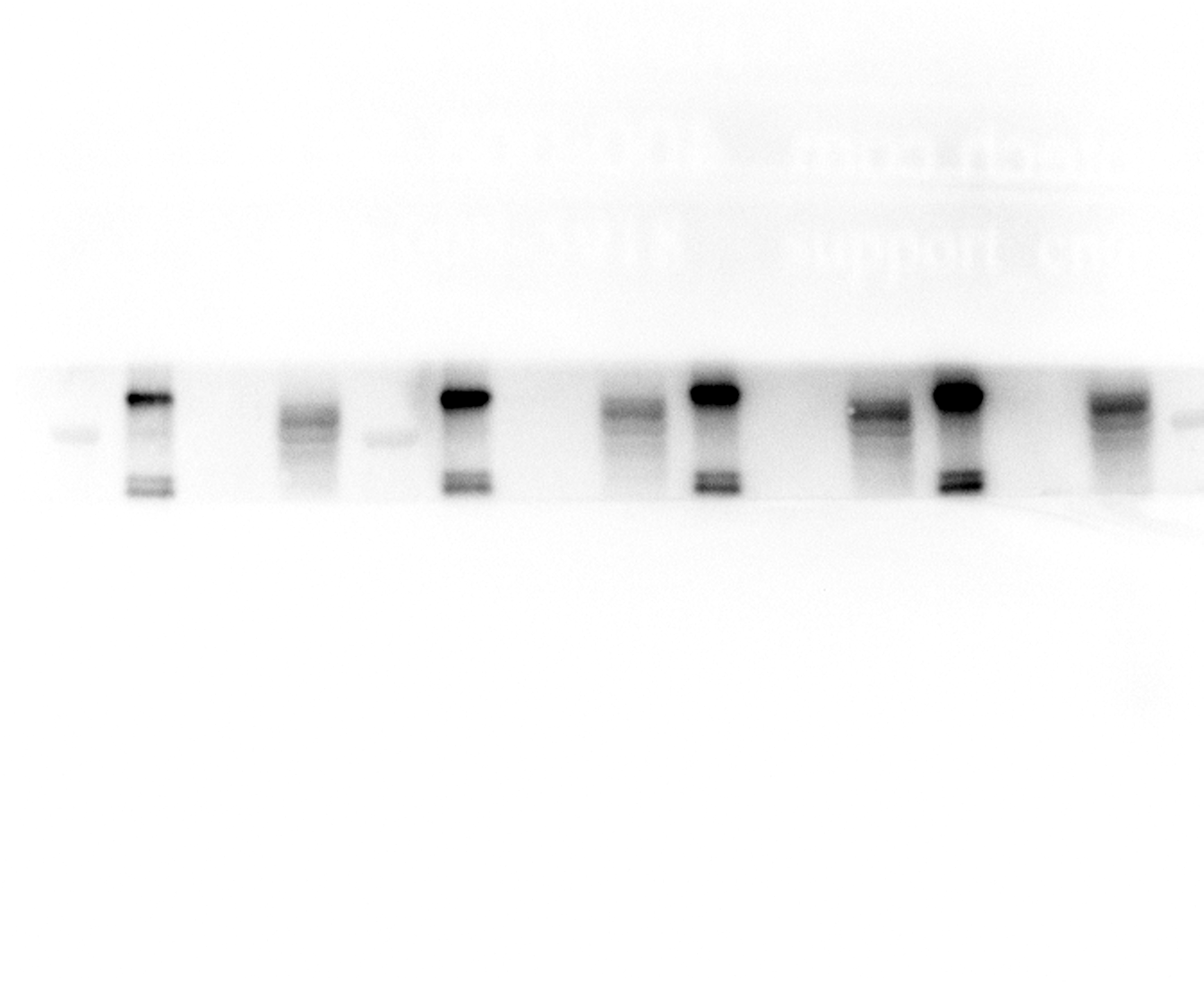

Supplement: Supplementary file 2 — Supporting Information [file ADVS-12-e06225-s001.zip › CO-IP/CD75-CD75液-HIP HIgG HIN-8.Tif]

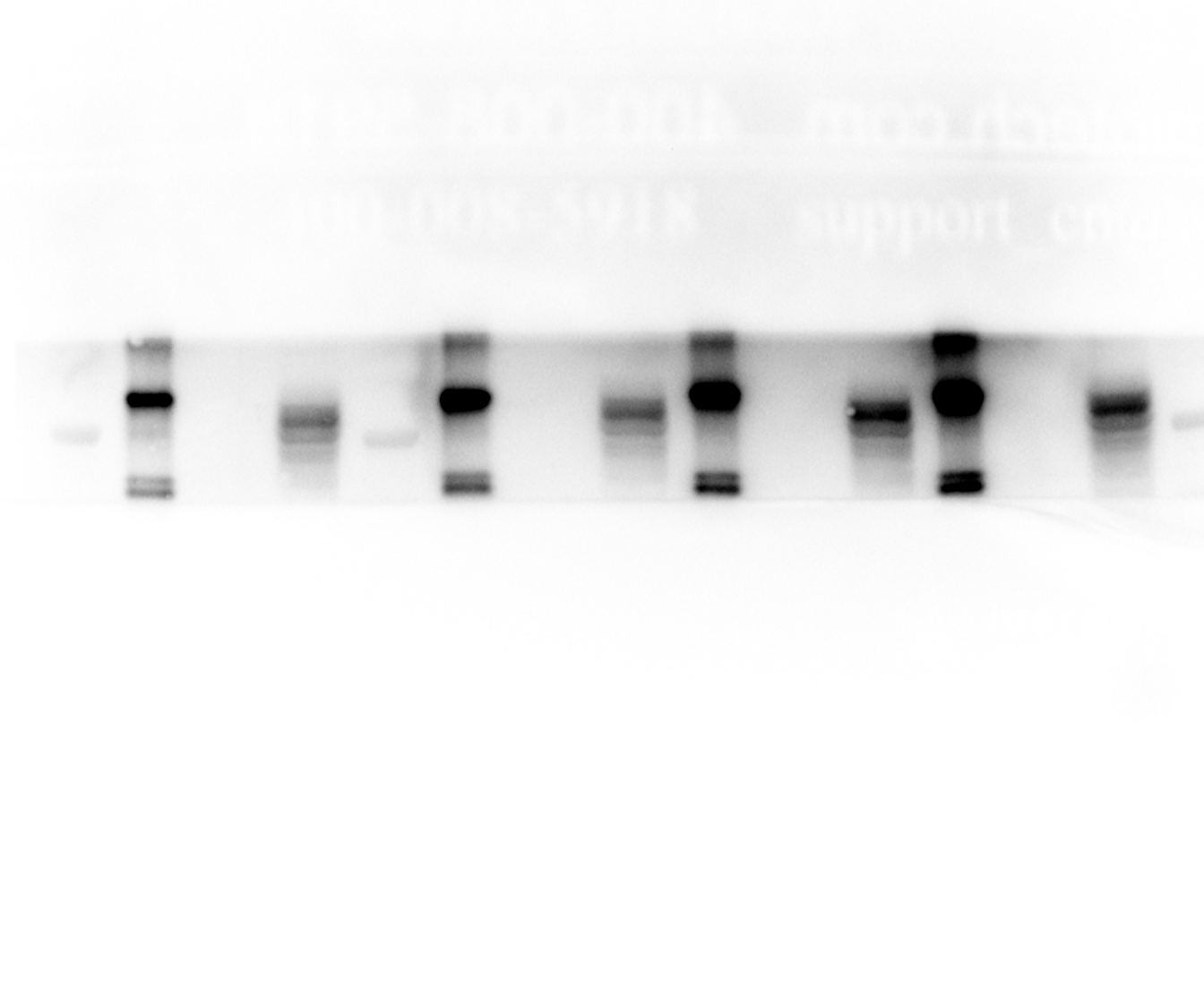

Supplement: Supplementary file 2 — Supporting Information [file ADVS-12-e06225-s001.zip › CO-IP/CD75-CD75液-HIP HIgG HIN-9.Tif]

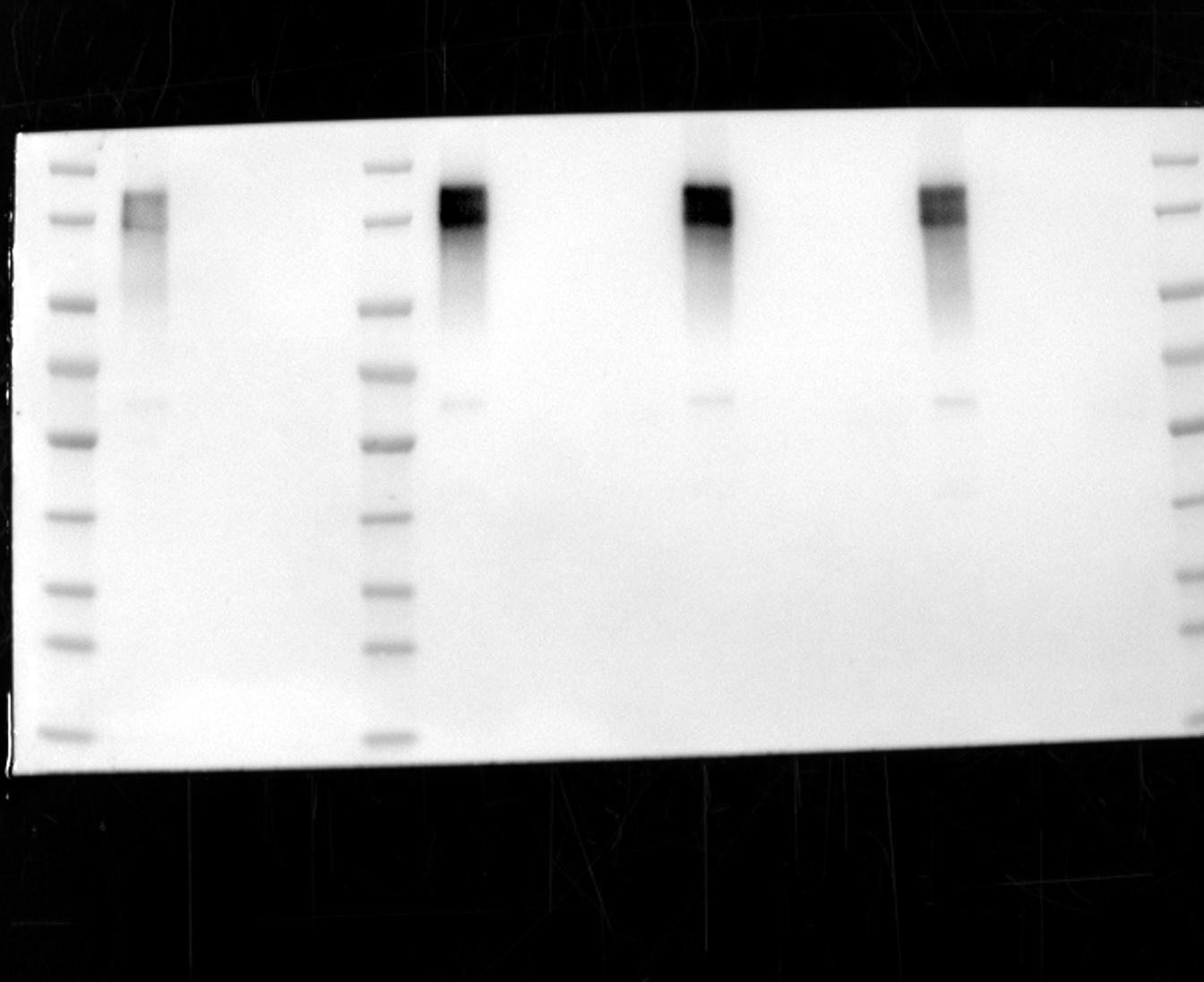

Supplement: Supplementary file 2 — Supporting Information [file ADVS-12-e06225-s001.zip › CO-IP/CD75-CD75液-HIP HIgG HIN-M.Tif]

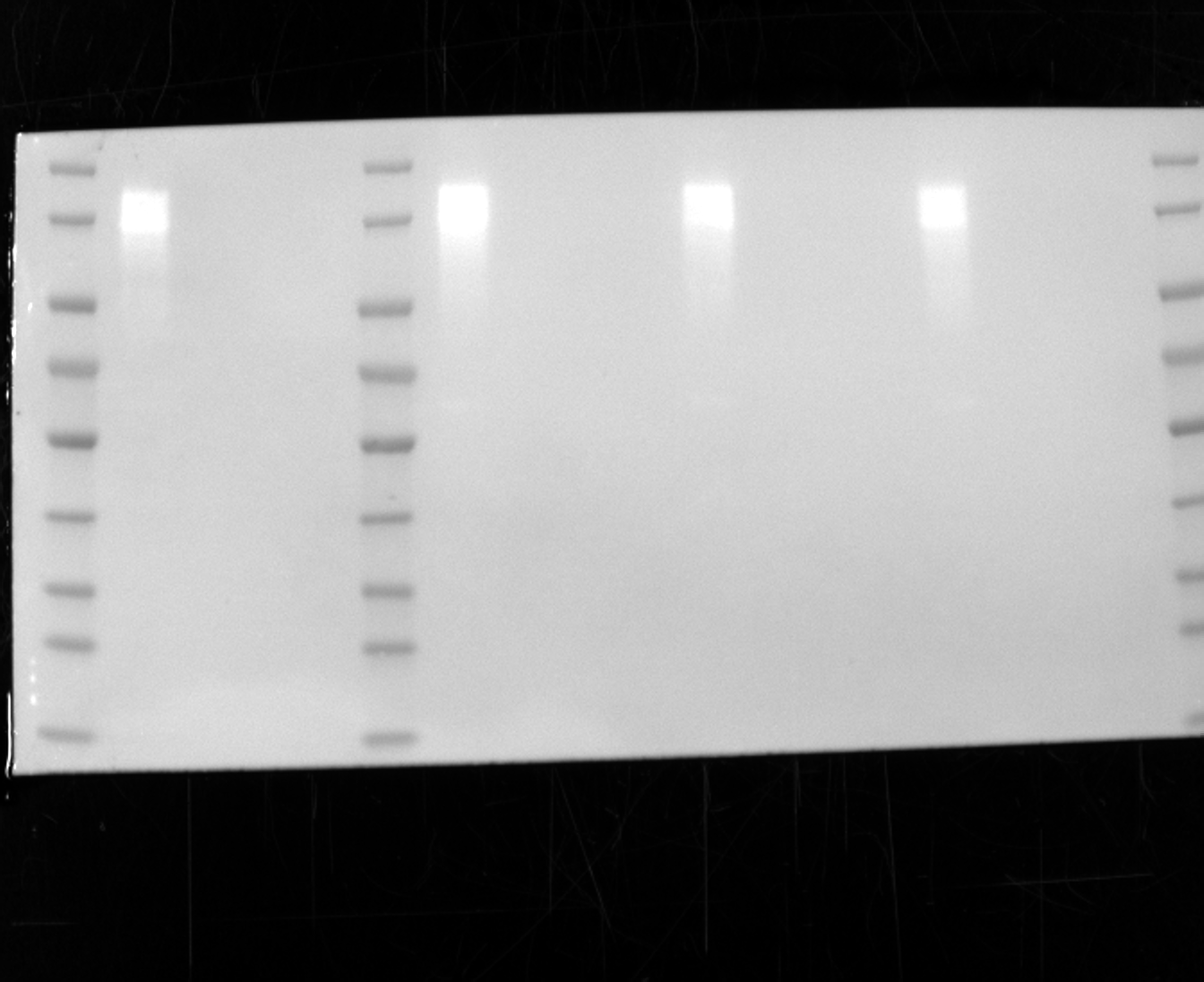

Supplement: Supplementary file 2 — Supporting Information [file ADVS-12-e06225-s001.zip › CO-IP/CD75-CD75液-HIP HIgG HIN-W.Tif]

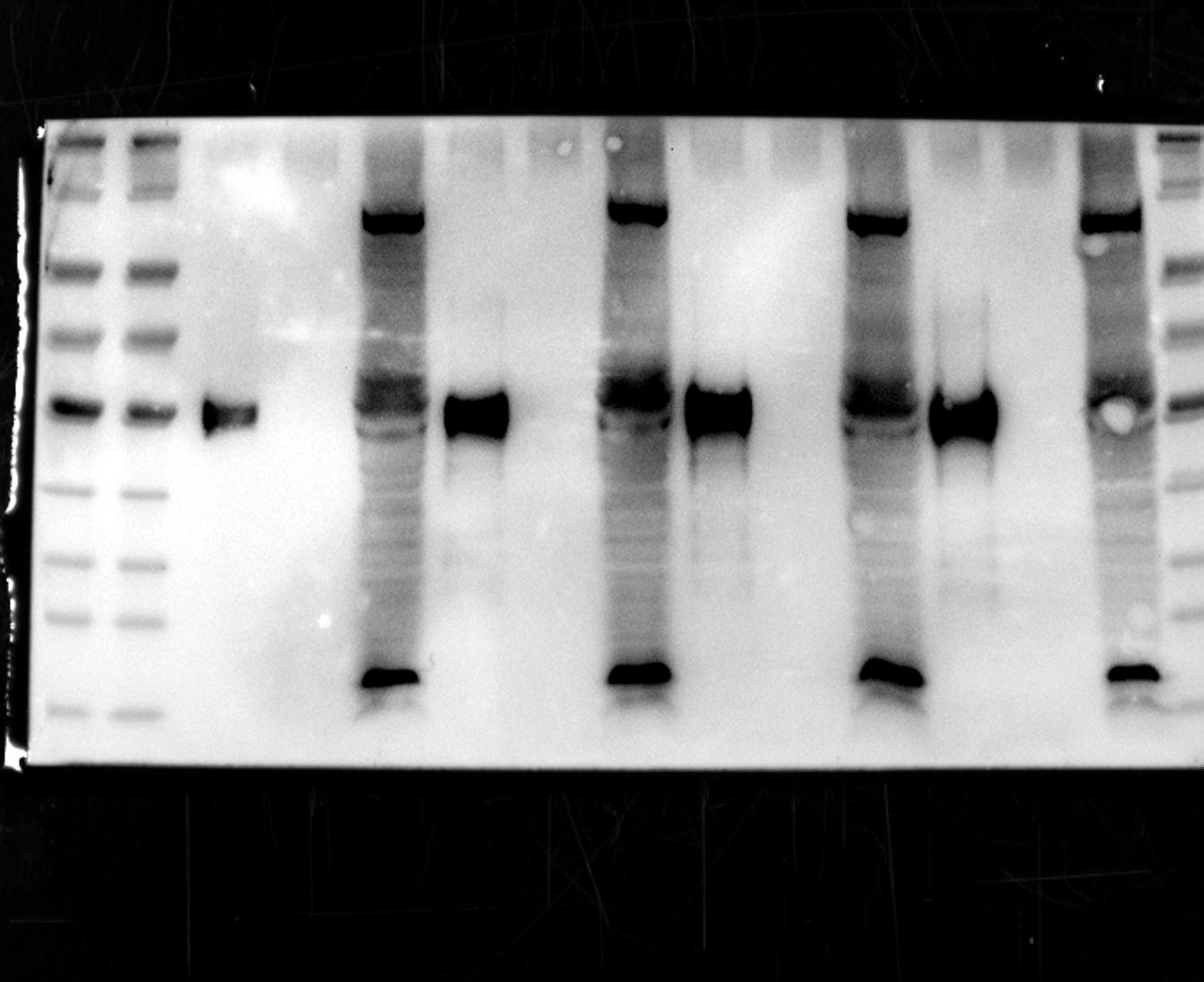

Supplement: Supplementary file 2 — Supporting Information [file ADVS-12-e06225-s001.zip › CO-IP/PDL1-CD75液-HIP HIgG HIN-1.Tif]

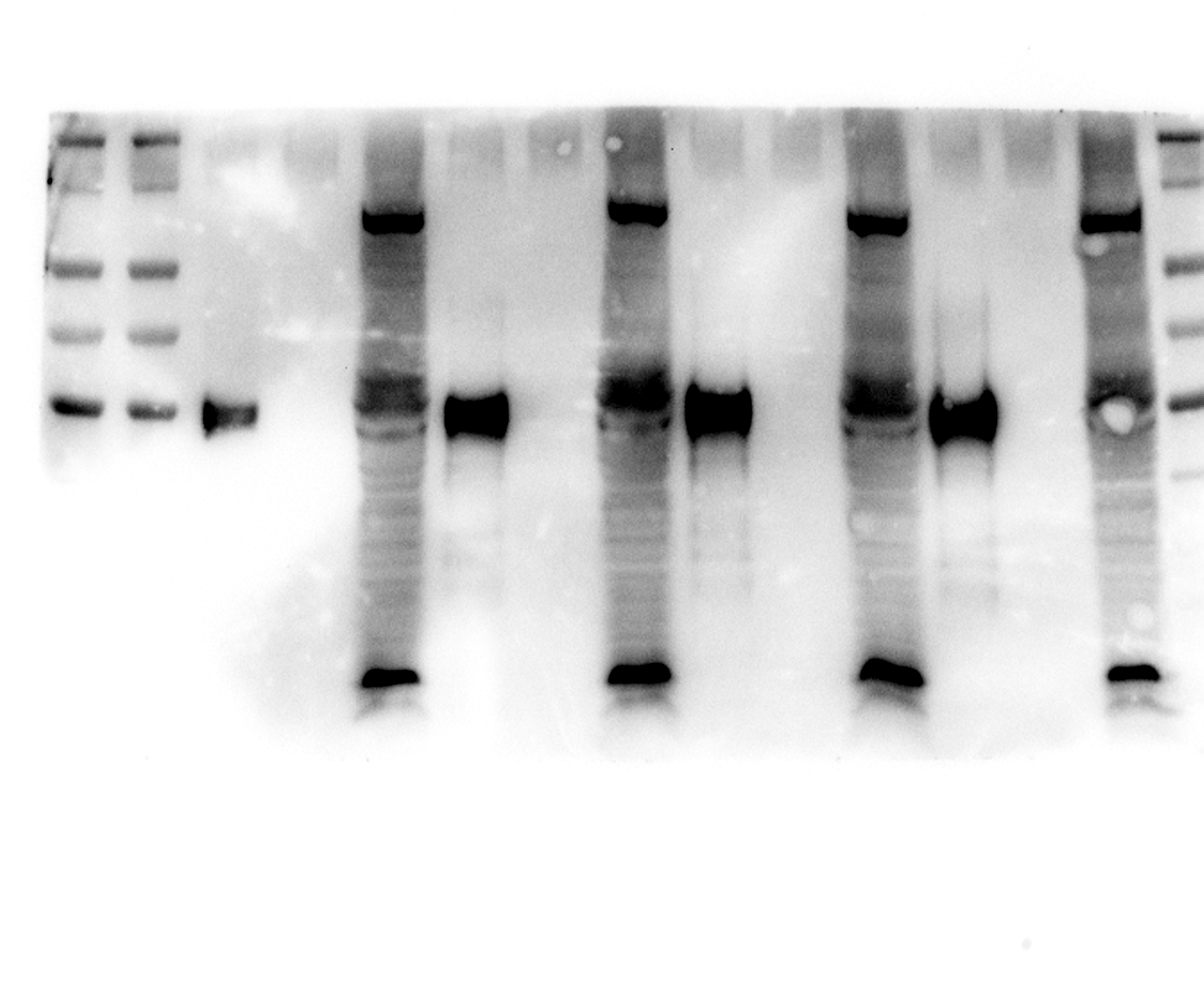

Supplement: Supplementary file 2 — Supporting Information [file ADVS-12-e06225-s001.zip › CO-IP/PDL1-CD75液-HIP HIgG HIN-10.Tif]

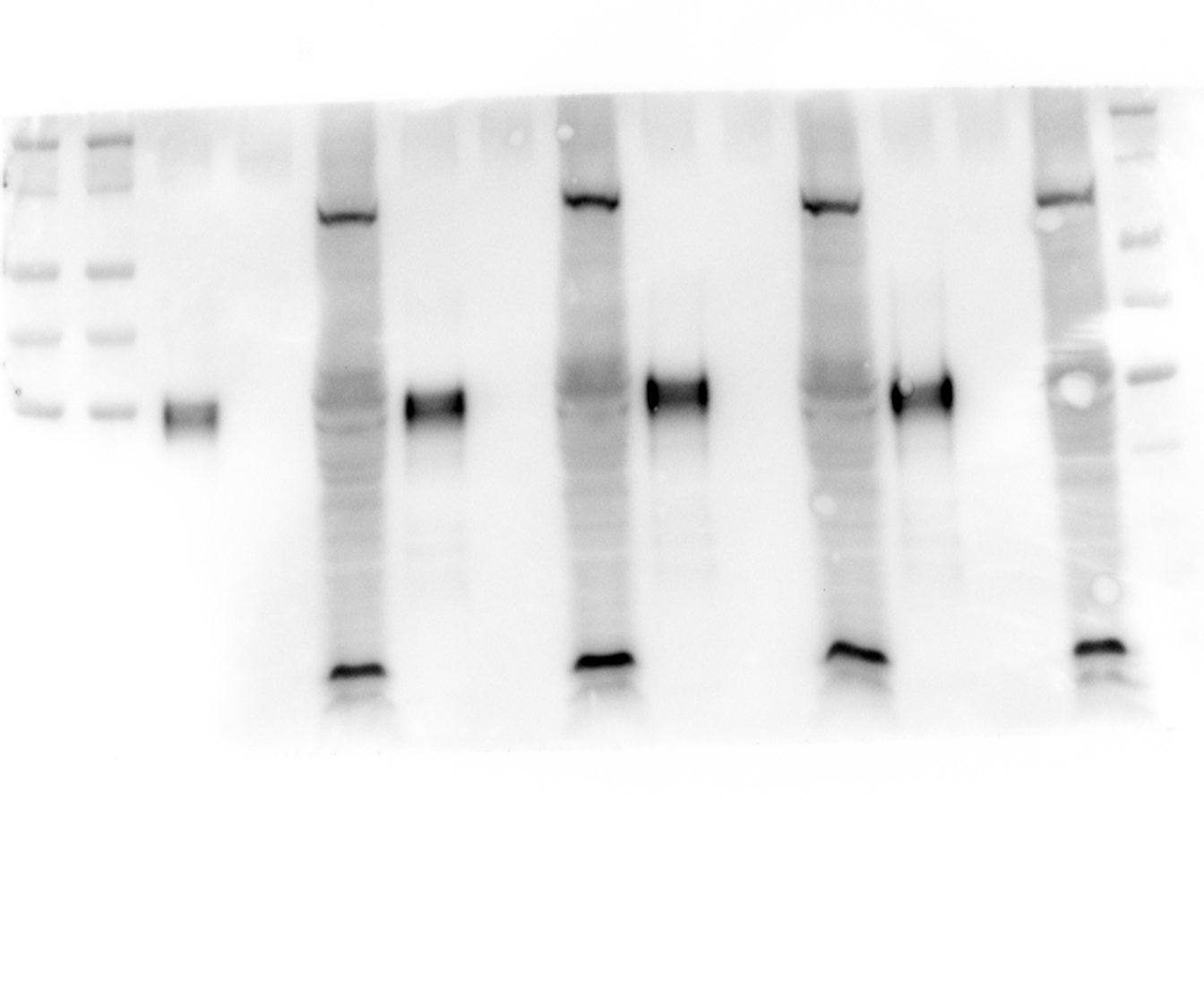

Supplement: Supplementary file 2 — Supporting Information [file ADVS-12-e06225-s001.zip › CO-IP/PDL1-CD75液-HIP HIgG HIN-11.Tif]

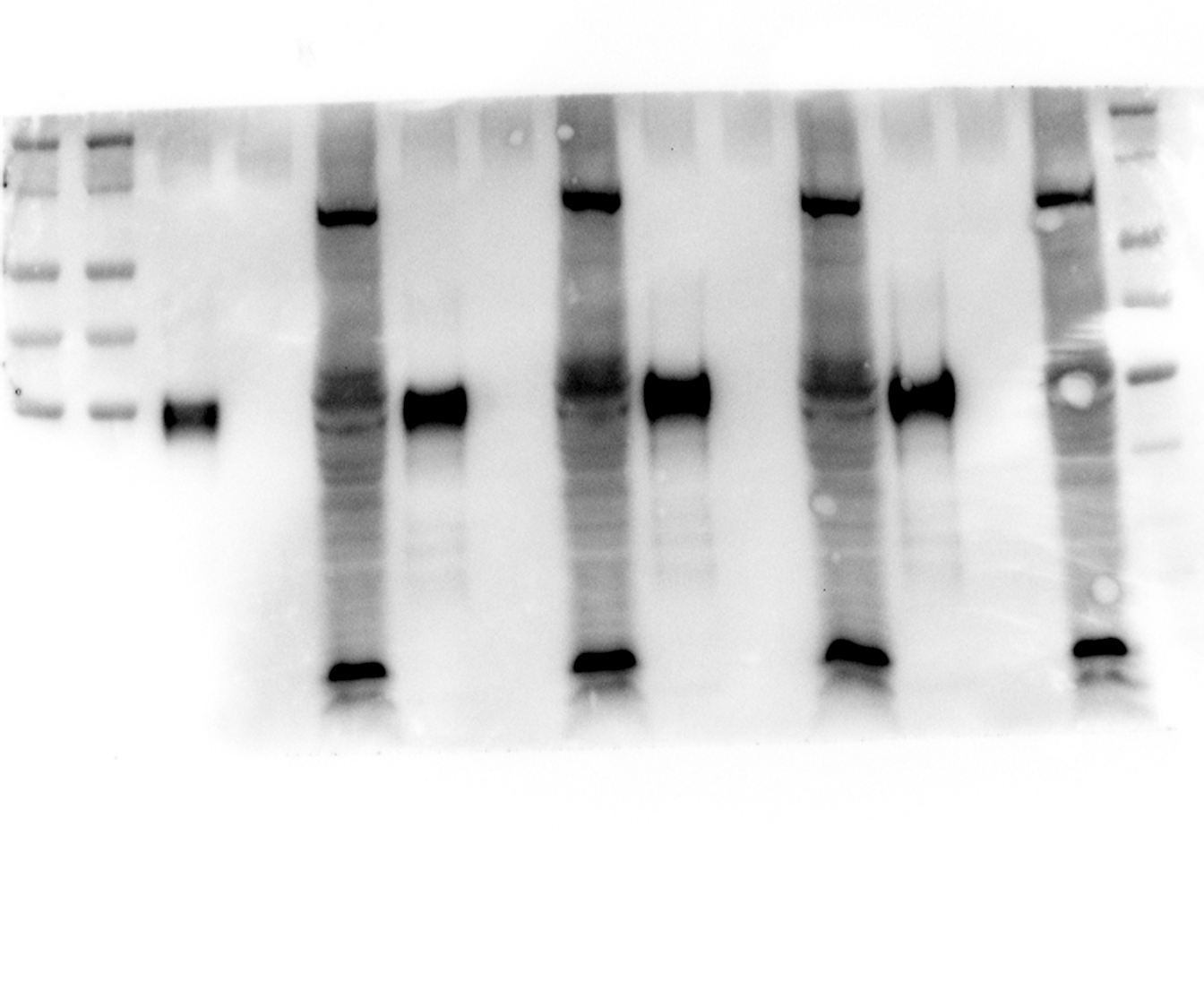

Supplement: Supplementary file 2 — Supporting Information [file ADVS-12-e06225-s001.zip › CO-IP/PDL1-CD75液-HIP HIgG HIN-12.Tif]

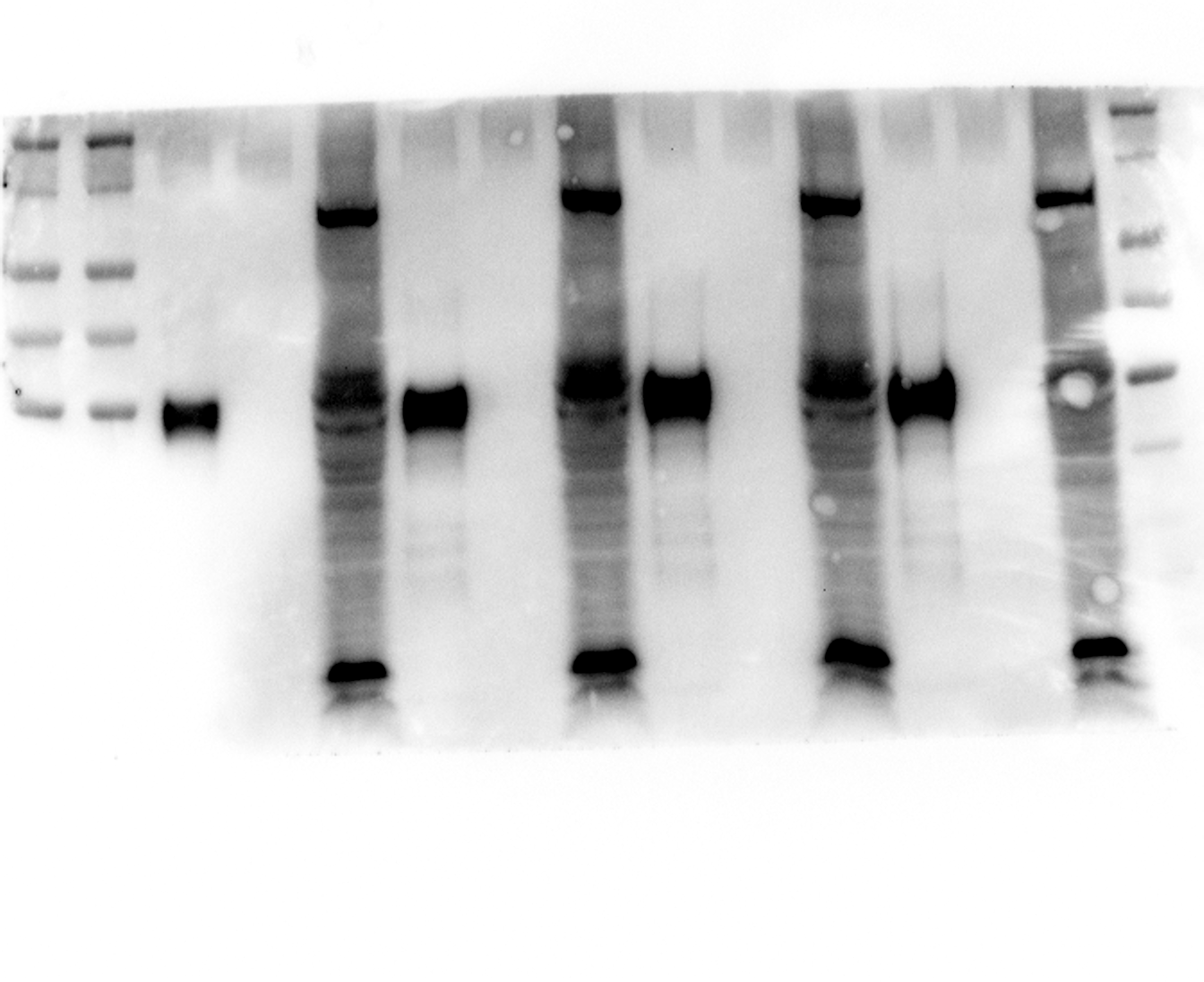

Supplement: Supplementary file 2 — Supporting Information [file ADVS-12-e06225-s001.zip › CO-IP/PDL1-CD75液-HIP HIgG HIN-14.Tif]

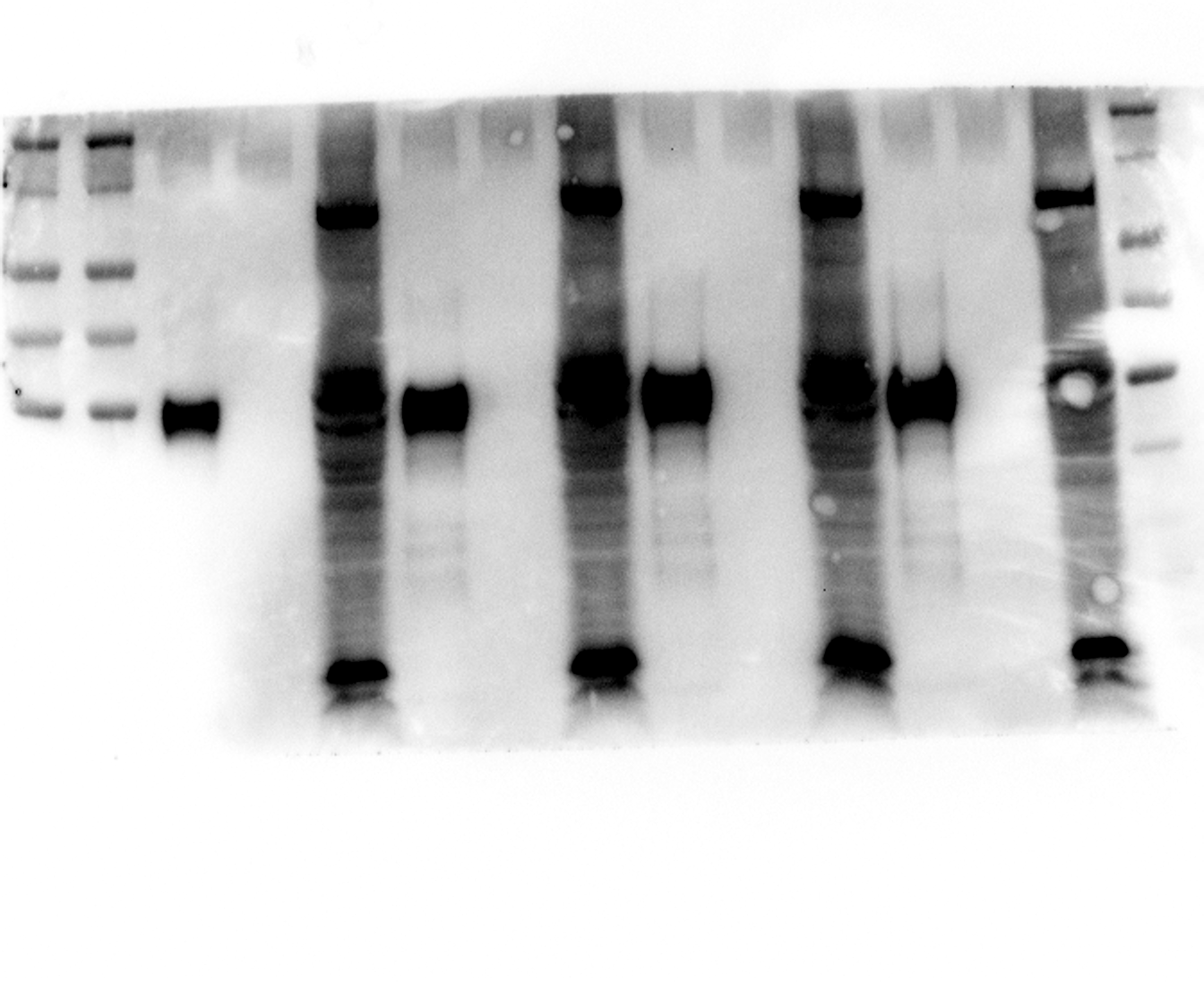

Supplement: Supplementary file 2 — Supporting Information [file ADVS-12-e06225-s001.zip › CO-IP/PDL1-CD75液-HIP HIgG HIN-15.Tif]

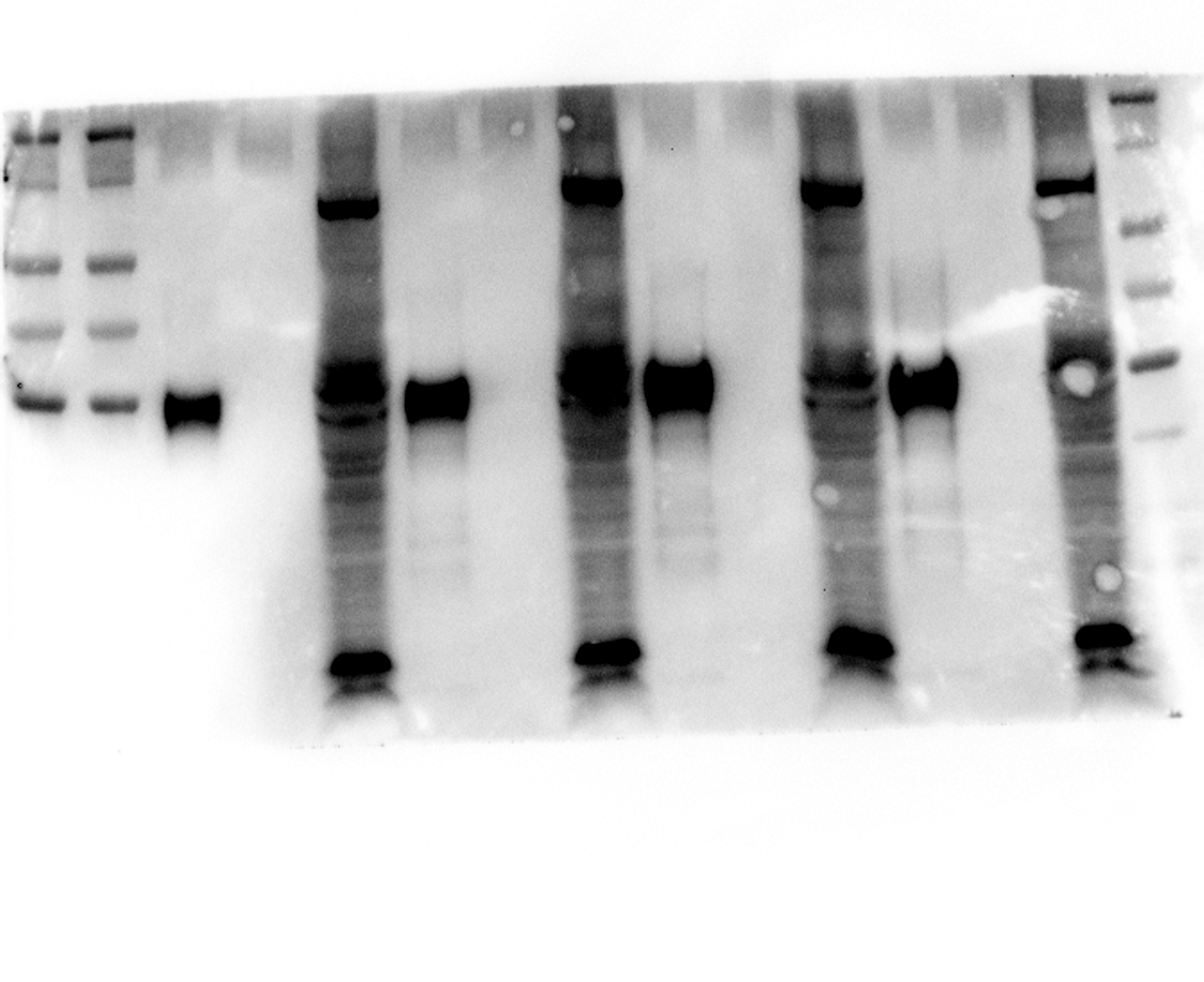

Supplement: Supplementary file 2 — Supporting Information [file ADVS-12-e06225-s001.zip › CO-IP/PDL1-CD75液-HIP HIgG HIN-16.Tif]

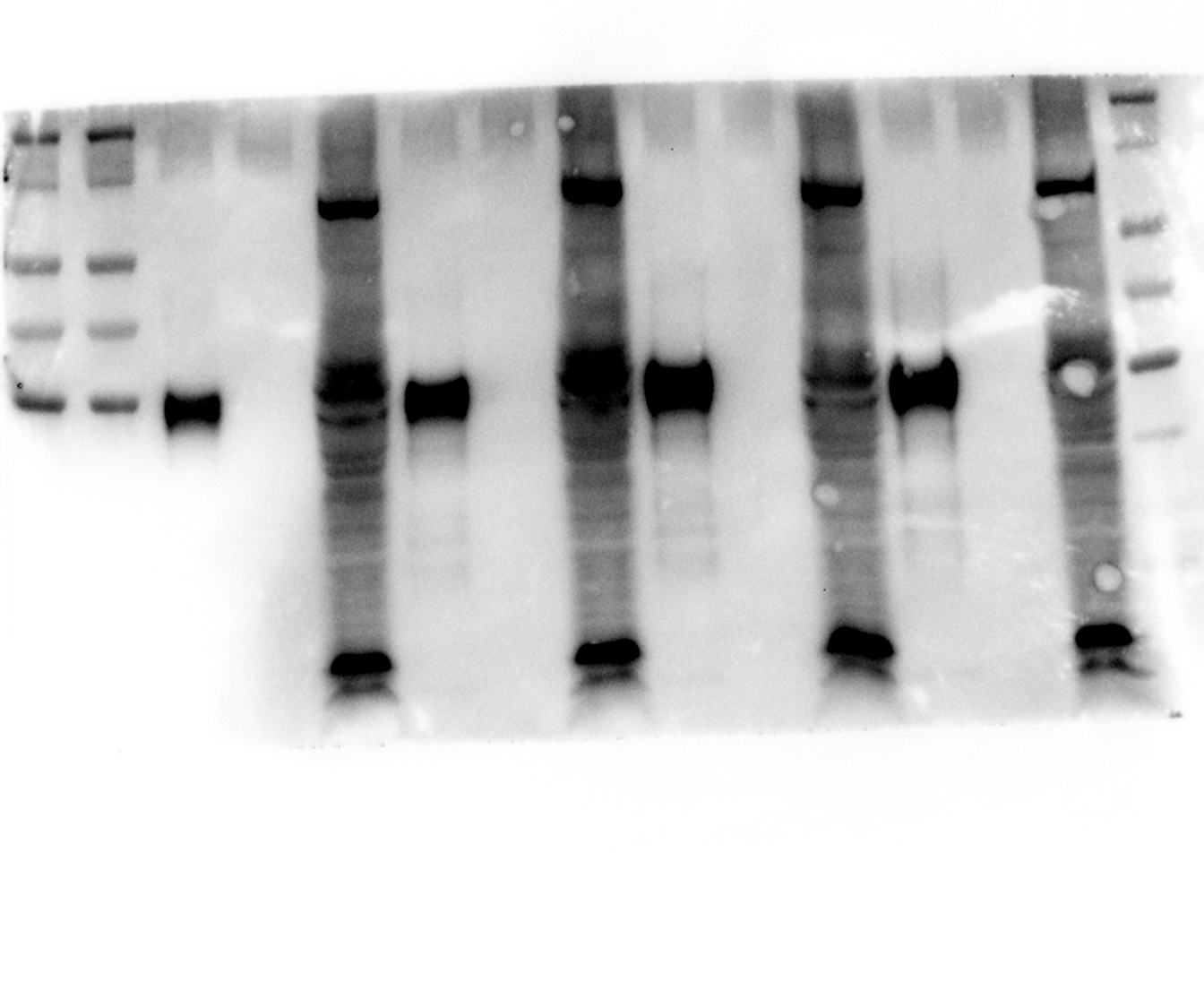

Supplement: Supplementary file 2 — Supporting Information [file ADVS-12-e06225-s001.zip › CO-IP/PDL1-CD75液-HIP HIgG HIN-17.Tif]

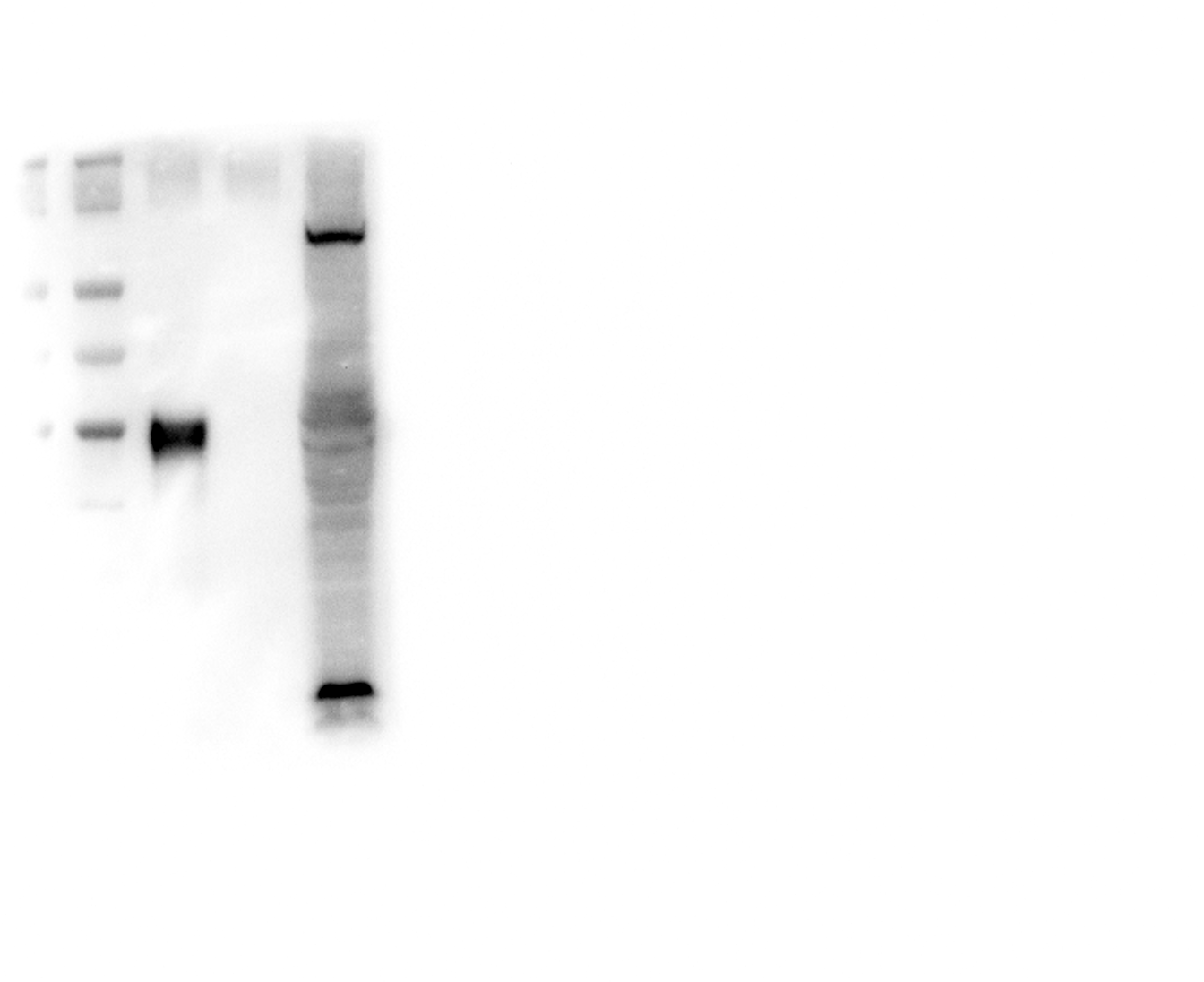

Supplement: Supplementary file 2 — Supporting Information [file ADVS-12-e06225-s001.zip › CO-IP/PDL1-CD75液-HIP HIgG HIN-18.Tif]

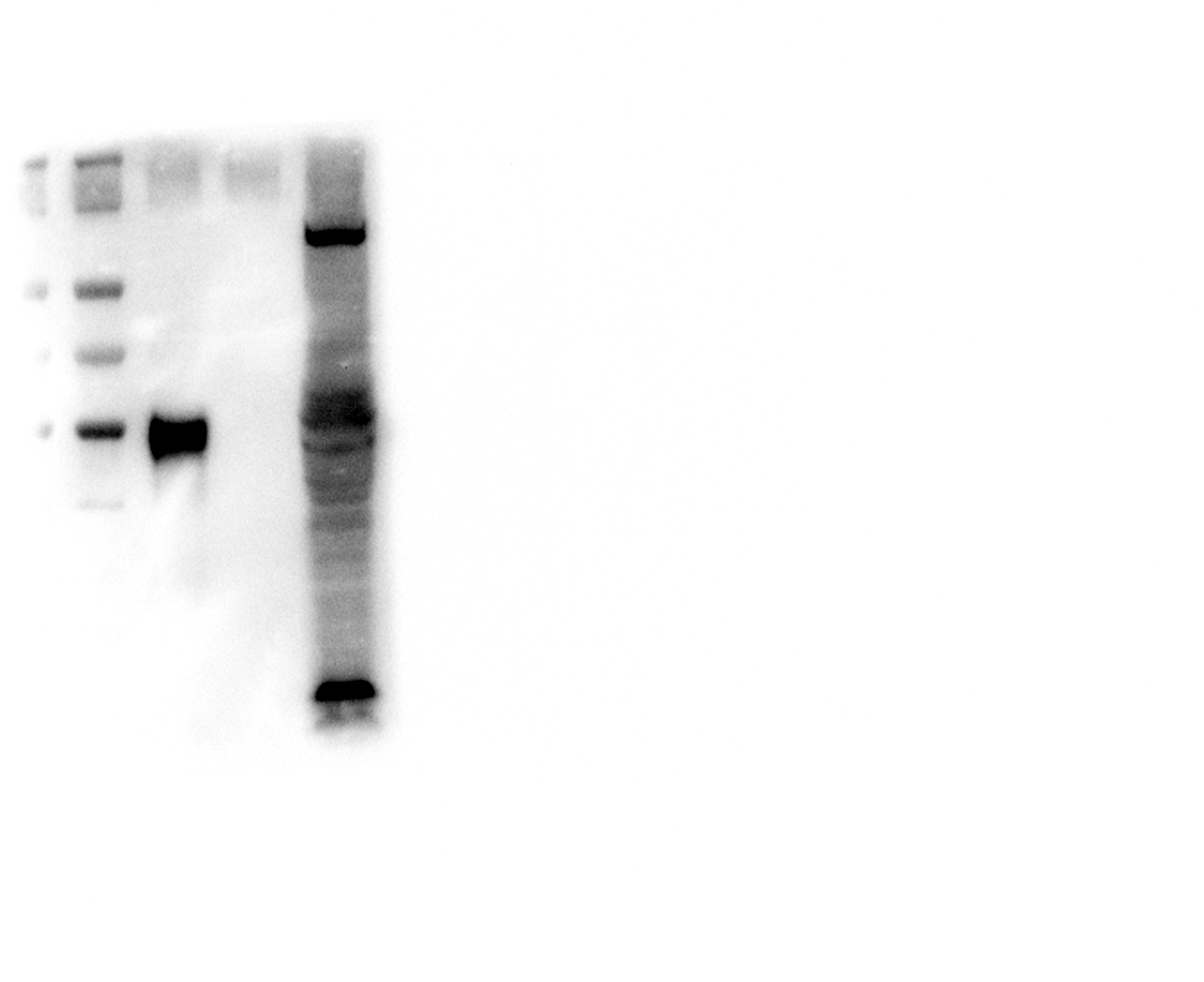

Supplement: Supplementary file 2 — Supporting Information [file ADVS-12-e06225-s001.zip › CO-IP/PDL1-CD75液-HIP HIgG HIN-19.Tif]

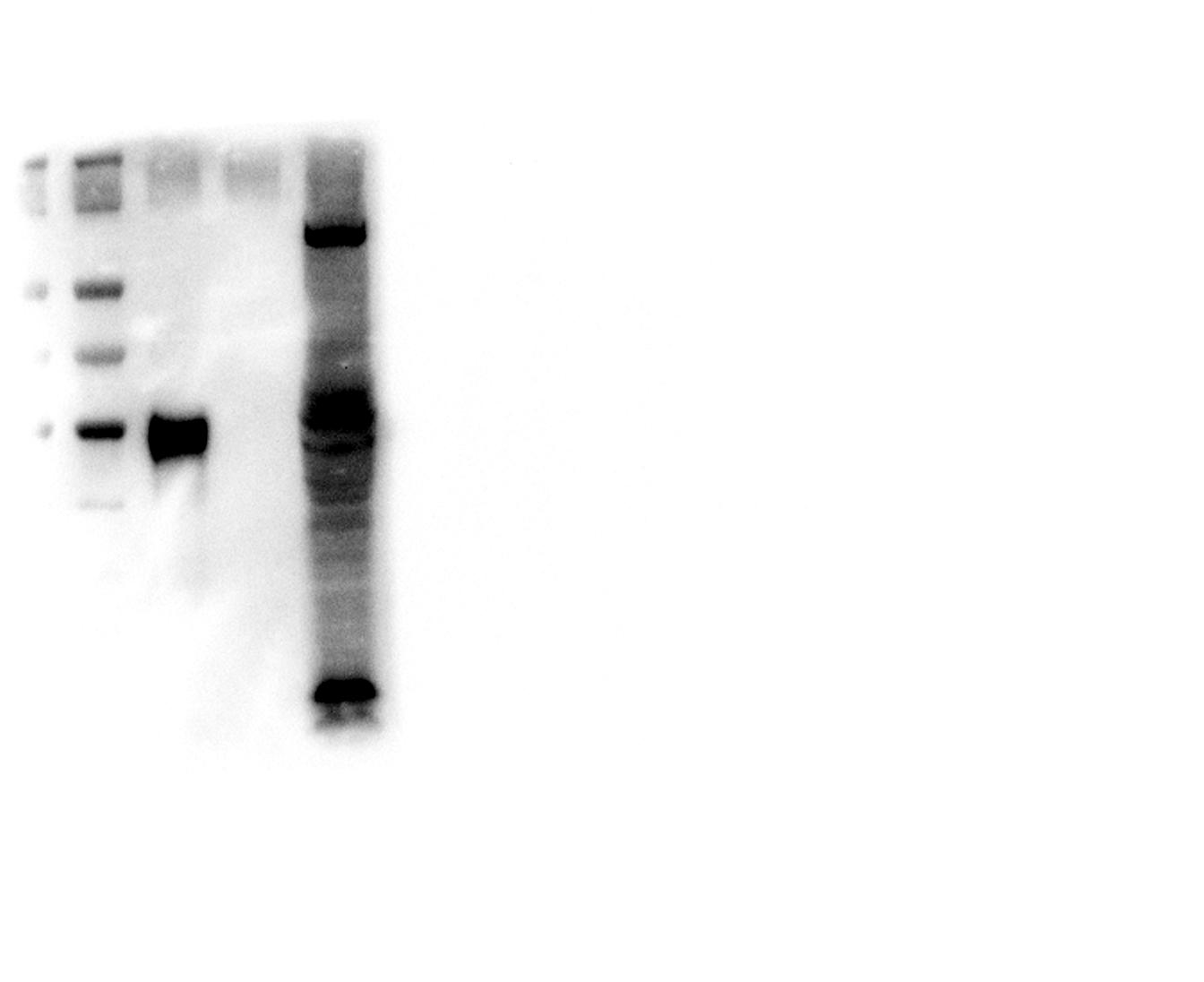

Supplement: Supplementary file 2 — Supporting Information [file ADVS-12-e06225-s001.zip › CO-IP/PDL1-CD75液-HIP HIgG HIN-20.Tif]

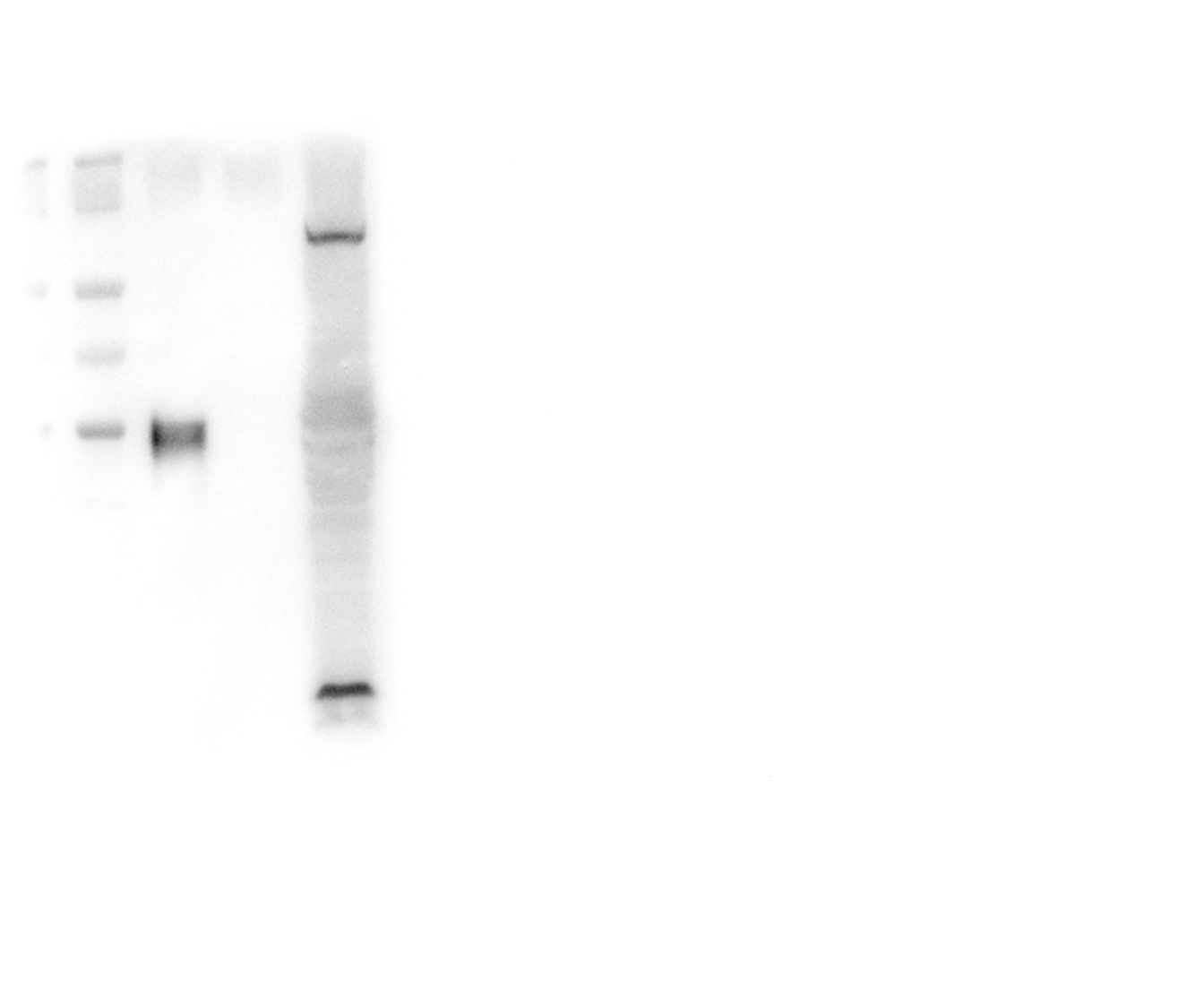

Supplement: Supplementary file 2 — Supporting Information [file ADVS-12-e06225-s001.zip › CO-IP/PDL1-CD75液-HIP HIgG HIN-22.Tif]

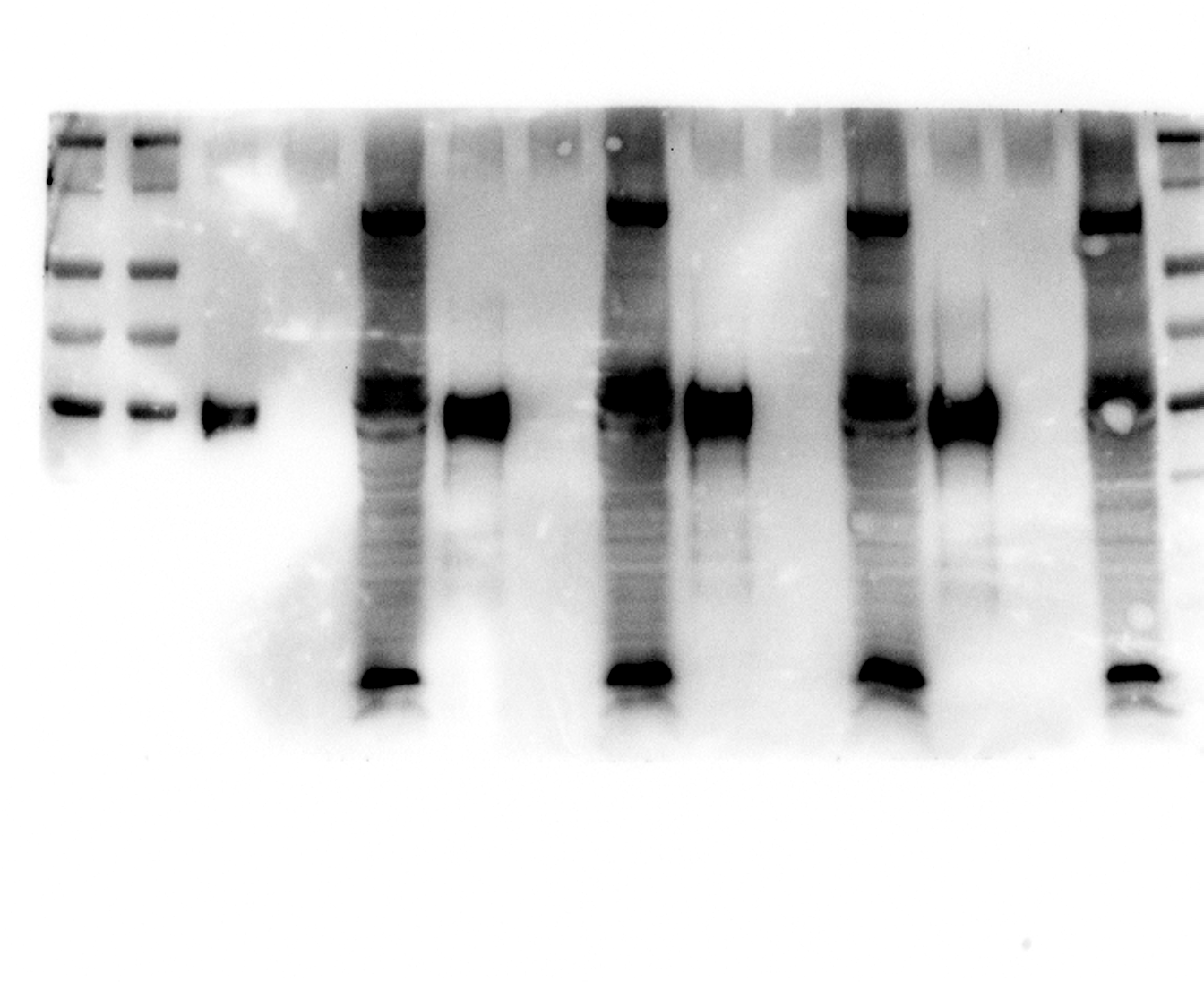

Supplement: Supplementary file 2 — Supporting Information [file ADVS-12-e06225-s001.zip › CO-IP/PDL1-CD75液-HIP HIgG HIN-3.Tif]

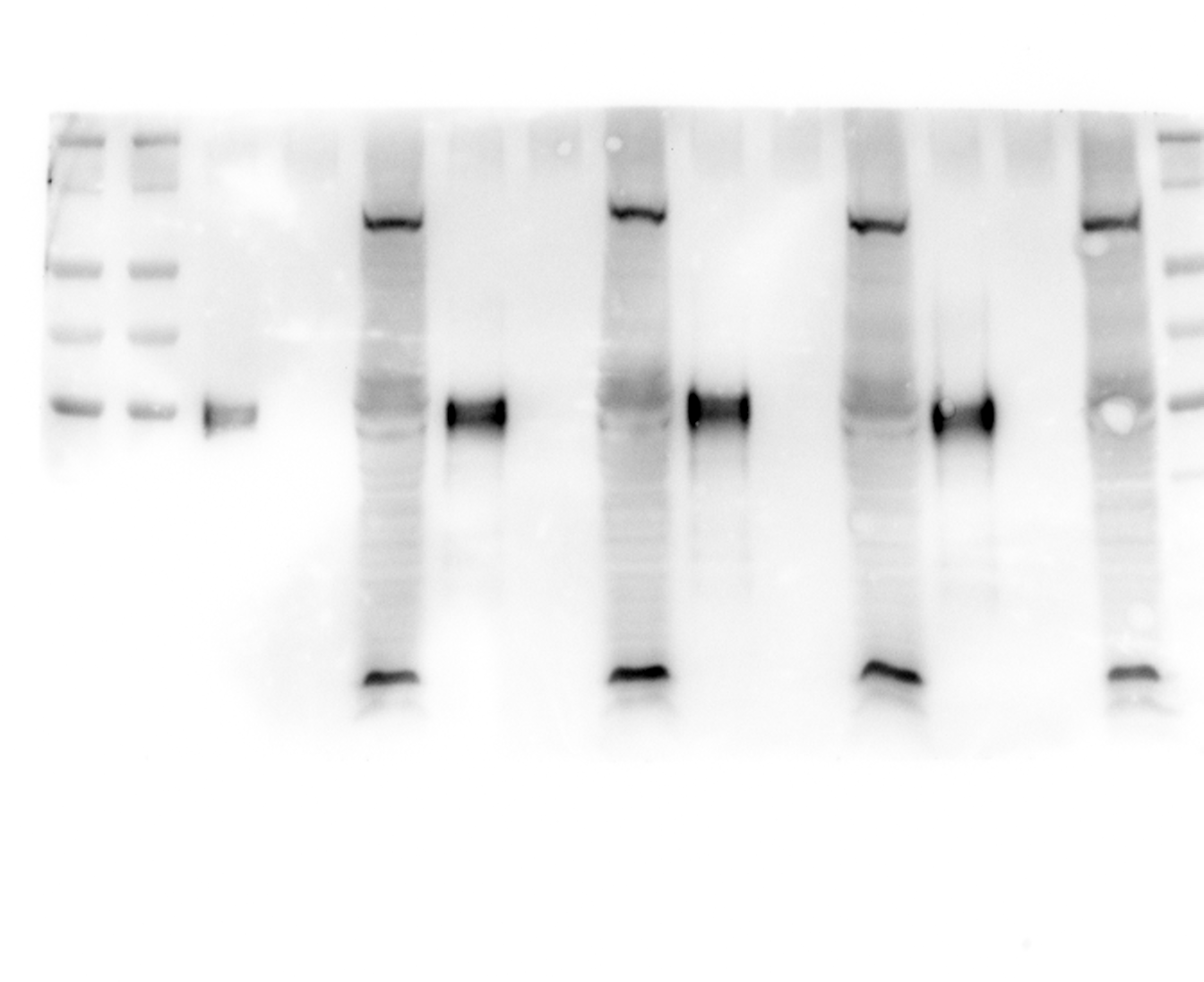

Supplement: Supplementary file 2 — Supporting Information [file ADVS-12-e06225-s001.zip › CO-IP/PDL1-CD75液-HIP HIgG HIN-4.Tif]

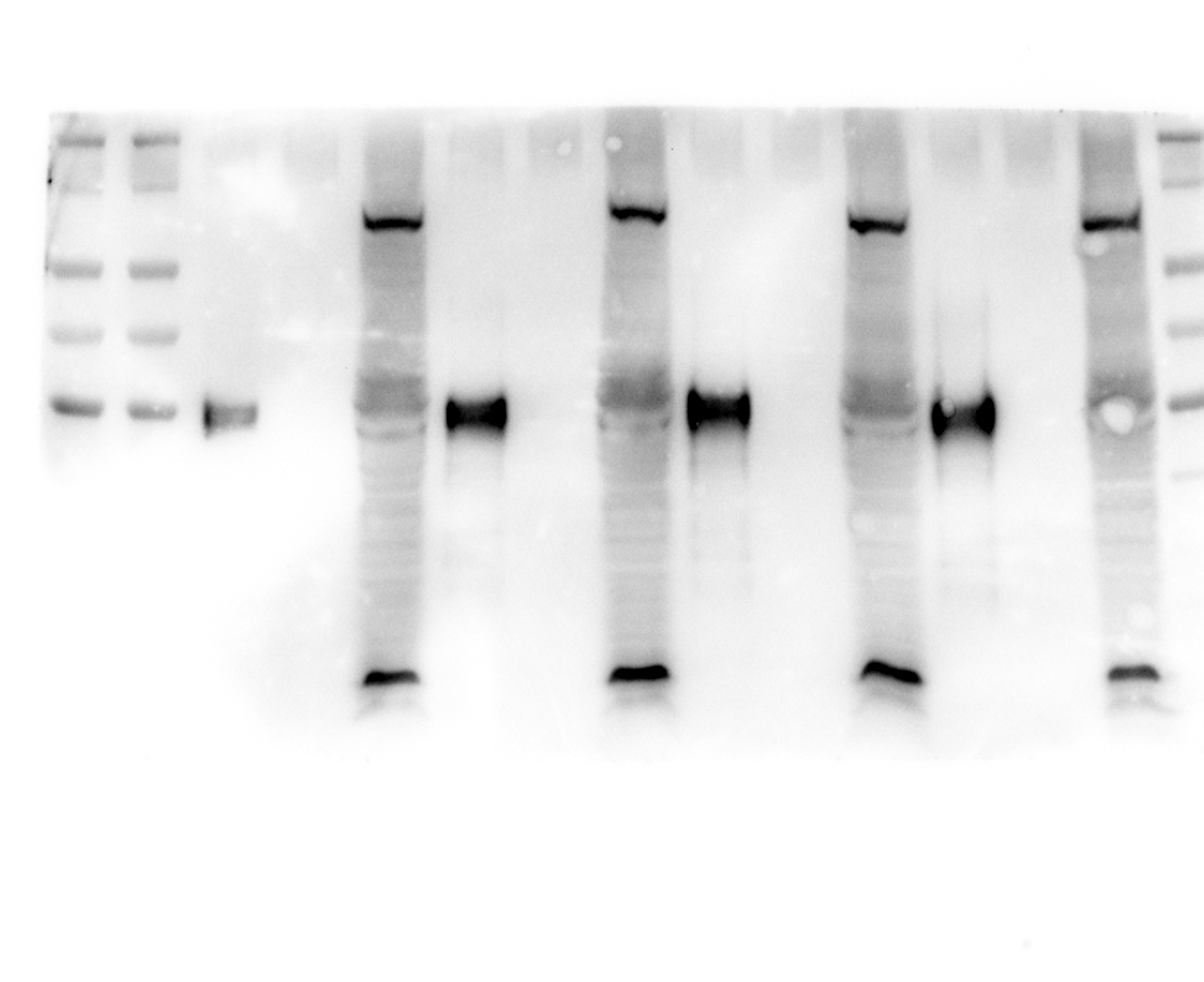

Supplement: Supplementary file 2 — Supporting Information [file ADVS-12-e06225-s001.zip › CO-IP/PDL1-CD75液-HIP HIgG HIN-5.Tif]

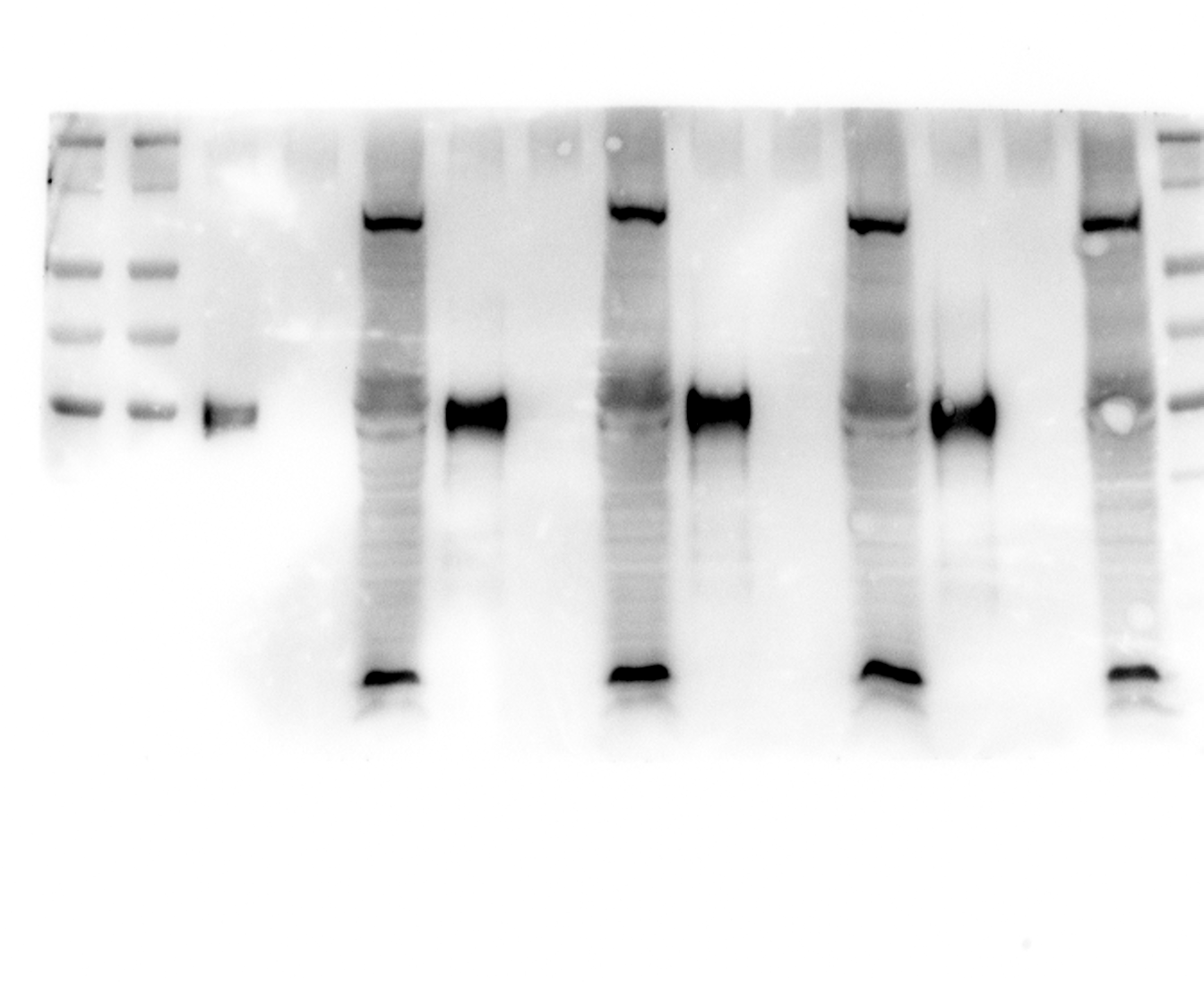

Supplement: Supplementary file 2 — Supporting Information [file ADVS-12-e06225-s001.zip › CO-IP/PDL1-CD75液-HIP HIgG HIN-6.Tif]

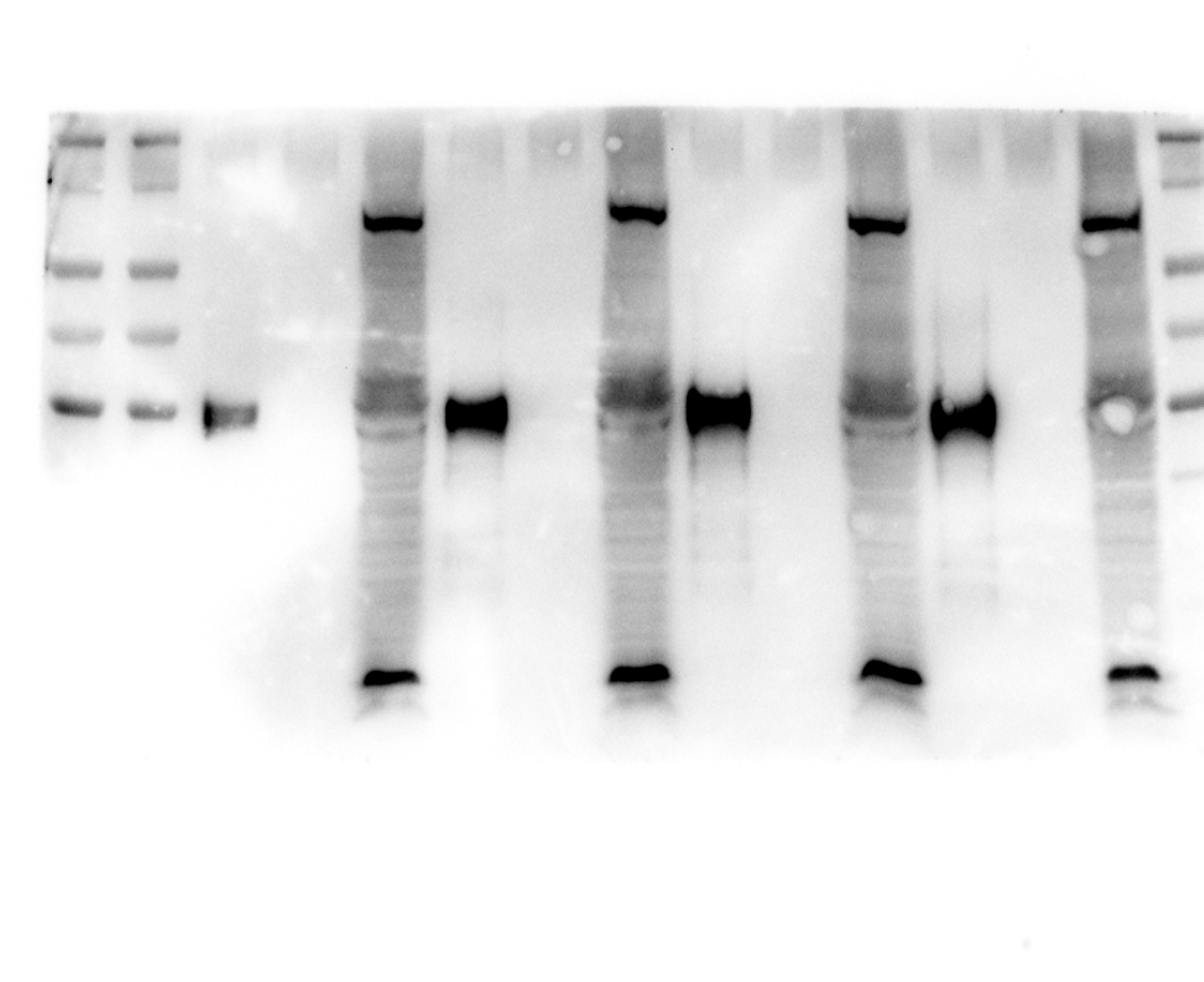

Supplement: Supplementary file 2 — Supporting Information [file ADVS-12-e06225-s001.zip › CO-IP/PDL1-CD75液-HIP HIgG HIN-7.Tif]

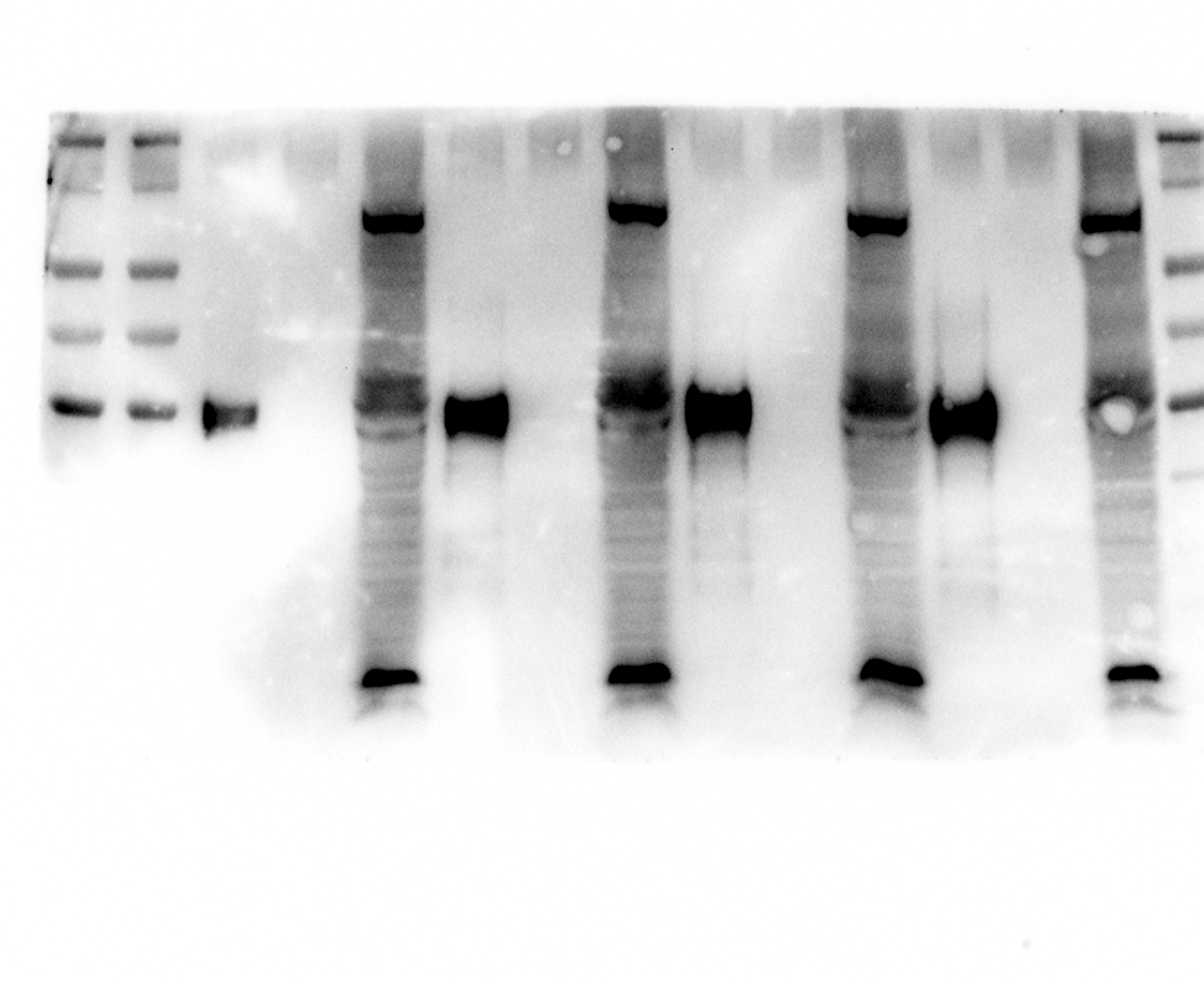

Supplement: Supplementary file 2 — Supporting Information [file ADVS-12-e06225-s001.zip › CO-IP/PDL1-CD75液-HIP HIgG HIN-8.Tif]

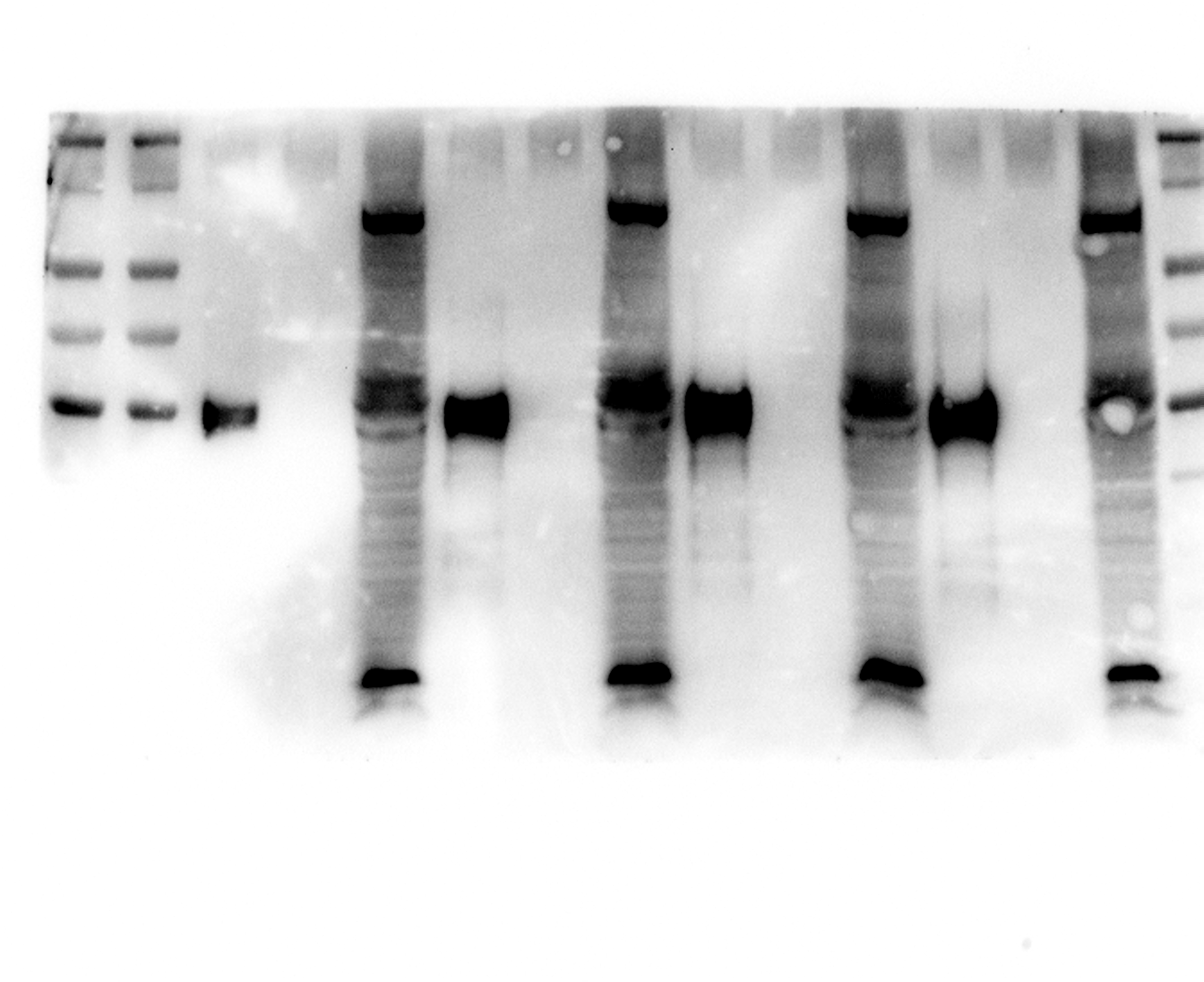

Supplement: Supplementary file 2 — Supporting Information [file ADVS-12-e06225-s001.zip › CO-IP/PDL1-CD75液-HIP HIgG HIN-9.Tif]

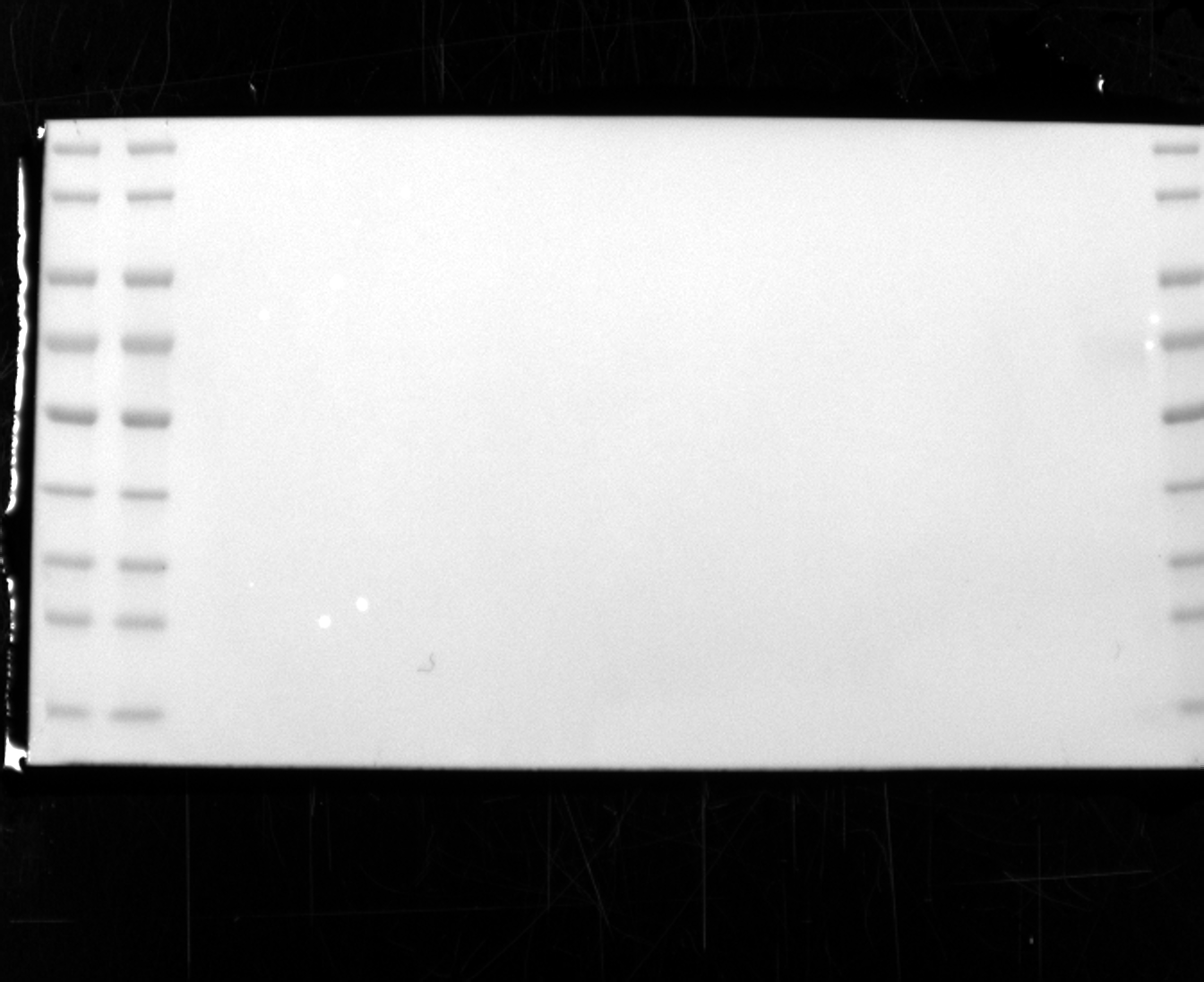

Supplement: Supplementary file 2 — Supporting Information [file ADVS-12-e06225-s001.zip › CO-IP/PDL1-CD75液-HIP HIgG HIN-W.Tif]

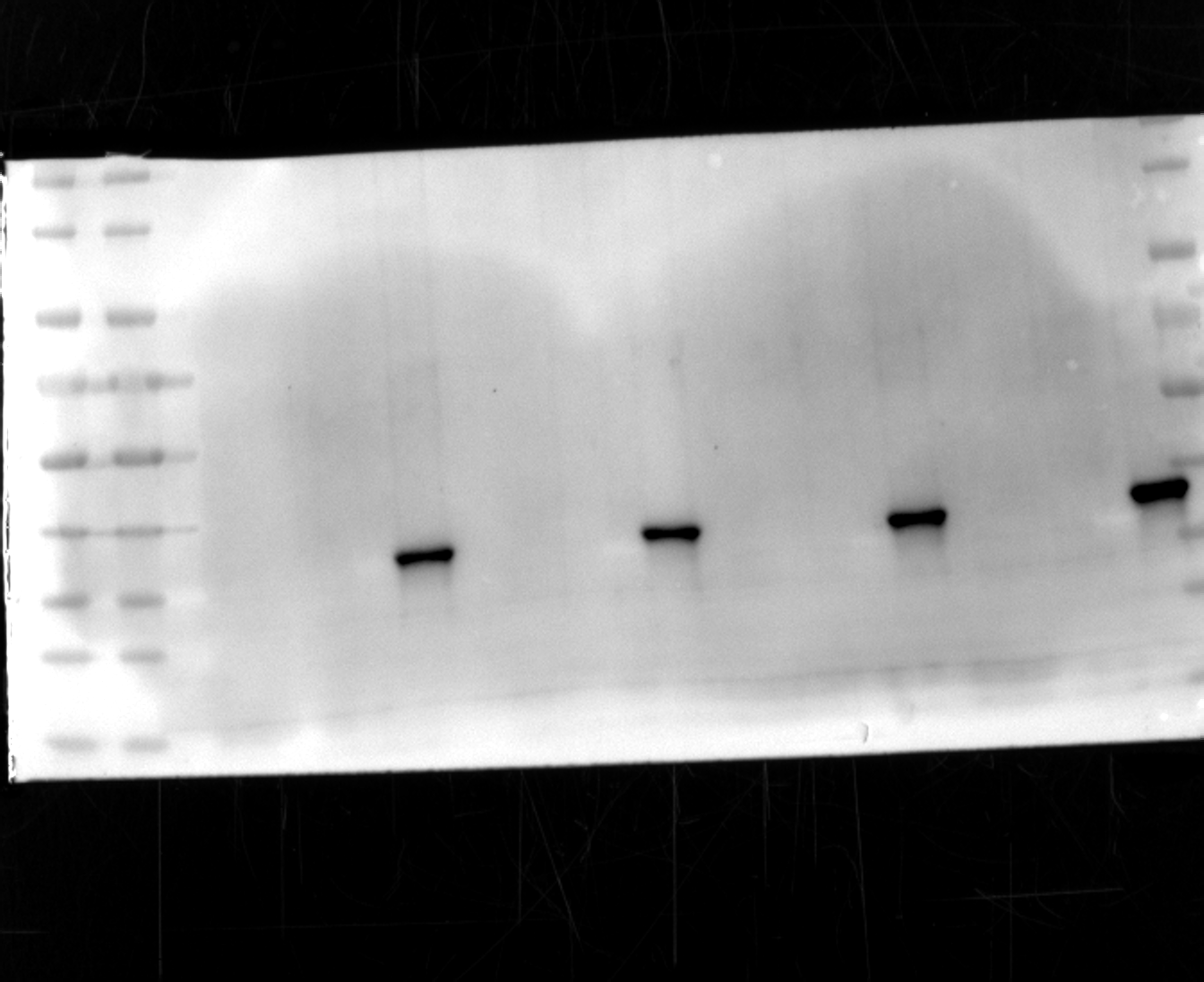

Supplement: Supplementary file 2 — Supporting Information [file ADVS-12-e06225-s001.zip › CO-IP/SNA-H-IP-H- HO HF -M-1.Tif]

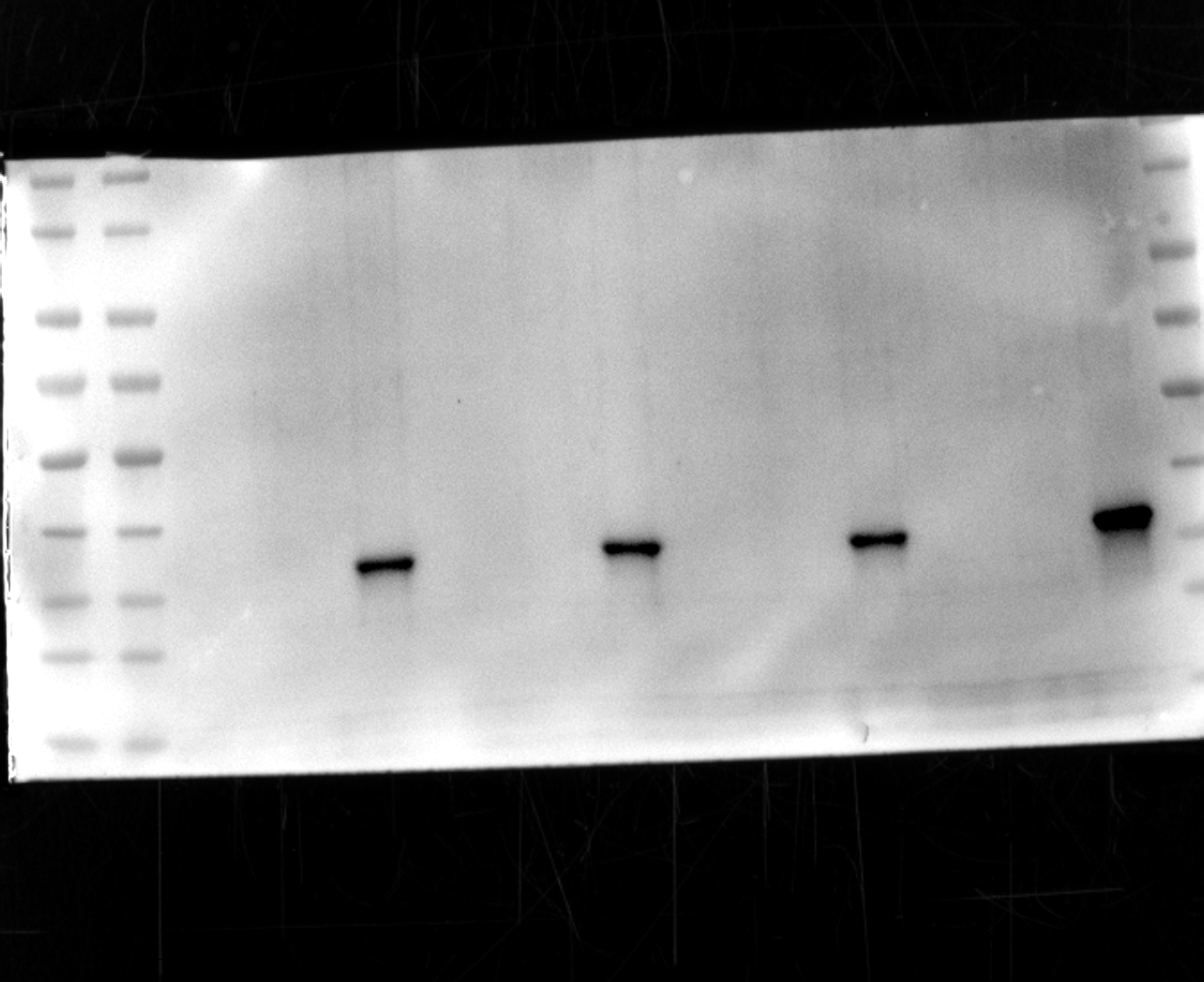

Supplement: Supplementary file 2 — Supporting Information [file ADVS-12-e06225-s001.zip › CO-IP/SNA-H-IP-H- HO HF -M.Tif]

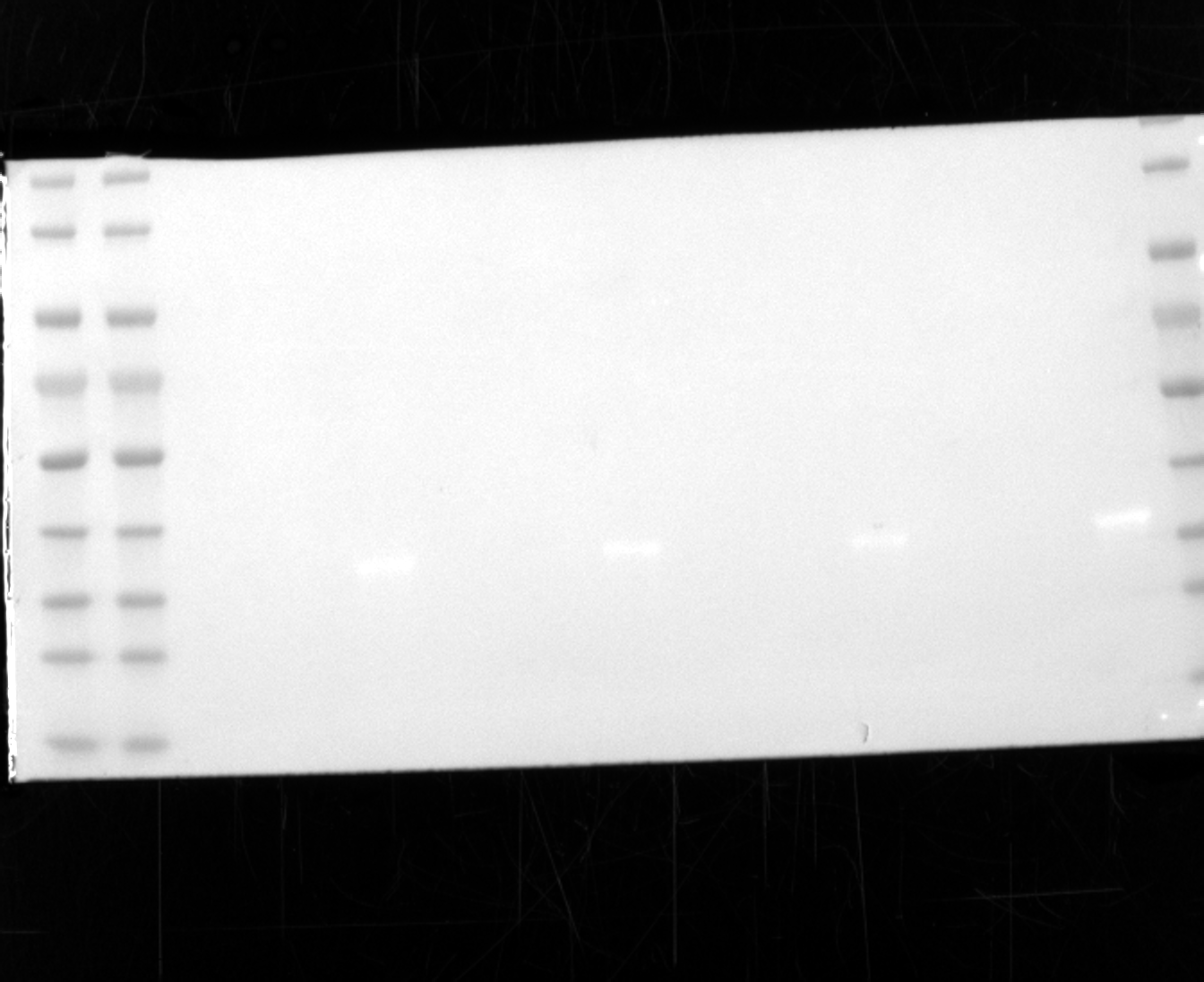

Supplement: Supplementary file 2 — Supporting Information [file ADVS-12-e06225-s001.zip › CO-IP/SNA-H-IP-H- HO HF -W.Tif]

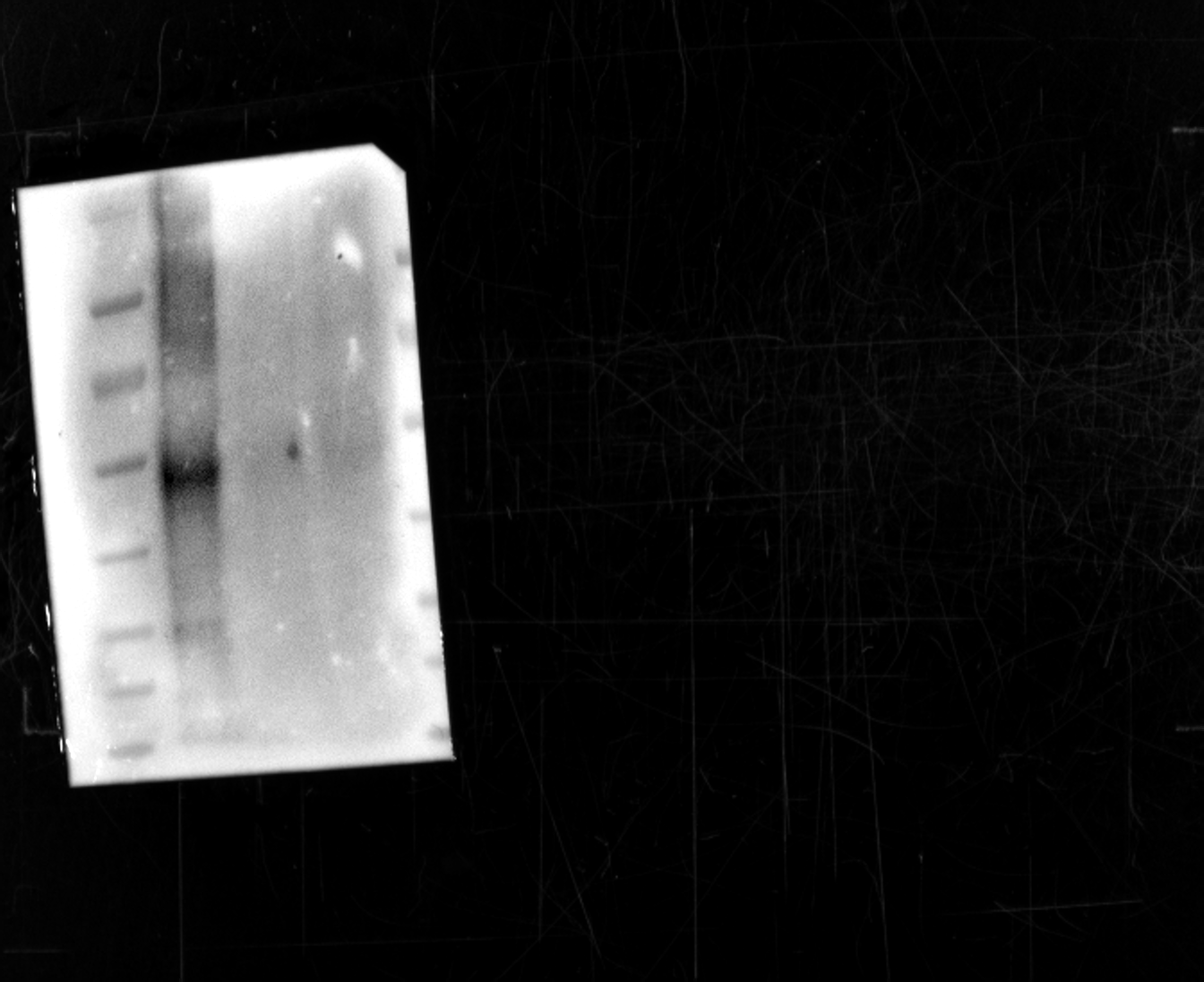

Supplement: Supplementary file 2 — Supporting Information [file ADVS-12-e06225-s001.zip › SNA-Lectin/2-SNA-L-HWT H1 H2-2M.Tif]

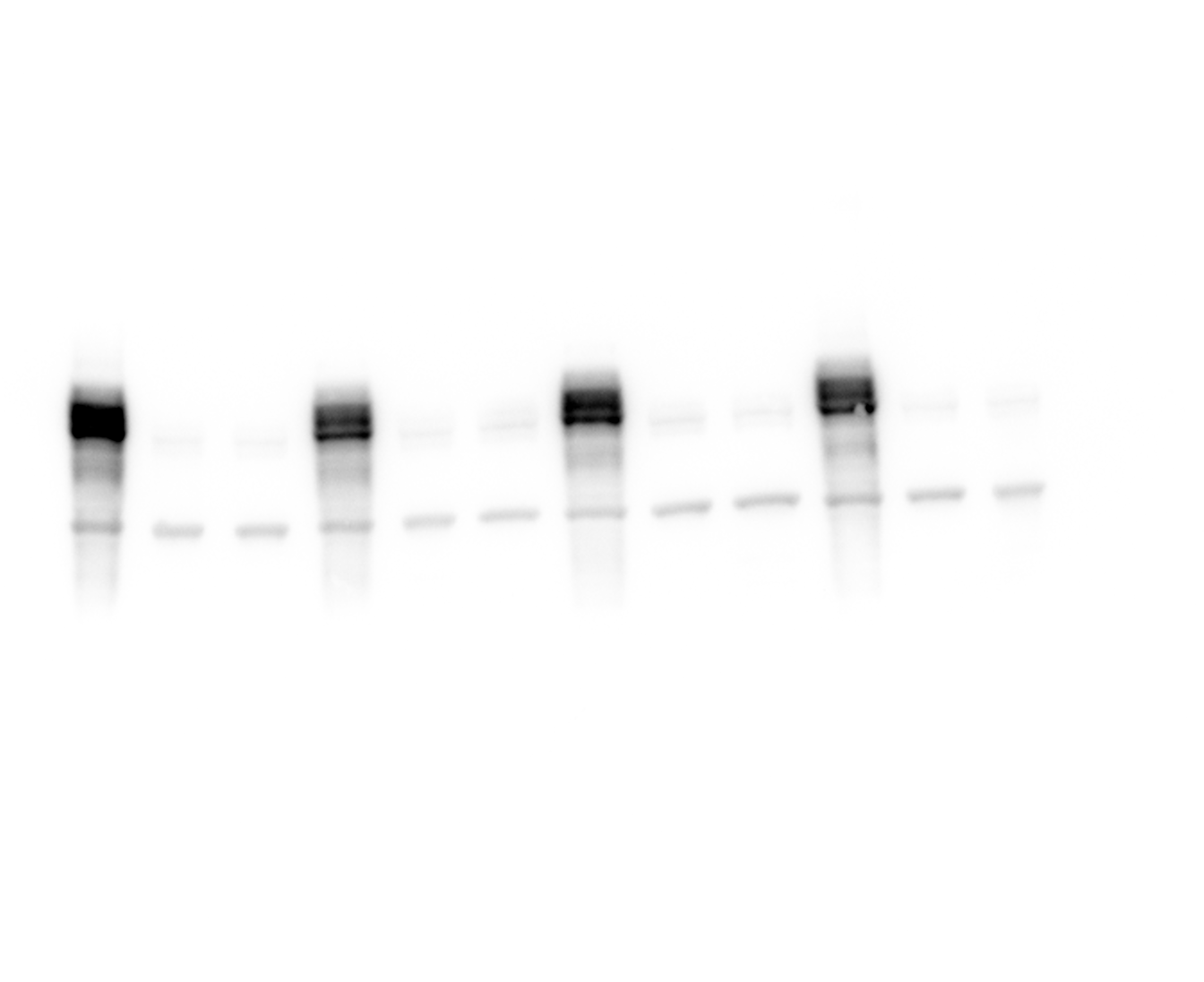

Supplement: Supplementary file 2 — Supporting Information [file ADVS-12-e06225-s001.zip › SNA-Lectin/CD75-116OE 116WT 116WT TQ TQ TQ -1S.Tif]

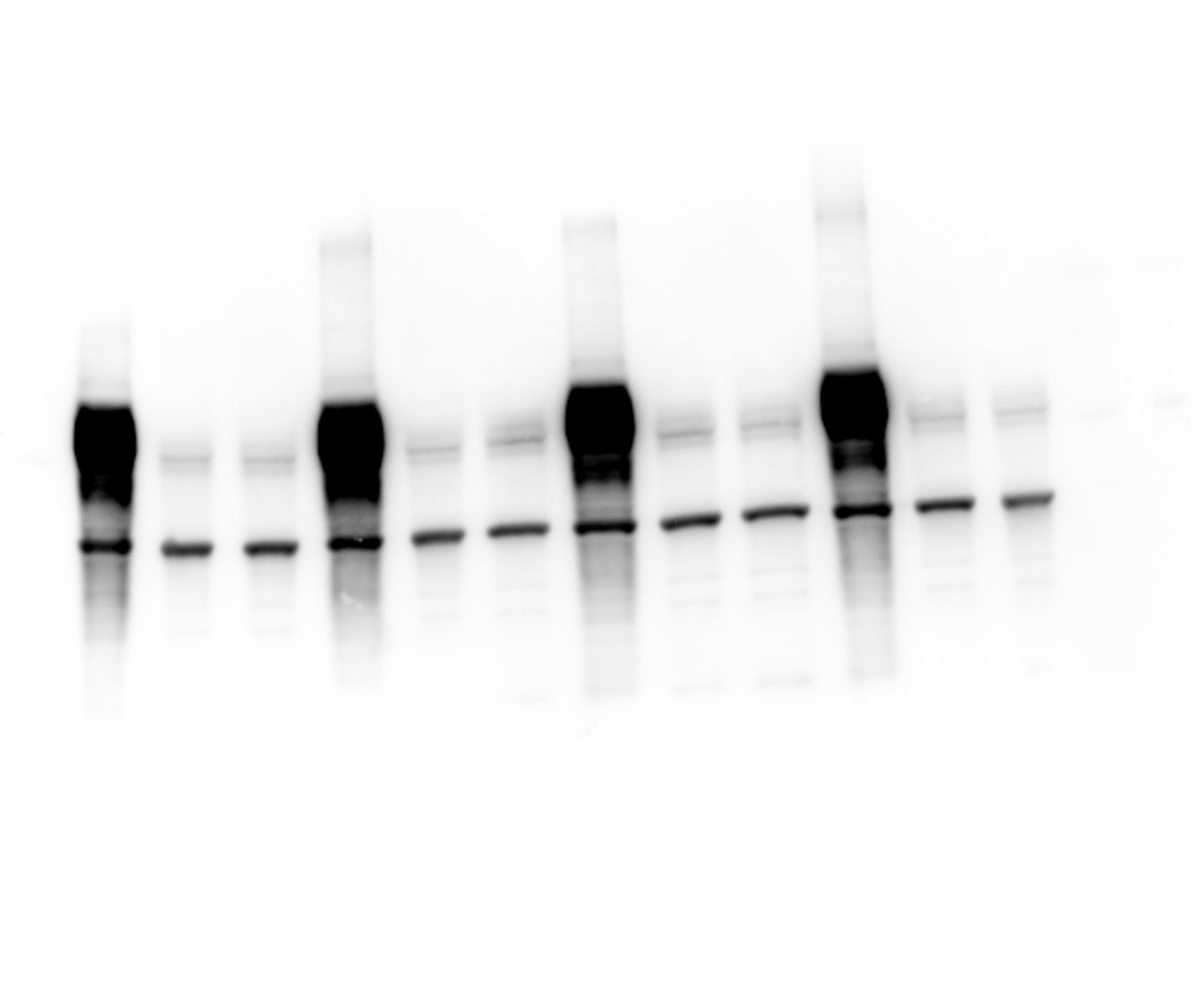

Supplement: Supplementary file 2 — Supporting Information [file ADVS-12-e06225-s001.zip › SNA-Lectin/CD75-116OE 116WT 116WT TQ TQ TQ -5S-1.Tif]

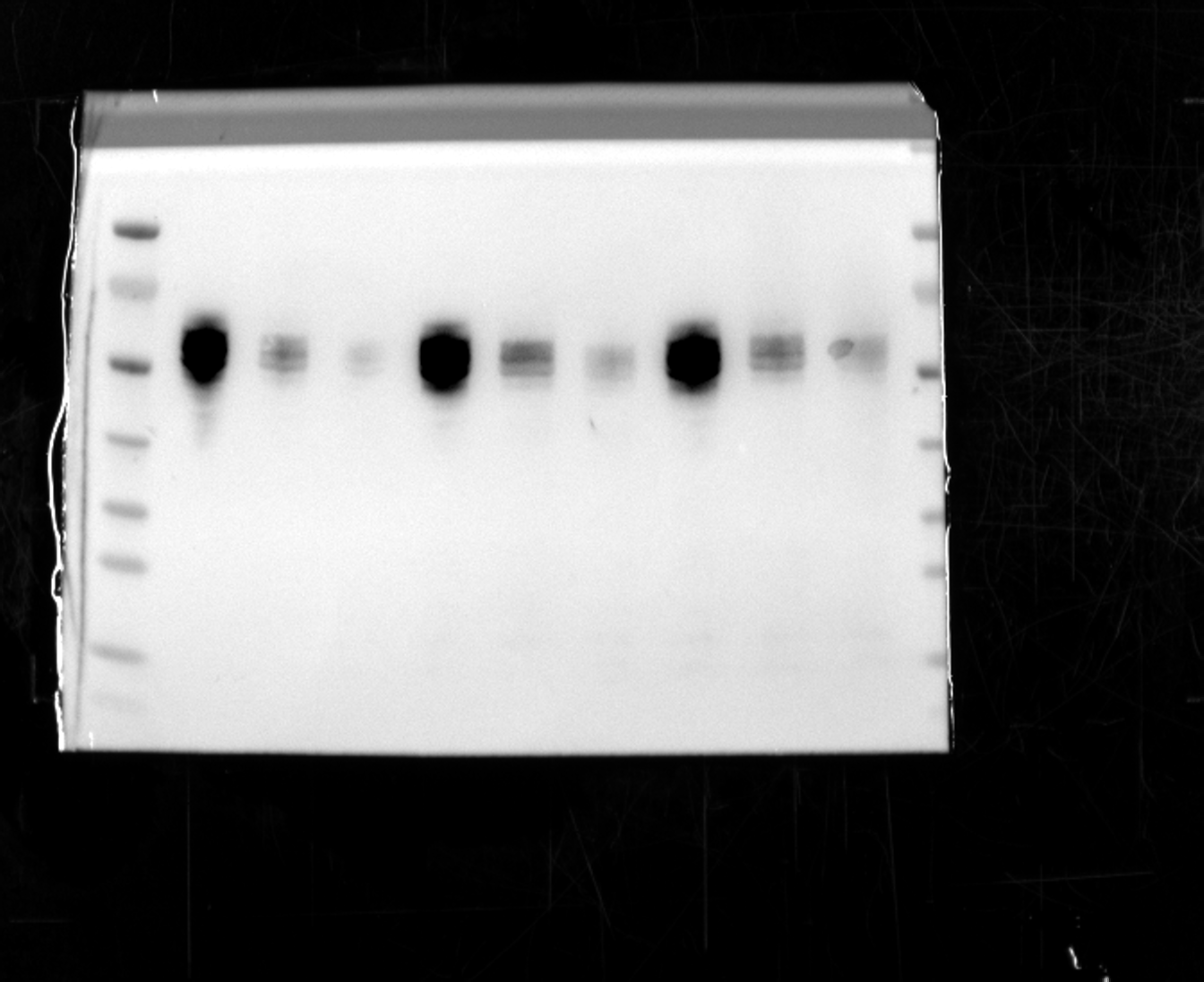

Supplement: Supplementary file 2 — Supporting Information [file ADVS-12-e06225-s001.zip › SNA-Lectin/CD75-H H1 H2 H H1 H2 H H1 H2-11-1.Tif]

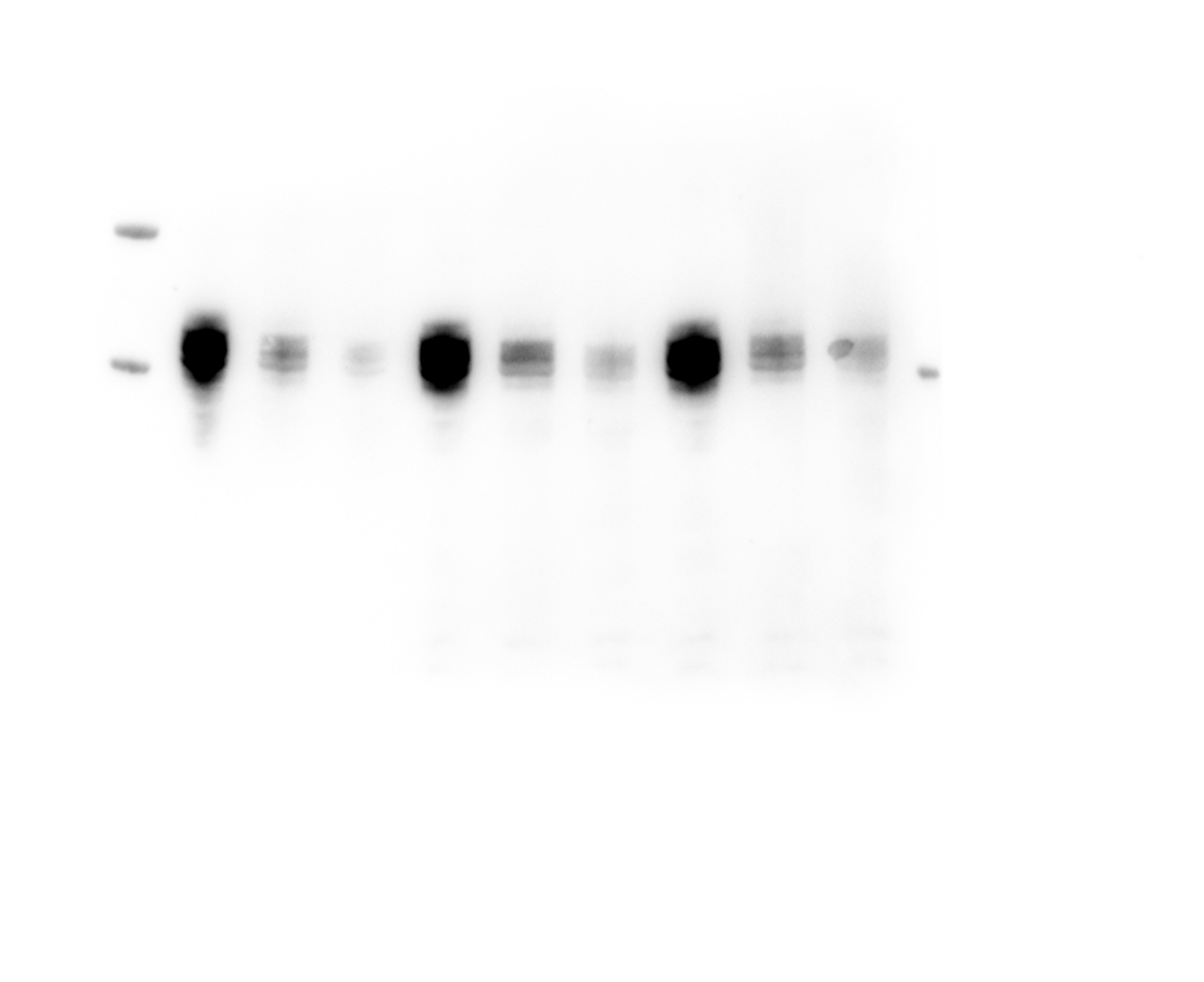

Supplement: Supplementary file 2 — Supporting Information [file ADVS-12-e06225-s001.zip › SNA-Lectin/CD75-H H1 H2 H H1 H2 H H1 H2-11-2.Tif]

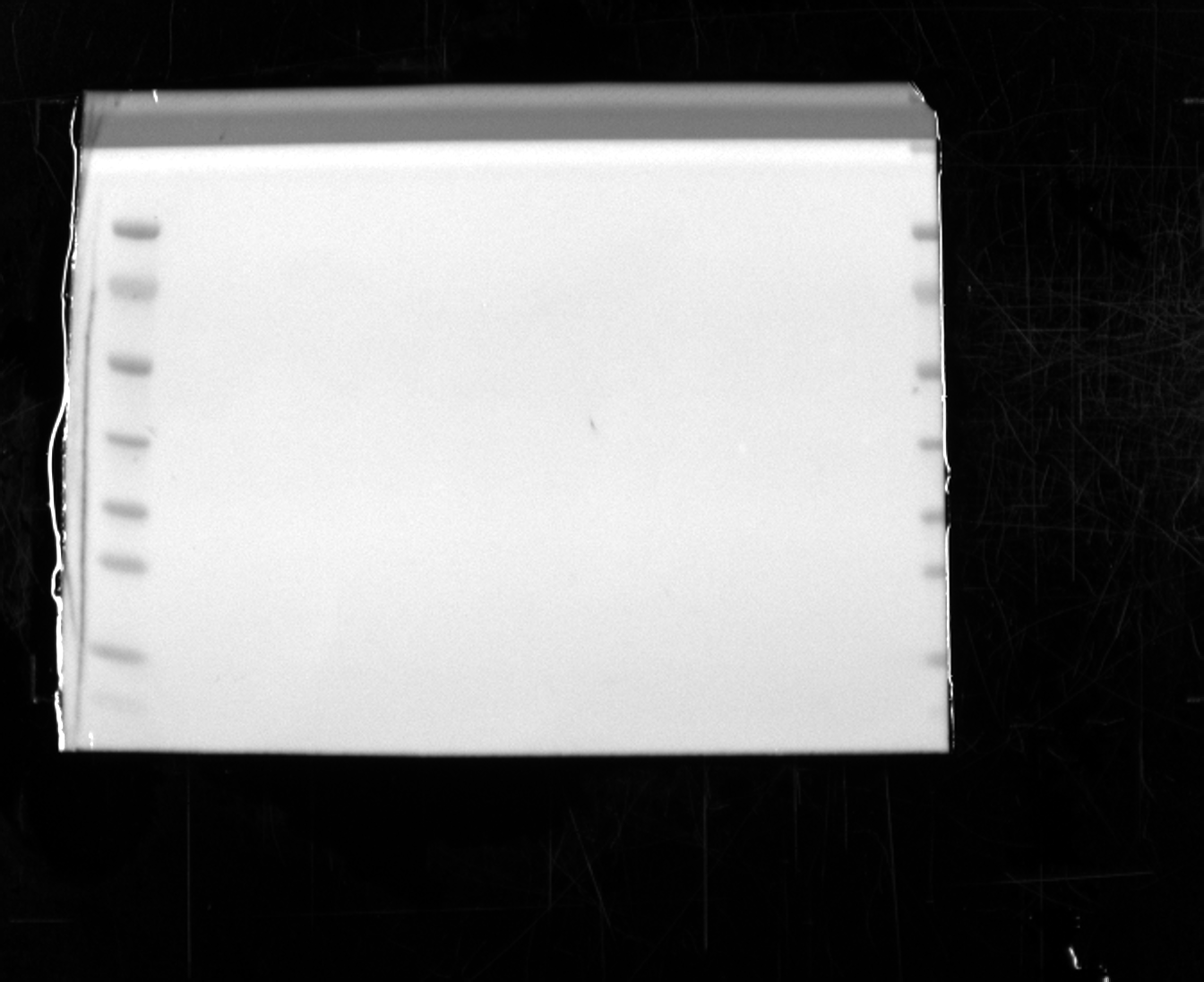

Supplement: Supplementary file 2 — Supporting Information [file ADVS-12-e06225-s001.zip › SNA-Lectin/CD75-H H1 H2 H H1 H2 H H1 H2-W.Tif]

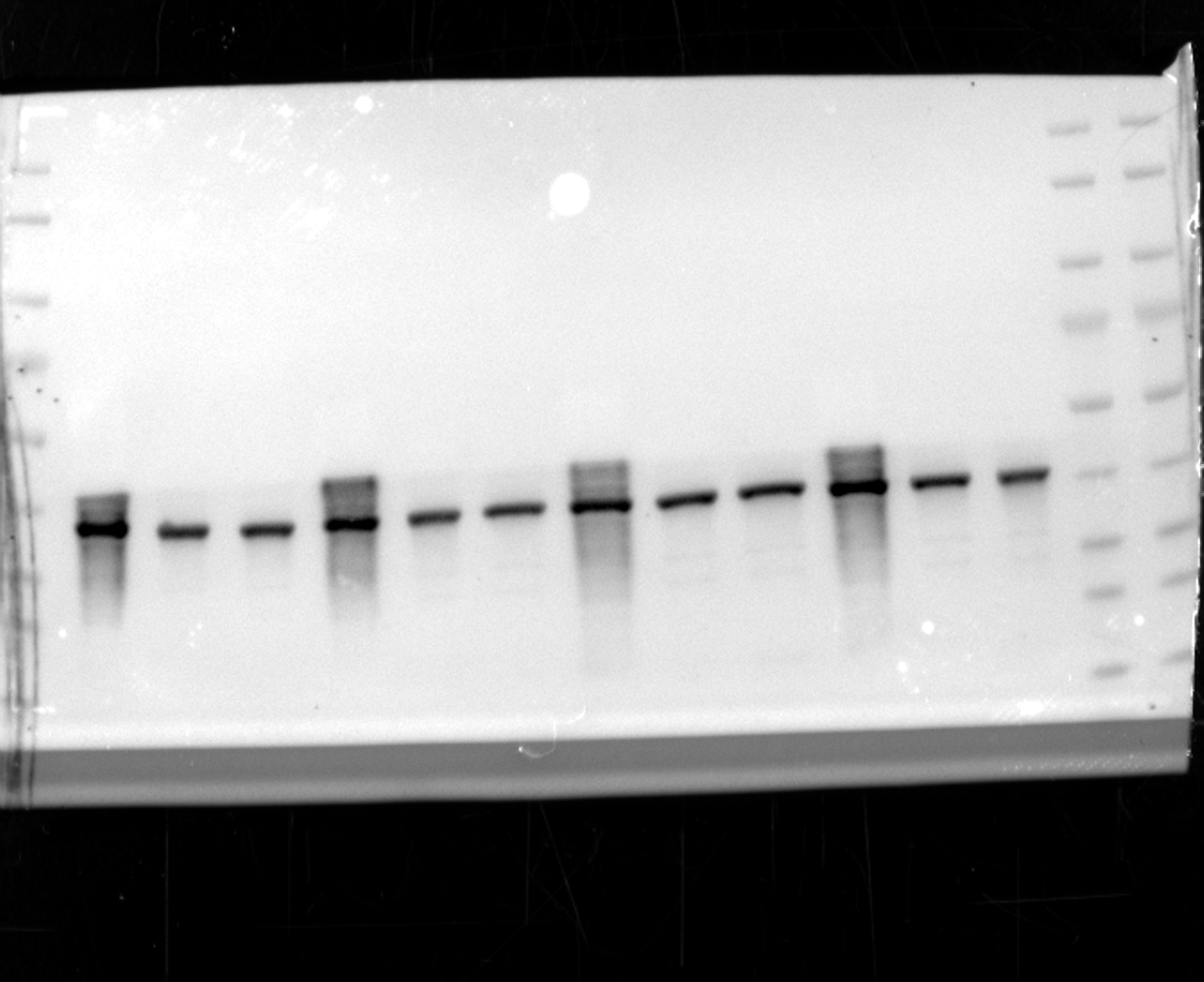

Supplement: Supplementary file 2 — Supporting Information [file ADVS-12-e06225-s001.zip › SNA-Lectin/GAP-CD75-116OE 116WT 116WT TQ TQ TQ -M.Tif]

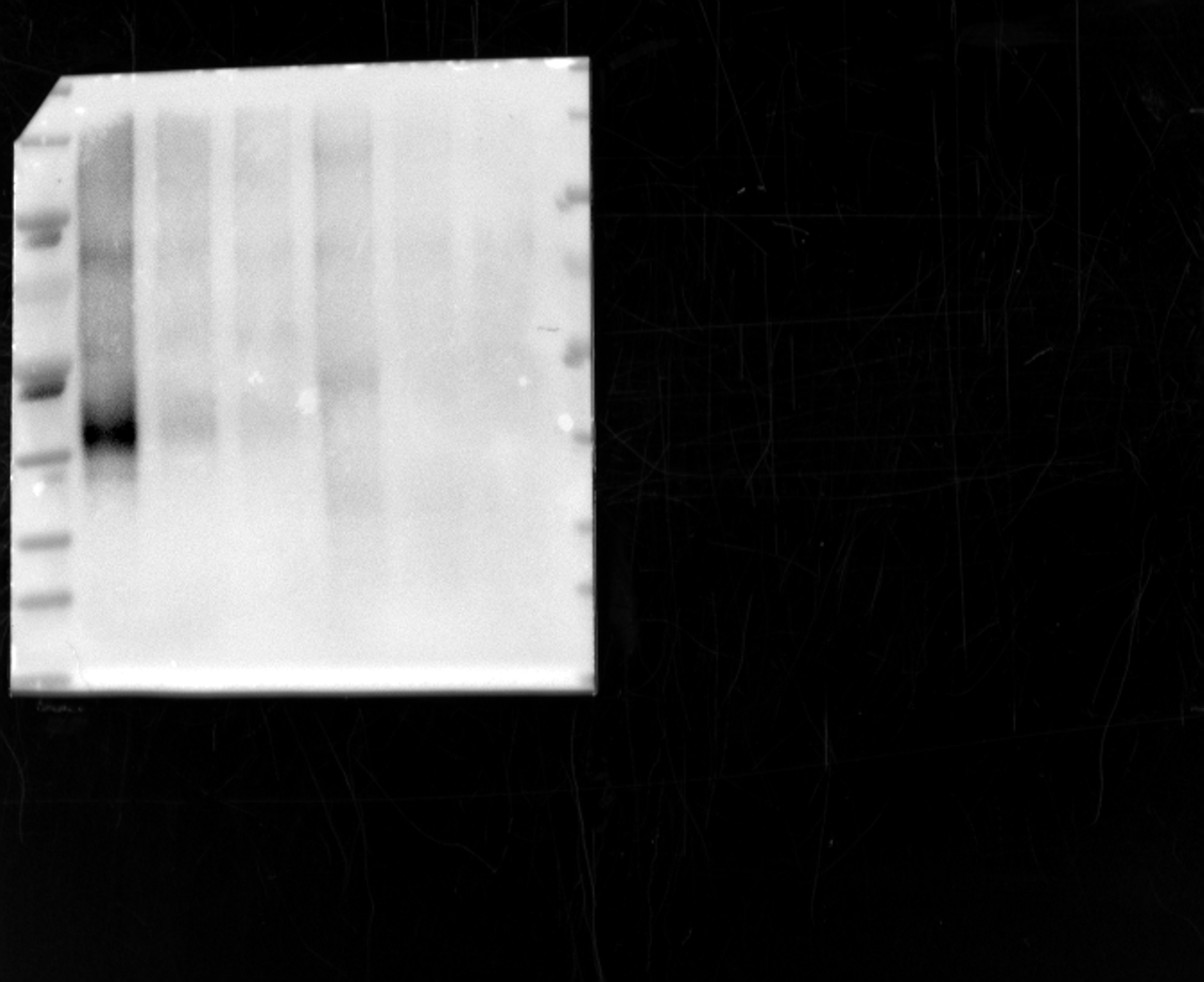

Supplement: Supplementary file 2 — Supporting Information [file ADVS-12-e06225-s001.zip › SNA-Lectin/SNA-L- H H1 H2--M.Tif]

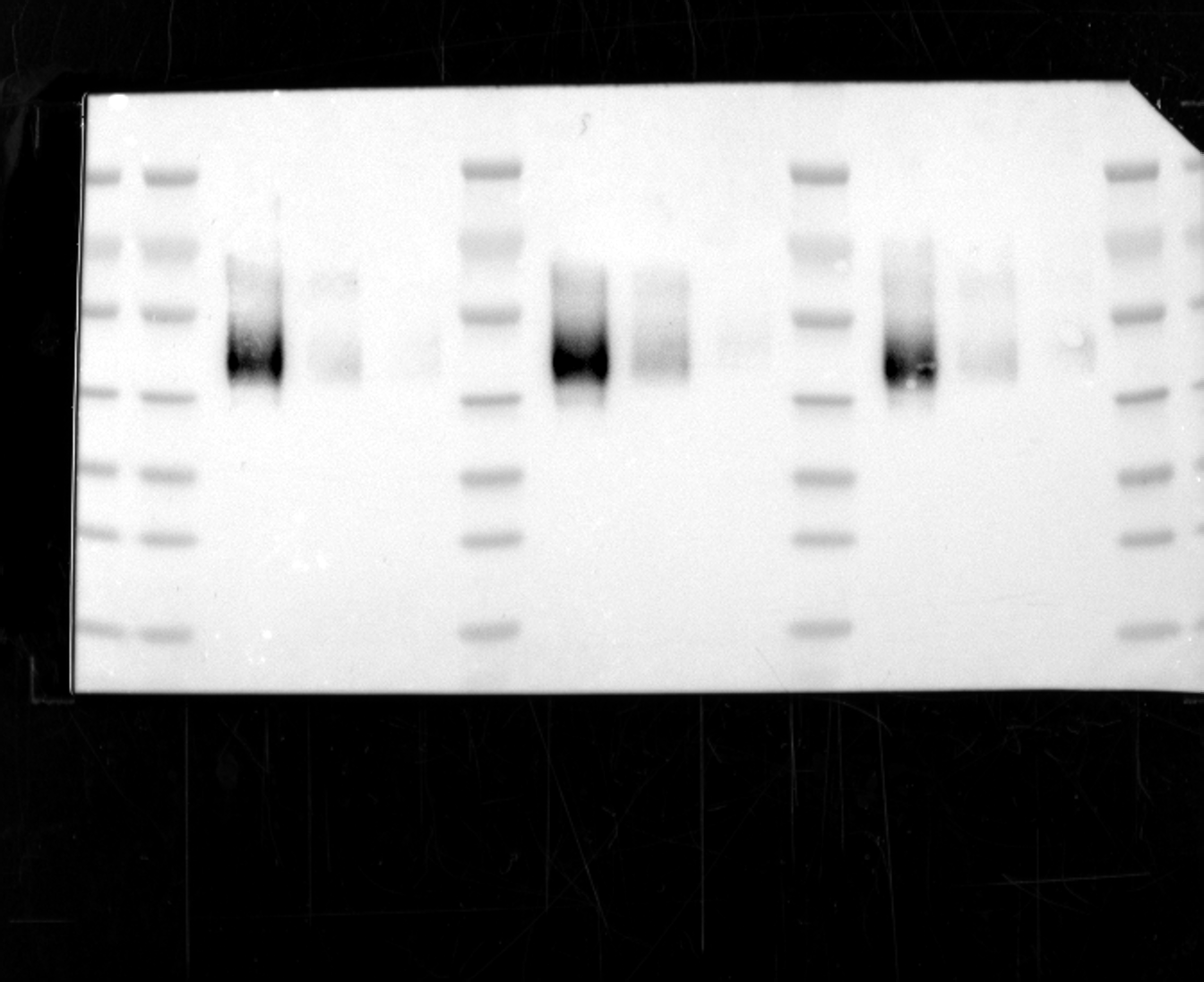

Supplement: Supplementary file 2 — Supporting Information [file ADVS-12-e06225-s001.zip › SNA-Lectin/SNA-L-116WT 116SH1 116SH2 M 116WT 116SH1 116SH2 M 116WT 116SH1 116SH2-----4S.Tif]

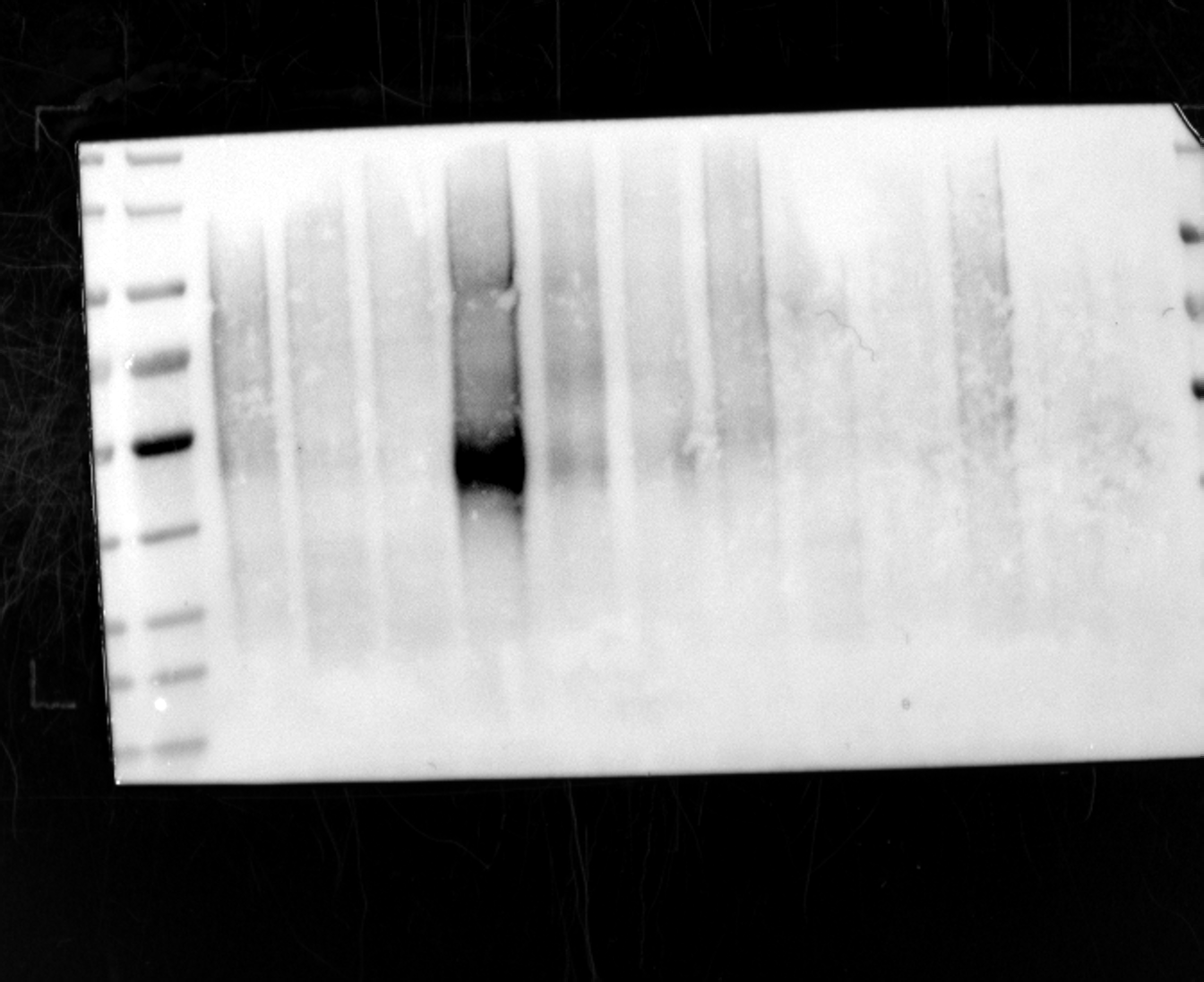

Supplement: Supplementary file 2 — Supporting Information [file ADVS-12-e06225-s001.zip › SNA-Lectin/SNA-L-H H1 H2 116WT 116SH1 116SH2 H H1 H2 H H1 H2-M.Tif]

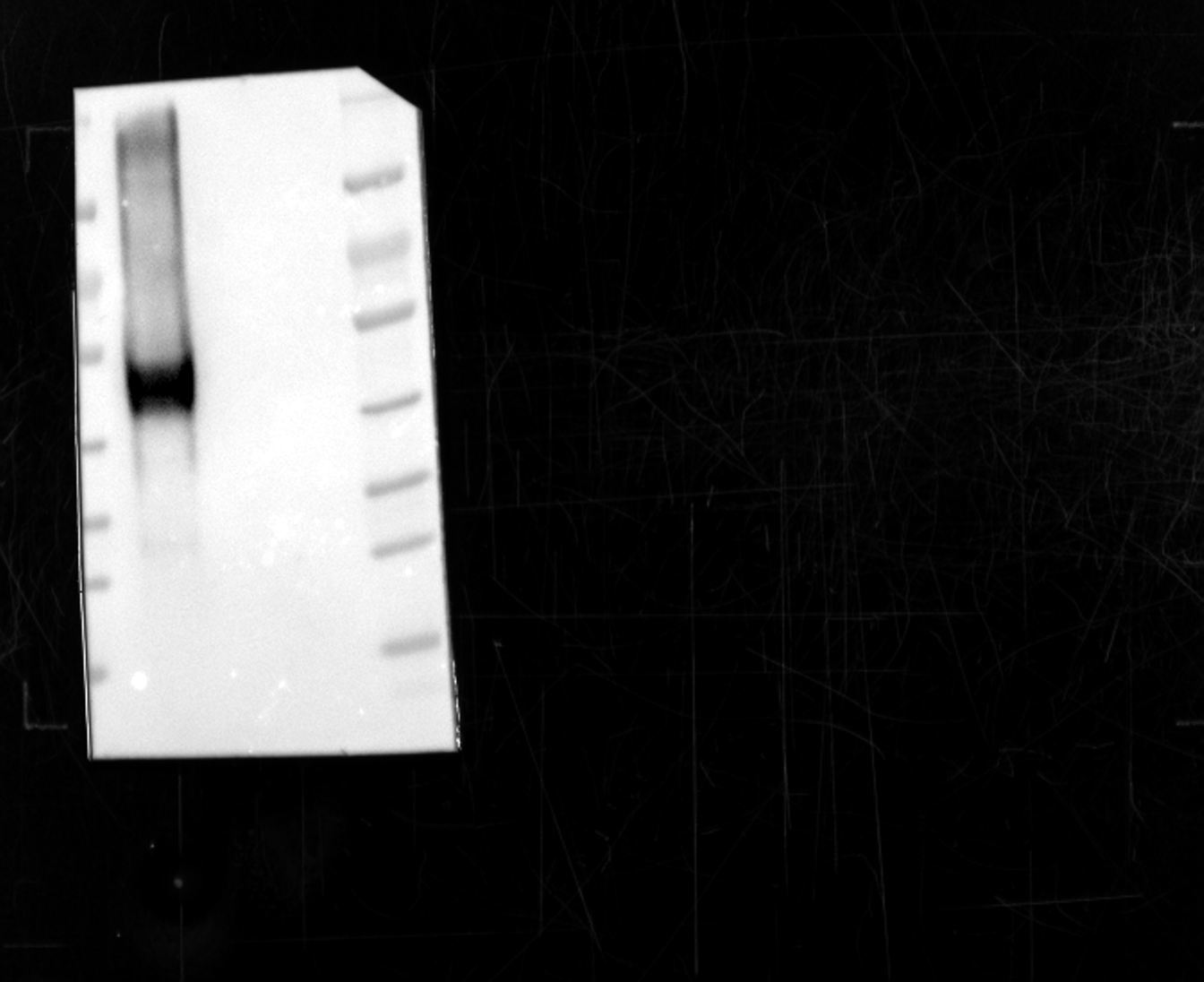

Supplement: Supplementary file 2 — Supporting Information [file ADVS-12-e06225-s001.zip › SNA-Lectin/SNA-L-HWT H1 H2-4M.Tif]

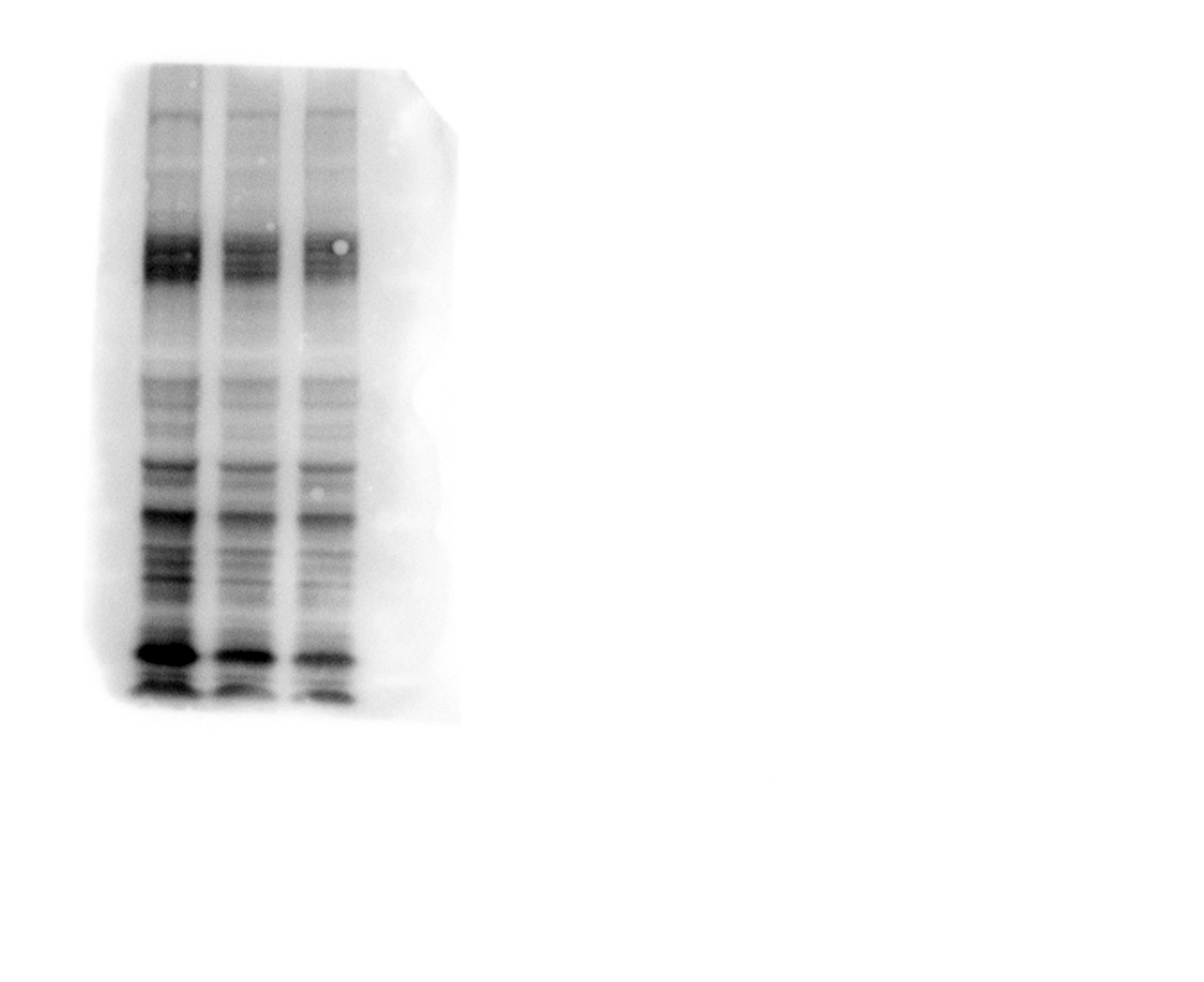

Supplement: Supplementary file 2 — Supporting Information [file ADVS-12-e06225-s001.zip › SNA-WB/1-SNA-H H1 H2-2S-7.Tif]

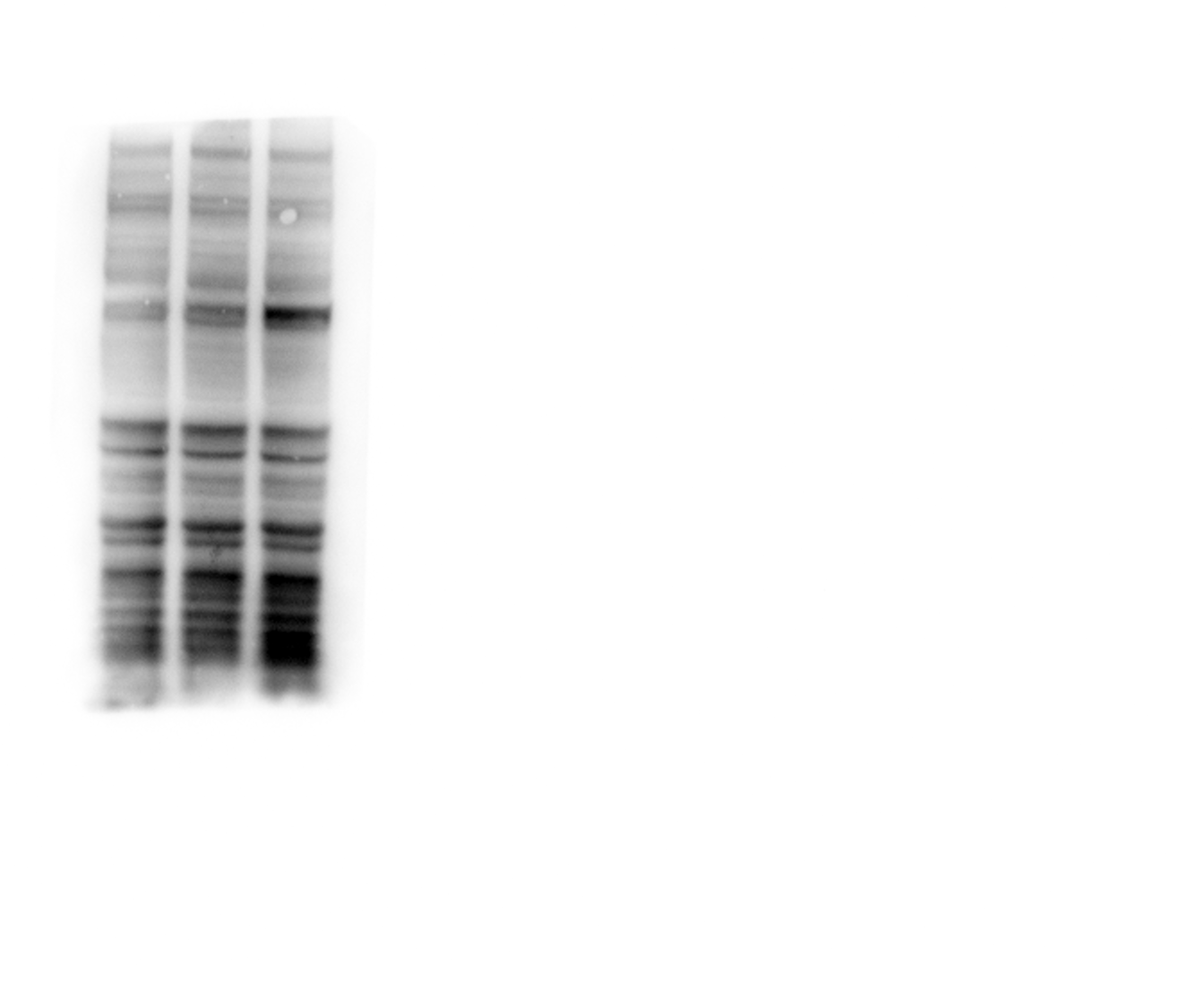

Supplement: Supplementary file 2 — Supporting Information [file ADVS-12-e06225-s001.zip › SNA-WB/2-SNA-116-1 116-2 116WT-1S--1.Tif]

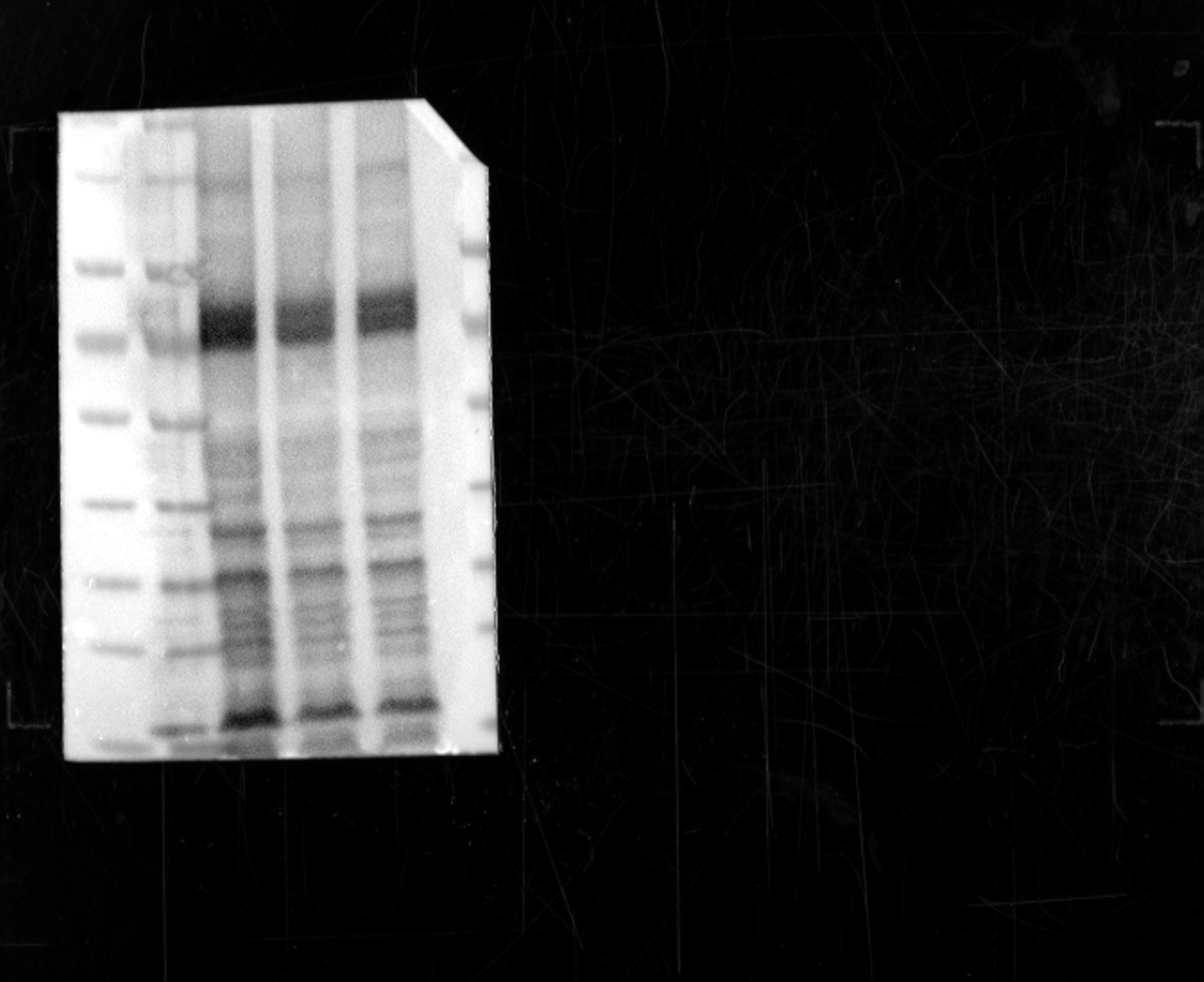

Supplement: Supplementary file 2 — Supporting Information [file ADVS-12-e06225-s001.zip › SNA-WB/2-SNA-H H1 H2-M.Tif]

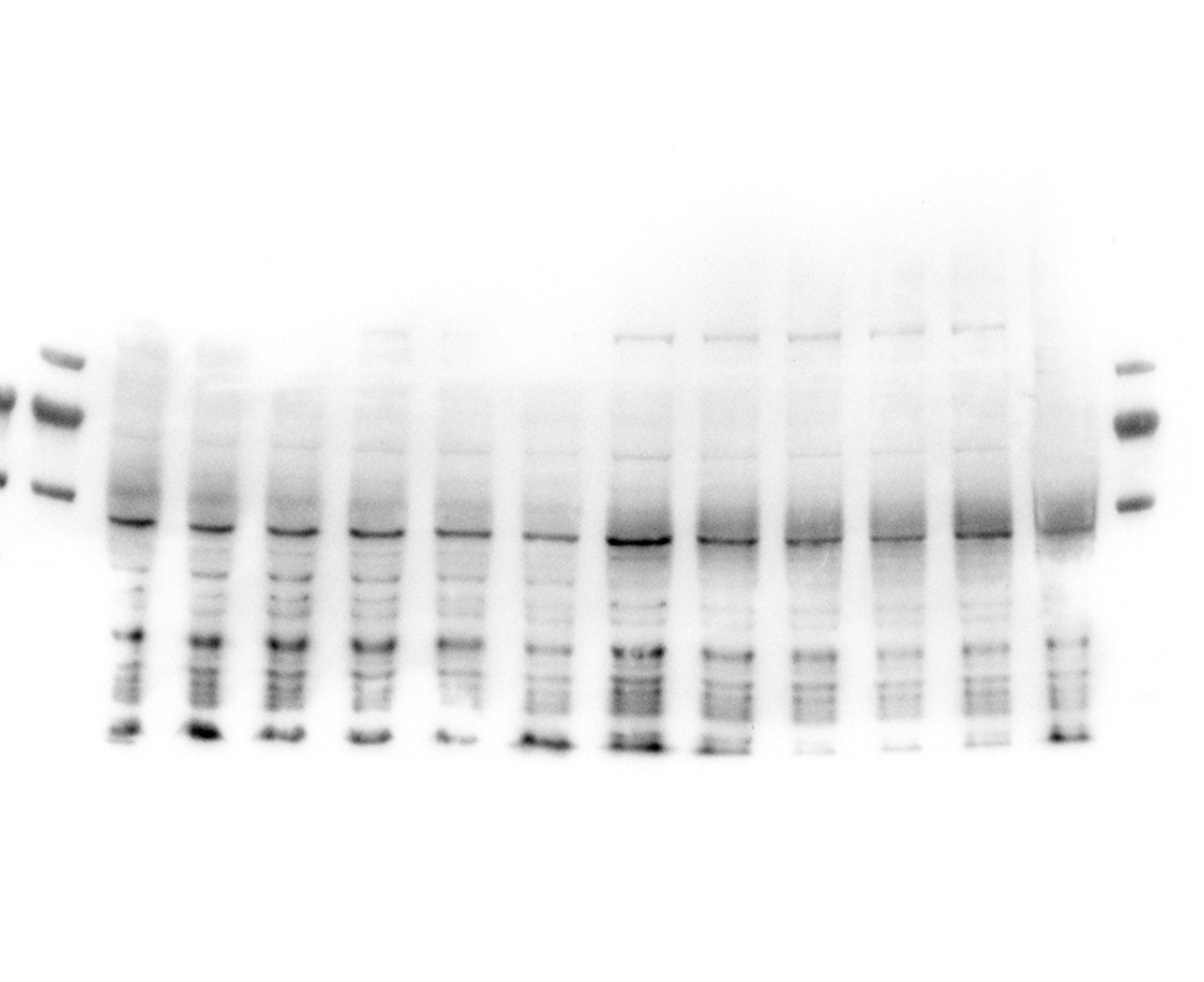

Supplement: Supplementary file 2 — Supporting Information [file ADVS-12-e06225-s001.zip › SNA-WB/CHX-H1 116WT-1S.Tif]

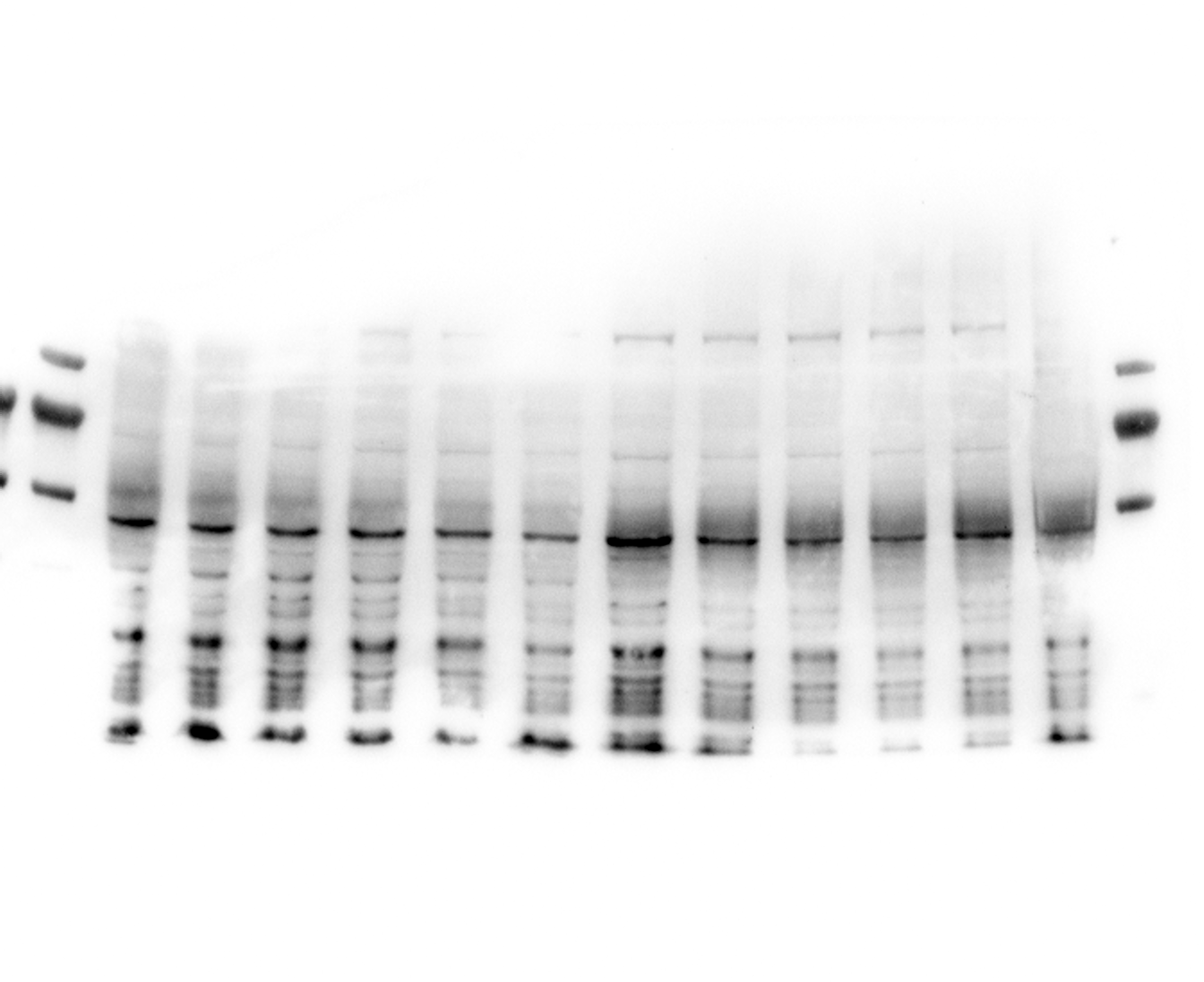

Supplement: Supplementary file 2 — Supporting Information [file ADVS-12-e06225-s001.zip › SNA-WB/CHX-H1 116WT-2S.Tif]

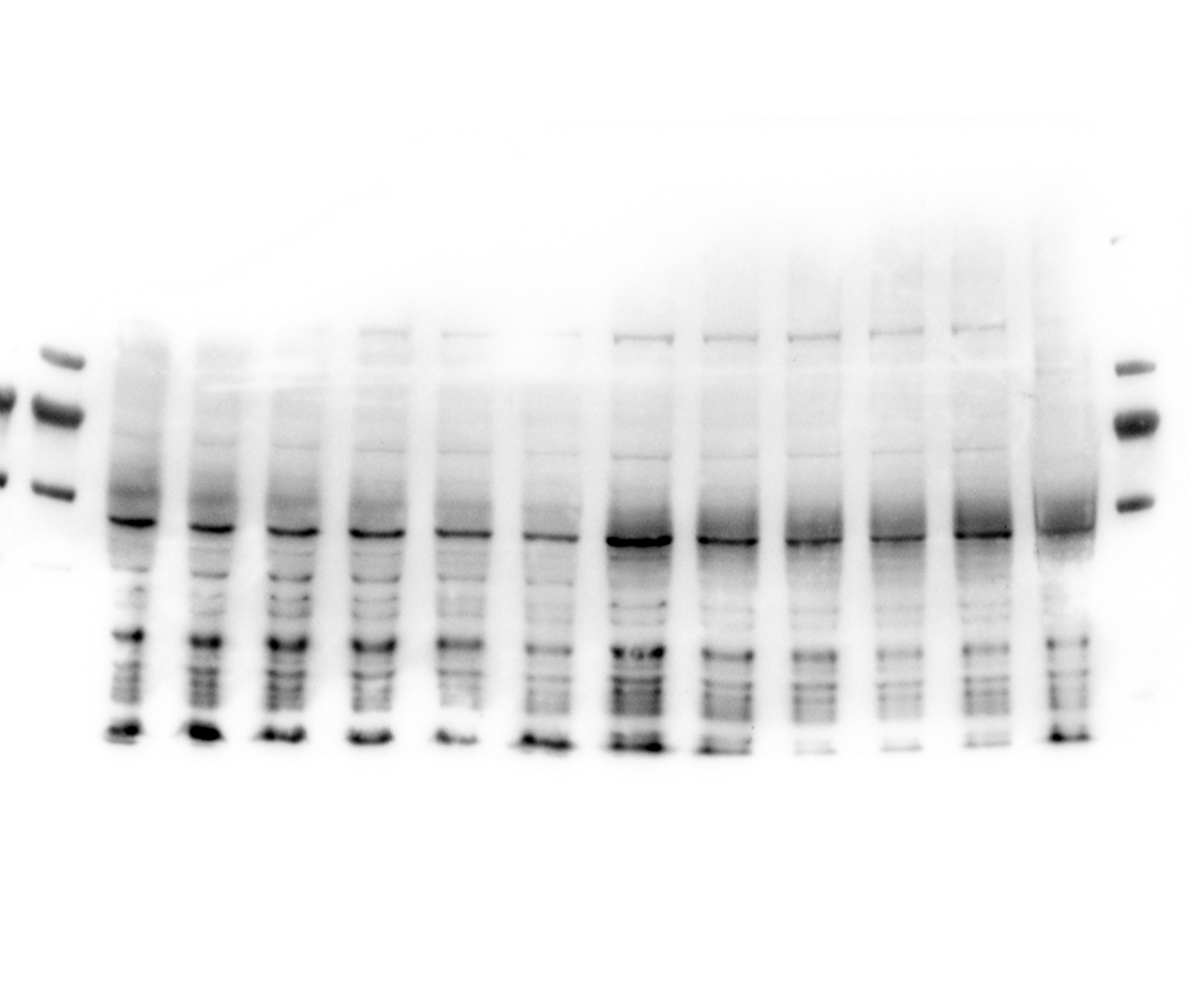

Supplement: Supplementary file 2 — Supporting Information [file ADVS-12-e06225-s001.zip › SNA-WB/CHX-H1 116WT-3S.Tif]

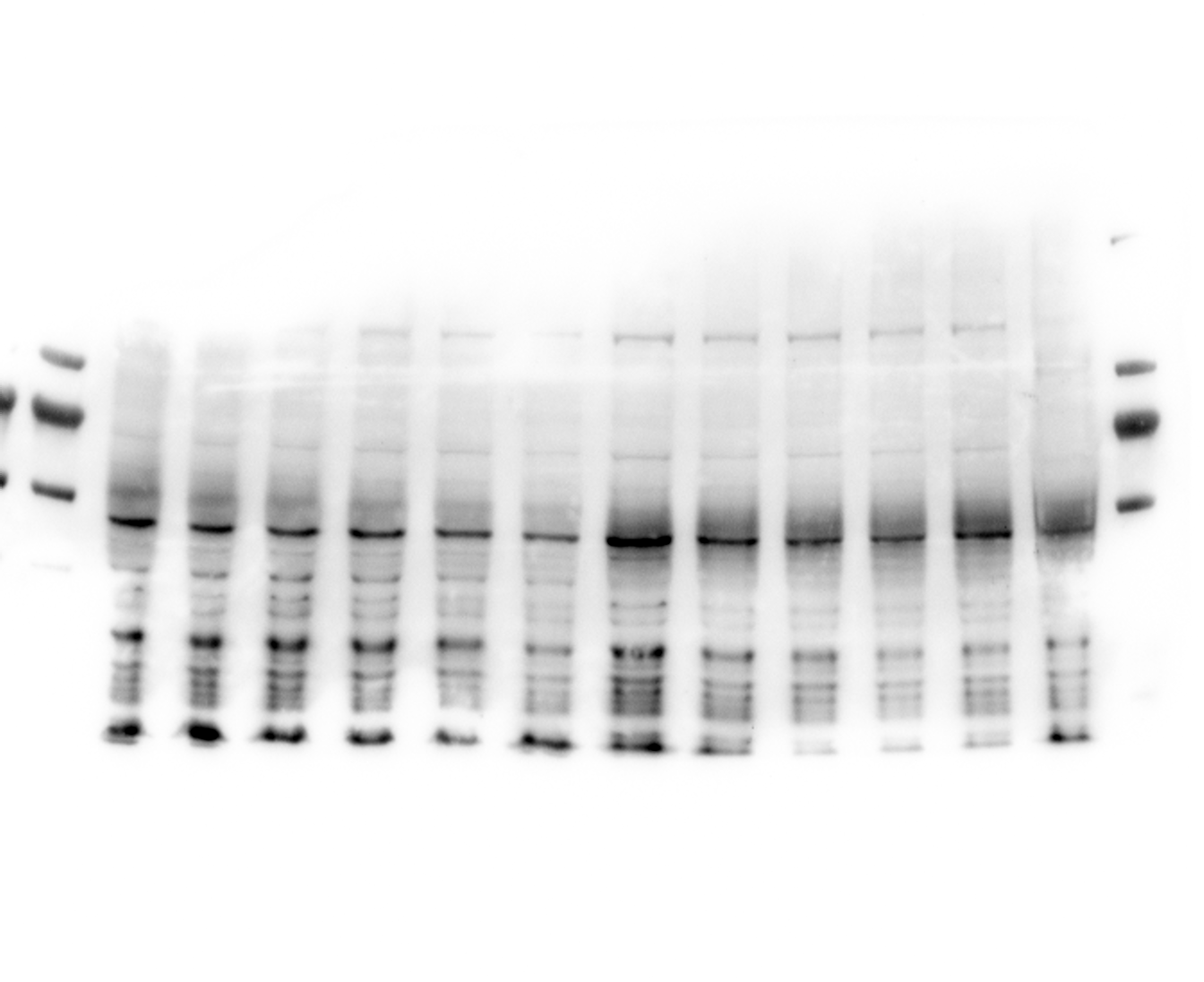

Supplement: Supplementary file 2 — Supporting Information [file ADVS-12-e06225-s001.zip › SNA-WB/CHX-H1 116WT-4S.Tif]

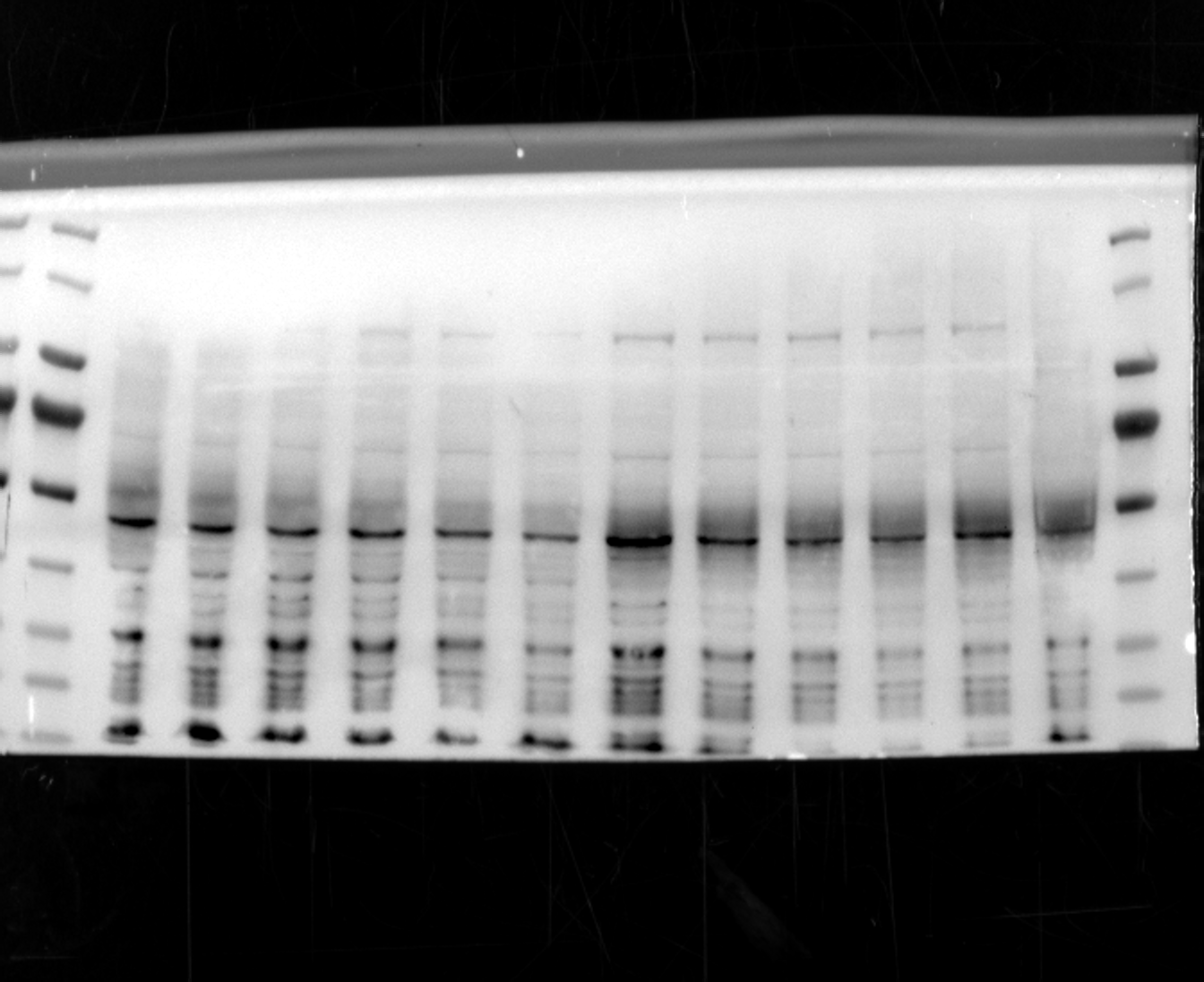

Supplement: Supplementary file 2 — Supporting Information [file ADVS-12-e06225-s001.zip › SNA-WB/CHX-H1 116WT-M.Tif]

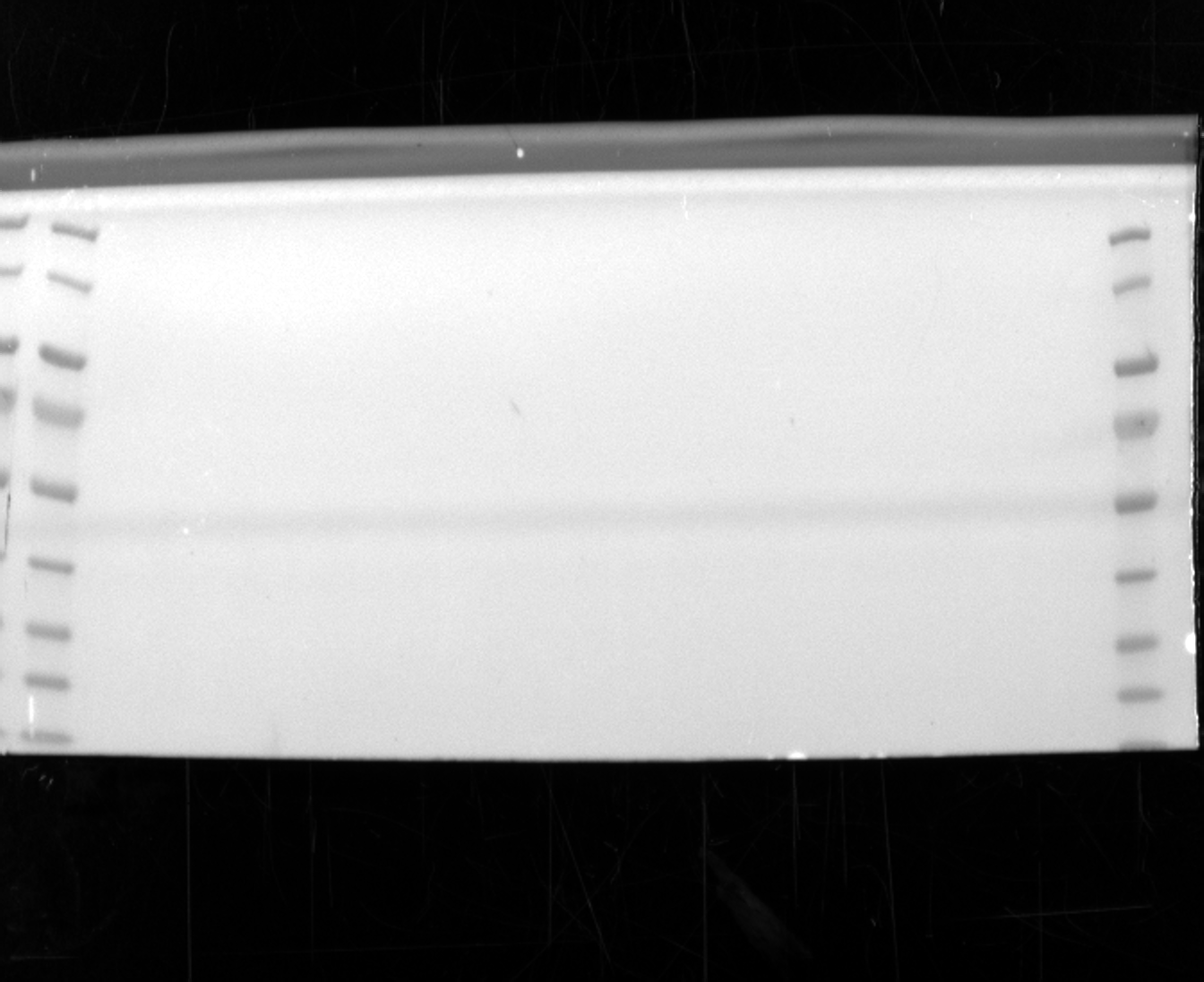

Supplement: Supplementary file 2 — Supporting Information [file ADVS-12-e06225-s001.zip › SNA-WB/CHX-H1 116WT-W.Tif]

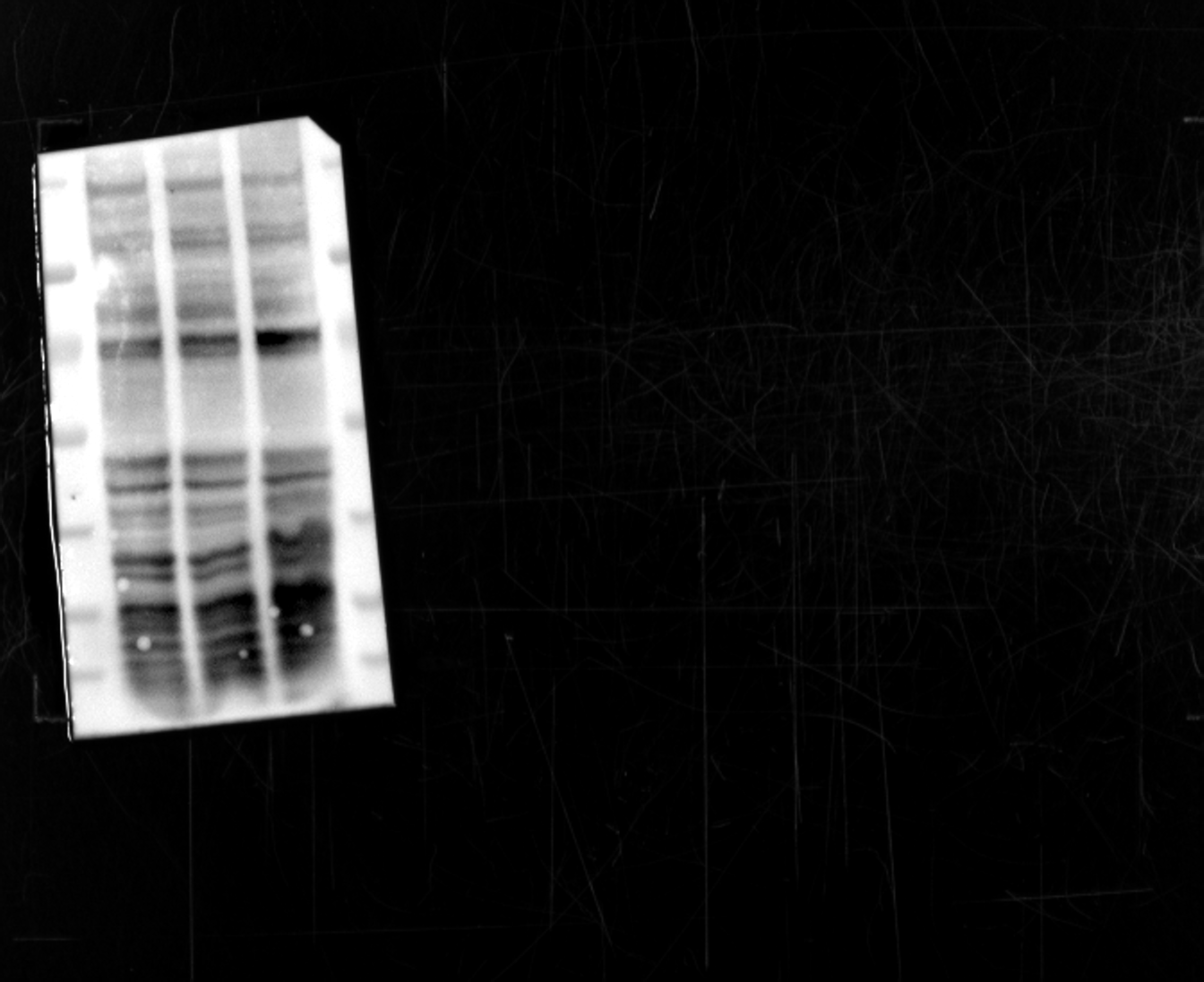

Supplement: Supplementary file 2 — Supporting Information [file ADVS-12-e06225-s001.zip › SNA-WB/SNA-116-1 116-2 116WT-M.Tif]

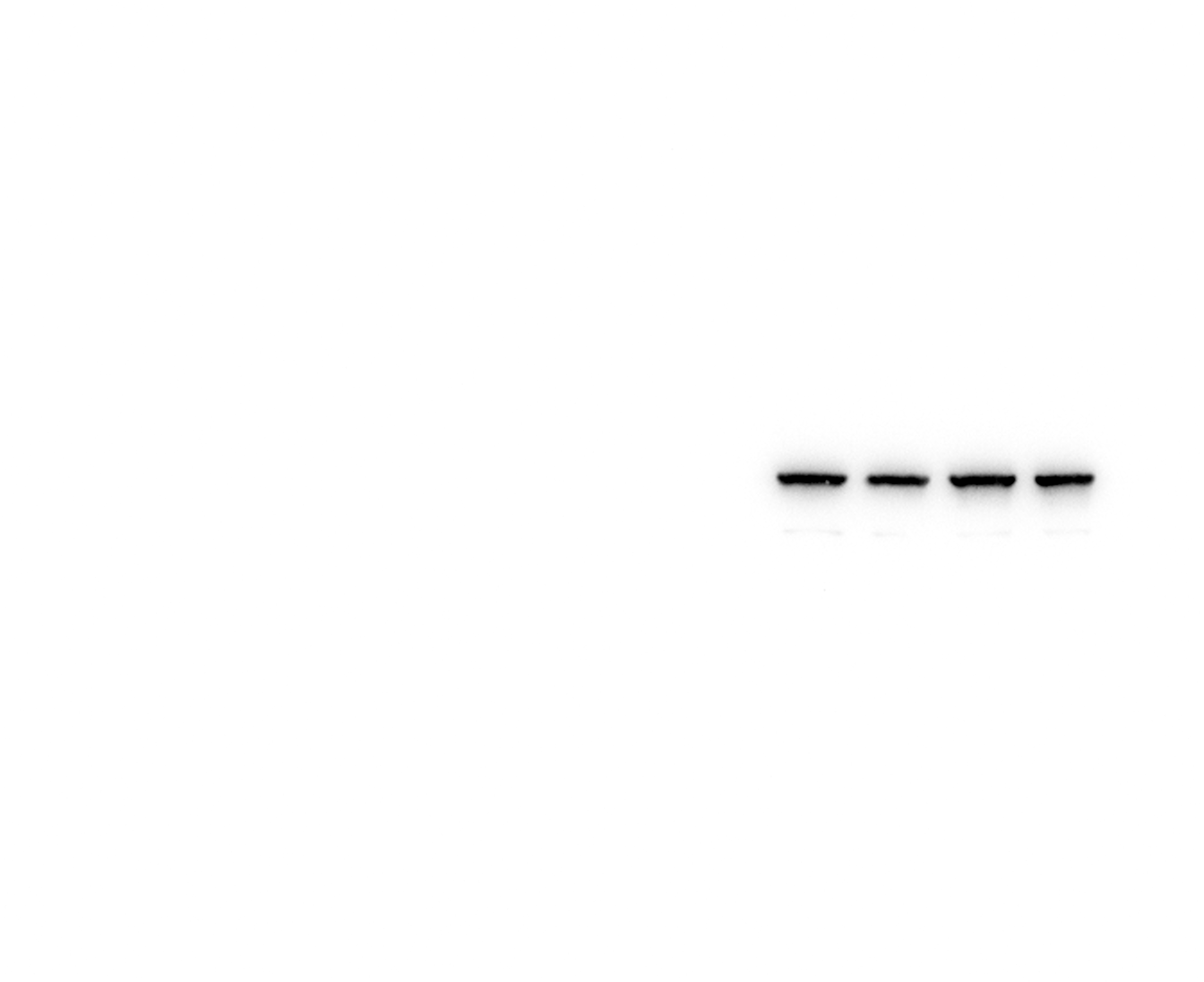

Supplement: Supplementary file 2 — Supporting Information [file ADVS-12-e06225-s001.zip › SNA-WB/SNA-L---GAP-116OE 116WT 116OE 116WT-1S-1.Tif]

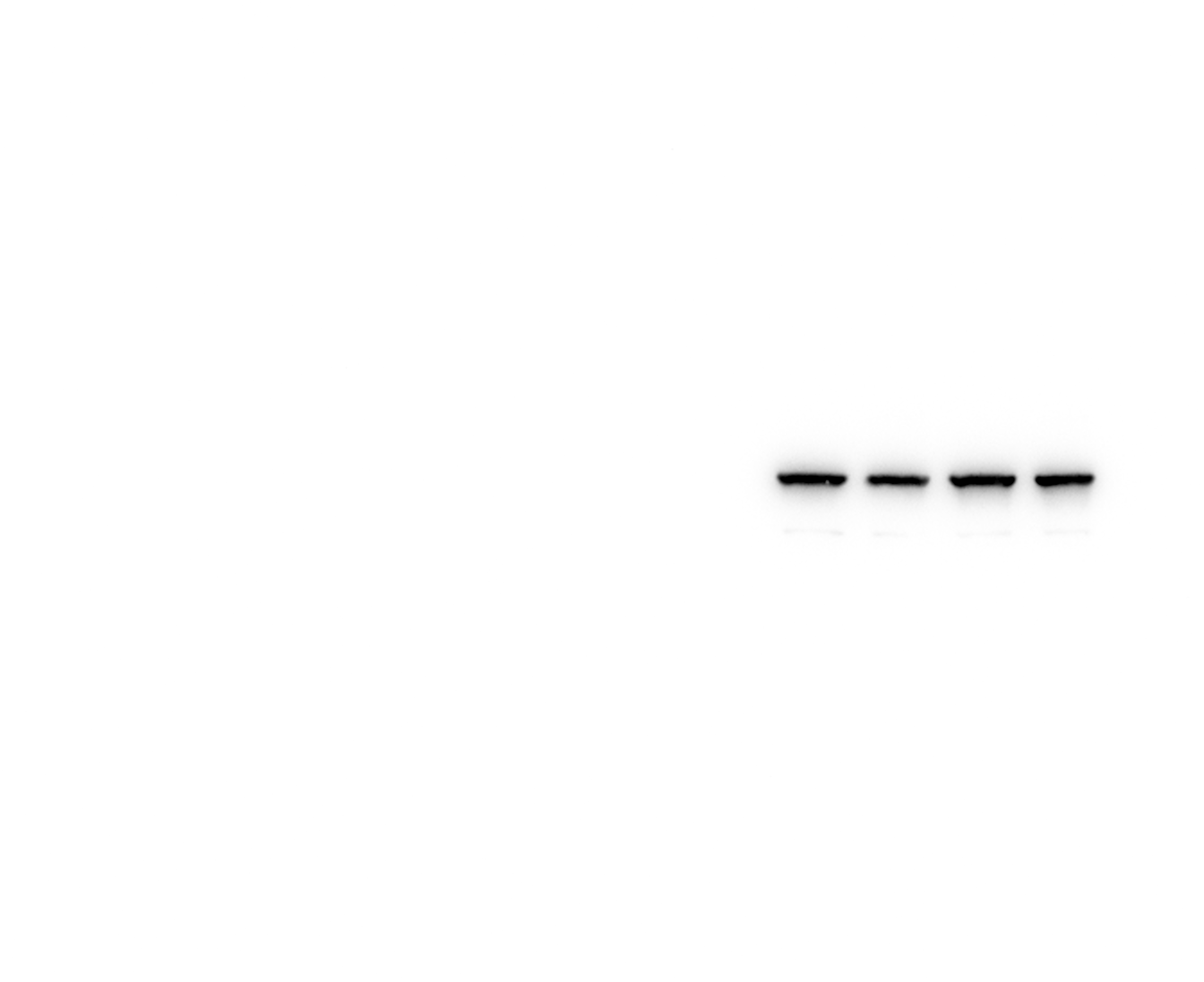

Supplement: Supplementary file 2 — Supporting Information [file ADVS-12-e06225-s001.zip › SNA-WB/SNA-L---GAP-116OE 116WT 116OE 116WT-2S.Tif]

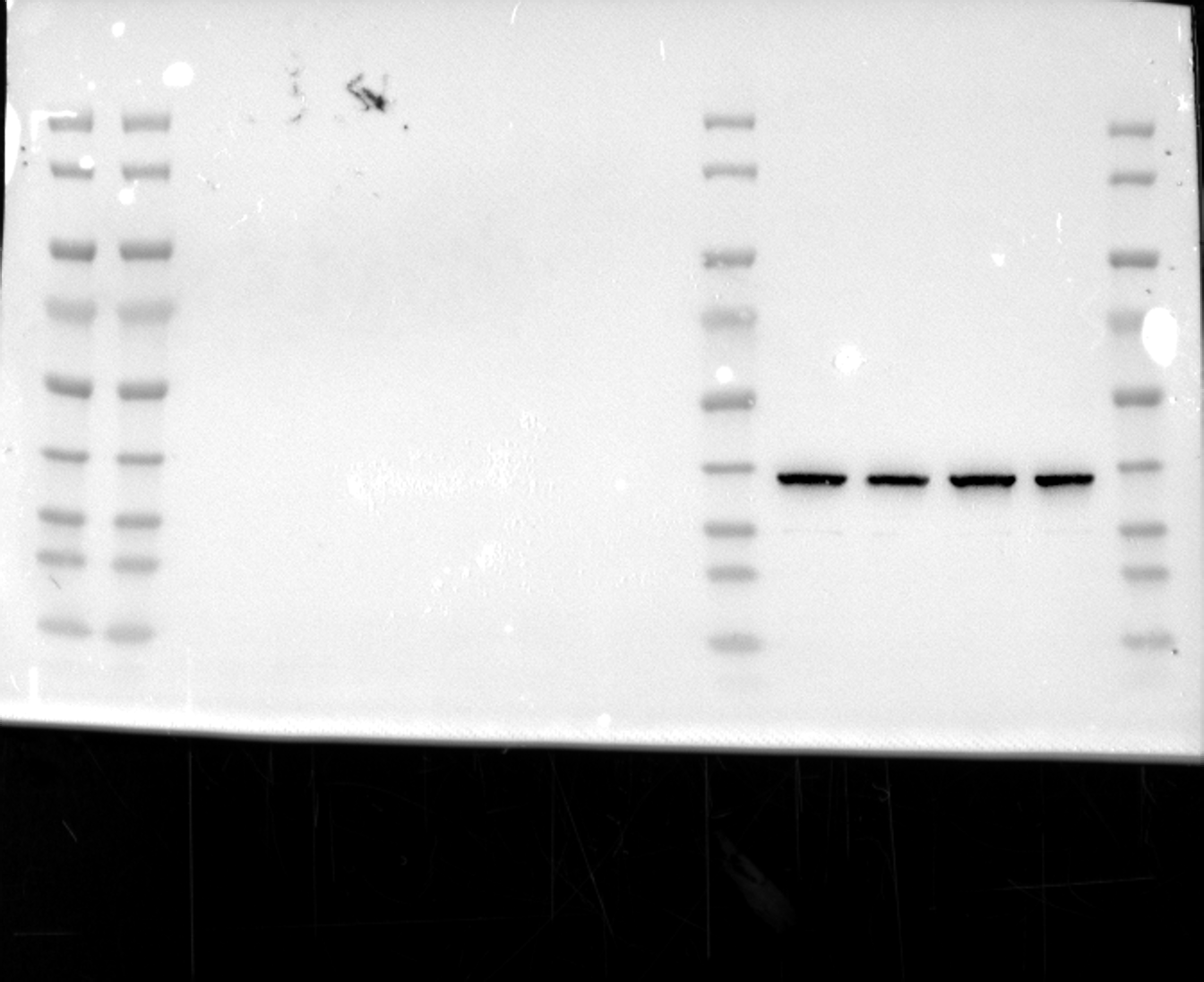

Supplement: Supplementary file 2 — Supporting Information [file ADVS-12-e06225-s001.zip › SNA-WB/SNA-L---GAP-116OE 116WT 116OE 116WT-M.Tif]

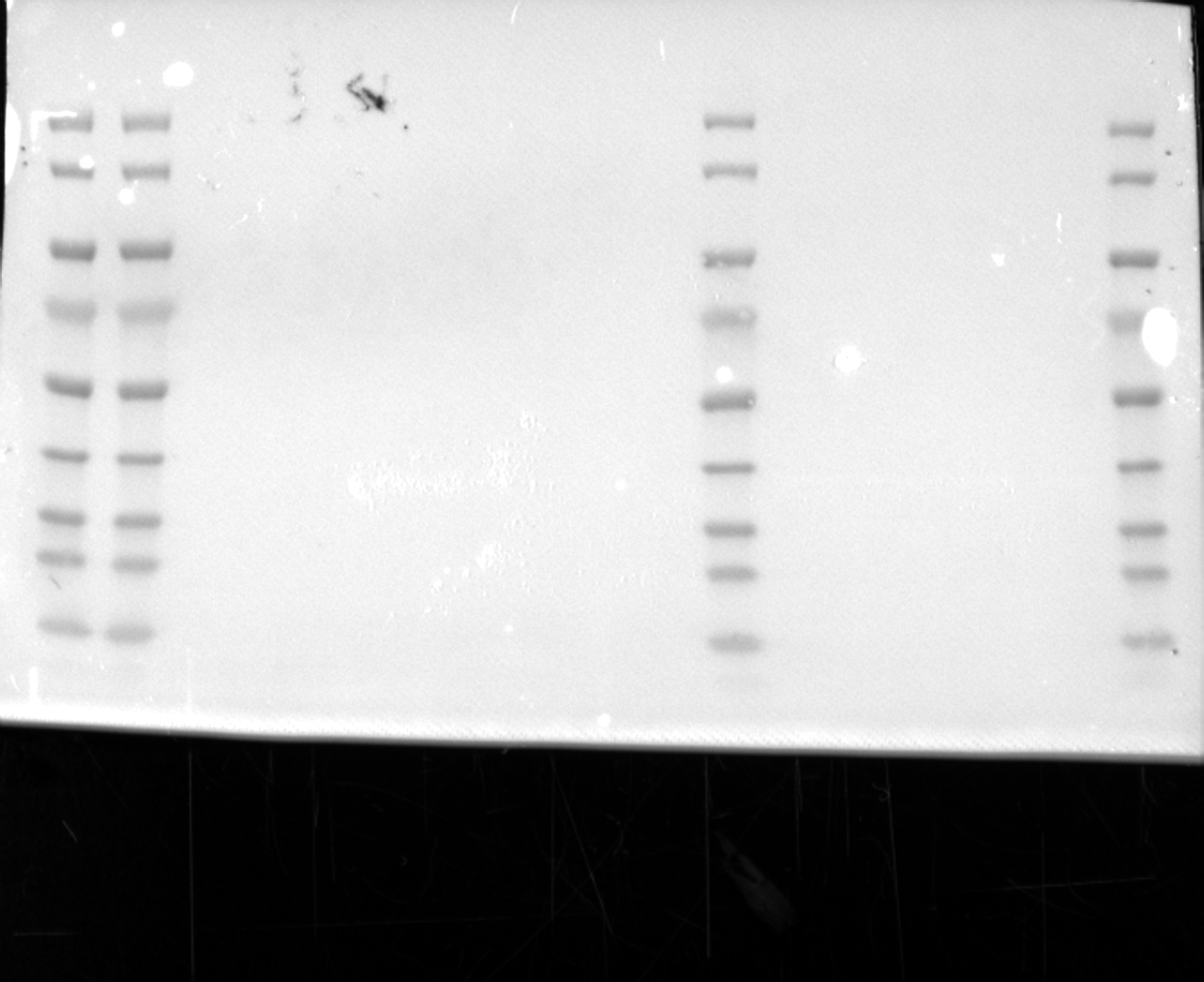

Supplement: Supplementary file 2 — Supporting Information [file ADVS-12-e06225-s001.zip › SNA-WB/SNA-L---GAP-116OE 116WT 116OE 116WT-W.Tif]

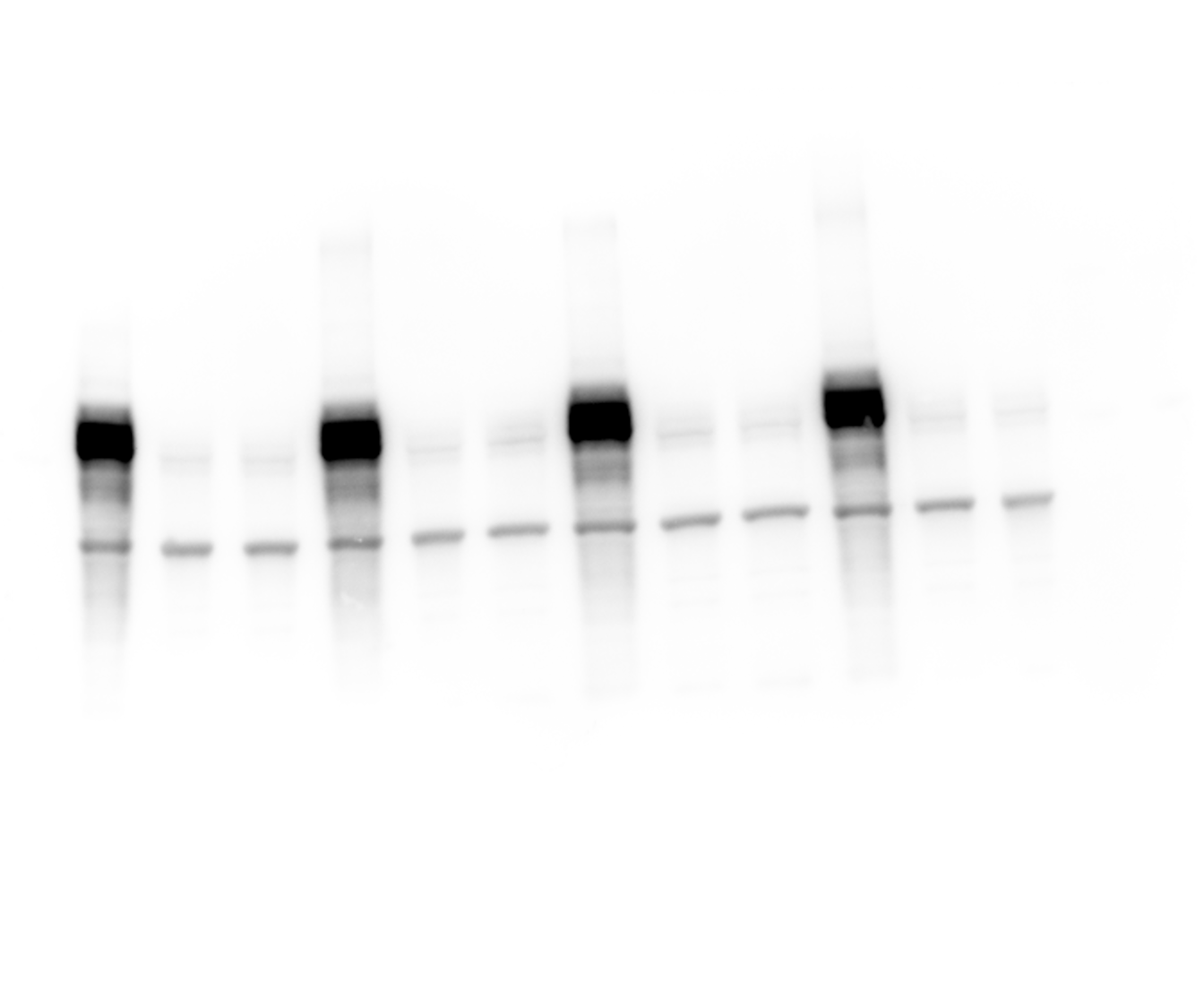

Supplement: Supplementary file 2 — Supporting Information [file ADVS-12-e06225-s001.zip › CD75/CD75-116OE 116m 116WT TQ TQ TQ -2S-1.Tif]

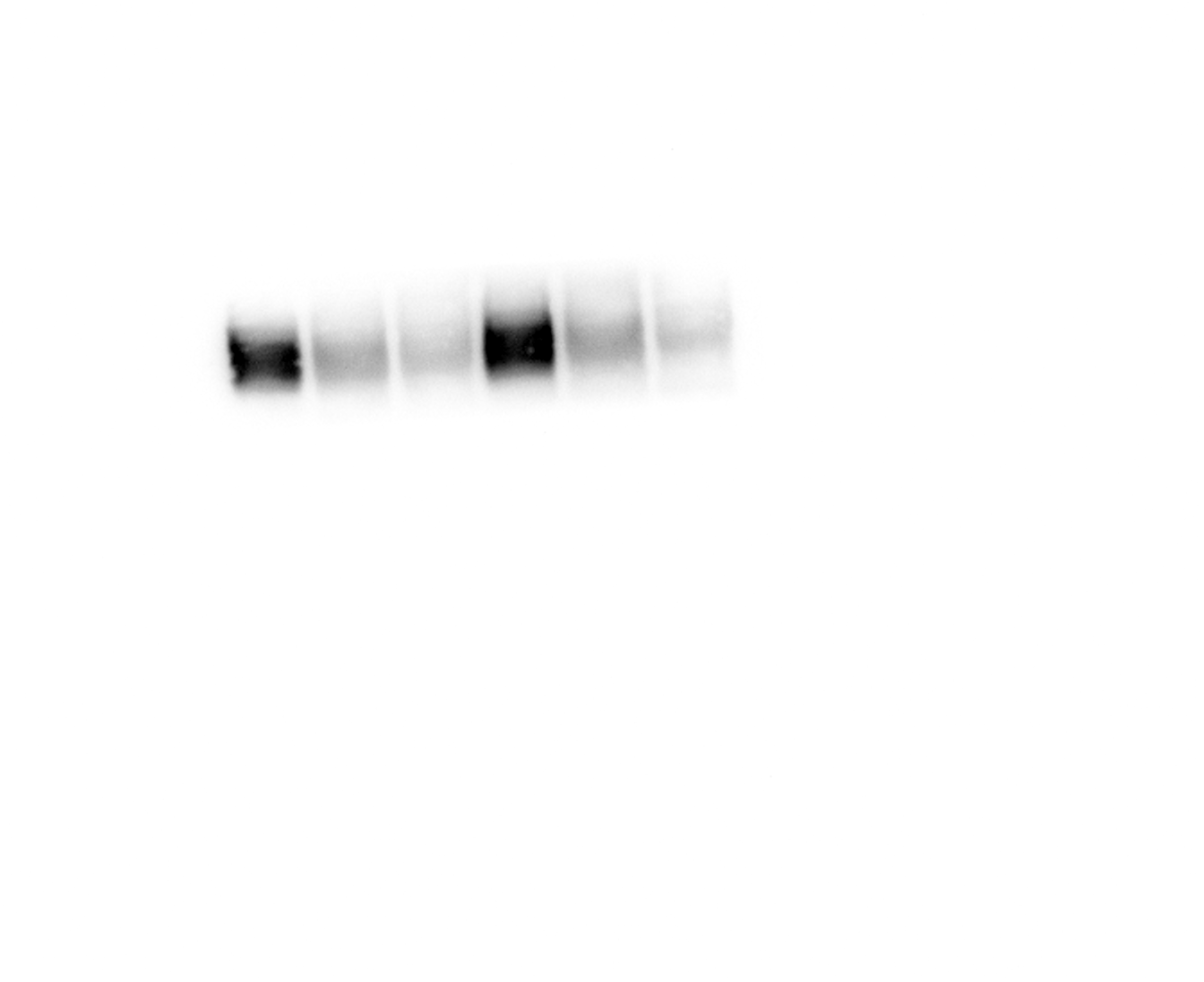

Supplement: Supplementary file 2 — Supporting Information [file ADVS-12-e06225-s001.zip › CD75/CD75-116WT 116SH1 116SH2 116WT 116SH1 116SH2-4S.Tif]

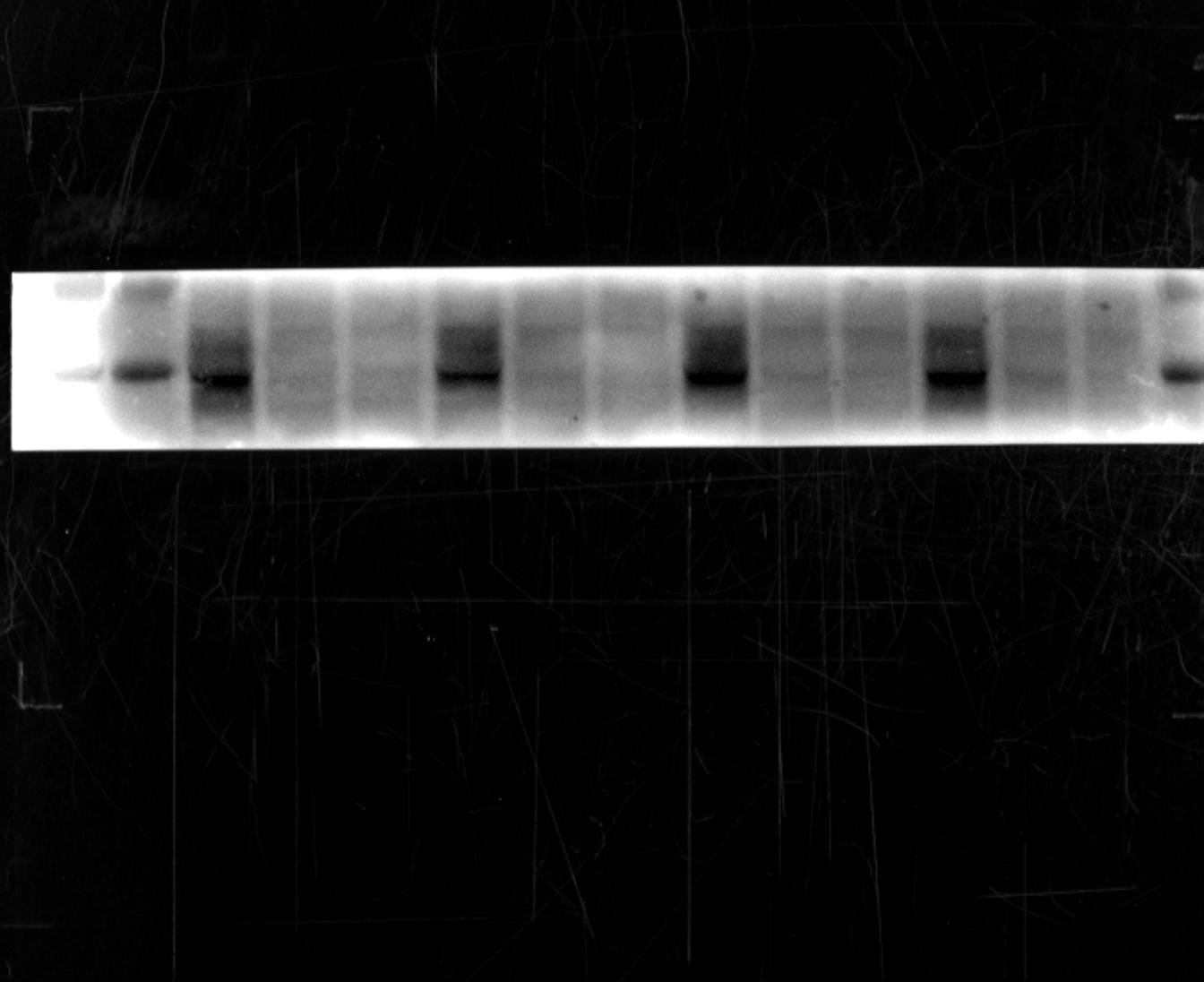

Supplement: Supplementary file 2 — Supporting Information [file ADVS-12-e06225-s001.zip › CD75/CD75-H H1 H2 H H1 H2 H H1 H2 H H1 H2-M.Tif]

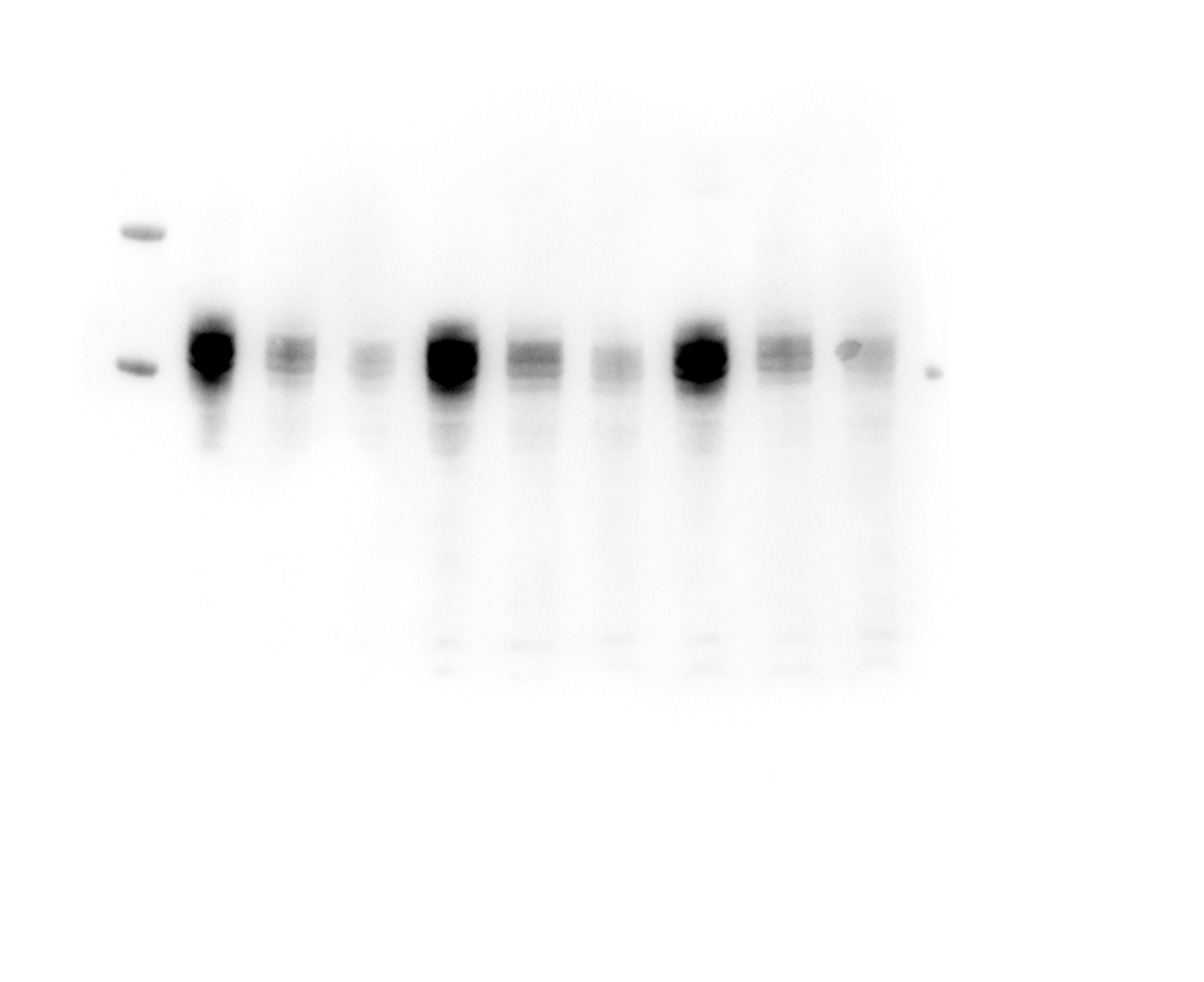

Supplement: Supplementary file 2 — Supporting Information [file ADVS-12-e06225-s001.zip › CD75/CD75-H H1 H2 H H1 H2 H H1 H2-5-1.Tif]

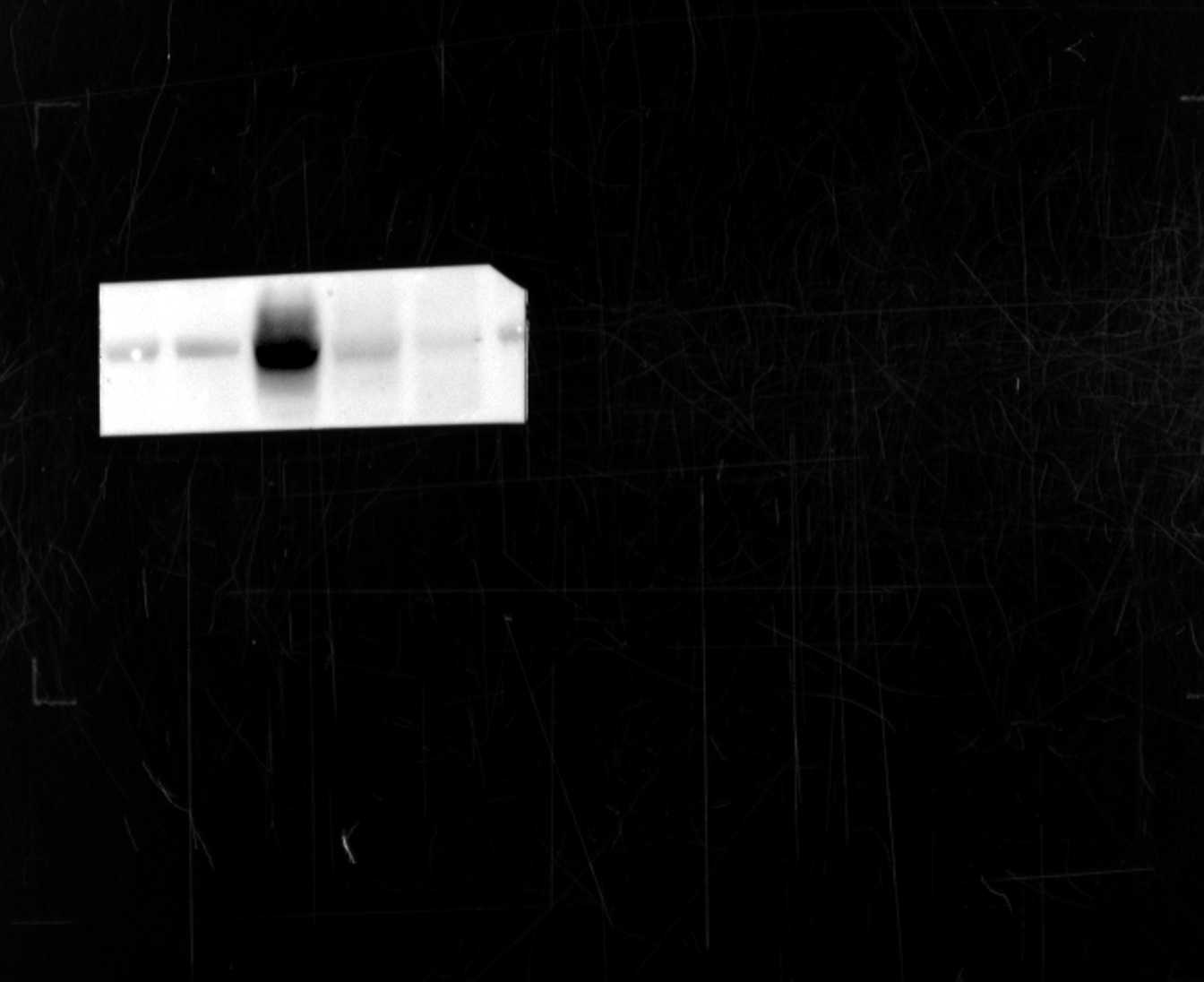

Supplement: Supplementary file 2 — Supporting Information [file ADVS-12-e06225-s001.zip › CD75/CD75-HWT H1 H2-M.Tif]

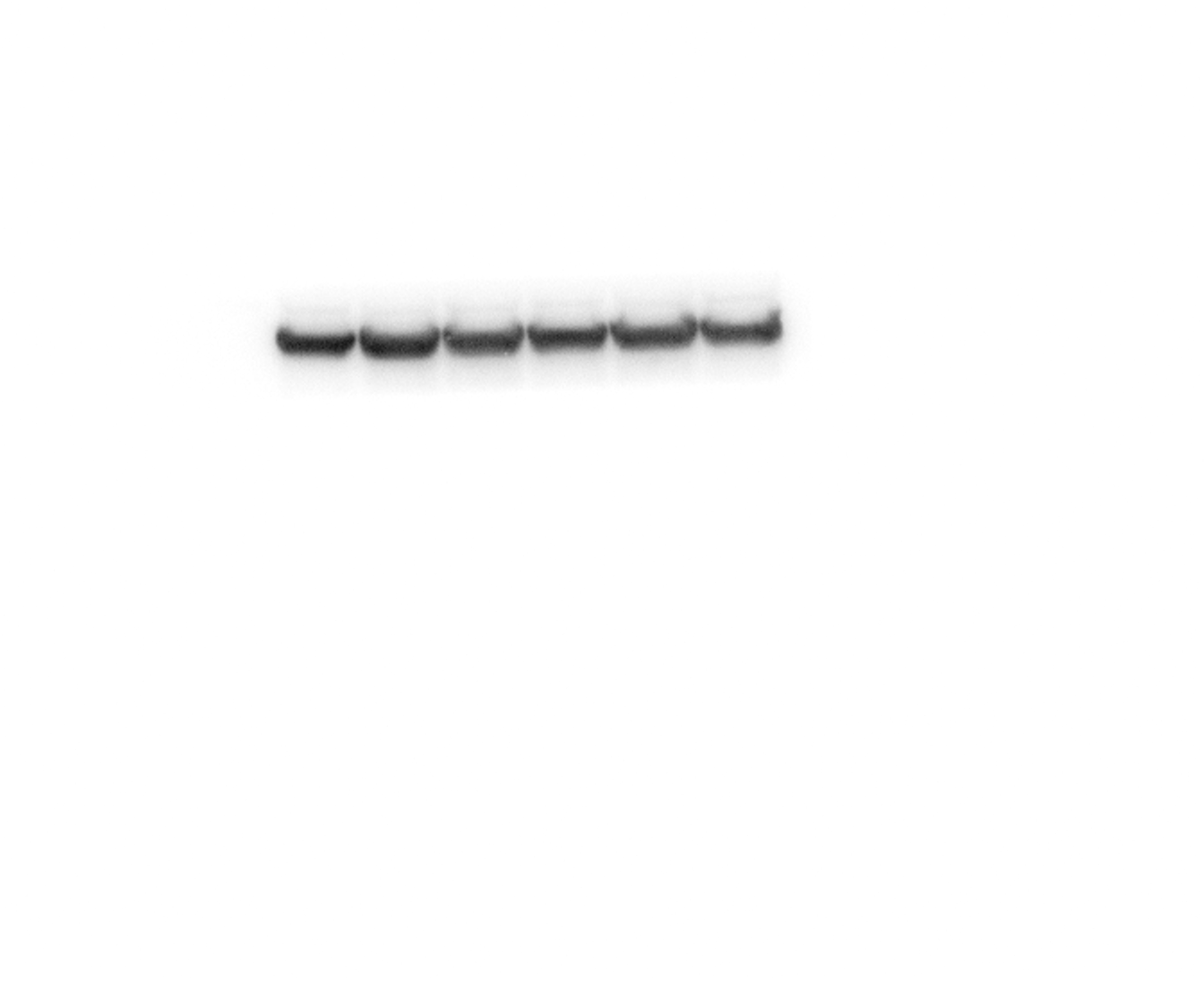

Supplement: Supplementary file 2 — Supporting Information [file ADVS-12-e06225-s001.zip › CD75/GAP-116WT 116SH1 116SH2 116WT 116SH1 116SH2-50MS.Tif]

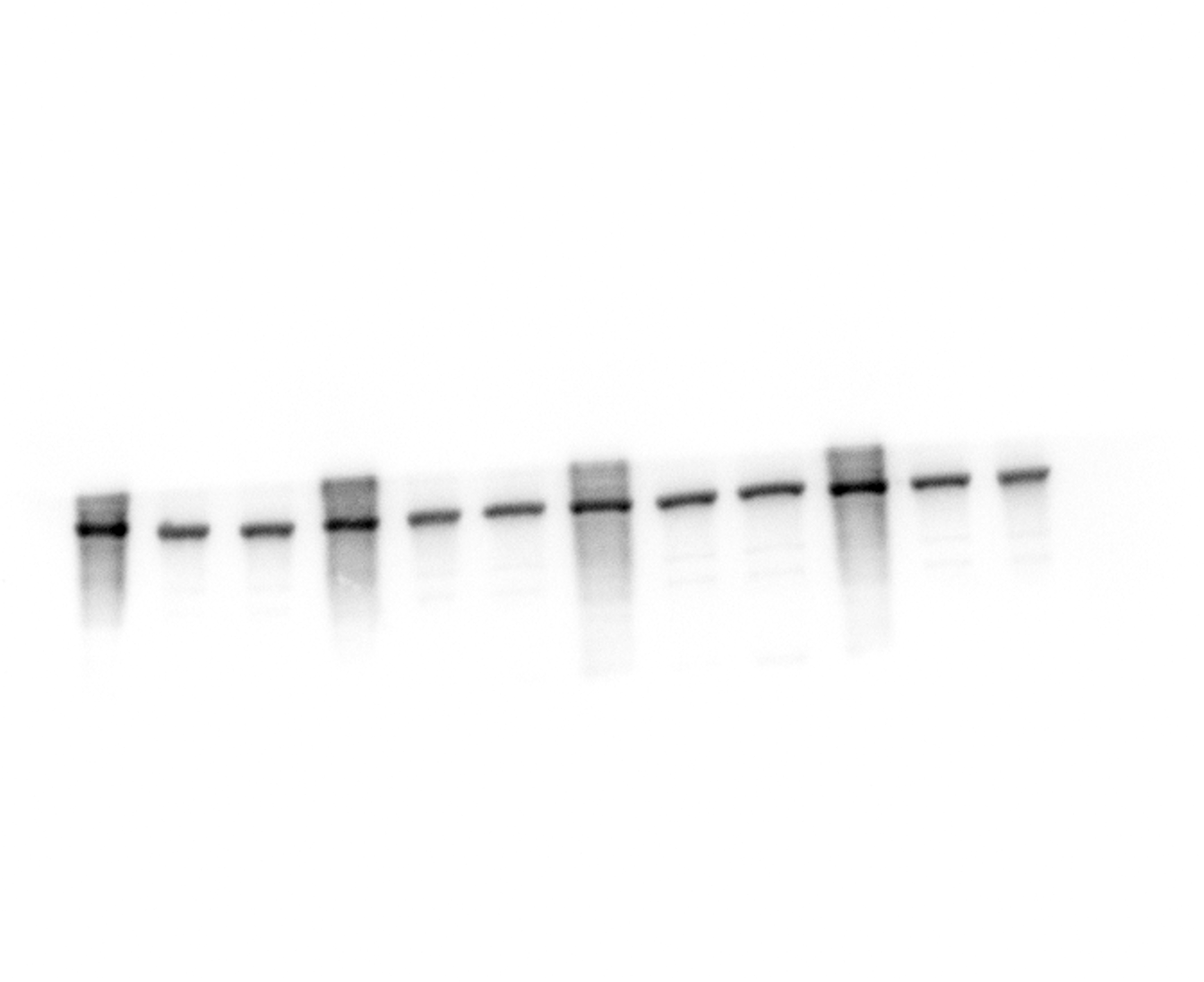

Supplement: Supplementary file 2 — Supporting Information [file ADVS-12-e06225-s001.zip › CD75/GAP-CD75-116OE 116m 116WT TQ TQ TQ -500MS.Tif]

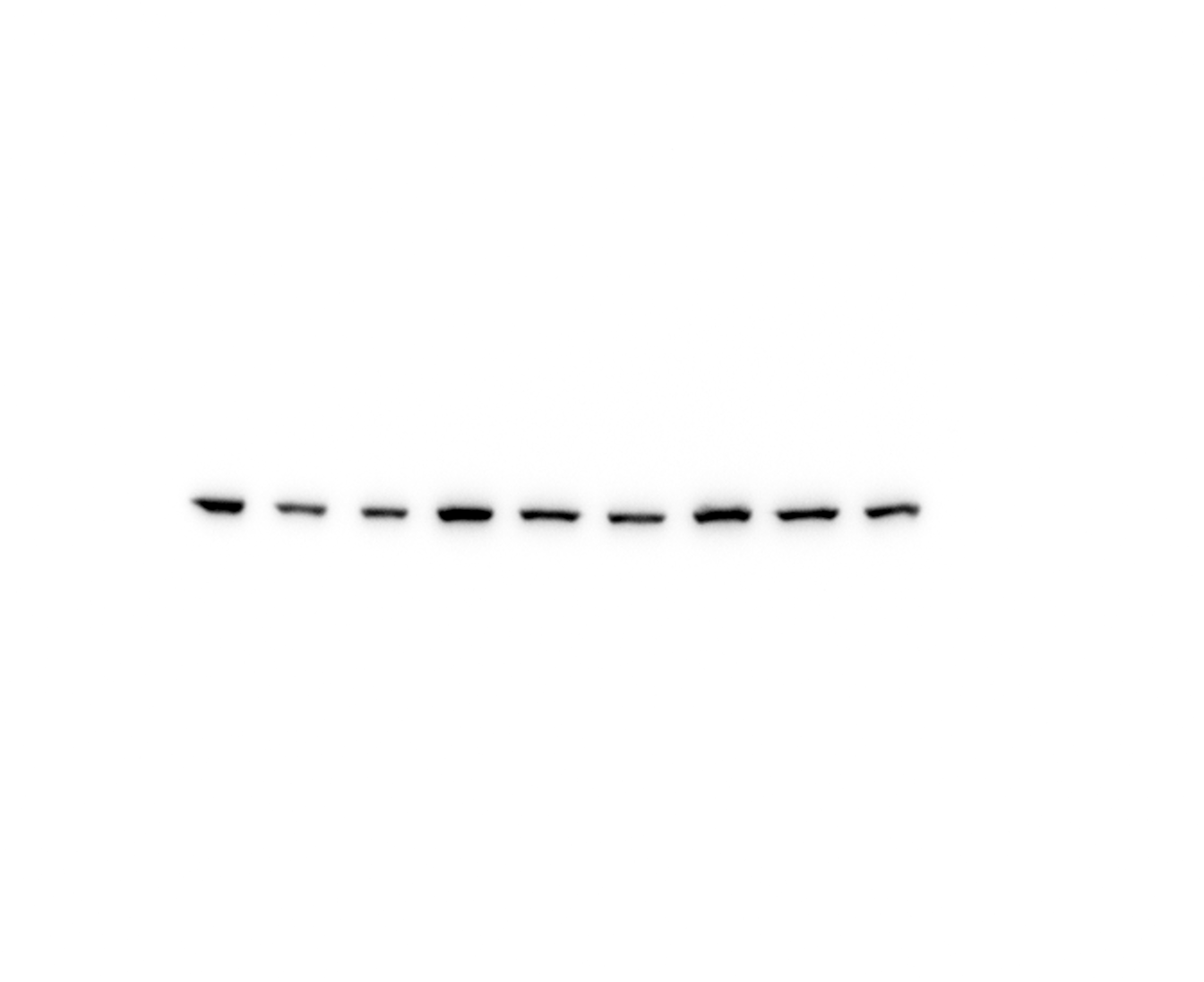

Supplement: Supplementary file 2 — Supporting Information [file ADVS-12-e06225-s001.zip › CD75/GAP-H H1 H2 H H1 H2 H H1 H2-800MS.Tif]

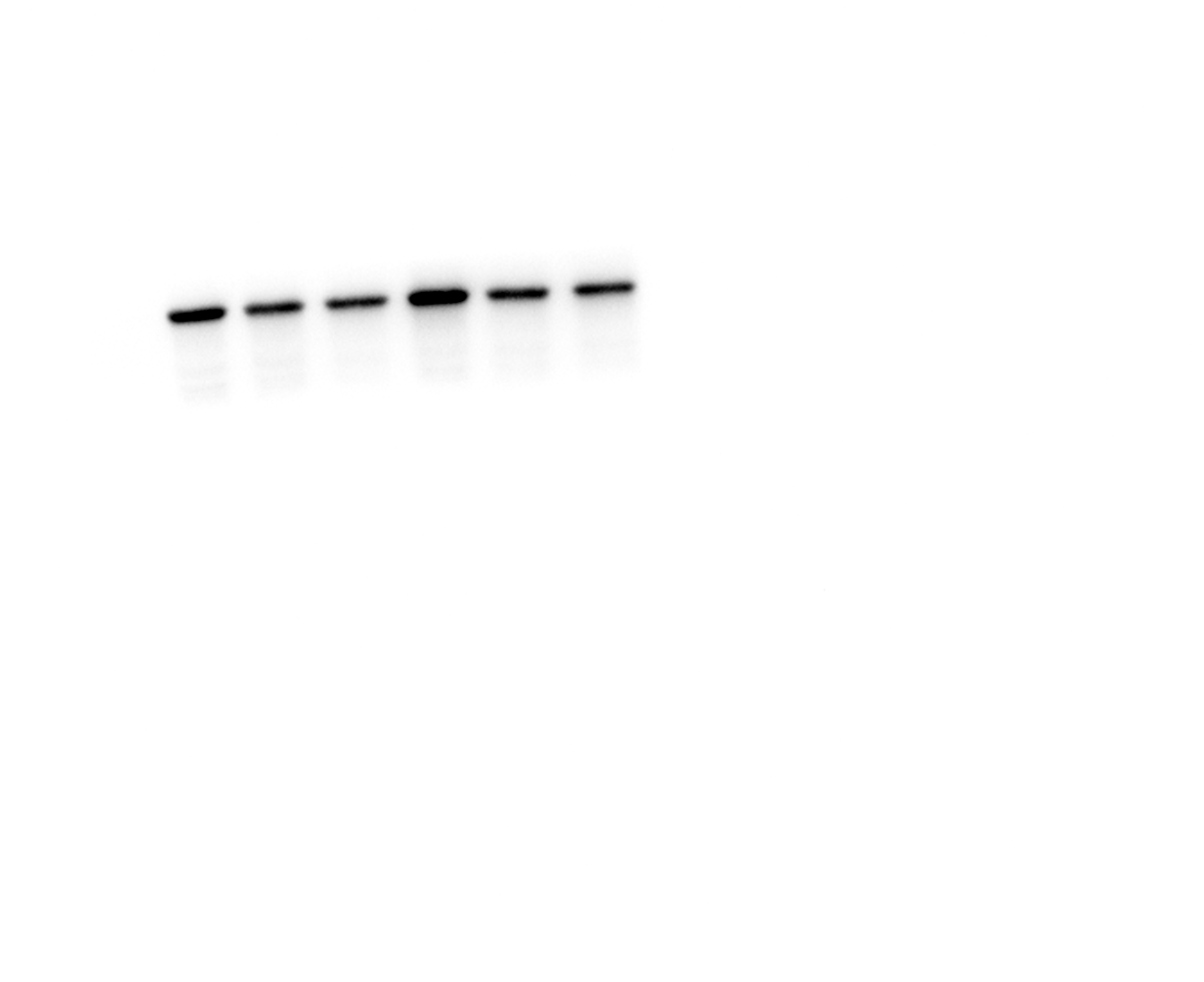

Supplement: Supplementary file 2 — Supporting Information [file ADVS-12-e06225-s001.zip › CD75/GAP-H H1 H2 H H1 H2-1S.Tif]

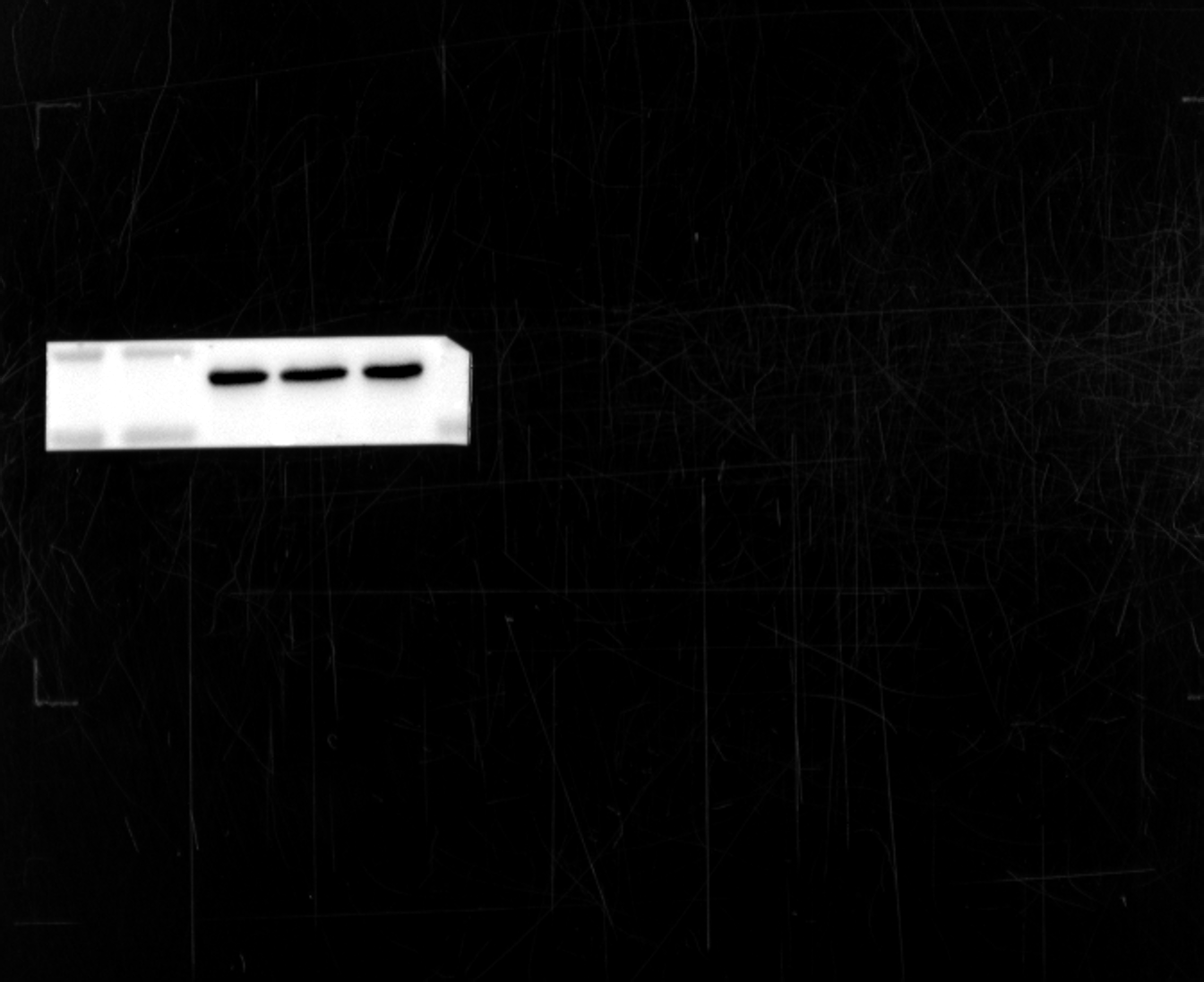

Supplement: Supplementary file 2 — Supporting Information [file ADVS-12-e06225-s001.zip › CD75/GAP-HWT H1 H2-M.Tif]

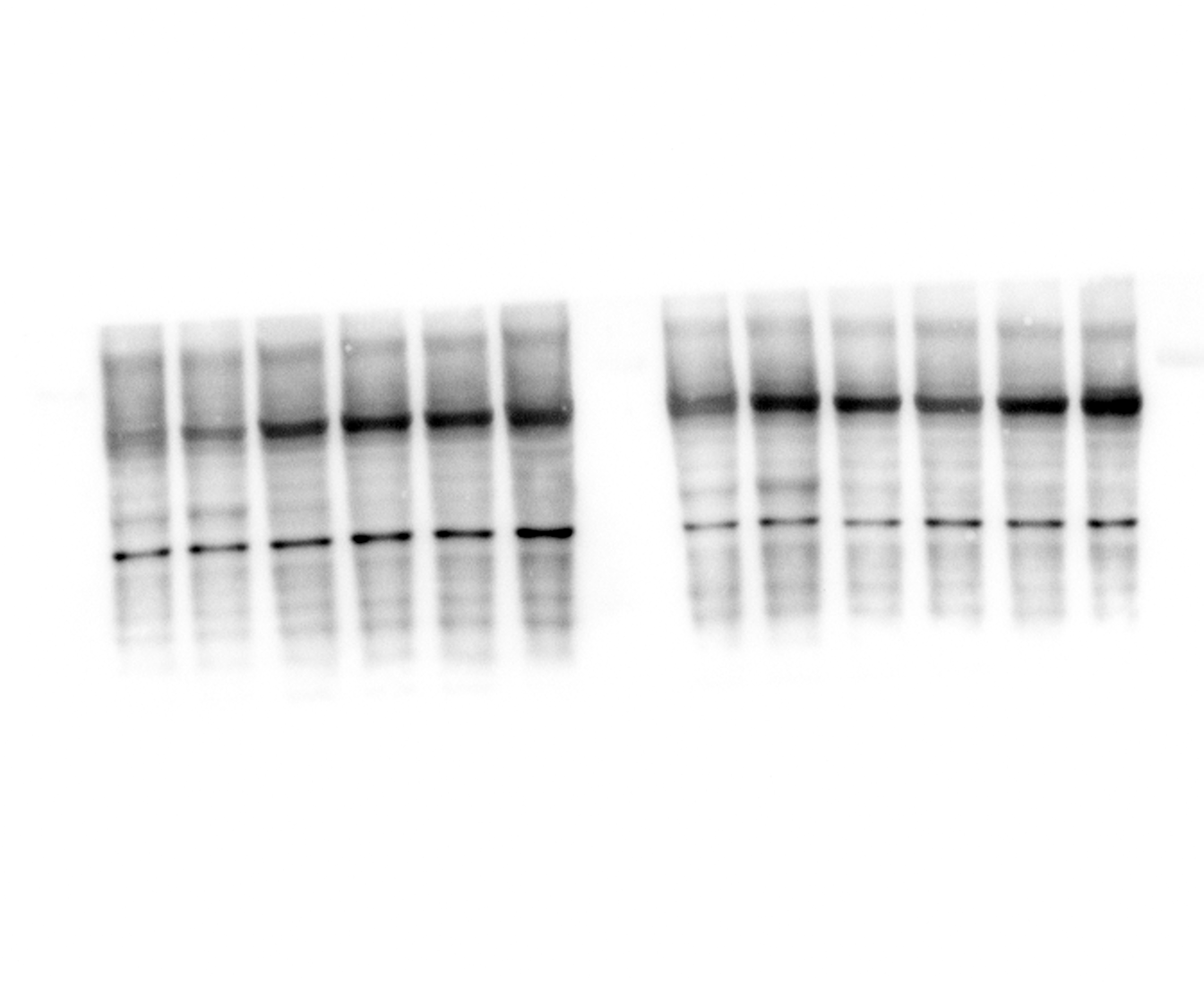

Supplement: Supplementary file 2 — Supporting Information [file ADVS-12-e06225-s001.zip › CHX/1-AB-PDL1-CHX-116SH1 24 12 6 3 1 0 M 116WT 24 12 6 3 1 0-2S--1.Tif]

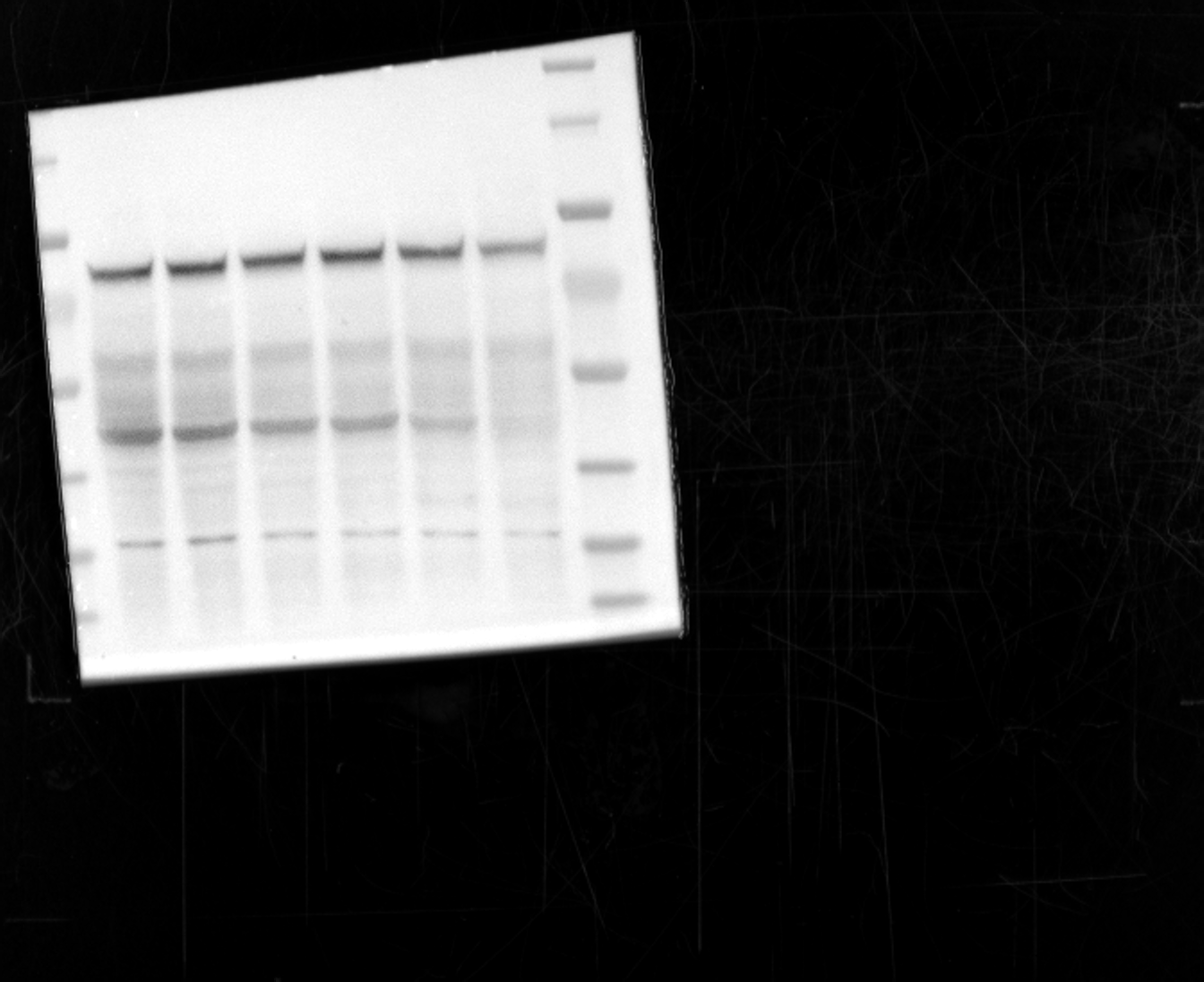

Supplement: Supplementary file 2 — Supporting Information [file ADVS-12-e06225-s001.zip › CHX/1-ab-PDL1-CHX-116SH2-0 1 3 6 12 24-M.Tif]

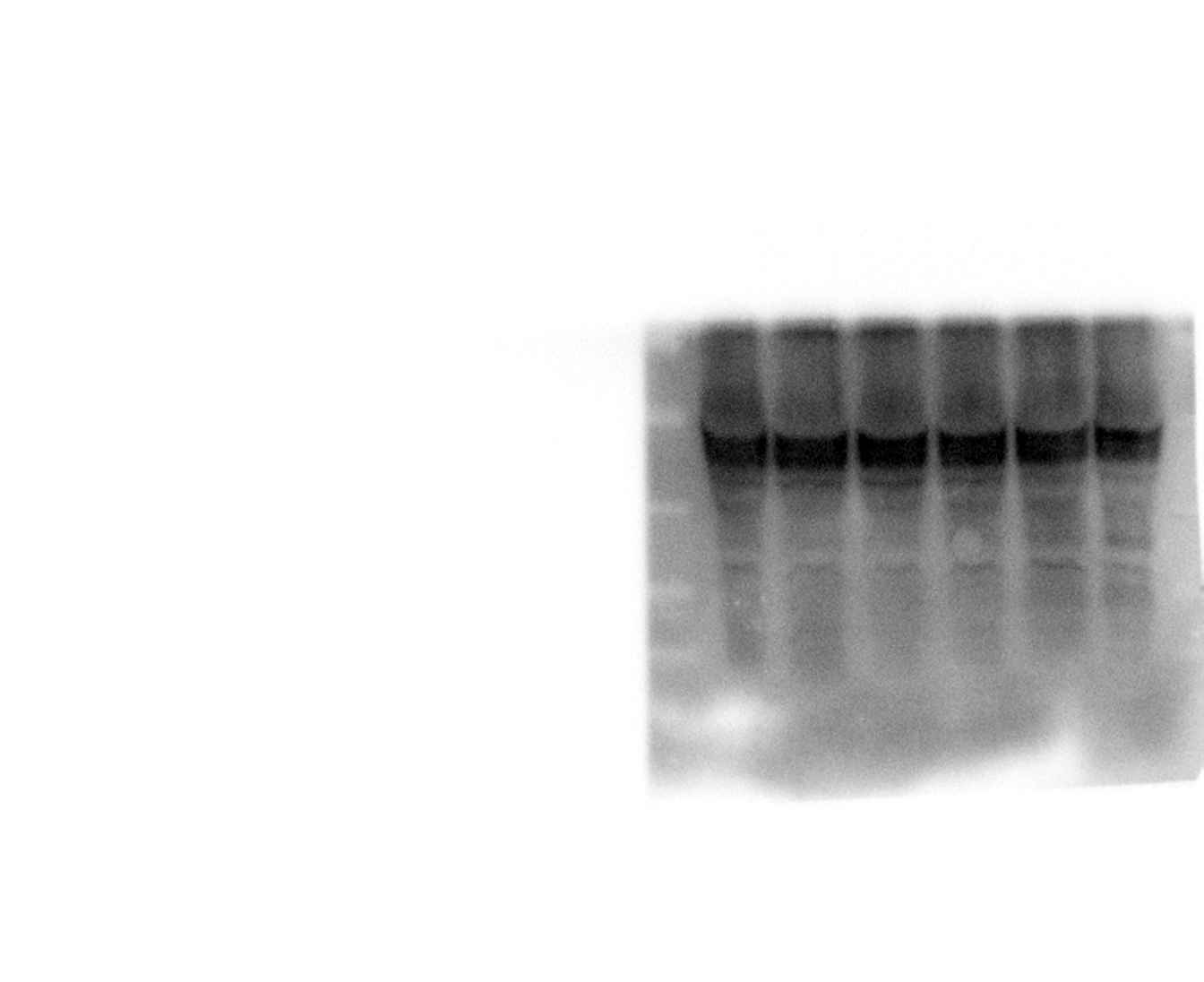

Supplement: Supplementary file 2 — Supporting Information [file ADVS-12-e06225-s001.zip › CHX/1-CHX HWT .Tif]

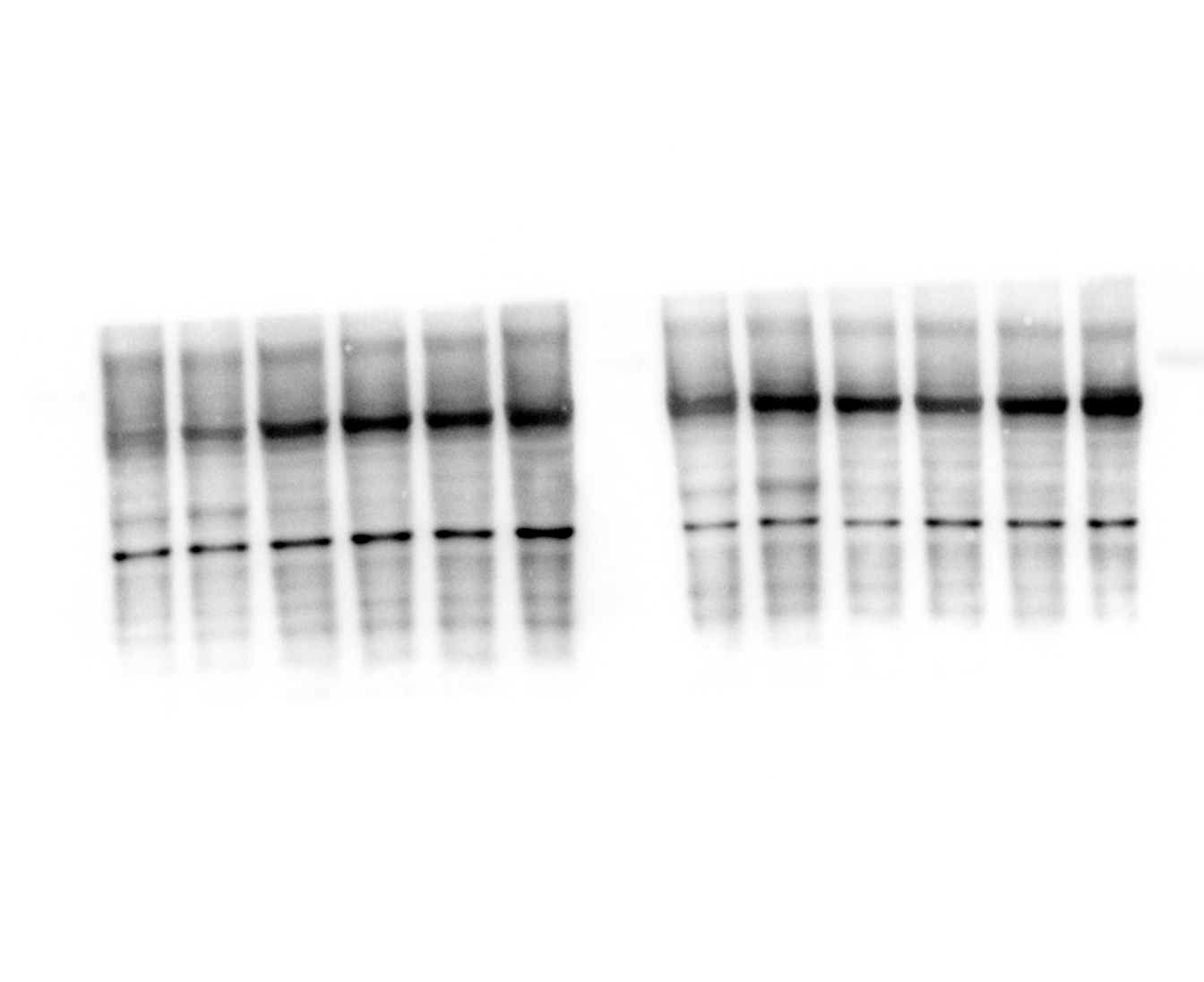

Supplement: Supplementary file 2 — Supporting Information [file ADVS-12-e06225-s001.zip › CHX/1-CHX-116SH1 M 116WT .Tif]

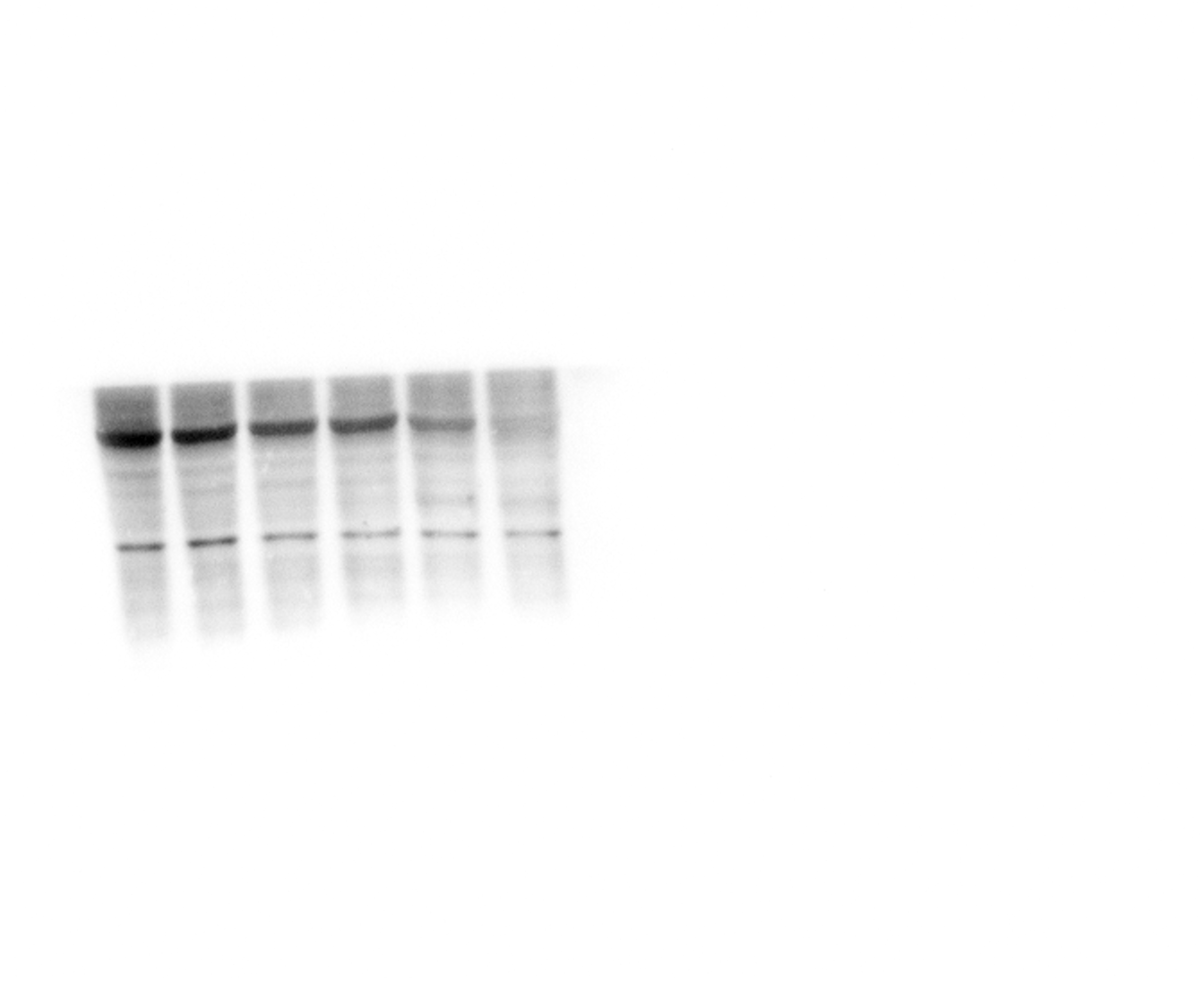

Supplement: Supplementary file 2 — Supporting Information [file ADVS-12-e06225-s001.zip › CHX/1-CHX-116SH2 .Tif]

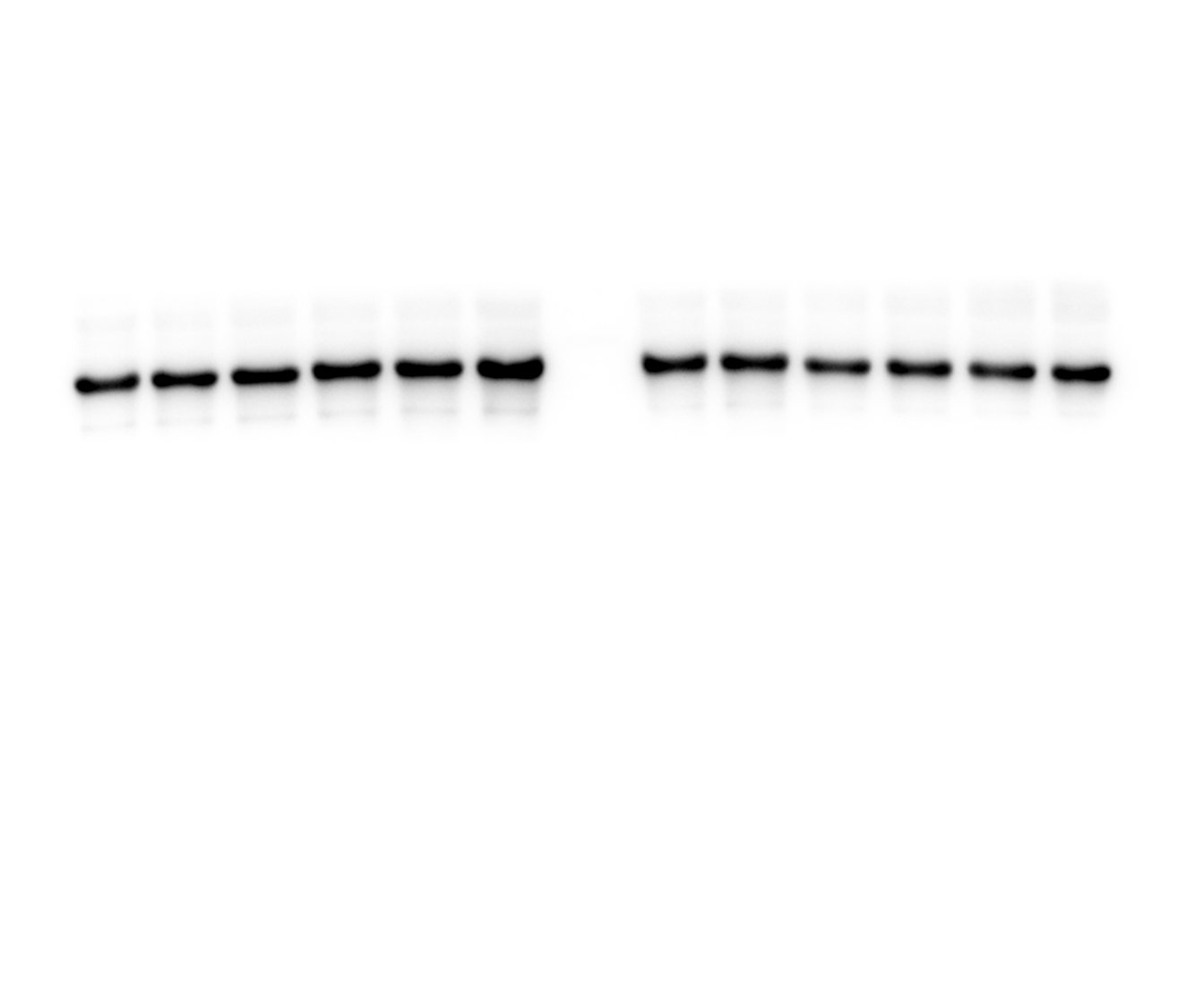

Supplement: Supplementary file 2 — Supporting Information [file ADVS-12-e06225-s001.zip › CHX/1-GAP-116SH1 M 116WT .Tif]

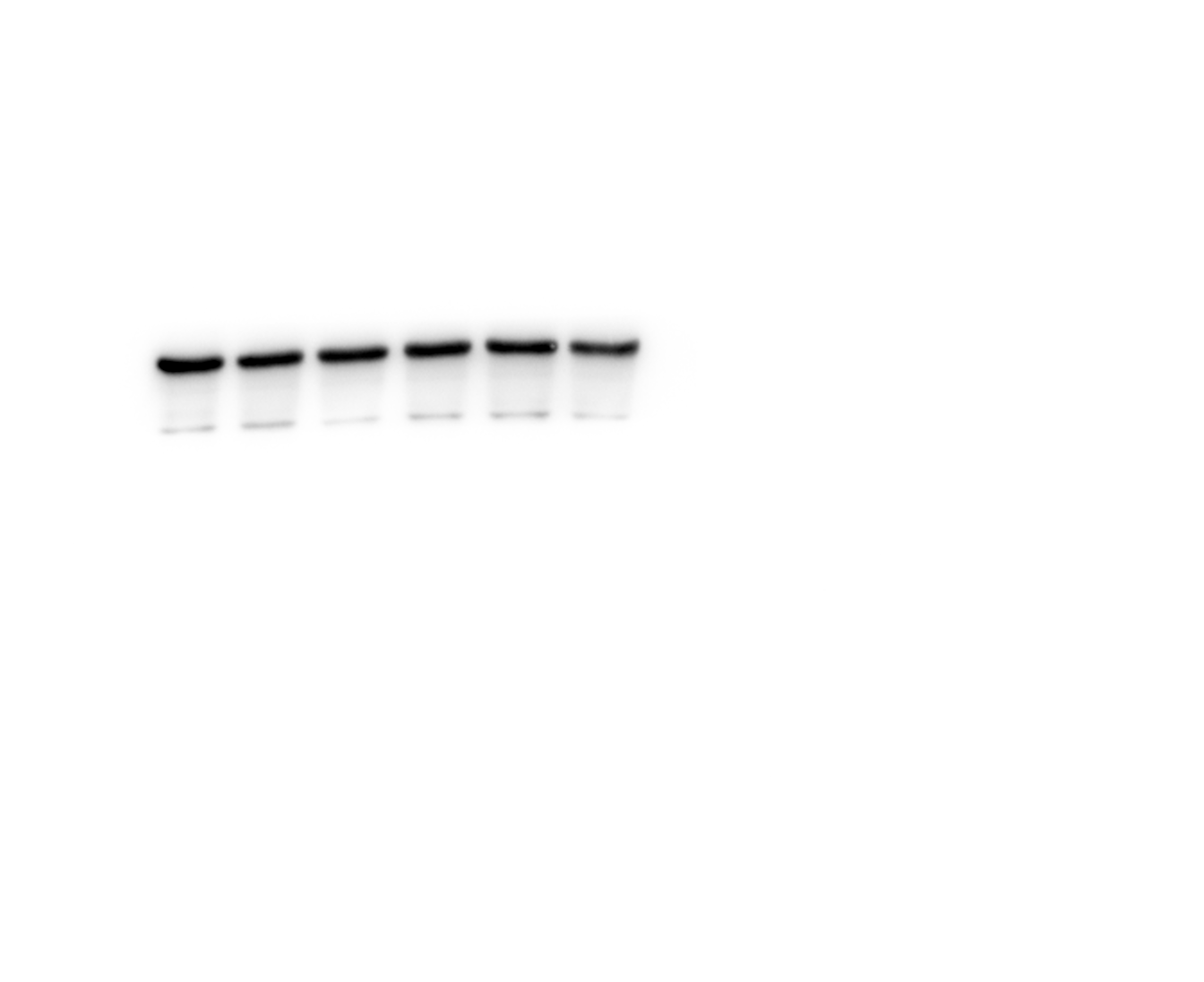

Supplement: Supplementary file 2 — Supporting Information [file ADVS-12-e06225-s001.zip › CHX/1-GAP-116SH2 .Tif]

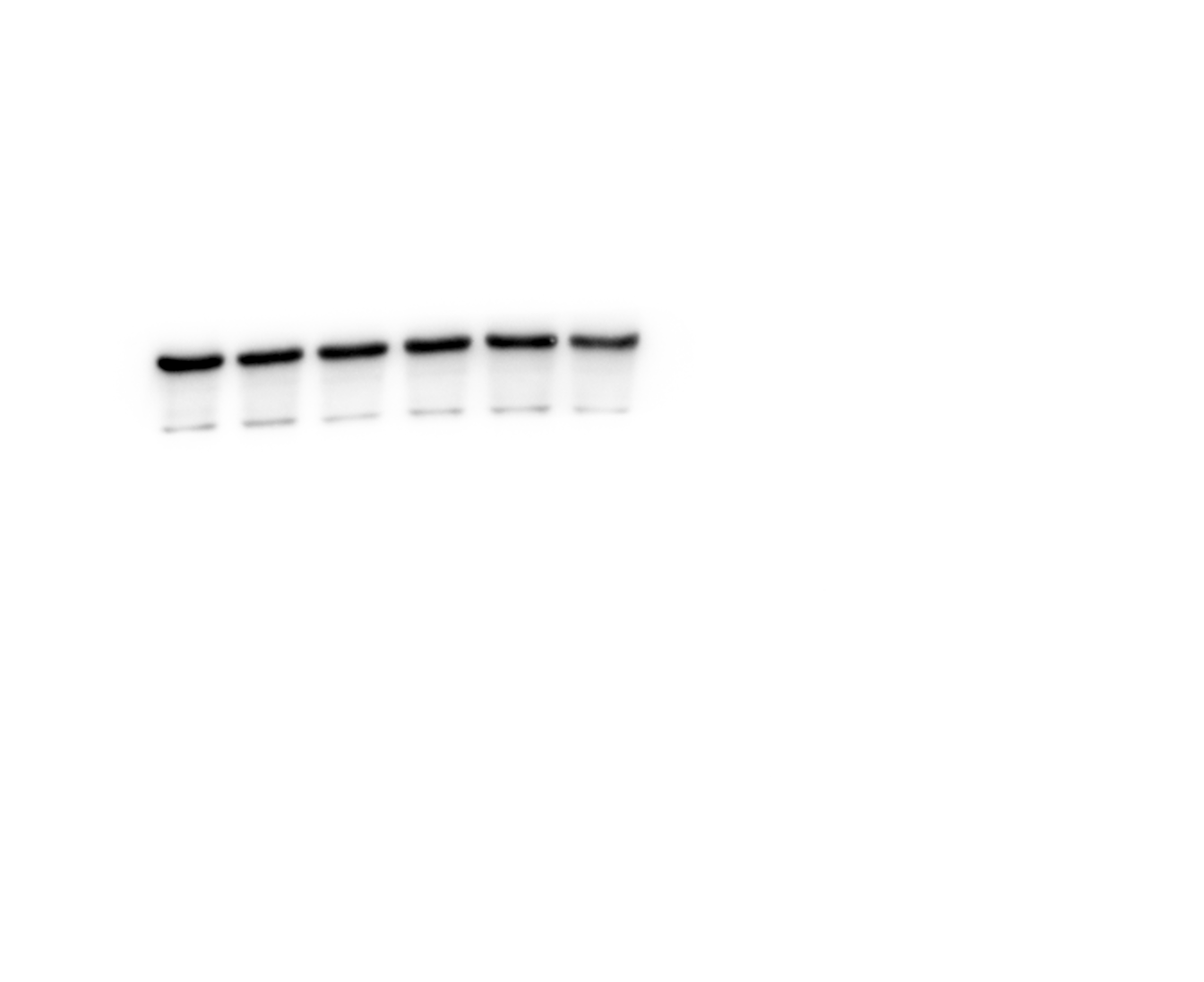

Supplement: Supplementary file 2 — Supporting Information [file ADVS-12-e06225-s001.zip › CHX/1-GAP-116SH2-0 1 3 6 12 24-1S-2.Tif]

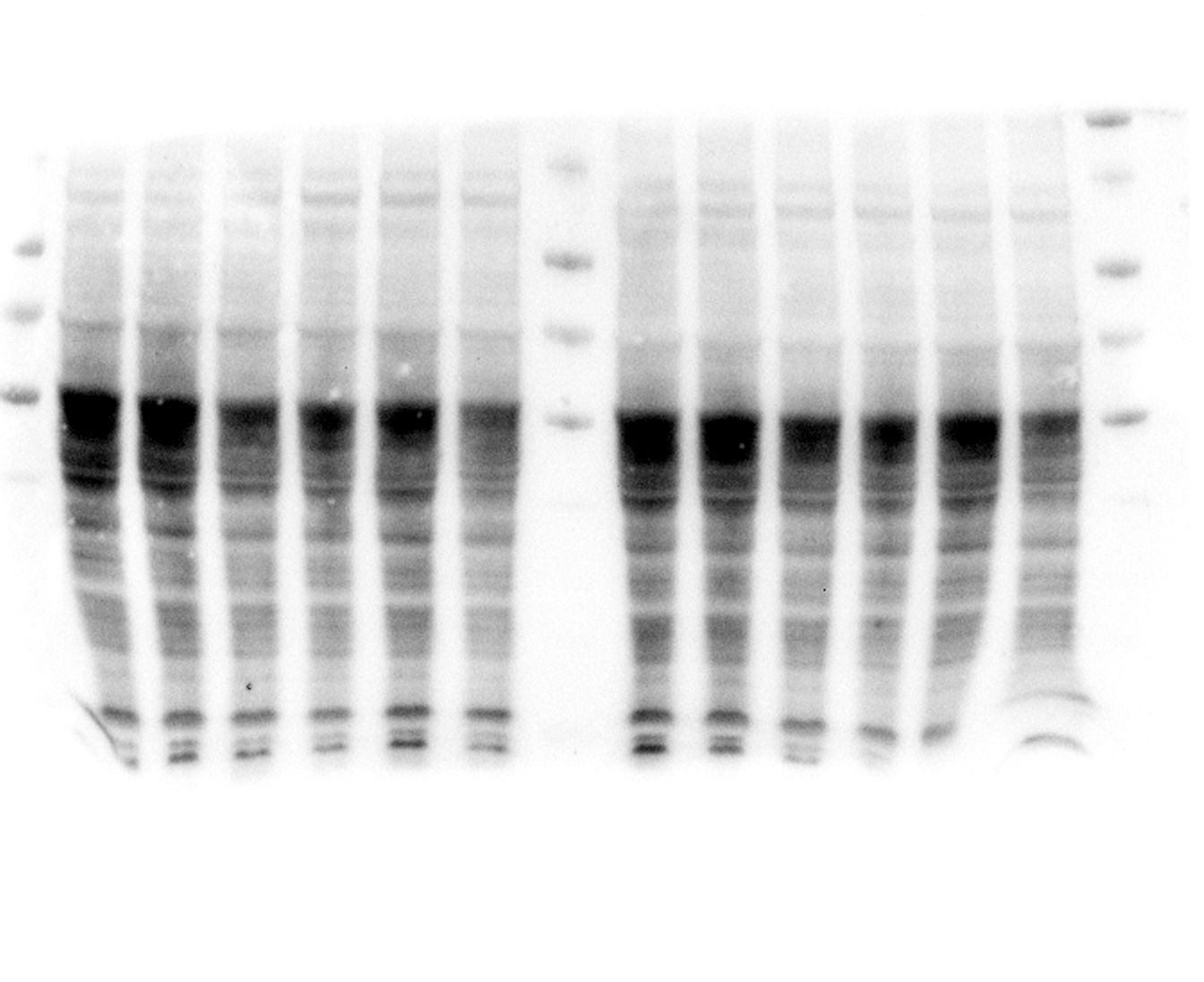

Supplement: Supplementary file 2 — Supporting Information [file ADVS-12-e06225-s001.zip › CHX/1CHX-H1- TQ .Tif]

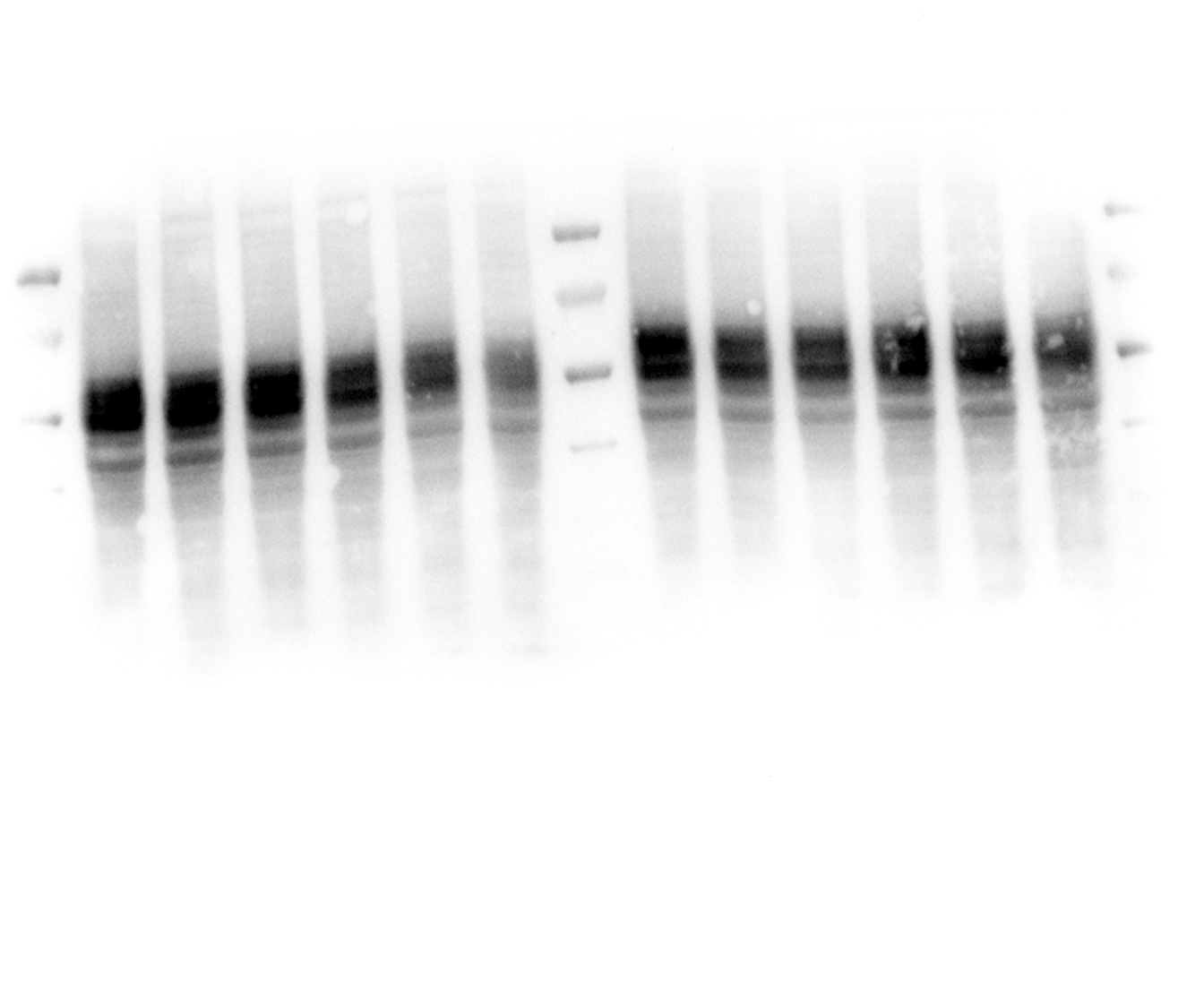

Supplement: Supplementary file 2 — Supporting Information [file ADVS-12-e06225-s001.zip › CHX/1CHX-H2 -----.Tif]

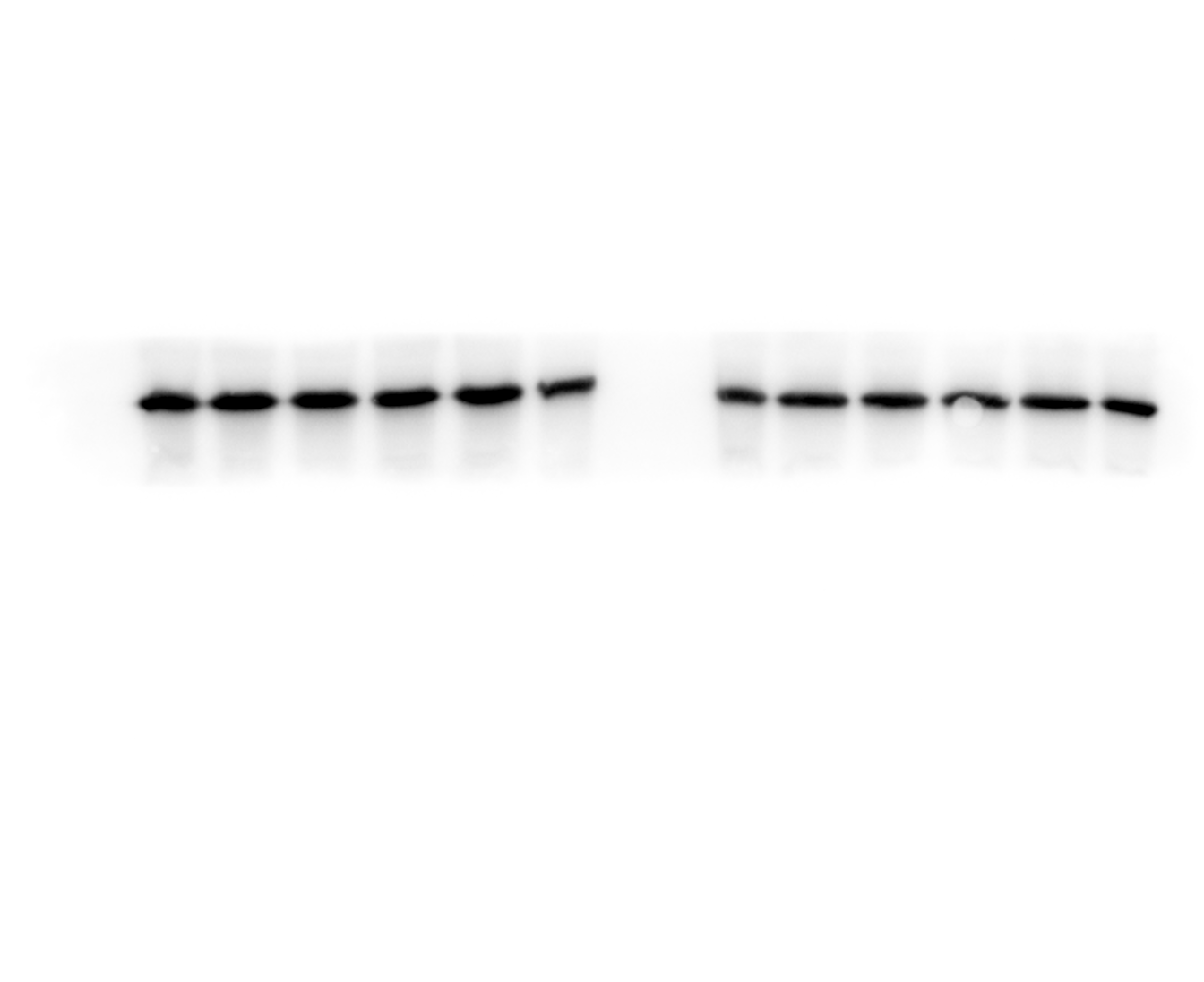

Supplement: Supplementary file 2 — Supporting Information [file ADVS-12-e06225-s001.zip › CHX/1GAP-----------M HWT .Tif]

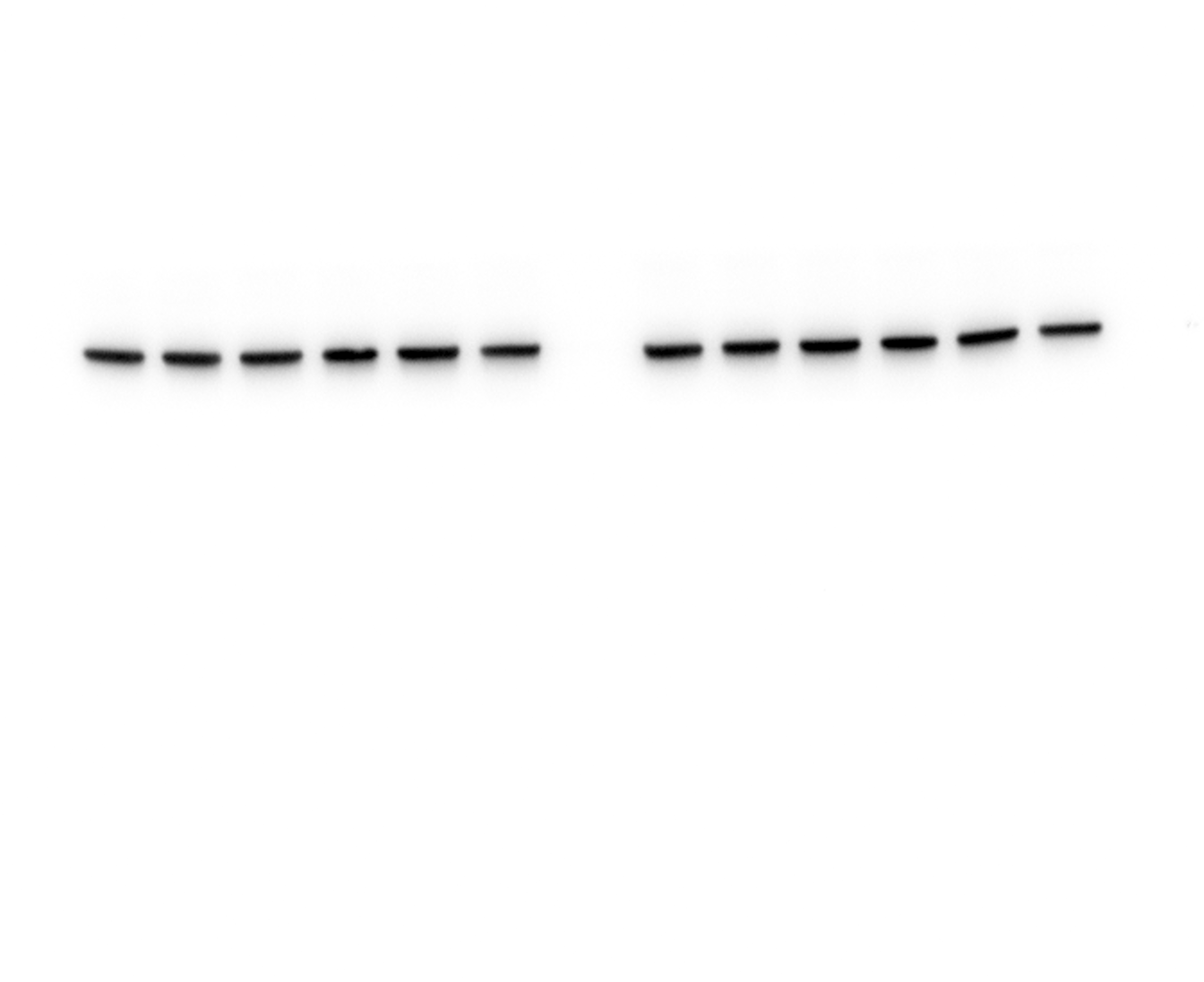

Supplement: Supplementary file 2 — Supporting Information [file ADVS-12-e06225-s001.zip › CHX/1GAP-H1-TQ .Tif]

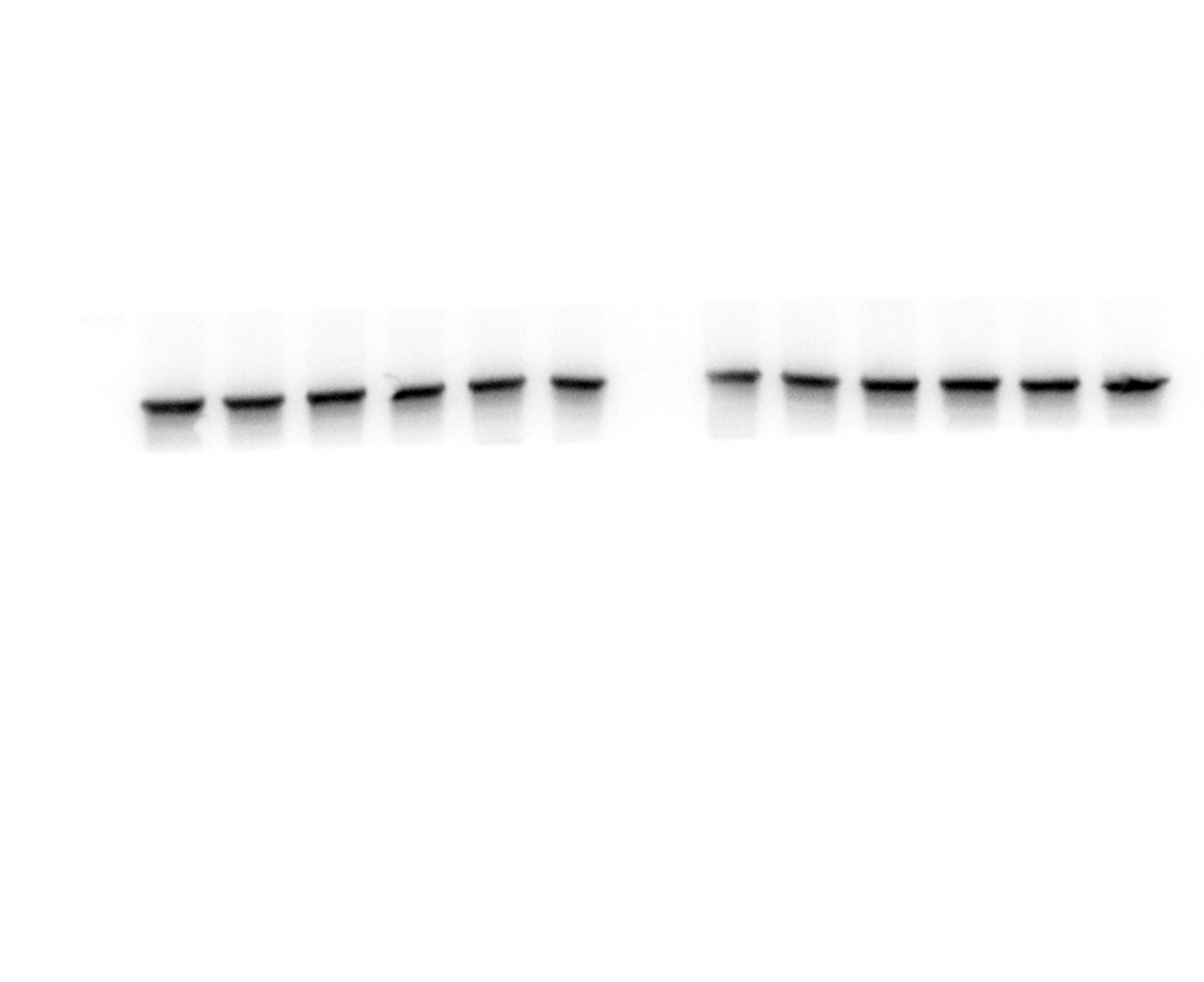

Supplement: Supplementary file 2 — Supporting Information [file ADVS-12-e06225-s001.zip › CHX/1GAP-H2-TQ .Tif]

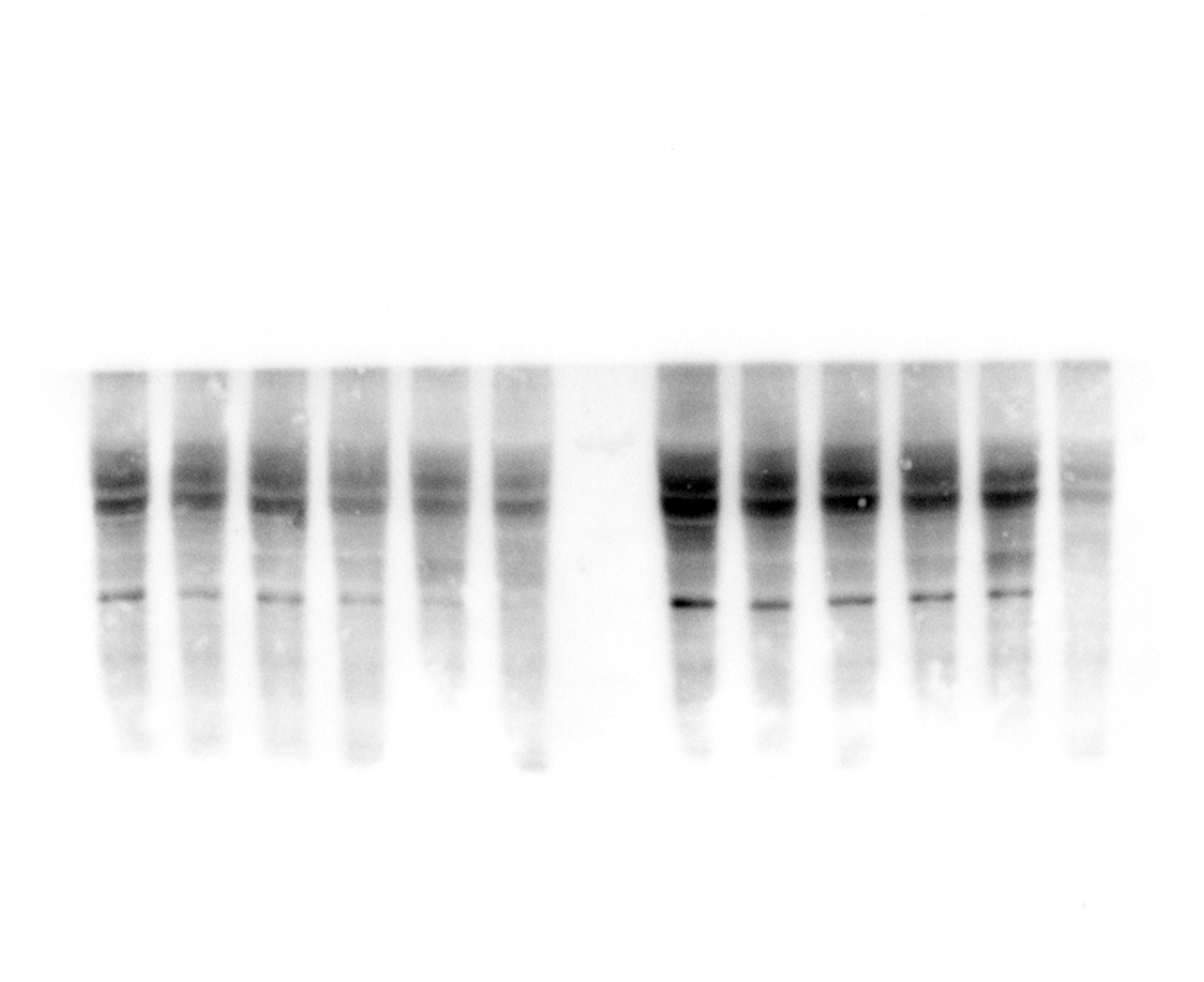

Supplement: Supplementary file 2 — Supporting Information [file ADVS-12-e06225-s001.zip › CHX/2-AB-PDL1-CHX-116SH1 0 1 3 6 12 24 M 116SH2 0 1 3 6 12 24-4S-1.Tif]

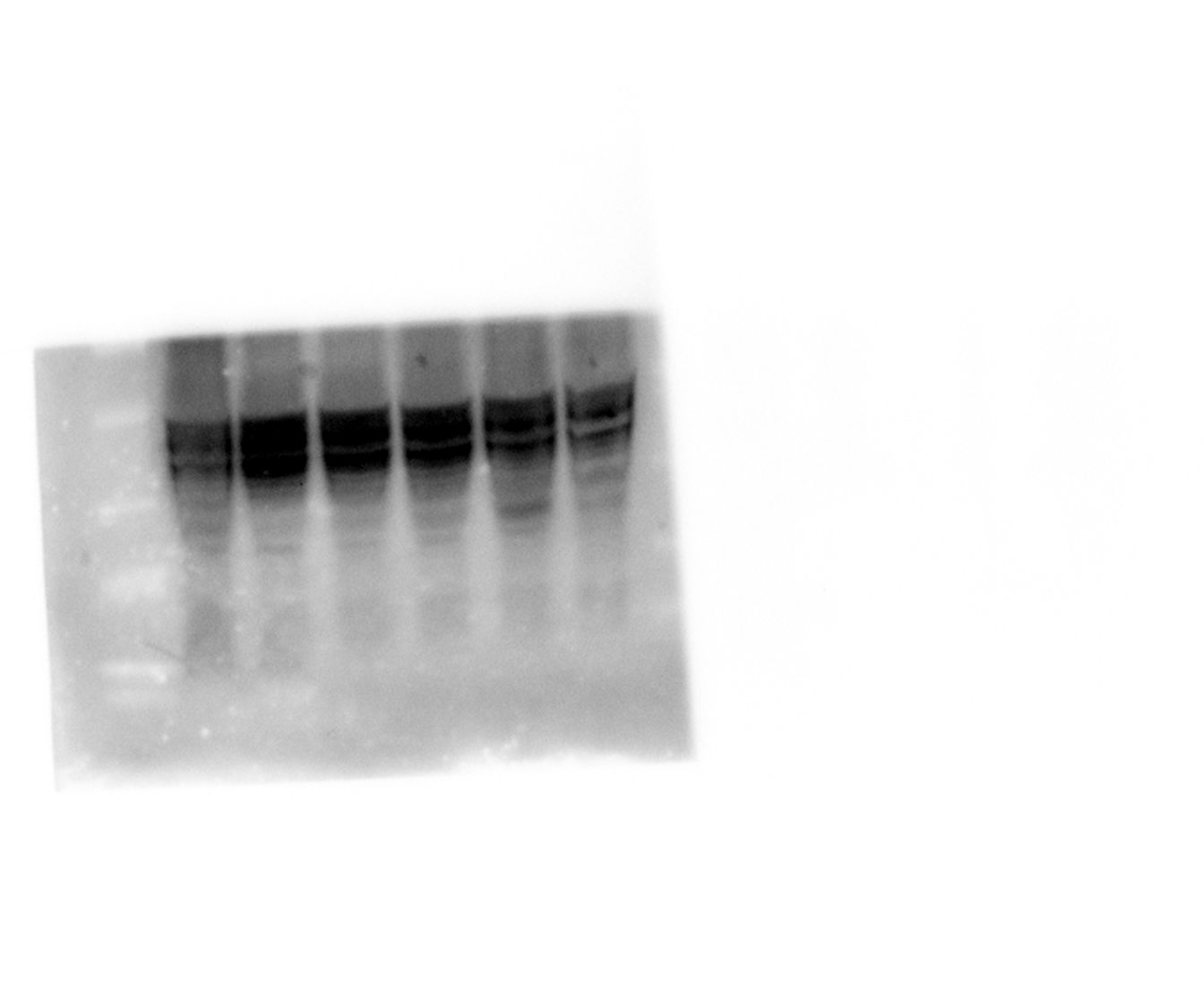

Supplement: Supplementary file 2 — Supporting Information [file ADVS-12-e06225-s001.zip › CHX/2-CHX 116WT .Tif]

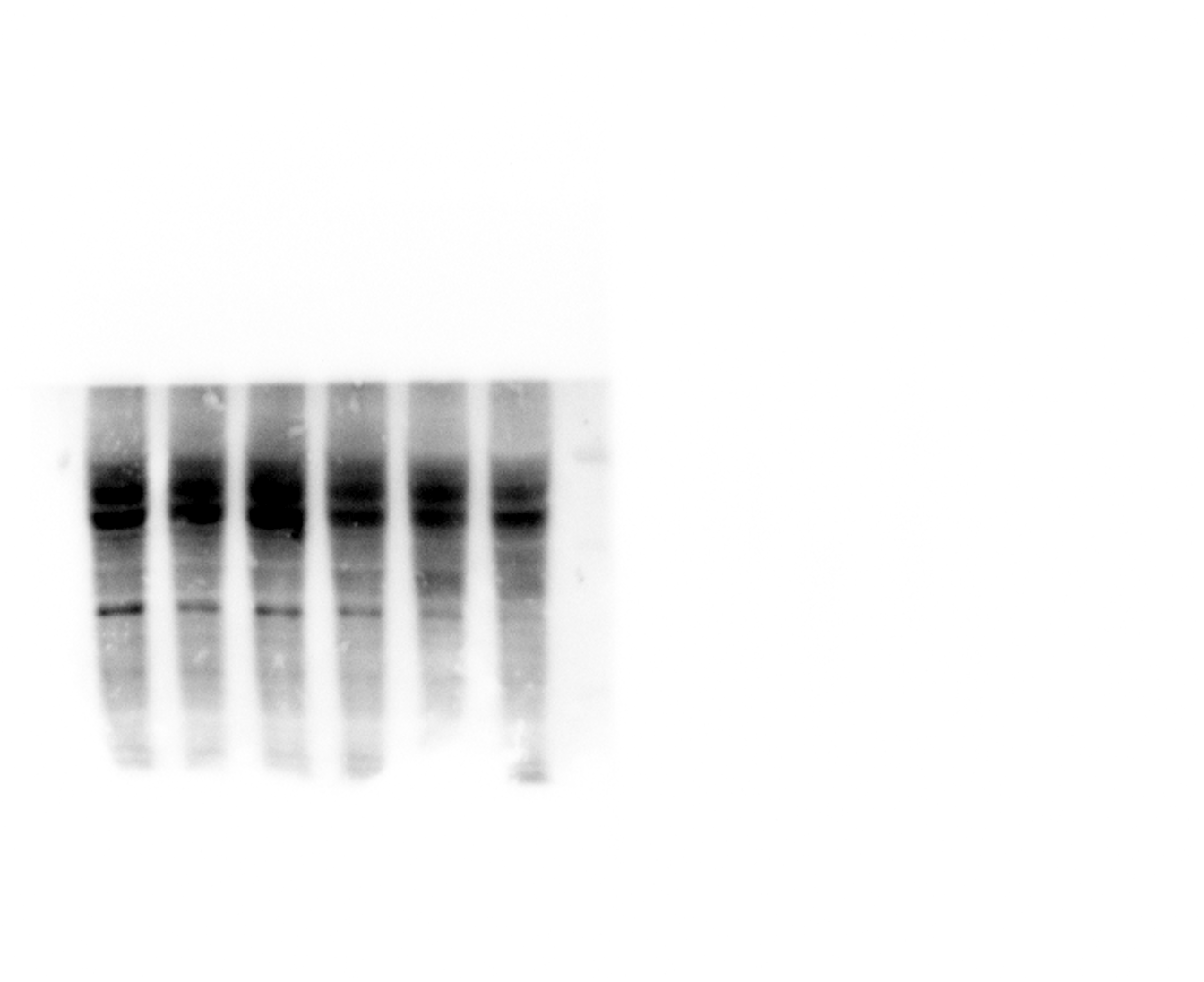

Supplement: Supplementary file 2 — Supporting Information [file ADVS-12-e06225-s001.zip › CHX/2-CHX-116SH1 .Tif]

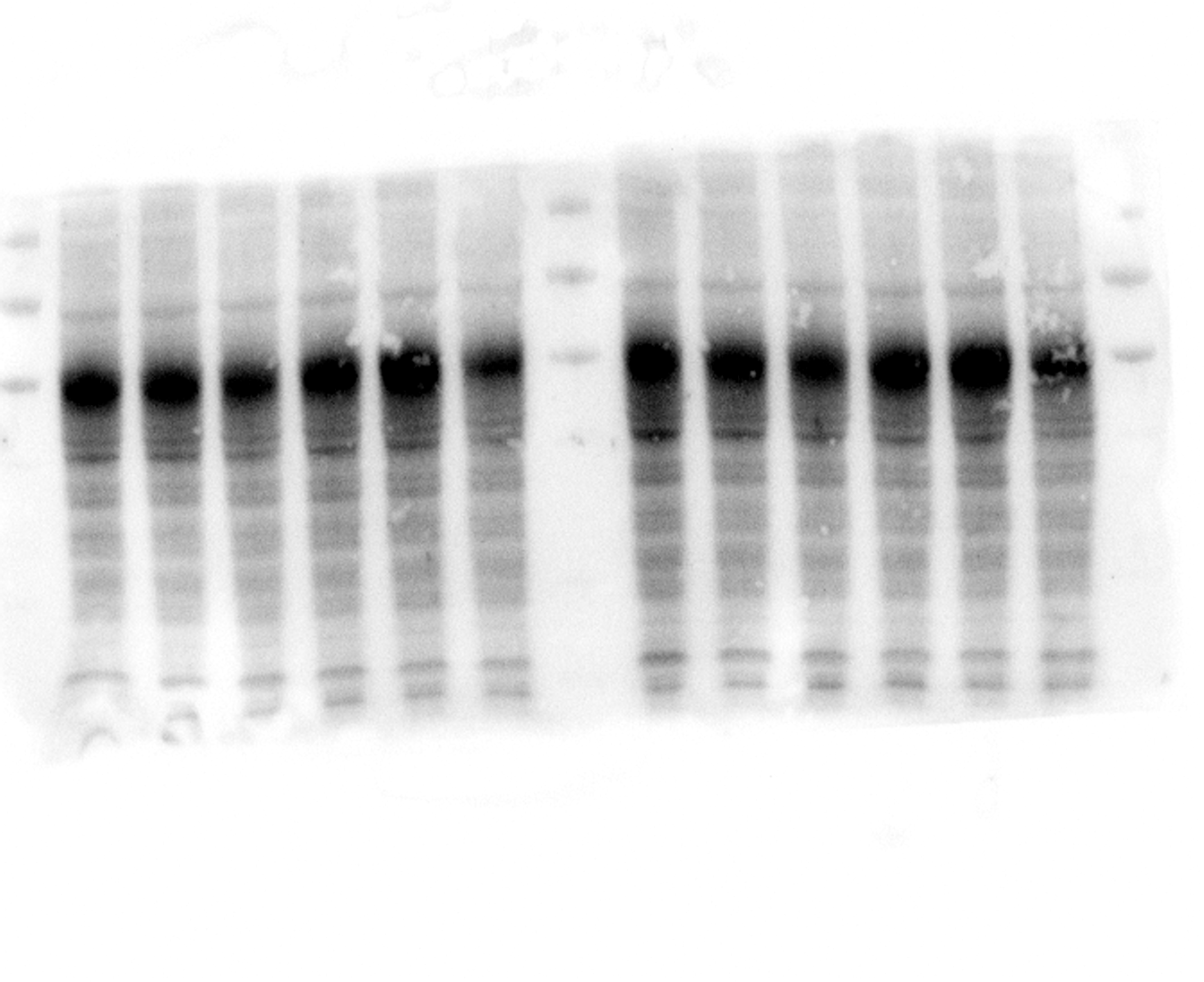

Supplement: Supplementary file 2 — Supporting Information [file ADVS-12-e06225-s001.zip › CHX/2-CHX-HWT -TQ-17.Tif]

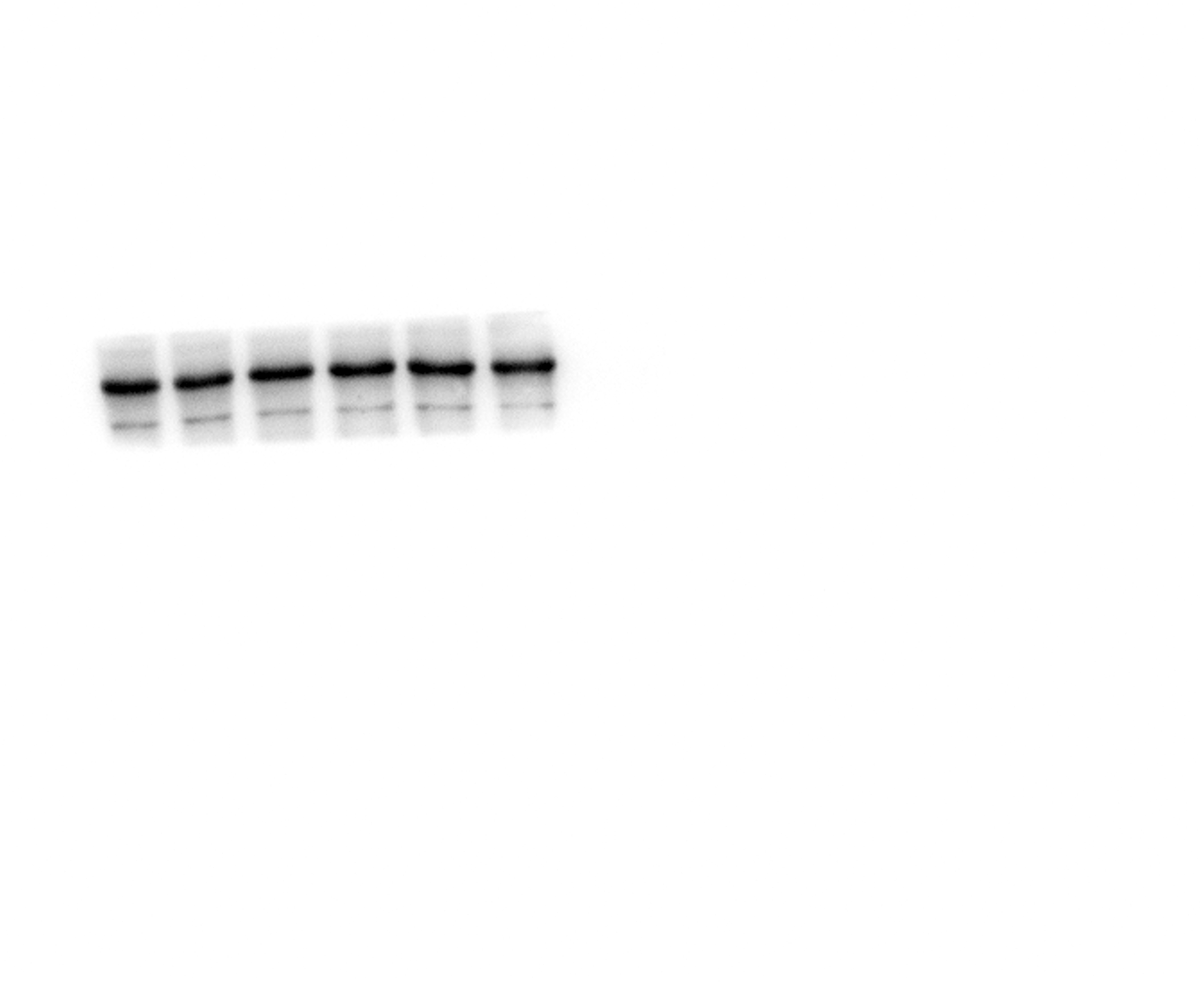

Supplement: Supplementary file 2 — Supporting Information [file ADVS-12-e06225-s001.zip › CHX/2-GAP-116SH2-0 1 3 6 12 24-1S-4.Tif]

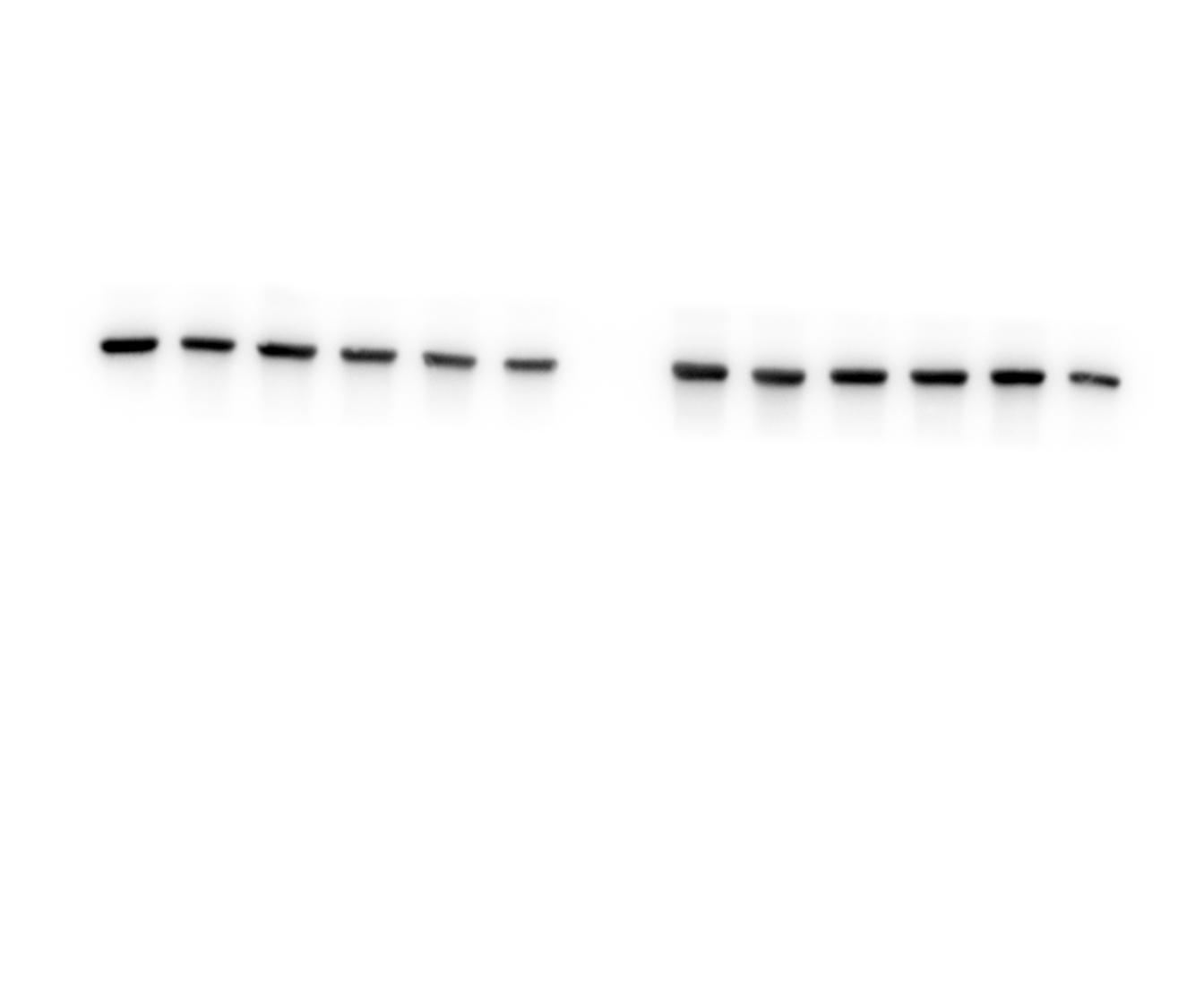

Supplement: Supplementary file 2 — Supporting Information [file ADVS-12-e06225-s001.zip › CHX/2-GAP-CHX-116SH1 0 1 3 6 12 24 M 116SH2 0 1 3 6 12 24-1S.Tif]

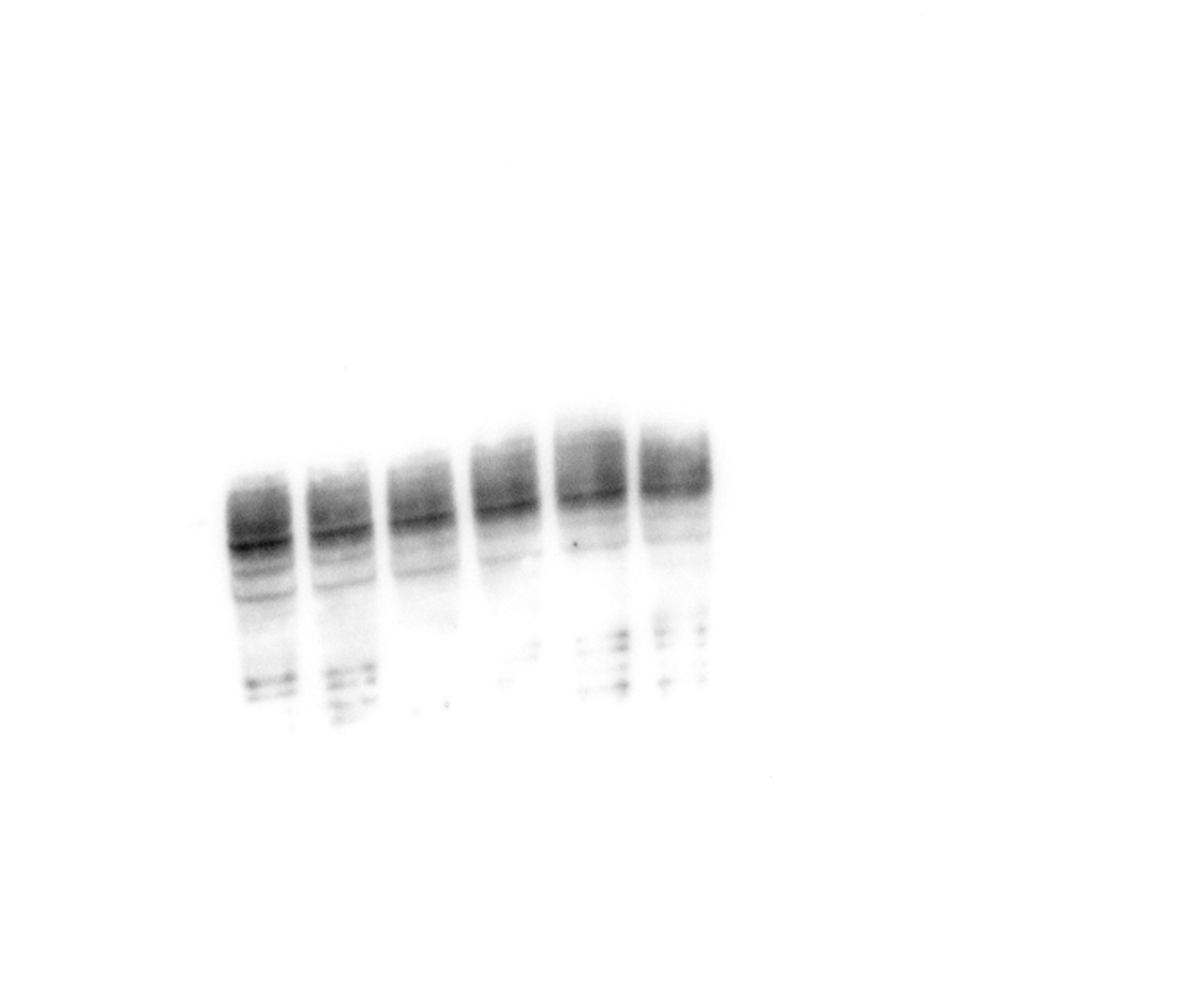

Supplement: Supplementary file 2 — Supporting Information [file ADVS-12-e06225-s001.zip › CHX/2-PDL1-CHX-116SH2-0 1 3 6 12 24-5S-2.Tif]

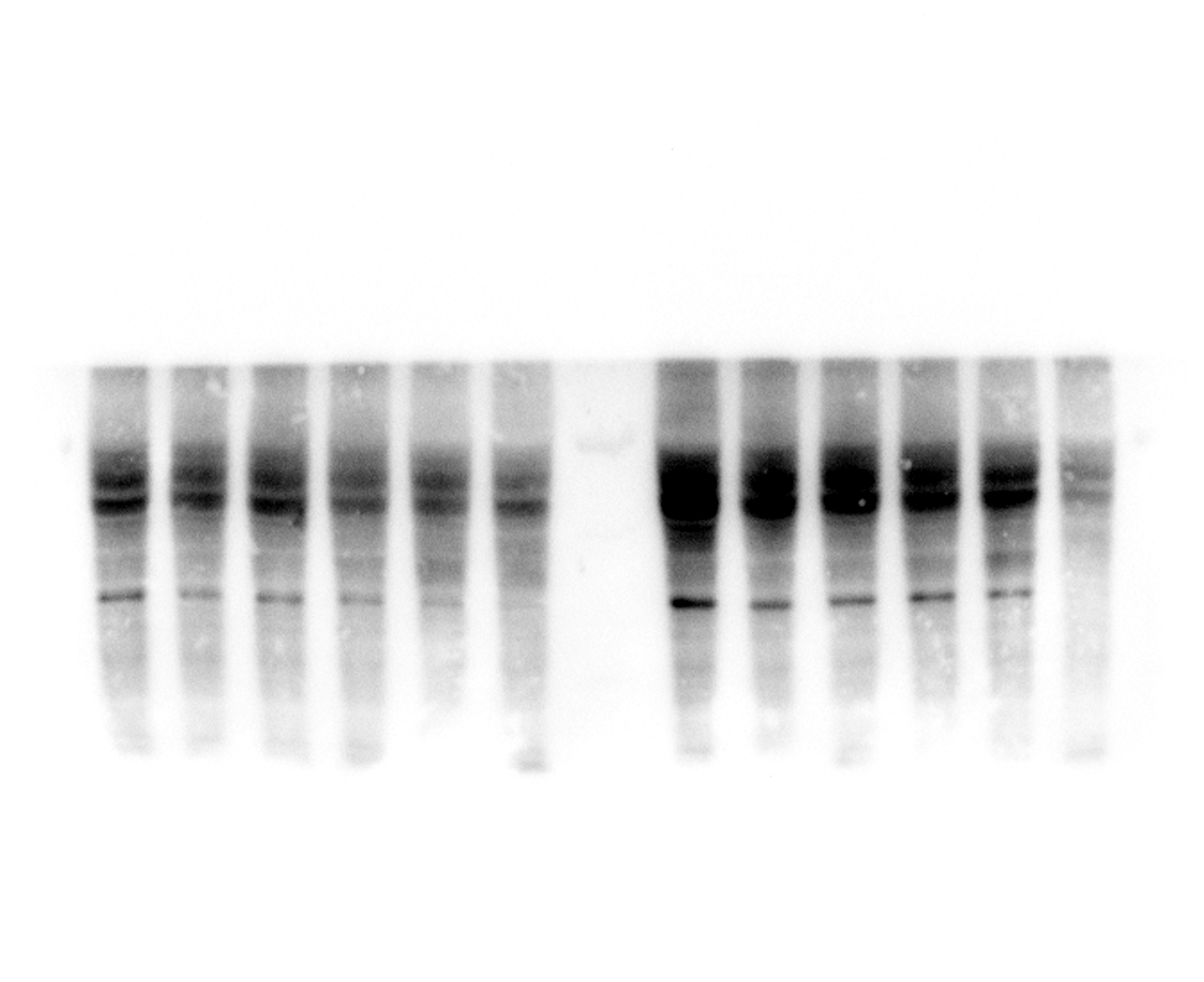

Supplement: Supplementary file 2 — Supporting Information [file ADVS-12-e06225-s001.zip › CHX/2CHX--------- M 116SH2 .Tif]

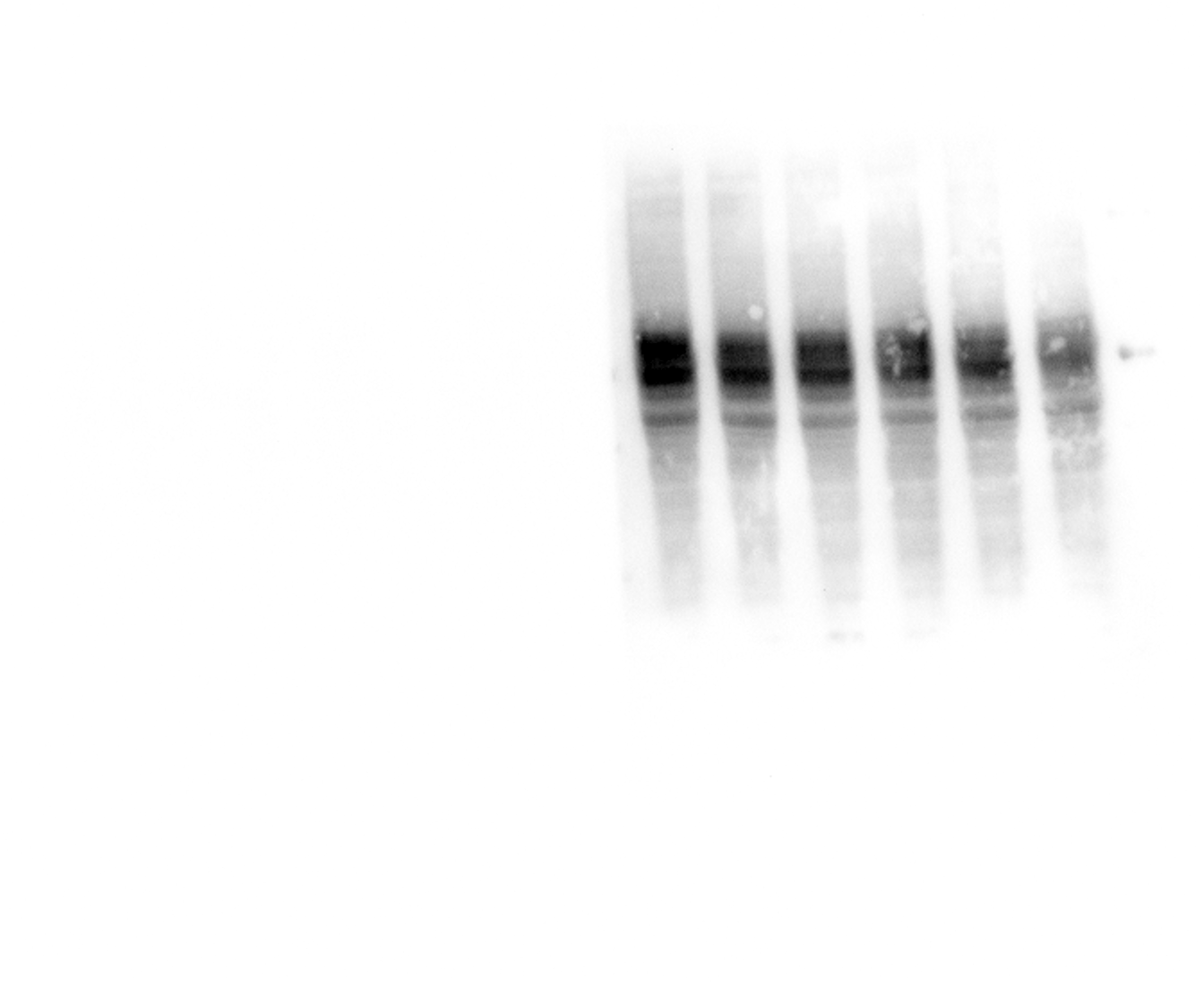

Supplement: Supplementary file 2 — Supporting Information [file ADVS-12-e06225-s001.zip › CHX/2CHX-H2.Tif]

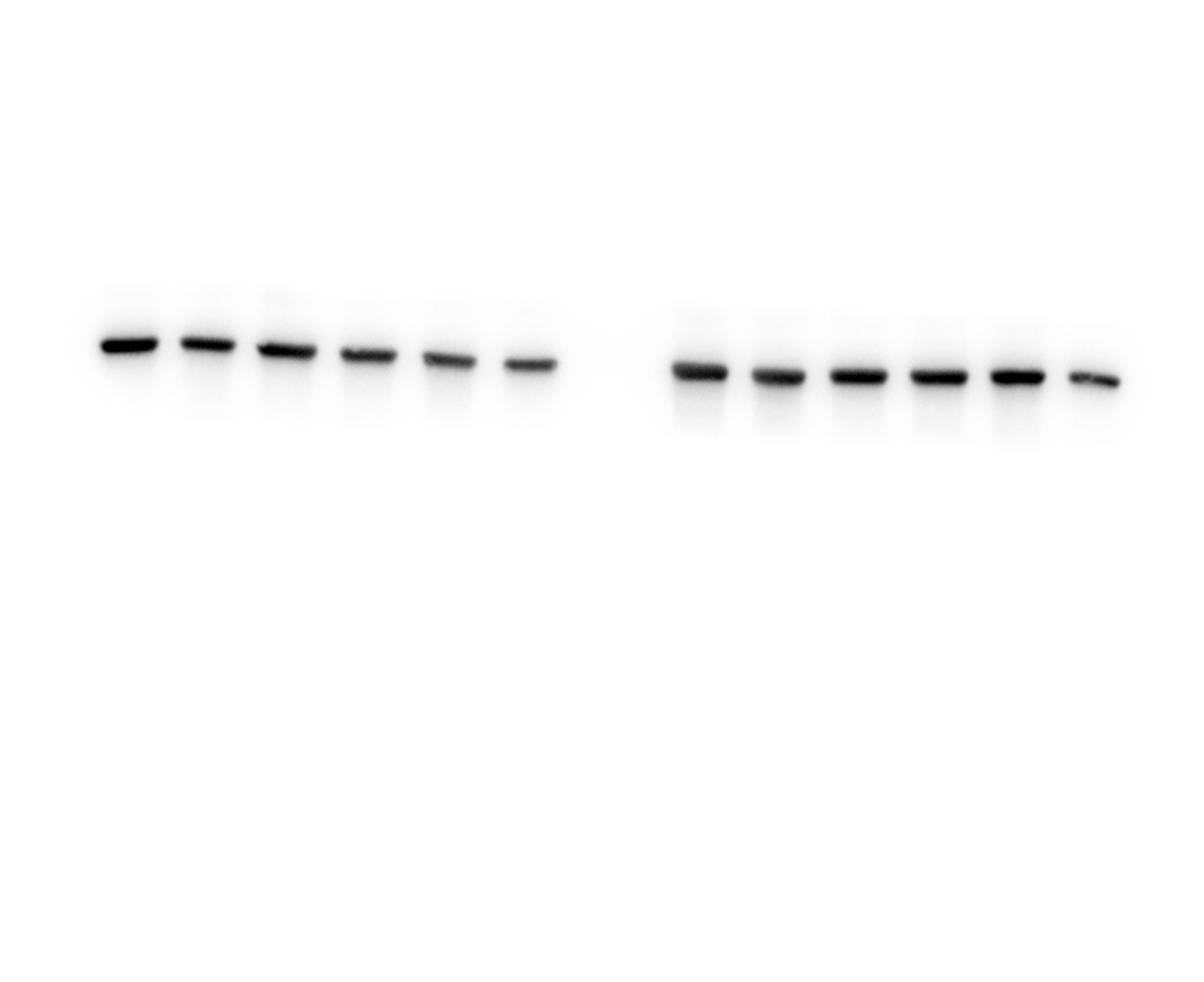

Supplement: Supplementary file 2 — Supporting Information [file ADVS-12-e06225-s001.zip › CHX/2GAP-116SH1 M 116SH2 .Tif]

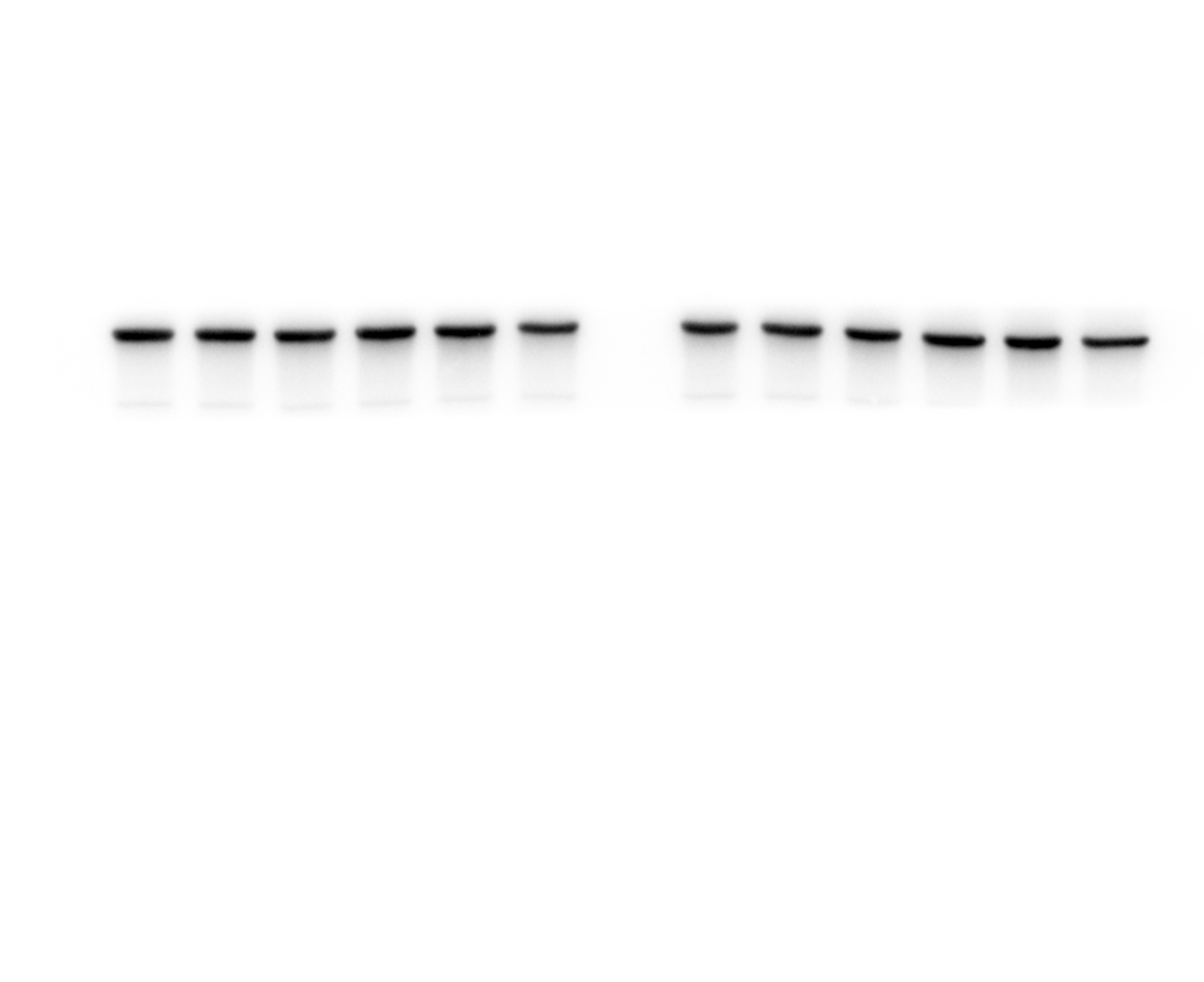

Supplement: Supplementary file 2 — Supporting Information [file ADVS-12-e06225-s001.zip › CHX/2GAP-H-TQ .Tif]

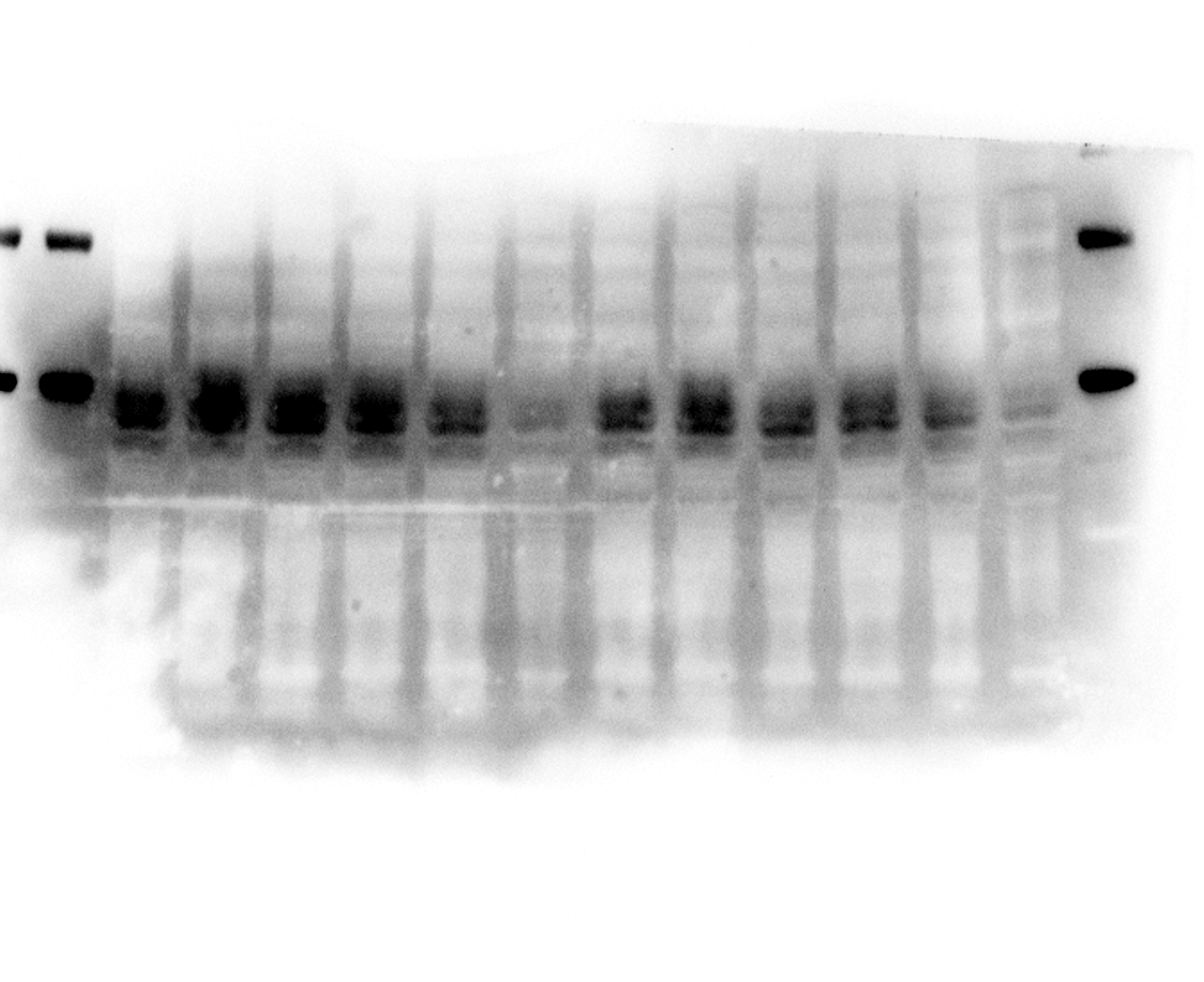

Supplement: Supplementary file 2 — Supporting Information [file ADVS-12-e06225-s001.zip › CHX/3-116-1- 0 1 3 6 12 24 TQ -3S-3.Tif]
